# A randomized, double-blind, active-controlled phase 3 multicenter trial of rHSA for cirrhotic ascites

**DOI:** 10.1038/s41392-026-02951-7

**Published:** 2026-09-10

**Authors:** Jidong Jia, Yajun An, Xiaojuan Ou, Weijia Duan, Yongzhong Li, Yong Deng, Jun Xie, Qinghai Wang, Xing Li, Zhongwei Pan, Yawen Luo, Lijia Wang, Xuanfang Zhong, Dajun Wu, Zhongji Meng, Zhirong Liu, Zonghua Chen, Liang Hong, Yali Zong, Yingmei Tang, Lihua Zhong, Cheng Wo, Bin Xu, Baolian Shu, Fei Shi, Guangming Li, Guangming Xiao, Fengqian Song, Guoqiang Zhang, Haiyan Chen, Aijun Liao, Hongjian Wei, Junyi Li, Xuan An, Fangfang Li, Chenwei Pan, Jie Pan, Zhanzhou Lin, Wei Hou, Hong Li, Jianyong Chen, Xiaoping Wu, Shuilin Sun, Yueming Shen, Xiaoqu Zhu, Bin Chen, Yuehong Zeng, Xingguo Xiao, Jiawei Geng, Heng Zhang, Caiyan Zhao, Lu Chen, Qinfang Wu, Yang Li, Xiongxiang Liu, Xueqiang Jiang, Hui Tian, Zhongkuo Li, Ming Zhao, Huai Li, Xiaobo Xiong, Rui Yao, Li Shao, Fengzhu Liu, Lirong Fu, Youwei Liao, Tao Guan, Xinhua Luo, Wenfang Yuan, Hongbo Ji, Xiaolin Zhou, Yongzhang Luo

**Affiliations:** 1 Liver Research Center, Beijing Friendship Hospital, Capital Medical University, State Key Laboratory of Digestive Health, National Clinical Research Center for Digestive Diseases, Beijing, China; 2 Shenzhen Protgen Co., Ltd., Shenzhen, Guangdong China; 3 The National Engineering Research Center for Protein Technology, Beijing, China; 4 Hunan University of Medicine General Hospital, Huaihua, Hunan China; 5 Pingxiang No. 2 People’s Hospital, Pingxiang, Jiangxi China; 6 First Affiliated Hospital of Gannan Medical University, Ganzhou, Jiangxi China; 7 The First People’s Hospital of Changde City, Changde, Hunan China; 8 Pingxiang People’s Hospital, Pingxiang, Jiangxi China; 9 Meihekou Central Hospital, Meihekou, Jilin China; 10 Affiliated Hospital of Zunyi Medical University, Zunyi, Guizhou China; 11 The First Affiliated Hospital of Henan University, Kaifeng, Henan China; 12 Huizhou First Hospital, Huizhou, Guangdong China; 13 Zhuzhou Central Hospital, Zhuzhou, Hunan China; 14 Taihe Hospital, Hubei University of Medicine, Shiyan, Hubei China; 15 Shandong Public Health Clinical Center, Jinan, Shandong China; 16 The Second People’s Hospital of Yibin, Yibin, Sichuan China; 17 The Third Affiliated Hospital of Wenzhou Medical University, Wenzhou, Zhejiang China; 18 The Ninth Hospital of Nanchang, Nanchang, Jiangxi China; 19 The Second Affiliated Hospital of Kunming Medical University, Kunming, Yunnan China; 20 The Fourth Affiliated Hospital of Harbin Medical University, Harbin, Heilongjiang China; 21 Jiujiang Third People’s Hospital, Jiujiang, Jiangxi China; 22 Beijing YouAn Hospital, Capital Medical University, Beijing, China; 23 Central Hospital of Hengyang, Hengyang, Hunan China; 24 Shenzhen People’s Hospital, Shenzhen, Guangdong China; 25 The Sixth People’s Hospital of Zhengzhou, Zhengzhou, Henan China; 26 Guangzhou Eighth People’s Hospital, Guangzhou Medical University, Guangzhou, Guangdong China; 27 Loudi Central Hospital, Loudi, Hunan China; 28 Luoyang Central Hospital, Luoyang, Henan China; 29 Xinyang Central Hospital, Xinyang, Henan China; 30 The First Affiliated Hospital of University of South China, Hengyang, Hunan China; 31 The Third People’s Hospital of Hunan Province, Yueyang, Hunan China; 32 The Third People’s Hospital of Kunming, Kunming, Yunnan China; 33 Chongqing University Three Gorges Hospital, Chongqing, China; 34 First People’s Hospital of Chenzhou, Chenzhou, Hunan China; 35 The Second Affiliated Hospital and Yuying Children’s Hospital of Wenzhou Medical University, Wenzhou, Zhejiang China; 36 Wenzhou Central Hospital, Wenzhou, Zhejiang China; 37 Huizhou Central People’s Hospital, Huizhou, Guangdong China; 38 Shunde Hospital of Southern Medical University, Foshan, Guangdong China; 39 Jiangxi Provincial People’s Hospital, Nanchang, Jiangxi China; 40 The First Affiliated Hospital of Nanchang University, Nanchang, Jiangxi China; 41 The Second Affiliated Hospital of Nanchang University, Nanchang, Jiangxi China; 42 Changsha Central Hospital, Changsha, Hunan China; 43 Wenzhou Traditional Chinese Medicine Hospital of Zhejiang Chinese Medical University, Wenzhou, Zhejiang China; 44 The First Hospital of Hunan University of Chinese Medicine, Changsha, Hunan China; 45 Yiyang Central Hospital, Yiyang, Hunan China; 46 Zhengzhou Central Hospital, Zhengzhou, Henan China; 47 The First People’s Hospital of Yunnan Province, Kunming, Yunnan China; 48 The Central Hospital of Wuhan, Wuhan, Hubei China; 49 Hebei Medical University Third Hospital, Shijiazhuang, Hebei China; 50 The First Affiliated Hospital of Wenzhou Medical University, Wenzhou, Zhejiang China; 51 Jiujiang NO.1 People’s Hospital, Jiujiang, Jiangxi China; 52 People’s Hospital of Zhengzhou, Zhengzhou, China; 53 The Central Hospital of Xiangtan, Xiangtan, Hunan China; 54 Sinopharm Dongfeng General Hospital, Shiyan, Hubei China; 55 Xuancheng People’s Hospital, Xuancheng, Anhui China; 56 Puyang Oilfield General Hospital, Puyang, Henan China; 57 Zhoukou Central Hospital, Zhoukou, Henan China; 58 Guizhou Provincial People’s Hospital, Guiyang, Guizhou China; 59 Shijiazhuang Fifth Hospital, Shijiazhuang, Hebei China; 60 Luoyang First People’s Hospital, Luoyang, Henan China; 61 Yichang Central People’s Hospital, Yichang, Hubei China; 62 School of Life Sciences, Tsinghua University, Beijing, China

**Keywords:** Clinical trials, Gastrointestinal cancer

## Abstract

In this multicenter, randomized, double-blind, active-controlled phase 3 trial (NCT06553456), we assessed the equivalence of recombinant human serum albumin (rHSA) expressed in *Pichia pastoris* to plasma-derived human serum albumin (pHSA) for elevating serum albumin (ALB) levels and non-inferiority regarding ascites improvement in patients with cirrhotic ascites. Using a rigorous dual-endpoint design, the primary endpoint was the change from baseline in serum ALB immediately after the final intravenous infusion. The key secondary endpoint was the ascites improvement rate after the final administration. Ultimately, 364 out of 390 patients completed the study. Statistical equivalence was established for the primary endpoint, with least-squares mean ALB changes of 11.16 ± 0.364 g/L (rHSA) and 10.95 ± 0.395 g/L (pHSA) (LSM difference: 0.21 g/L [95% CI: −0.75, 1.16]). For the secondary endpoint, ascites improvement was non-inferior, demonstrating a 58.0% response rate for rHSA versus 48.7% for pHSA (difference: 9.4% [95% CI: −0.56%, 19.10%]). Both endpoints of the trial were successfully achieved. The overall incidence of adverse events was similar in the two groups; crucially, no anti-drug antibodies were detected in the rHSA group. Exploratory analysis revealed that rHSA significantly reduced glycated ALB (−12.24%), whereas pHSA increased it ( + 12.52%) (*P* < 0.001). Corroborated by LC‒MS characterization, silver-staining purity assessment, and four biochemical quality attributes (free thiol, Hcy, AGEs, and carbonyl levels), this divergence highlights the preserved molecular integrity of rHSA. This trial demonstrated that in patients with cirrhotic ascites, rHSA was statistically equivalent to pHSA in elevating serum ALB and non-inferior in ascites improvement, with a favorable safety profile.

## Introduction

Human serum albumin (HSA), the most abundant plasma protein synthesized by the liver, plays multifaceted physiological roles, including the maintenance of oncotic pressure, ligand transport, and systemic anti-inflammatory and antioxidant activities.^1^ Since the 1940s, HSA has been widely used in clinical practice.^2^ However, the global clinical supply of HSA currently faces unprecedented challenges, especially in developing regions.^3^ The World Health Organization (WHO) has repeatedly highlighted the acute shortage of plasma-derived products in low- and middle-income countries, which struggle to meet escalating clinical demands.^4,5^ Global blood donor shortages and the low, yet non-negligible, risk of blood-borne pathogen transmission associated with plasma-derived human serum albumin (pHSA) remain significant hurdles for its secure clinical application.^6,7^ Consequently, regulatory agencies encourage the adoption of recombinant DNA technology to produce non-animal-derived HSA.^8^ Recombinant human serum albumin (rHSA) expressed in yeast systems offers the potential for pathogen-free, large-scale production.^9,10^

The investigational rHSA evaluated in this trial was produced using a *Pichia pastoris* expression platform. This manufacturing paradigm offers distinct molecular characteristics compared with pHSA, notably the absence of detectable non-enzymatic glycation modifications, along with enhanced structural homogeneity. Furthermore, this highly efficient process ensures shortened manufacturing cycles and controllable costs, facilitating scalable production^11,12^ while completely eliminating the risk of viral contamination.

Preclinical studies have demonstrated that this rHSA exhibits physicochemical properties highly consistent with native HSA, while preserving a more intact, native conformation. Crucially, the formulation composition of rHSA is identical to that of clinical-grade pHSA. Prior phase 1b (NCT05249374) and phase 2 (NCT05858853) trials demonstrated favorable safety profiles and promising efficacy in patients with cirrhotic ascites and hypoalbuminemia.

Building on these findings, we conducted a multicenter, randomized, double-blind, active-controlled, phase 3 confirmatory trial (NCT06553456) to evaluate the efficacy and safety of rHSA at 20 g/day for 7 consecutive days compared with pHSA in patients with cirrhotic ascites and hypoalbuminemia.

Notably, this represents the first phase 3 trial for rHSA employing a rigorous dual-endpoint design, enabling the simultaneous evaluation of a pharmacodynamic surrogate marker (serum albumin elevation) and a direct clinical outcome (ascites improvement). Rigorous patient stratification based on baseline albumin levels, ascites severity, and diuretic use was implemented to mitigate confounders, alongside standardized ultrasonographic protocols to ensure endpoint reliability. Finally, a 90-day follow-up period was incorporated to ensure a comprehensive long-term safety assessment, aligning with the stringent regulatory requirements of the Center for Drug Evaluation (CDE) of the National Medical Products Administration (NMPA) of China.

## Results

### Participant disposition

**Fig. 1:**
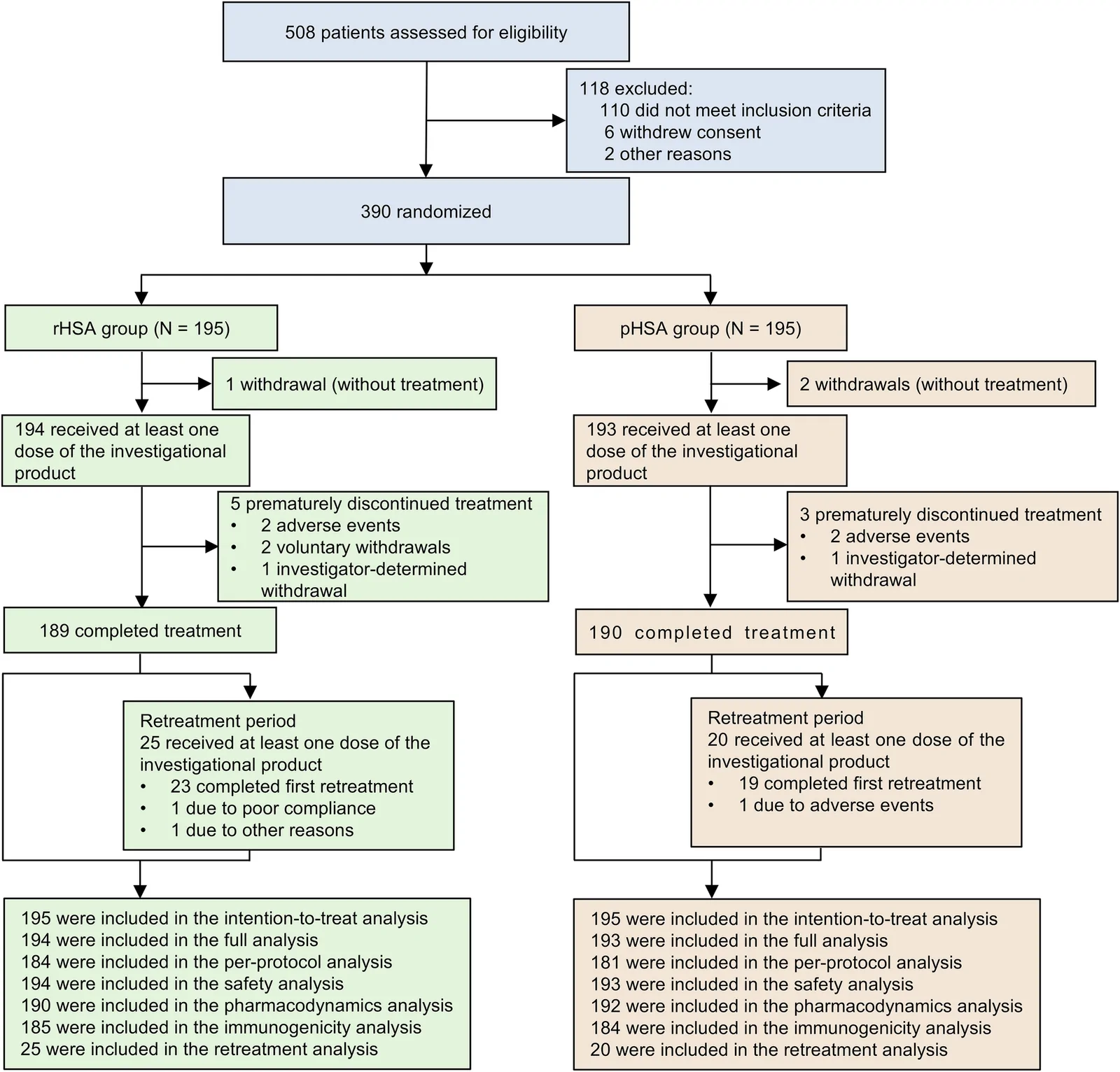
Flowchart of patient disposition. Of the 390 randomized patients, 387 patients received at least one dose of rHSA or pHSA treatment (3 patients did not receive treatment). During the study period, 45 patients received at least one retreatment

From August 2024 through May 2025, a total of 508 patients were screened; of these, 390 were enrolled and randomly assigned to receive rHSA 20 g/day (195 patients) or pHSA 20 g/day (195 patients). Hepatitis B was the major cause of cirrhosis in most of these patients. Overall, 387 (99.2%) randomized patients received at least one treatment. Subsequently, 379 (97.2%) completed the scheduled treatment-period dosing, while 8 patients (2.1%) prematurely withdrew from treatment (5 in the rHSA group, 3 in the pHSA group). Overall, 364 (93.3%) out of 390 randomized patients completed the study. A total of 45 (11.5%) patients received retreatment, of whom 3 (6.7%) patients discontinued therapy due to poor compliance (*n* = 1), adverse events (*n* = 1), or other reasons (*n* = 1). Consequently, 387 (99.2%) patients were included in the full analysis set (FAS) and safety analysis set (SS), 382 (97.9%) in the pharmacodynamics (PD) analysis set, 369 (94.6%) in the immunogenicity analysis set, and 365 (93.6%) in the per-protocol set (PPS). The exclusion of 14 patients who completed scheduled dosing from the PPS was strictly accounted for by major protocol deviations, including unauthorized diuretic dose escalations (*n* = 8), prohibited concomitant medications (*n* = 3), randomization errors (*n* = 2), and missing post-treatment imaging data for the key secondary endpoint (*n* = 1). For the retreatment analysis set, 45 (11.5%) patients who received treatment were included (Fig. 1).

### Baseline characteristics

The characteristics of the participants at baseline were generally similar in the two groups with respect to the cause of cirrhosis, serum ALB level, ascites depth, body weight, abdominal circumference, plasma colloid osmotic pressure, glycated ALB level, and diuretic use. Serum creatinine level, total bilirubin level, Model for End-Stage Liver Disease (MELD) 3.0 score, and Child‒Pugh score were also similar. Overall, the demographic and baseline clinical characteristics of the patients were comparable (Table 1).

**Table 1:** Demographic and clinical characteristics of the patients at baseline^a^

Characteristic	rHSA group (*N* = 194)	pHSA group (*N* = 193)
Mean age, year	54.7 (8.44)	55.5 (8.22)
Sex, no. (%)		
Male	133 (68.6)	148 (76.7)
Female	61 (31.4)	45 (23.3)
Race, no. (%)		
Asian	194 (100%)	193 (100%)
BMI, kg/m^2^	23.60 (2.7)	23.61 (3.1)
Cause of cirrhosis, no. (%)^b^		
Hepatitis B	114 (58.8%)	103 (53.4%)
Alcohol	34 (17.5%)	37 (19.2%)
Hepatitis C	13 (6.7%)	8 (4.1%)
Mixed	10 (5.2%)	15 (7.8%)
Other reason	23 (11.8%)	30 (15.5%)
Maximum ascites depth, mm	48.66 (27.7)	49.32 (29.2)
Ascites depth, no. (%)		
> 0 to < 3 cm	63 (32.5)	62 (32.1)
≥ 3 to < 6 cm	55 (28.4)	53 (27.5)
≥ 6 to ≤ 10 cm	76 (39.2)	78 (40.4)
Serum ALB, g/L^a^	27.292 (3.6)	27.556 (3.7)
< 25 g/L, no. (%)	47 (24.2)	51 (26.4)
≥ 25 g/L, no. (%)	147 (75.8)	142 (73.6)
Serum creatinine level, μmol/L	71.8 (24.7)	77.1 (36.9)
Total bilirubin level, μmol/L	38.8 (19.8)	37.2 (19.8)
MELD 3.0 score^c^	14.2 (3.3)	14.4 (3.5)
Child‒Pugh score^d^	9.2 (1.2)	9.2 (1.2)
Glycated ALB, g/L	5.88	5.83
Diuretic use, no. (%)		
Yes^e^	153 (78.9)	151 (78.2)
Spironolactone	145 (74.7)	145 (75.1)
Furosemide	132 (68.0)	136 (70.5)
Body weight, kg	62.51 (9.4)	62.93 (11.2)
Abdominal circumference, cm	86.14 (8.0)	87.02 (10.3)
Plasma colloid osmotic pressure, mmHg	19.07 (2.8)	19.27 (3.0)

^a^Plus-minus values are mean ± SD

^b^The etiology of cirrhosis was obtained from clinical records; for patients with multiple causes, the primary etiology was adjudicated by the investigators

^c^The Model for End-Stage Liver Disease (MELD) 3.0 score lower limit is 6 points, with higher scores indicating a higher risk of mortality at 3 months. Based on the FAS, after excluding patients with missing baseline creatinine, 192 and 191 patients in the rHSA and pHSA groups, respectively, were available for analysis. The evaluable sample size for the creatinine level was identical

^d^Child‒Pugh scores range from 5 to 15 points, stratifying liver function into three classes (A, B, and C), in which higher scores denote progressively severe hepatic impairment

^e^Baseline diuretic use was defined as the administration of any diuretic within 3 days prior to randomization

### Primary endpoint

**Fig. 2:**
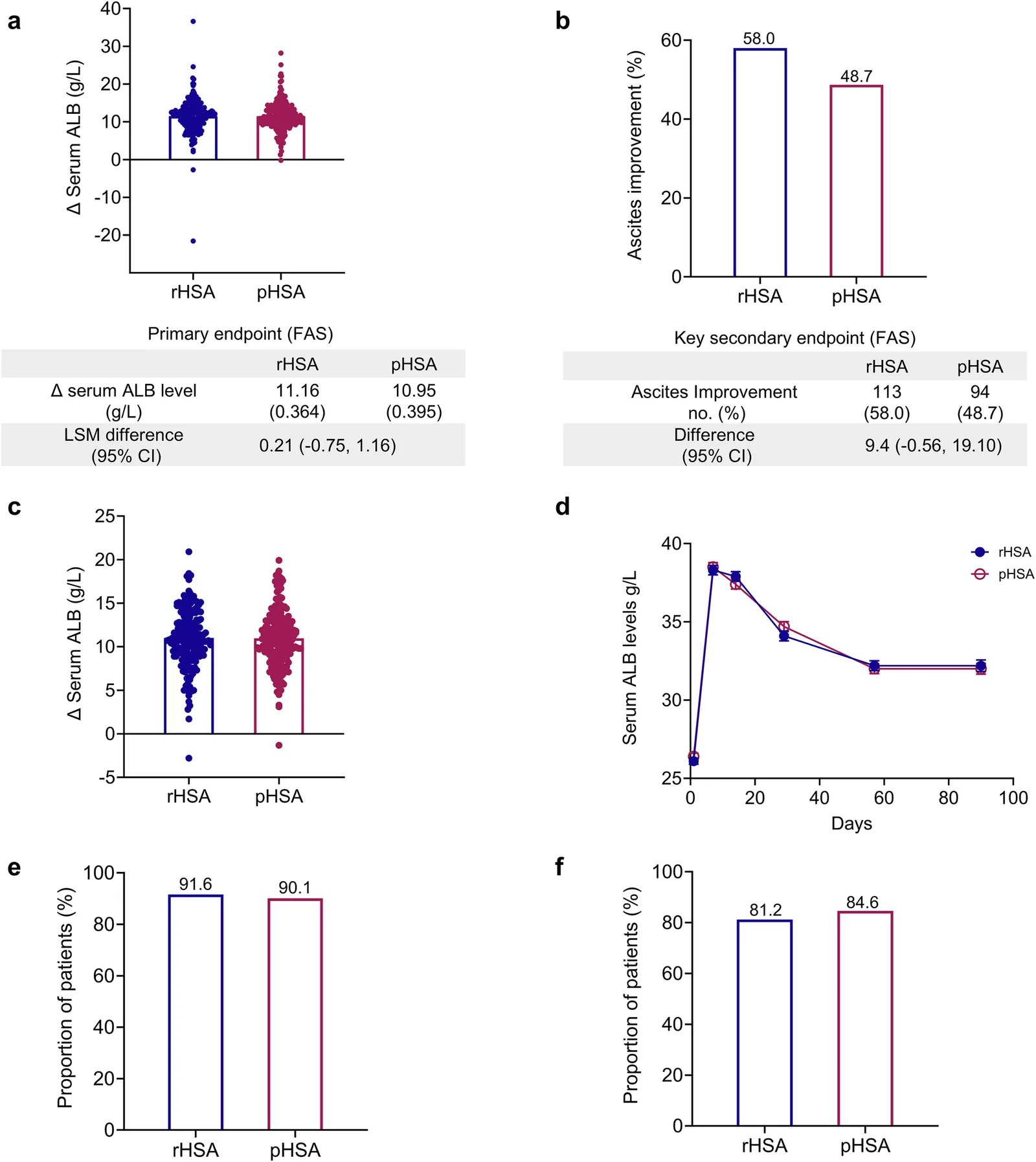
Primary, key secondary, and other secondary endpoints of the trial. **a** Primary endpoint: Change from baseline in serum albumin (ALB) levels (central laboratory) to Day 7 or the end of treatment (EOT). Data shown in the graph are observed values (mean ± standard error (SE); rHSA, *n* = 190; pHSA, *n* = 192). For the primary and key secondary endpoints, multiple imputation was performed to account for intercurrent events. The corresponding results are presented as the least-squares mean ± SE (rHSA, *n* = 194; pHSA, *n* = 193) and are shown beneath the figure. **b** Key secondary endpoint: Ascites improvement rate after the final administration. **c** Secondary endpoint: Change from baseline in serum ALB levels (local laboratory) to Day 7 or EOT. Data are presented as observed values (mean ± SE; rHSA, *n* = 181; pHSA, *n* = 188). **d** Serum ALB levels over time (local laboratory; mean ± SE). **e** Proportion of patients with serum ALB ≥ 35 g/L at EOT (central laboratory; rHSA, *n* = 190; pHSA, *n* = 192). **f** Proportion of patients with serum ALB ≥ 35 g/L at EOT (local laboratory; rHSA, *n* = 181; pHSA, *n* = 188). All analyses were performed in the full analysis set (FAS). Missing serum ALB measurements immediately after the final administration or at EOT were observed in 4 and 1 patients in the rHSA and pHSA groups, respectively, for the central laboratory analyses and in 13 and 5 patients, respectively, for the local laboratory analyses

At the end of treatment (EOT or Day 7), changes in serum ALB levels measured at the central laboratory immediately after the final administration demonstrated equivalence between the rHSA and pHSA groups, with LSM ± SE changes of 11.16 ± 0.364 g/L and 10.95 ± 0.395 g/L (LSM difference: 0.21 g/L [95% CI: −0.75, 1.16]), respectively (Fig. 2a).

All analyses across the ITT, FAS, and per-protocol populations—as well as supplementary, sensitivity, and post hoc ANCOVA/ANOVA evaluations performed without data imputation—yielded highly consistent results, thereby robustly supporting the primary therapeutic conclusions (Supplementary Tables S2–S10). The non‑imputed analyses met the equivalence criteria, with the 95% confidence interval for the treatment difference lying entirely within the prespecified equivalence margins and tightly centered around −0.07 g/L, further confirming the robustness of our conclusions. Subgroup analyses were also generally consistent with the primary analysis, with no meaningful heterogeneity detected across most strata (Supplementary Fig. 1 and Table S11).

### Key secondary endpoint

After treatment, the ascites depth and grade in the rHSA and pHSA groups were substantially reduced from their baselines, with ascites improvement rates of 58.0% (95% CI: 50.84, 64.77) and 48.7% (95% CI: 41.75, 55.71), respectively. The ascites improvement rate in the rHSA group was non-inferior to that in the pHSA group per design (difference: 9.4% [95% CI: −0.56, 19.10]) (Fig. 2b). Analysis of the ITT population similarly demonstrated a statistical non-inferiority conclusion (Supplementary Table S12). Sensitivity, supplementary, and subgroup analyses were also consistent with the primary analysis. For the key secondary endpoint, no notable subgroup effects were observed. Given that diuretic use directly impacts the evaluation of ascites improvement, we performed a post hoc analysis of diuretic administration across all study phases. Baseline utilization rates (78.9% for rHSA vs. 78.2% for pHSA, *P* = 0.805) and drug types were highly balanced between the two groups. During the active 7-day treatment period, overall diuretic utilization drastically decreased to 9.8% in the rHSA group and 13.5% in the pHSA group (*P* = 0.267), which is consistent with the possibility that the observed clinical improvements were not primarily driven by intensified diuretic therapy but rather by albumin supplementation (Supplementary Table S13).

### Secondary endpoints

The changes in serum ALB levels (local laboratory measurement) from baseline to immediately after the final administration demonstrated statistical equivalence between the rHSA and pHSA groups, with LSM ± SE changes of 10.747 ± 0.260 g/L and 10.809 ± 0.258 g/L (LSM difference: −0.062 g/L [95% CI: −0.692, 0.568]) (Fig. 2c), respectively. After treatment, serum ALB levels in both groups substantially increased and remained elevated until the 90-day follow-up (Fig. 2d). Central laboratory measurements confirmed serum ALB levels ≥35 g/L in 91.6% of patients receiving rHSA and 90.1% of those receiving pHSA (difference: 1.3% [95% CI: −4.76, 7.51]) (Fig. 2e). The results from local laboratories were consistent (81.2% vs. 84.6%; difference: −3.6% [95% CI: −11.43, 4.13]) (Fig. 2f). The median time to achieve serum ALB ≥ 35 g/L was similar between groups (5.98 days vs. 5.96 days; relative risk: 0.80 [95% CI: 0.66, 1.03]; *P* = 0.089).

The reduction in ascites depth was also comparable between the groups, with LSM ± SE changes of −17.40 ± 2.088 mm for rHSA and −15.54 ± 2.077 mm for pHSA (LSM difference: −1.86 mm [95% CI: −6.55, 2.84]). Both groups exhibited similar decreases in body weight and abdominal circumference, alongside significant increases in plasma colloid osmotic pressure (PCOP) from baseline (Supplementary Table S14).

**Fig. 3:**
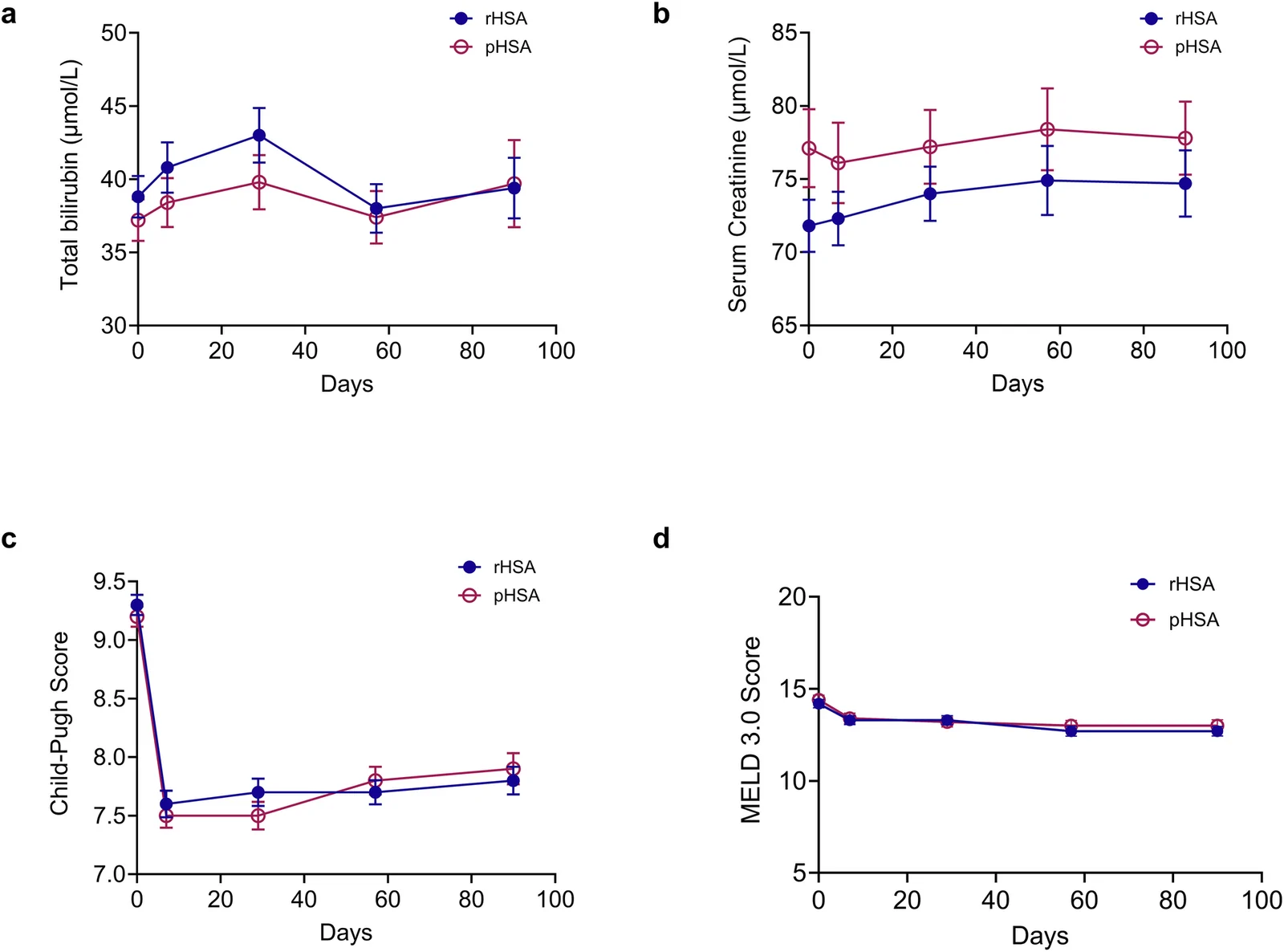
Liver function parameters. **a**–**d** Total bilirubin, serum creatinine, Child‑Pugh score, and MELD 3.0 score from baseline to end of follow‑up. Longitudinal comparison revealed a significant difference between rHSA and pHSA only for serum creatinine (*P* = 0.0113, *two‑way ANOVA*), with no intergroup differences for the other measures (all *P* > 0.05). MELD, Model for End‑stage Liver Disease. Data are mean ± standard error (SE)

Regarding biochemical profiles, total bilirubin showed no significant intergroup differences (overall *P* = 0.2077; all individual visits *P* > 0.05, *two-way ANOVA*). In contrast, serum creatinine exhibited a significant overall difference between the treatment groups (rHSA vs. pHSA; overall *P* = 0.0113) but no significant differences at any individual visit (all *P* > 0.05, *two-way ANOVA*). Notably, despite these modest increases, both MELD 3.0 and Child-Pugh scores decreased by > 1 point, with this improvement maintained through D90 (Fig. 3a–d). During the retreatment period, both groups again demonstrated comparable marked elevations in serum ALB levels (Supplementary Table S15).

### Exploratory endpoint

**Fig. 4:**
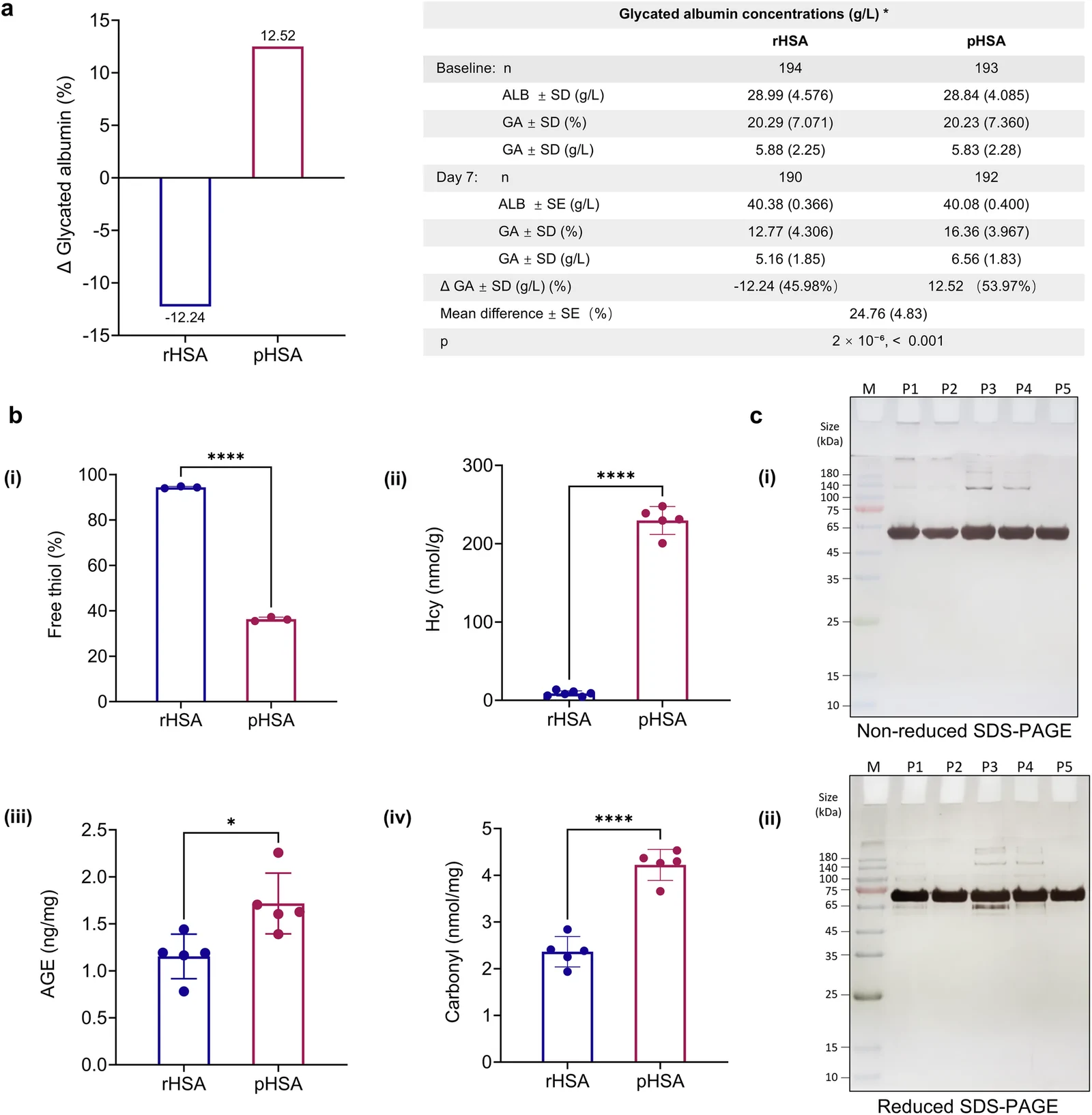
Molecular integrity profiling of rHSA and pHSA. **a** Clinical changes in glycated albumin (GA) after administration. The percentage changes in GA concentrations (ΔGA, %) were calculated at the end of treatment (Day 7). **b** Intrinsic molecular quality attributes: (i) free thiol percentage; (ii) homocysteine (Hcy) concentrations; (iii) advanced glycation end-product (AGE) concentrations; and (iv) carbonyl concentrations. Between-group differences in panels a and b were assessed using Welch’s *t*-test. Data are presented as the mean ± standard deviation (SD). *P < 0.05, ****P < 0.0001. **c** Assessment of the conformational homogeneity and purity of commercially available pHSA and rHSA produced using different expression platforms: (i) Non-reduced SDS-PAGE and (ii) Reduced SDS-PAGE, both using silver staining. Five distinct sources were evaluated at 1 μg of protein per lane: P1 (representative international pHSA), P2 (representative domestic pHSA), P3 (representative non-yeast-expressed rHSA sample), P4 (representative yeast-expressed, excipient-grade rHSA), and P5 (the yeast-expressed rHSA investigated in this study)

Calculated from aggregated data, glycated albumin levels decreased by 12.24% in the rHSA group but increased by 12.52% in the pHSA group on Day 7, yielding a between-group difference of 24.76%. Welch’s *t*-test confirmed a significant treatment effect (*P* ≈ 2 × 10^-6^) (Fig. 4a). These findings suggested that the administered albumin preparations possessed distinct pre-existing modification states before entering circulation. HbA1c did not significantly change after treatment, and the two groups were similar (Supplementary Table S16).

### Safety and immunogenicity

At least one adverse event occurred in 94.3% of the patients receiving rHSA and in 95.9% of those receiving pHSA. Adverse events that were considered by the investigators to be related to the trial regimen were reported in 37.6% of the patients receiving rHSA and in 36.8% of those receiving pHSA (Table 2). The majority (78.6%) of adverse events were resolved during the follow-up period. Infusion-related reactions were rare; the rHSA group reported 3 (1.5%) infusion-related reactions (one episode of fever on Day 5 leading to treatment interruption but resumed on the same day after receiving antipyretics; one episode of infusion site pain on Day 4 requiring no intervention; and one infusion-related reaction on Day 4 leading to discontinuation), while the pHSA group reported 1 (0.5%) hypersensitivity reaction (occurring on Day 1, leading to temporary interruption, resolved with antihistamines and recovered on Day 2) (Supplementary Table S17). All of these patients recovered following standard clinical management. Serious adverse events occurred in 18.6% of the patients in the rHSA group and 20.2% of those in the pHSA group. The most commonly reported serious adverse events were hepatic encephalopathy (3.6% rHSA group vs. 4.7% pHSA group), upper gastrointestinal bleeding (2.6% vs. 3.1%), esophageal variceal bleeding (2.6% vs. 3.1%), and spontaneous bacterial peritonitis (2.1% vs. 2.1%). Two patients reported treatment-related SAEs, which included an infusion-related reaction (rHSA group, CTCAE grade 4) and an esophageal variceal hemorrhage (pHSA group, CTCAE grade 3), and both of them recovered (Supplementary Table S18).

**Table 2:** Safety and adverse events^a^

Event	rHSA (*n* = 194)	pHSA (*n* = 193)
Any adverse event occurring during the treatment period^b^	183 (94.3)	185 (95.9)
Adverse event related to rHSA or pHSA	73 (37.6)	71 (36.8)
Any adverse event leading to the interruption of rHSA or pHSA	2 (1.0)	2 (1.0)
Any adverse event leading to discontinuation of rHSA or pHSA	3 (1.5)	4 (2.1)
Any serious adverse event occurring during treatment period^c^	36 (18.6)	39 (20.2)
Any serious adverse event related to rHSA or pHSA^d^	1 (0.5)	1 (0.5)
Death^e^	5 (2.6)	7 (3.6)
Most frequent adverse events^f^		
Neutrophil count decreased	85 (43.8)	76 (39.4)
White blood cell count decreased	82 (42.3)	75 (38.9)
Platelet count decreased	80 (41.2)	65 (33.7)
Anemia	57 (29.4)	62 (32.1)
Lymphocyte count decreased	50 (25.8)	41 (21.2)
Blood bilirubin increased	47 (24.2)	47 (24.4)
Hypokalemia	34 (17.5)	40 (20.7)
Hypocalcemia	30 (15.5)	29 (15.0)
Fibrinogen decreased	24 (12.4)	25 (13.0)

^a^Data are shown as *n* (%) of all participants. The safety population consisted of all participants who received at least one dose of rHSA or pHSA

^b^Adverse events were coded according to the Medical Dictionary for Regulatory Activities (MedDRA), version 28.0

^c^A serious adverse event is any event that results in death, is life-threatening, causes persistent or substantial disability or incapacity, substantially disrupts normal life functions, or results in or prolongs hospitalization (excluding social or prescheduled admissions, with the reason for hospitalization reported whenever possible). A serious adverse event also includes congenital anomalies, birth defects, or other medically significant events, as per the investigator’s judgment

^d^An infusion-related reaction occurred in the rHSA group (CTCAE Grade 4) and an esophageal variceal hemorrhage in the pHSA group (CTCAE Grade 3); both patients recovered

^e^Five deaths occurred in the rHSA group and seven in the pHSA group, all of which were adjudicated by the investigators as unrelated to the treatment. Two patients in the rHSA group and three in the pHSA group died following trial completion

^f^Events occurring in > 10% of patients in either group

In this trial, 12 deaths were reported: 5 (2.6%) in the rHSA group and 7 (3.6%) in the pHSA group (Supplementary Table S19). In the rHSA group, 2 of the 5 deaths occurred after the end of follow-up (primary disease, Day 125; gastrointestinal bleeding, Day 101). In the pHSA group, 3 of the 7 deaths occurred after the end of follow-up (acute-on-chronic liver failure, Day 100; cerebral hemorrhage, Day 104; hepatic encephalopathy, Day 135). All deaths were assessed by the investigators as being unrelated to treatment. Except for fatal SAEs, the majority of SAEs (96.3%) had been resolved or improved by the end of the trial.

At baseline, 15.98% of patients in the rHSA group and 17.10% in the pHSA group had a history of peritonitis (within 6 months). Following treatment, the incidence of peritonitis-related adverse events decreased to 3.61% and 5.70%, respectively. Prior abdominal paracentesis ( ≥ 1) was reported in 15.46% of the rHSA group and 17.10% of the pHSA group, which decreased to 2.58% and 4.15% post-treatment, respectively. Specifically, only 2 patients (1.03%) in the rHSA group and 1 patient (0.52%) in the pHSA group required therapeutic paracentesis during the 7-day treatment phase. Over the entire study duration, the cumulative incidence remained low, with 5 patients (2.58%) in the rHSA group and 8 patients (4.15%) in the pHSA group undergoing the procedure. At baseline, 9.3% of patients in the rHSA group and 5.7% in the pHSA group had a history of hyponatremia (within 6 months). Following treatment, the incidence of hyponatremia-related adverse events remained comparable between the two arms (8.2% vs. 7.3%, respectively). One acute kidney injury (AKI) adverse event occurred in each group; both were deemed unrelated to treatment and subsequently resolved. Moreover, no other nephrotoxicity was observed. Notably, no cases of pulmonary edema, acute heart failure, or acute circulatory volume overload were observed in this trial.

Immunogenicity analysis confirmed that all evaluable patients treated with rHSA remained negative for both rHSA-specific anti-drug antibodies (ADAs) and host-cell-protein (HCP) ADAs at all assessed time points, reaffirming the product’s high purity and low immunogenic potential.

## Discussion

To our knowledge, this is the first report of a multicenter, randomized, double-blind, active-controlled phase 3 clinical trial evaluating rHSA in patients with cirrhotic ascites. The study used a robust dual-endpoint design, incorporating an equivalence test for the primary endpoint and a non-inferiority test for the key secondary endpoint, to assess efficacy and safety. This trial confirms that the rHSA and pHSA treatments achieved statistical equivalence in elevating serum ALB levels. Furthermore, the rate of ascites improvement in the rHSA group was non-inferior to that in the pHSA group. Strikingly, nearly 60% of patients receiving rHSA achieved a reduction in ascites to grade 0 or 1 by the end of the treatment period. Sensitivity and supplementary analyses further corroborated the primary equivalence conclusion, reinforcing the robustness of the efficacy profile. Executed under a rigorous, GCP-compliant protocol, this study demonstrates the comprehensive clinical benefit of albumin as a therapeutic intervention for cirrhotic ascites, successfully achieving elevations in serum albumin levels and improvements in ascites control among patients receiving permitted diuretic therapy.

However, contextualizing these findings within global albumin data requires a distinct paradigm. Although the ATTIRE trial^13^ showed no survival benefits from targeted albumin escalation in acute decompensated cirrhosis, evaluating a novel recombinant biologic is fundamentally different. Here, changes in serum albumin levels served as a direct surrogate for exogenous drug exposure, as current assays cannot distinguish rHSA from endogenous pHSA in vivo. Crucially, by linking this exposure to clinical efficacy via simultaneous biochemical equivalence (albumin elevation) and clinical non-inferiority (ascites improvement), our dual-validation framework ensures a robust therapeutic evaluation.

Consistent with our phase 2 clinical trial findings, glycated ALB levels were reduced after rHSA treatment, whereas they increased in the pHSA group. Meanwhile, HbA1c and blood glucose levels remained relatively stable. We attribute this discrepancy primarily to inherent differences in the baseline glycation status of the two protein sources. These findings indicate that albumin therapy may need to be evaluated not only by quantitative restoration of circulating albumin concentration but also by the molecular characteristics of the administered albumin population. Human serum albumin is susceptible to various post-translational modifications (PTMs) during physiological circulation and manufacturing processes. Glycation, a prevalent modification, predominantly occurs at specific lysine residues, including K64, K233, K525, and K545.^14^

In this study, LC–MS analysis identified multiple glycation sites in pHSA, whereas glycation was not detected in rHSA under the analytical conditions used (Table 3). This stark disparity directly accounts for the “bidirectional” trend observed in our clinical data: the increase in the pHSA group represents an exogenous influx of pre-modified albumin species accumulated during physiological circulation, while the reduction in the rHSA group reflects the systemic replenishment of a molecularly young (minimally modified), non-glycated alternative. This underscores that the observed changes in glycated albumin status were primarily driven by the molecular integrity of the administered protein rather than endogenous metabolic shifts.

**Table 3:** Site-specific glycation profiling of pHSA and rHSA by LC‒MS

Peptide segment	Modification type	Modification site	Modification ratio (%)	
			pHSA	rHSA
TCVADESAENCD**K**SLHTLFGDK	Glycation	K64	6.92	ND
LVRPEVDVMCTAFHDNEETFL**K**K	Glycation	K136	1.23	ND
AAFTECCQAAD**K**AACLLPK	Glycation	K174	1.01	ND
AEFAEVS**K**LVTDLTK	Glycation	K233	8.71	ND
L**K**ECCE**K**PLLEK	Glycation	K276 K281	1.00	ND
RPCFSALEVDETYVP**K**EFNAETFTFHADICTLSEK	Glycation	K500	1.17	ND
**K**QTALVELVK	Glycation	K525	6.93	ND
EQL**K**AVMDDFAAFVEK	Glycation	K545	9.14	ND

pHSA undergoes glycation, with the primary modification sites identified at Lys64, Lys233, Lys525, and Lys545. ND, not detected

Notably, quality parameter analyses demonstrated that rHSA and pHSA exhibited distinct molecular integrity profiles regarding free thiol, Hcy, AGE, and carbonyl levels (Fig. 4b). Building upon our non-clinical discoveries regarding “young, undamaged” recombinant mouse serum albumin^15,16^ and the “Albumosome”—a novel membraneless organelle formed by pre-folded albumin in the liver^17^—we postulate that structurally intact rHSA may harbor previously unrecognized therapeutic potential. Crucially, our observations suggest that rHSA administration could mitigate cirrhotic complications, including peritonitis and the need for paracentesis. Nevertheless, these promising clinical benefits warrant further validation in prospective trials specifically powered to assess changes in complication rates.

Overall, rHSA demonstrated a favorable safety and tolerability profile in patients with cirrhotic ascites. The overall incidence and spectrum of adverse events were comparable between the two groups. By the end of the study, the vast majority (78.6%) of adverse events had been resolved in both arms. The incidence of infusion-related reactions was 1.5% in the rHSA group vs. 0.5% in the pHSA group (difference: 1.03% [95% CI: −0.98, 3.04]). This difference was not statistically significant according to Fisher’s exact test (*P* = 0.62). Most patients who died during the trial had severe baseline conditions, with deaths primarily attributed to the underlying liver disease itself. In one case of acute coronary syndrome, gastrointestinal bleeding occurred during follow-up, necessitating surgery due to the risk of rebleeding; the patient subsequently developed fatal heart failure postoperatively.

This study confirmed that rHSA exhibits a favorable safety and tolerability profile, characterized by minimal immunogenicity. Throughout our phase 1–3 clinical trials, all rHSA-treated patients remained anti-drug antibody (ADA)-negative. This ADA-negative profile is directly attributable to our proprietary manufacturing and ultra-pure refinement processes, which effectively mitigate potential immunogenicity risks often associated with recombinant expression systems. In view of the fact that rHSA requires unusually high gram-scale clinical dosing (20 g/day)—far exceeding that of typical recombinant proteins—strict regulation of yeast-derived impurities (e.g., host cell proteins) is imperative. Nonclinical toxicology studies further supported this, demonstrating that trace impurity levels remained substantially below the 5 μg/kg safety threshold in rats, thereby providing a > 1500-fold safety margin. To achieve this, we established stringent quality specifications using highly sensitive analytical techniques (mass spectrometry, silver-stained SDS‒PAGE, CE‒SDS, ELISA, etc.) that ultimately exceed current standards for pHSA.

The structural complexity of HSA—characterized by 17 pairs of disulfide bonds, a single free thiol at Cys-34, and 36 helix-breaking residues (24 Prolines and 12 Glycines) interspersed throughout its 30 α-helices—poses a formidable challenge for correct refolding to the precise native conformation of rHSA. Our previous studies on the kinetics and thermodynamics of protein folding intermediates and disulfide bond constraint^18,19^ underscore that even subtle environmental stressors—such as pH fluctuations, temperature, ionic strength, organic solvents, or detergents—can steer the polypeptide toward misfolded states or persistent intermediates. Beyond environmental factors, improper post-translational modifications (PTMs), glycation, oxidation, or suboptimal storage conditions can induce conformational deviations in either pHSA or rHSA. These changes directly compromise biological function and increase immunogenicity risk.

Regarding purity, our rHSA reached an ultrahigh purity of 99.999,999%, with host cell protein (HCP) levels rigorously controlled below 10 ng/g. Critically, we contend that ELISA-based measurements alone are insufficient. We employed a more stringent dual-verification protocol: silver staining—with a detection limit of ≤ 1 ng—was used as a stringent benchmark. We propose that the evaluation of rHSA purity should be conducted at its clinically relevant formulation concentration (e.g., 200 mg/mL). Our data demonstrate that while our rHSA at 200 mg/mL maintains a strictly monomeric profile on silver-stained SDS‒PAGE (1 µg per lane), commercially available albumin products, including pHSA, frequently exhibit dimeric or even polymeric populations under identical conditions (Fig. 4c). Critically, maintaining this monomeric state presents a significant concentration- and time-dependent challenge. While achieving a single band with loading amounts up to 40 µg is possible at the laboratory scale (Supplementary Figs. S2a, b), the risk of intermolecular disulfide exchange inherently increases during extended storage periods (e.g., exceeding two years) due to the high reactivity of Cys-34, especially at high concentrations. Consequently, the sustained maintenance of structural homogeneity at 200 mg/mL serves as a more stringent benchmark for defining high-quality clinical-grade albumin. By relying on raw, original analytical data to confirm these conformational parameters, we demonstrate that rHSA achieves exceptional structural fidelity.

These conformational variations, which can arise across different expression systems (even within the same system) or manufacturing batches, are not merely structural nuances; they can influence the protein’s biological profile and immunogenicity. Given that high intrinsic quality is fundamental to ensuring consistent clinical efficacy and safety, we propose adopting more refined quality standards—specifically by the aforementioned 4 biochemical parameters monitoring the free thiol level while strictly limiting the levels of redox-related impurities and oxidative damage (Hcy, AGEs, and carbonyl groups)—as essential criteria for rHSA to ensure a secure transition toward recombinant alternatives in global clinical practice.

Several limitations of this study should be acknowledged. First, the ascites improvement rate was evaluated using a composite assessment of ascites grade and depth. Recognizing the inherent variability in ultrasonographic depth measurements, we reached a consensus with the Center for Drug Evaluation (CDE) prior to study initiation. Consequently, we established a rigorous Standard Operating Procedure (SOP) for abdominal ultrasonography in ascites measurement and grading to guarantee robust comparability between groups. Second, the sample size calculation was designed to achieve statistical power for both primary and key secondary endpoints simultaneously. For the key secondary endpoint, the assumption was that the experimental group would demonstrate a 5% higher improvement rate than the control group—a hypothesis distinct from the conventional non-inferiority design based on equality between groups. The phase 3 results ultimately validated this assumption (difference: 9.4% [95% CI: −0.56%, 19.10%]). Third, data collection was limited to 90 days, restricting insight into the long-term effects of rHSA. However, this study may provide information regarding the impact of rHSA on cirrhosis-related complications and mortality, as previously reported with pHSA^20^. To address this, it is justified to conduct post-marketing real-world clinical studies with a special focus on long-term clinical outcomes. Fourth, the study population was exclusively Asian and thus lacked racial and ethnic diversity. However, considering that pHSA has been globally used for decades and our rHSA shows a safety and efficacy profile essentially identical to pHSA with no new safety concerns, extending rHSA use to other populations is conceivable, provided that bridging studies or international multicenter trials are conducted to account for potential ethnic differences.

In summary, this phase 3 clinical trial demonstrated that rHSA is equivalent to pHSA in elevating serum ALB levels and non-inferior to pHSA for ascites improvement in patients with cirrhotic ascites and hypoalbuminemia. Furthermore, rHSA demonstrated a favorable safety profile, high tolerability, and minimal immunogenicity. Beyond the clinical outcomes, rHSA offers distinct manufacturing advantages, including a condensed production cycle, high intrinsic purity, cost-effectiveness, and a fully scalable supply capacity. These results strongly support its broader clinical integration and warrant further investigation across a wider spectrum of indications. More broadly, these findings support the incorporation of molecular integrity assessment as a complementary dimension for the development and evaluation of future therapeutic albumin products.

## Methods

### Study design and patients

This was a multicenter, randomized, double-blind, active-controlled phase 3 clinical trial (NCT06553456) conducted at 61 centers in China (Supplementary Table S1). To ensure rigorous protocol compliance and high-fidelity data collection, all participating sites were restricted to certified tertiary Grade A or specialized academic hospitals holding official National Medical Products Administration (NMPA) Good Clinical Practice (GCP) certification and possessing extensive clinical expertise in hepatology. The protocol and informed consent form were approved by the ethics committees of the leading site, Beijing Friendship Hospital, Capital Medical University (approval number: 2022-P1-076-06/07/09), and all participating sites. All patients provided written informed consent before participating in the clinical trial. In cases where a participant or their legal guardian was unable to read, an impartial witness was utilized. The trial was conducted in accordance with the Declaration of Helsinki (1964, updated in 2024), ICH-GCP guidelines, and local regulations. The study design, endpoints, and statistical hypotheses were formally communicated to the CDE of the NMPA.

The inclusion criteria were adults aged 18–70 years with cirrhotic ascites (grades 1–2) and serum ALB level < 30 g/L, with a BMI between 17.0–29.0 kg/m^2^, regardless of sex. Effective contraceptive measures were required for fertile men and women of childbearing age.

The key exclusion criteria included allergy to yeast or yeast-derived products; severe and active underlying diseases of the digestive, cardiovascular, and metabolic systems; alanine aminotransferase (ALT) > 5 × ULN, aspartate aminotransferase (AST) > 5 × ULN, total bilirubin (TBIL) > 4 × ULN; serum creatinine (sCr) > 3 × ULN, proteinuria; absolute neutrophil count (ANC) < 1.0 × 10^9^/L; platelets (PLT) < 20 × 10^9^/L; hemoglobin (HGB) < 70 g/L; prothrombin time (PT) prolonged > 5 s; receipt of human plasma preparations within seven days before the first administration of the study drug; history of organ transplantation; and Grade III or IV hepatic encephalopathy (HE). Patient enrollment followed a rigorous sequential screening process aligned with the CONSORT statement.

### Randomization and intervention

Patients were randomly assigned (1:1) to receive either 20 g/day of rHSA or 20 g/day of pHSA for 7 days. Stratified block randomization (a block size of 4) was used to ensure balanced allocation across treatment arms. Stratification was based on screening serum ALB level ( < 25 g/L vs. ≥ 25 g/L), baseline ascites depth ( > 0 to <3 cm; ≥ 3 to < 6 cm; ≥ 6 to ≤ 10 cm), and baseline diuretic use (yes/no).

The trial employed a double-blind design, and blinding integrity was rigorously maintained. Patients, investigators, and sponsor personnel remained blinded throughout. Unblinded site personnel (e.g., administrators/dispensers) were responsible for drug distribution but were strictly prohibited from disclosing treatment allocation.

Emergency unblinding could be performed by investigators via the IWRS if a participant prematurely discontinued the blinded treatment phase or in the event of a medical emergency. Any unblinding event must be promptly reported to the medical monitor. Unblinding for any other reason was considered a protocol deviation.

### Procedures

All randomized patients were hospitalized and were assigned to receive intravenous infusions of rHSA or pHSA for 7 days or until their serum ALB level reached ≥ 40 g/L (whichever occurred first) and then entered the follow-up period (90 days) as outpatients. Re-treatment between Day 29 and Day 76, if clinically indicated, followed the same drug selection and dosing regimen as the initial treatment period. During the trial, patients were assessed, and blood samples were collected at pre-specified time points. Patients were instructed to follow a standardized “light diet” (low-salt diet) per routine clinical practice for decompensated cirrhosis. Strict quantitative restrictions on daily sodium intake were not enforced to avoid non‑compliance or reporting bias in this large‑scale real‑world setting. This pragmatic instruction was applied uniformly to both the rHSA and pHSA arms, eliminating inter‑group confounding. While this approach may introduce minor unmeasured variability in fluid dynamics, it ensures robust comparability under standard clinical conditions.

For participants who were receiving diuretic therapy at screening, the diuretic regimen was required to remain unchanged from baseline through Day 7. Every effort was made to maintain the diuretic regimen without modification during the follow-up period (Day 8 through Day 90). However, investigators were permitted to adjust the diuretic regimen during follow-up based on clinical judgment. In addition, dose adjustments were allowed at the investigators’ discretion in the event of diuretic-related adverse events, such as electrolyte disturbances. Any increase from the baseline dose of concomitant diuretics during the treatment period was handled as a protocol-specified intercurrent event, using a standardized approach to minimize the potential impact of this variable on efficacy outcomes. The investigational products, rHSA (designated as PG-rHSA, manufactured by Shenzhen Protgen Co., Ltd.) and pHSA (Chengdu Rongsheng Pharmaceuticals Co., Ltd.), were supplied as a 20% solution (10 g/50 mL per vial) and administered intravenously without prior dilution. The standard dose of 20 g (2 × 10 g/50 mL, 20%) was infused over 1 h ( ± 10 min). Following the infusion, a standard intravenous line flush with normal saline was performed for 3 to 6 minutes, which concluded the entire administration procedure.

Ascitic depth was measured by trained operators *via* standardized abdominal ultrasound. The maximum anteroposterior depth of anechoic fluid from a comprehensive four-quadrant and pelvic scan was recorded as the final depth. To minimize intra-observer variation, the same operator ideally performed all longitudinal measurements for a given participant.

To ensure objective evaluation, samples related to the primary endpoint (serum albumin), pharmacodynamics (PD), immunogenicity, and glycated albumin were analyzed by a qualified independent central laboratory. All resulting data were then transferred in a blinded manner to an independent third-party biostatistician for statistical analysis.

Hcy, AGEs, protein carbonyl, and free thiol levels were determined using a commercial Hcy assay kit (Jiangsu Aidisheng Biological Technology Co., Ltd., Cat. #ADS-W-D013), an AGE ELISA kit (Cloud-Clone Corp., Cat. #CEB353Ge), a Protein Carbonyl Content Assay Kit (Abcam, Cat. #ab126287), and Ellman’s method, respectively.

### Endpoints

The primary endpoint was the change in serum ALB levels measured at the central laboratory from baseline to immediately after the final intravenous administration. The key secondary endpoint was the rate of ascites improvement after the final administration (evaluated centrally between baseline and the same day immediately following the last administration of albumin). Ascites improvement was defined using grade-specific criteria: for patients with Grade 1 ascites, complete resolution of ascites; for those with Grade 2, a reduction in ascites by ≥ 1 grade or a decrease ≥ 25% and > 1 cm in the maximum depth of ascitic fluid. Other secondary endpoints included the change in serum ALB levels determined by the local laboratory from baseline to immediately after the final administration, the proportion of patients with ALB ≥ 35 g/L at the completion of the final administration, the time to reach ALB ≥ 35 g/L, changes in ascites depth, body weight, abdominal circumference, and changes in plasma colloid osmotic pressure (PCOP) from baseline. Exploratory efficacy endpoints included the changes in glycated albumin and glycated hemoglobin (HbA1c) levels from baseline to immediately following the final administration.

### Safety

Adverse events were coded according to the Medical Dictionary for Regulatory Activities (MedDRA). The incidence of adverse events during treatment was summarized by severity and causal relationship with the study drug, based on the MedDRA System Organ Class (SOC) and Preferred Term (PT). Grading was performed according to the National Cancer Institute Common Terminology Criteria for Adverse Events (NCI CTCAE) v5.0. Blood samples for immunogenicity evaluation were collected on Days 1 and 90 or at the last visit for those who prematurely withdrew.

### Statistical analysis

Except for the analysis of exploratory endpoints, all statistical analyses were performed using SAS v9.4.

The primary endpoint was analyzed using an equivalence hypothesis test with a margin of ± 2 g/L. This margin was strictly evaluated and formally communicated to the Center for Drug Evaluation (CDE) of the National Medical Products Administration (NMPA) during our protocol design phase, with reference to the confirmatory trial of Medway.^21^ From a clinical perspective, a 2 g/L variance falls entirely within the boundaries of normal physiological fluctuation and central laboratory assay tolerance, thereby exerting a negligible impact on real-world clinical decision-making and long-term prognostic evaluations.

Two one-sided tests at *α* = 0.025 provided 90% power with 328 patients (164 receiving rHSA, 164 receiving pHSA). The key secondary endpoint was analyzed by a non-inferiority hypothesis with a −10% margin at a one-sided *α* = 0.025. Based on the Phase 2 trial results, the between-group difference in ascites improvement rate (rHSA group minus pHSA group) was 10.9%. After adjusting for stratification factors used in the Phase 3 trial as covariates, the adjusted difference was 5.8%. Based on these findings, assuming a 50% improvement rate in the pHSA group and a 5% difference between the rHSA and pHSA groups, a sample size of 348 patients was required to achieve 80% power. Assuming a 10% dropout rate, a total of 388 patients were planned to be enrolled (1:1 allocation). Other efficacy endpoints were analyzed exploratorily. Unless otherwise specified, all statistical tests used a two-sided confidence level of α = 0.05 and calculated two-sided 95% confidence intervals (CI). Given the exploratory nature of the secondary and exploratory endpoints, no formal multiplicity adjustments were applied; hence, associated *P*-values are nominal, and any confirmatory interpretation based on them would be speculative.

For the primary endpoint (FAS), an analysis of covariance (ANCOVA) model evaluated the central laboratory serum ALB change from baseline to the end of treatment (EOT). The treatment group served as the fixed effect, with baseline ALB, ascites depth strata, and diuretic use as covariates. The results are presented as least squares means (LSM) ± standard errors (SE) and LSM differences with 95% CIs.

Intercurrent events (premature discontinuation due to lack of efficacy, adverse events, or other reasons; increased diuretic therapy, use of additional albumin, blood products, prohibited concomitant therapies during treatment, or death) were addressed through multiple imputation under pre-specified event-specific strategies. Sensitivity analyses were conducted using both an analysis of variance (ANOVA) model and an ANCOVA model with baseline serum ALB as the only covariate. Supplementary analyses were based on the FAS or PPS, and subgroup analyses were based on the FAS, and the primary analysis was repeated to assess the robustness of the primary analysis results. Furthermore, post hoc primary (ANCOVA) and sensitivity (ANOVA and ANCOVA) analyses were performed without data imputation using pre-specified event-specific strategies.

For the key secondary endpoint, based on the FAS, the ascites improvement rate at the final infusion was computed by treatment group with 95% CI (Clopper-Pearson); the between-group rate difference (rHSA − pHSA) and its 95% CI were estimated using the stratified Newcombe method. Intercurrent events were addressed through multiple imputation under pre-specified event-specific strategies. Supplementary and subgroup analyses were based on the Per-Protocol Set (PPS), and the primary analysis was repeated. Sensitivity analyses were conducted using the Newcombe method to estimate the between-group rate difference (rHSA − pHSA) and its 95% CI.

Post-hoc supplementary analyses were performed for the primary and key secondary endpoints using the ITT population (defined as all randomized patients) based on the primary analysis methodology. These supplementary analyses were conducted to evaluate the robustness of the findings, as the ITT analysis set was not pre-specified in the statistical analysis plan.

For other secondary endpoints, ANCOVA models similarly assessed local-laboratory ALB changes, ascites depth, body weight, and abdominal circumference, utilizing corresponding baselines as covariates. Intercurrent events (premature discontinuation due to lack of efficacy, adverse events, or other reasons; increased diuretic therapy; use of additional albumin, blood products, prohibited concomitant therapies, or death) were addressed through multiple imputation under pre-specified event-specific strategies.

Based on the FAS, the proportion of patients achieving a serum ALB level ≥ 35 g/L at the completion of the final administration and the corresponding 95% two-sided CI (based on the Clopper-Pearson method) were calculated by treatment group. Time to reach serum ALB level ≥ 35 g/L in each treatment group was obtained using the Kaplan‒Meier method, and the two-sided 95% CI of the corresponding estimates was constructed using the log-log transformation of the Brookmeyer-Crowley method. Patients who did not achieve ALB ≥ 35 g/L by the end of the study were censored at their last available assessment.

Based on the pharmacodynamics (PD) analysis set, PCOP was summarized by treatment group and visit.

Analysis of the exploratory endpoint, the glycated albumin level, was derived from the serum ALB level and the percentage of glycated albumin. The standard deviation was calculated assuming independence between the two variables, yielding a conservative estimate. The percentage change between groups was compared using Welch’s *t*-test. The analysis of HbA1c was conducted by treatment group and visit within the full analysis set (FAS). Safety profiles were analyzed descriptively.

## Supplementary information

Supplementary

Protocol

Statistical Analysis Plan (SAP)

## Source data

Source Data

## Data Availability

The study protocol and the datasets supporting the findings of this study are included in the Article, Supplementary Information, and Source Data files. Due to patient privacy considerations and regulatory restrictions, the raw individual participant data are not publicly available. Deidentified individual participant data may be accessed upon reasonable request to the corresponding author. Requesters must submit a research proposal detailing the study objectives and statistical analysis plan for approval, and all procedures must comply with applicable ethical, institutional, and regulatory requirements. Source data are provided with this paper.
