# Supplementary material for: A randomized, double-blind, active-controlled phase 3 multicenter trial of rHSA for cirrhotic ascites
Source: Signal Transduct Target Ther. 2026 Sep 10;11:390. doi: 10.1038/s41392-026-02951-7 (PMC13588796; doi:10.1038/s41392-026-02951-7)
Supplement: Supplementary file 1 [file 41392_2026_2951_MOESM2_ESM.pdf]

A Randomized, Double-blind, Parallel-group Phase II/III Clinical Trial to Evaluate the Efficacy and Safety of Recombinant Human Serum Albumin Compared to Plasma-derived Human Serum Albumin in Patients with Cirrhotic Ascites

Clinical Trial Protocol

Version No.: 3.0 Version date: July 18, 2024

**Clinical Trial Notification No.:**	2020LP00899
**Protocol No.:**	rHSA 2020-3
**Investigational Drug:**	Recombinant Human Serum Albumin Injection
**Trial Phase:**	Phase II/III
**Leading Site:**	Beijing Friendship Hospital, Capital Medical University
**Principal Investigator:**	Professor Jidong Jia

**Sponsor:**	Shenzhen Protgen Co., Ltd.
**Statement of Compliance:**	This study will be conducted in strict accordance with the Good Clinical Practice (GCP) requirements.

Confidentiality Statement

The information contained in this study protocol is the property of Shenzhen Protgen Co., Ltd. Therefore, it is only provided for review by the sponsor, investigators, co-investigators, Ethics Committees (ECs), regulatory authorities, and other relevant medical institutions. Unauthorized individuals are asked to delete, abandon, or mask this document when receiving it. Disclosure of any information to third parties not related to this study is strictly forbidden without the written approval of the sponsor, except for necessary explanations to potential patients of this study when signing an informed consent form (ICF).

PROTOCOL SIGNATURE PAGE

We have read the clinical trial protocol for the "A randomized, double-blind, parallel-group Phase II/III clinical trial to evaluate the efficacy and safety of Recombinant Human Serum Albumin compared to Plasma-derived Human Serum Albumin in patients with cirrhotic ascites." (Protocol No. rHSA 2020-3, Version No. 3.0, Version date: July 18, 2024), and we have confirmed this clinical trial protocol and agreed to conduct the clinical trial according to this protocol.

We will keep this clinical trial protocol and relevant contents confidential.

Sponsor: Shenzhen Protgen Co., Ltd.

Address: Room 1088, 10th Floor, Investment Building, Longcheng Street, Longgang District, Shenzhen, Guangdong Province, China

Project Director of Sponsor (Print): **(Signature):**

Date: (Year/Month/Day)

STATEMENT OF INVESTIGATOR

1) I agree to personally participate in or direct this clinical study.
2) I have received the Investigator's Brochure (IB) and have been informed about the preclinical study of the investigational drug.
3) We have read the clinical trial protocol for the "A randomized, double-blind, parallel-group Phase II/III clinical trial to evaluate the efficacy and safety of Recombinant Human Serum Albumin compared to Plasma-derived Human Serum Albumin in patients with cirrhotic ascites." (Protocol No. rHSA 2020-3, Version No. 3.0, Version date: July 18, 2024). The study will be conducted in accordance with the ethical and scientific principles set forth in the Declaration of Helsinki and GCP. I agree to conduct this clinical study in accordance with the design and provisions of this protocol and to inform the Sponsor prior to making any modifications. The modification can only be implemented after being approved by the Ethics Committee unless action is necessary to protect the safety, rights, and interests of the participants.
4) I will, in accordance with GCP requirements, ensure that all patients sign the written informed consent form (ICF) prior to being enrolled in the study.
5) I will be responsible for making clinical decisions to ensure that participants receive timely and appropriate treatment for adverse events (AEs) during the study and to record and report serious adverse events (SAEs) in accordance with relevant national regulations.
6) I guarantee authentic, accurate, complete, and timely data entry into the study's medical records. I will accept monitoring and auditing by the monitor or auditor appointed by the Sponsor and auditing and inspection by the drug regulatory authority to ensure the quality of the clinical trial.
7) I promise to keep the patients' information and related matters in confidence. I have been informed that breaking my promise will result in legal liability.
8) I agree to disclose my full name and occupation to the sponsor and to disclose expenses related to the clinical study upon request. I agree that commercial and economic activities related to this study are prohibited.

Clinical study site:

Address:

Principal Investigator (Print): **(Signature):**

Date: (Year/Month/Day)

PROTOCOL SIGNATURE PAGE

Title:	A Randomized, Double-blind, Parallel-group Phase II/III Clinical Trial to Evaluate the Efficacy and Safety of Recombinant Human Serum Albumin Compared to Plasma-derived Human Serum Albumin in Patients with Cirrhotic Ascites
Protocol No.:	rHSA 2020-3
Investigational Drug:	Recombinant Human Serum Albumin Injection
Version No.:	3.0
Version Date:	July 18, 2024

Prepared/revised by:

Name: Date: (Year/Month/Day)

Title: Company:

Table of Content

TABLE OF CONTENT 1
ABBREVIATIONS 6
SYNOPSIS 8
TRIAL FLOWCHART (PHASE II 10 G/DAY DOSE GROUPS [GROUP 1 AND GROUP 3]) 37
STUDY FLOW DIAGRAM (PHASE II, 20 G/DAY GROUP [GROUP 2 AND GROUP 4]) 42
TRIAL FLOWCHART (PHASE III, GROUP A AND GROUP B) 46
TRIAL FLOWCHART (PHASE III - RETREATMENT PERIOD DURING FOLLOW-UP) 51
CLINICAL TRIAL PROTOCOL — MAIN TEXT 54
1 STUDY BACKGROUND 54
1.1 Introduction to Hepatic Cirrhotic Ascites 54
1.2 Introduction to Plasma-derived Human Serum Albumin (pHSA) 55
1.2.1 Application of pHSA in the treatment of hepatic cirrhotic ascites 56
1.2.2 Market overview of plasma-derived human serum albumin (pHSA) 57
1.3 Research and Development Progress of Injection-grade Recombinant Human Serum Albumin. 58
1.4 About the Investigational Drug 59
1.4.1 Introduction to the investigational drug recombinant human serum albumin (rHSA) 59
1.4.2 Phase Ia (single-ascending-dose) clinical trial in healthy volunteers 59
1.4.3 Phase Ib (multiple-ascending-dose) clinical trial in patients with cirrhotic ascites 60
1.5 Risk-Benefit Assessment 62
1.5.1 Potential risks 62
1.5.2 Potential benefits 64
1.5.3 Potential risks and benefit assessment 65
2 STUDY OBJECTIVES 66
2.1 Phase II 66
2.2 Phase III 66
3 STUDY DESIGN 67
3.1 Overall Design 67
3.2 Selection of Study Population and Rationale 69
3.3 Selection and Rationale for the Dosing Regimen 71
3.4 Selection and Rationale for Primary and Key Secondary Endpoints in Phase III 74
3.5 Provisions for Re-treatment during the Follow-up Period of the Phase III Stage 75
3.6 PD Study 76
3.7 Immunogenicity Study 77
3.8 Interim Analysis 78
3.9 Establishment of IDMC 78
3.10 Trial Suspension or Termination 78
4 POPULATION SELECTION 79
4.1 Inclusion Criteria 79
4.2 Exclusion Criteria 80
4.3 Criteria for Treatment Termination 81

4.4 Early Withdrawal Criteria ... 82
4.5 Screening Failure and Rescreening Regulations ... 83
4.6 Randomization ... 83
4.7 Participant assignment and numbering ... 84
4.8 Blinding and Unblinding Regulations ... 84
4.8.1 Blinding execution ... 84
4.8.2 Drug coding ... 85
4.8.3 Routine unblinding ... 85
4.8.4 Emergency unblinding ... 85
5 INVESTIGATIONAL DRUGS ... 86
5.1 Basic Information of Investigational Product ... 86
5.2 Packaging and Labeling ... 87
5.3 Investigational Product Management ... 87
5.4 Instructions for Use of Investigational Product by Participants ... 88
5.4.1 Usage and dosage of investigational product ... 88
5.4.2 Dietary management for participants during investigational product administration ... 89
5.4.3 Medication errors and management ... 90
5.5 Concomitant Medications and Treatments ... 90
6 TRIAL PROCEDURES ... 92
6.1 Screening Period (D-28 to D-1) ... 92
6.2 Treatment Period ... 95
6.2.1 10 g/day dose group (D1–D14) ... 97
6.2.2 20 g/day dose group (Phase II and Phase III (D1–D7)) ... 100
6.3 Follow-up Period ... 102
6.3.1 10 g/day Dose Group (Phase II) (D15–D57) ... 102
6.3.2 20 g/day Dose Group (Phase II) (D8–D57) ... 103
6.3.3 Groups A and B (Phase III) (D8–D90) ... 105
6.4 Retreatment Period during Follow-up (Applicable Only to Phase III) ... 107
6.4.1 RD1 visit ... 109
6.4.2 RD3 visit ... 109
6.4.3 RD7 Visit ... 109
6.4.4 Re-treatment EOT visit ... 110
6.5 Unscheduled Visits ... 111
6.6 Early Discontinuation Visit ... 111
6.7 PD Study ... 112
6.8 Immunogenicity Study ... 113
7 STUDY ASSESSMENTS ... 113
7.1 Efficacy Assessments113
7.1.1 Waist circumference and weight measurement ... 113
7.1.2 Abdominal ultrasound assessment ... 114
7.1.3 Albumin test ... 114
7.1.4 Efficacy endpoints ... 115
7.2 Safety Evaluation ... 118
7.2.1 Vital signs ... 118

7.2.2 Physical examinations 119
7.2.3 12-lead ECG 120
7.2.4 Thyroid function test 120
7.2.5 Thyroid ultrasound 121
7.2.6 Iodine uptake examination 121
7.2.7 Fundus examination 121
7.2.8 Routine laboratory analysis 122
7.2.9 Infectious disease screening 123
7.2.10 Pregnancy testing 123
7.2.11 ECG 123
7.3 PD Evaluation 124
7.4 Immunogenicity Evaluation 124
7.5 Other Evaluations 124
7.5.1 Demographic 124
7.5.2 Medical history 124
7.5.3 Treatment history 125
7.5.4 Exploratory evaluation 125
8 ADVERSE EVENT 126
8.1 Definition of Adverse Event 126
8.2 Recording of AEs 127
8.3 Follow-up of AEs 128
8.4 Abnormal Laboratory Test Results 130
8.5 Severity of AEs 130
8.6 Criteria for Causality Assessment of AEs 131
8.7 Definition of SAE 132
8.8 Reporting of SAEs and Suspected Unexpected Serious Adverse Reactions 134
8.9 Reporting of Pregnancy Events 135
9 RISK CONTROL AND MANAGEMENT 136
10 STATISTICAL ANALYSES 137
10.1 Estimand 138
10.1.1 Primary estimand 138
10.1.2 Key secondary estimand 139
10.1.3 Other efficacy endpoints 140
10.2 Statistical Assumptions and Sample Size Estimation 140
10.3 Interim Analysis 142
10.4 Statistical Analysis Population 142
10.4.1 Full analysis dataset (FAS) 142
10.4.2 Per protocol set (PPS) 143
10.4.3 Safety set (SS) 143
10.4.4 PD analysis set 143
10.4.5 Immunogenicity analysis set 143
10.4.6 Retreatment analysis set 143
10.5 Statistical Analysis Method 144
10.5.1 Demographic data and other baseline characteristics 144

10.5.2 Efficacy analysis 144
10.5.3 Safety analysis 147
10.5.4 Pharmacodynamic analysis 148
10.5.5 Immunogenicity analysis 148
10.5.6 Exploratory endpoint analysis 148
11 DATA PROCESSING 149
11.1 Source Data and Source Document 149
11.2 Data Management 150
11.3 Storage of Documents 151
12 CLINICAL MONITORING 152
13 QUALITY CONTROL AND ASSURANCE 155
14 PROTOCOL COMPLIANCE AND DEVIATION 155
15 REPORTING AND PUBLISHING 157
15.1 Clinical Trial Report 157
15.2 Confidentiality and Ownership of Trial Data 157
15.3 Publication and Public Disclosure 157
15.3.1 Publication policy 157
15.3.2 Public disclosure policy 158
16 THICAL CONSIDERATIONS AND INFORMED CONSENT 158
16.1 EC Review and Approval 158
16.2 Informed Consent of Participants 159
17 LIABILITY AND INSURANCE 161
17.1 Participant Benefits and Privacy Protection 161
17.2 Liability and Insurance 161
17.3 Documentation 162
17.3.1 Investigator documentation 162
17.3.2 Essential documents of clinical trial 162
17.4 Responsibilities of Each Party and Other Relevant Provisions 162
17.5 Study Institutions and Participants 167
17.5.1 Sponsor 167
17.5.2 Research unit 167
17.5.3 Data management and statistical analysis unit 167
17.5.4 Clinical audit units 167
17.5.5 Biological sample testing unit 167
18 REFERENCES 168
19 APPENDIX 168
19.1 Appendix I Instructions for Waist Circumference Measurement 170
19.2 Appendix II Standard Procedures for Anthropometric Measurement 170

List of In-text Tables and Figures

Table 1. Intercurrent Events and Their Handling Strategies for the Primary Estimand 138
Table 2. Intercurrent Events and Handling Strategies for Key Secondary Estimand 139
Table 3 Sensitivity, Supplementary, and Subgroup Analysis for the Primary Estimand in the Phase III Stage 145
Table 4 Sensitivity, Supplementary, and Subgroup Analysis for the Key Secondary Estimand in the Phase III Stage.. 146

ABBREVIATIONS

Abbreviation	Full term	Abbreviation	Full term
AASLD	American Association for the Study of Liver Diseases	LDH	Lactate dehydrogenase
ADA	Anti-drug antibody	LSM	Least-squares method
AE	Adverse event	LVP	large volume paracentesis
ALB	Albumin	MAFLD	Metabolic associated fatty liver disease
ALD	Alcoholic liver disease	MedDRA	Medical Dictionary for Regulatory Affairs
ALT	Alanine aminotransferase	NCI CTCAE	National Cancer Institute Common Terminology Criteria for Adverse Events
ANC	Absolute neutrophil count		
ANCOVA	Analysis of covariance	PD	Pharmacodynamics
APTT	Activated partial thromboplastin time	pHSA	Plasma-derived Human Serum Albumin
AST	Aspartate aminotransferase	PK	Pharmacokinetics
AUC	Area under the level-time curve	PKCS	Pharmacokinetic concentration analysis set
BMI	Body mass index	PKPS	Pharmacokinetic parameter analysis set
COP	Plasma colloid osmotic pressure	PLT	Platelets
CDE	Center for Drug Evaluation, National Medical Products Administration	PPS	Per protocol set
CRO	Contract Research Organization	PT	Preferred Term
EC	Ethics Committee	RAAS	Renin-angiotensin-aldosterone system
ECG	Electrocardiogram	RCT	Randomised controlled trial
eCRF	Electronic case report form	rHSA	Recombinant human serum albumin
EDC	Electronic Data Acquisition and Management System	SAE	Serious adverse event
FAS	Full analysis set	SAP	Statistical analysis plan
GCP	Good Clinical Practice	SBP	Spontaneous bacterial peritonitis
HBV	Hepatitis B virus	SD	Standard deviation

Abbreviation	Full term
HCC	Hepatocellular carcinoma
HCP	Host cell protein
HCV	Hepatitis C Virus
HE	Hepatic encephalopathy
ICMJE	The International Committee of Medical Journal Editors

Abbreviation	Full term
SOC	System Organ Class
SS	Safety set
SUSAR	Suspected unexpected serious adverse reaction
T-Bil	Serum bilirubin
TT3	Serum total triiodothyronine

Synopsis

**General Information of the Clinical Trial**	Registration classification: Class 1 therapeutic biologics<br>Clinical trial protocol No.: rHSA 2020-3<br>Protocol version No./version date: 3.0 / July 18, 2024<br>Study phase: II/III<br>Investigational drug: Recombinant Human Serum Albumin Injection<br>Sponsor: Shenzhen Protgen Co., Ltd.
**Study Title**	A Randomized, Double-blind, Parallel-group Phase II/III Clinical Trial to Evaluate the Efficacy and Safety of Recombinant Human Serum Albumin Compared to Plasma-derived Human Serum Albumin in Patients with Cirrhotic Ascites
**Study Objectives**	**Phase II**<br>➢ **Primary objectives:**<br>To evaluate the efficacy of Recombinant Human Serum Albumin in increasing serum albumin levels in patients with cirrhotic ascites, and the relationship between dose and response.<br>➢ **Secondary objectives:**<br>1) To evaluate the safety of Recombinant Human Serum Albumin in patients with cirrhotic ascites.<br>2) To evaluate the pharmacodynamics of Recombinant Human Serum Albumin in patients with cirrhotic ascites.<br>3) To evaluate the immunogenicity of Recombinant Human Serum Albumin in patients with cirrhotic ascites.<br>➢ **Exploratory objective**: To evaluate the effect of Recombinant Human Serum Albumin on glycated albumin in patients with cirrhotic ascites.<br>**Phase III**<br>➢ **Primary objectives:**<br>To evaluate the efficacy of Recombinant Human Serum Albumin in patients with cirrhotic ascites, as indicated by the change from baseline in serum albumin level immediately after the final intravenous administration.<br>➢ **Secondary objectives:**<br>1) The efficacy of Recombinant Human Serum Albumin in patients with cirrhotic ascites based on additional efficacy endpoints.<br>2) To evaluate the safety of Recombinant Human Serum Albumin in patients with cirrhotic ascites.<br>3) To evaluate the pharmacodynamics of Recombinant Human Serum

	Albumin in patients with cirrhotic ascites.<br>4) To evaluate the immunogenicity of Recombinant Human Serum Albumin in patients with cirrhotic ascites.<br>➢ **Exploratory objective**: To evaluate the effect of Recombinant Human Serum Albumin on glycated albumin and glycated hemoglobin in patients with cirrhotic ascites.
**Study Design**	**Overall Design:**<br>This study is a randomized, double-blind, active-controlled, parallel-group Phase II/III clinical trial that tests Recombinant Human Serum Albumin (rHSA) in patients with cirrhotic ascites. This study consists of two parts: Phase II and Phase III. In the Phase II part, Plasma-derived Human Serum Albumin will be used as a comparator to evaluate the efficacy of rHSA in increasing serum albumin levels in patients with cirrhotic ascites at different doses and treatment courses, and to establish the dose–response relationship, thereby providing a basis for the dosing regimen and sample size calculation for the Phase III study. In the Phase III part, the change from baseline in serum albumin level immediately after completion of the final intravenous administration will be used as the primary endpoint to evaluate the efficacy of rHSA, and its safety, pharmacodynamic (PD) profile, and immunogenicity will be further evaluated.<br>In the Phase II part, 92 participants are planned to be enrolled. Using baseline serum albumin < 25 g/L vs. ≥ 25 g/L as the stratification factor, participants will be randomly assigned to the following four groups (23 participants per group) in a 1:1:1:1 ratio:<br>➢ Group 1: Investigational drug 10 g/day, administered continuously for 14 days;<br>➢ Group 2: Investigational drug 20 g/day, administered continuously for 7 days;<br>➢ Group 3: Comparator drug 10 g/day, administered continuously for 14 days;<br>➢ Group 4: Comparator drug 20 g/day, administered continuously for 7 days.<br>In the Phase II part, the statistical analysis is planned to be done after all participants in Phase II have completed their final visits, and the statistical hypotheses and sample size in Phase III will be determined based on the results of Phase II.<br>In Phase III, the change in serum albumin level from baseline immediately after the final intravenous administration (based on central laboratory tests) will be evaluated as the primary efficacy endpoint to verify the equivalence hypothesis between the investigational drug and the comparator drug. In Phase III, the clinical benefit of albumin supplementation in participants will also be assessed based on ascites

	improvement. At the individual level, ascites improvement is defined as: resolution of ascites in participants with Grade 1 ascites at baseline; or downgrading of ascites, or a ≥ 25% reduction in ascitic fluid depth from baseline, in participants with Grade 2 ascites at baseline. The ascites improvement rate at the time of completion of the final intravenous administration (i.e., the proportion of participants in each group meeting the criteria for ascites improvement) will be used as a key secondary efficacy endpoint for non-inferiority testing, to further evaluate the efficacy of the investigational drug (rHSA) in patients with cirrhotic ascites. The results from Phase II indicated that the change from baseline in serum albumin level and ascitic fluid depth at the time of completion of the final intravenous administration may be influenced by serum albumin level at baseline, grade of ascites, and diuretic use. Therefore, in Phase III, the baseline serum albumin level (< 25 or ≥ 25 g/L), baseline ascitic fluid depth (< 3 cm; 3-6 cm [inclusive of 3, exclusive of 6]; 6-10 cm [inclusive of 6 and 10]), and baseline diuretic use (yes or no) will be used as stratification factors. In Phase III, 388 participants are planned to be enrolled and randomly assigned in a 1:1 ratio to the following two groups (194 participants per group).<br>➢ Group A: Investigational drug 20 g/day, administered continuously for 7 days.<br>➢ Group B: Comparator drug 20 g/day, administered continuously for 7 days.<br>The Phase II study includes a screening period, a treatment period, and a follow-up period. Each participant will enter the follow-up period after completing 7 or 14 consecutive days of dosing according to their assigned dosing regimen. All participants will undergo the final visit on D57±5 during the follow-up period.<br>The Phase III study includes a screening period, a treatment period, and a follow-up period. Participants will enter the follow-up period after completing 7 consecutive days of dosing and undergo the final visit on D90±7 during the follow-up period. If a participant's serum albumin level drops to < 30 g/L during the follow-up period (D29 to D76), accompanied by Grade 2 ascites or judged by the investigator as requiring re-supplementation with human serum albumin, re-treatment will be administered. The participant will remain blinded and re-treated with the investigational product (rHSA or pHSA) according to their randomization at the time of initial treatment, with a dosing regimen of 20 g/day, administered intravenously once daily for 7 days or until serum albumin ≥ 35 g/L (whichever comes first). If a participant undergoes re-treatment, relevant safety and efficacy tests should be completed during the re-treatment period (i.e., the period of re-dosing, not exceeding 7 days), including on day 1 of re-treatment (RD1), RD3 (if dosing is received on the same day), RD7 (if dosing is received on the same day), or the last day of re-treatment. Visits during the re-

	treatment period are conducted independently and do not affect the visits scheduled for the follow-up period. All participants received their last visit on D90±7.<br>This Phase III study is planned to conduct one unblinded interim analysis when at least 328 participants have completed the D7 visit. The interim analysis will be performed by the Independent Data Monitoring Committee (IDMC), while the entire project team remains blinded. Further details regarding the interim analysis will be provided in the IDMC Charter and Statistical Analysis Plan (SAP).<br>During the trial, participants will have blood samples collected at specified time points for PD and immunogenicity analysis.<br><br>**Selection criteria and basis for the trial population:**<br>Hepatic cirrhosis is a chronic condition characterized by diffuse hepatic fibrosis and the formation of regenerative nodules resulting from various types of chronic liver injury, primarily manifesting as hepatocellular dysfunction and portal hypertension. Patients with decompensated cirrhosis may experience complications such as ascites, esophageal and gastric variceal bleeding, and hepatic encephalopathy. Among these, ascites is the most common complication in patients with cirrhosis, which can further progress to severe complications including spontaneous bacterial peritonitis (SBP) and hepatorenal syndrome (HRS), potentially leading to fatal outcomes. The most common mechanism for the development of ascites in cirrhosis is portal hypertension. Additional mechanisms include hypoalbuminemia and decreased plasma oncotic pressure resulting from impaired hepatic synthesis function, as well as abnormalities in the neuroendocrine system.<br>Albumin (ALB) is synthesized by hepatocytes and accounts for approximately 50% to 60% of the total plasma protein content. It possesses the physical properties of expanding circulating blood volume and maintaining plasma oncotic pressure (COP), along with various biological functions. Plasma-derived Human Serum Albumin (pHSA), the medicinal preparation of ALB, is widely used in clinical practice. It is utilized not only for the management of patients with various acute and critical illnesses but also frequently for the treatment of ascites, cerebral edema, and hypoalbuminemia.<br>pHSA is widely used in patients with cirrhotic ascites. Multiple guidelines recommend the use of pHSA for the prevention and treatment of post-paracentesis circulatory dysfunction (PPCD) following large-volume paracentesis (LVP), short-term infusion therapy for hospitalized patients with cirrhotic ascites, and long-term treatment for outpatients. According to the *Rapid Recommendation Guidelines for the Use of Plasma-derived Human Serum Albumin (pHSA) in the Treatment of Hepatic*

	Cirrhosis, pHSA is recommended for patients without ascites whose plasma ALB level is below 25 g/L, or for patients with ascites whose plasma ALB level is below 30 g/L. Furthermore, the dosage of pHSA should be individualized based on the patient's actual clinical status. For patients with severe grade 3 ascites (mostly refractory ascites), paracentesis is a rapid and effective treatment to relieve abdominal distension. Guidelines recommend that serial large-volume paracentesis (4–6 L/day) supplemented with pHSA (8 g per 1000 mL of ascites removed) is more effective and associated with fewer complications than the use of diuretics alone.<br>Results from the structural characterization studies of the investigational drug, recombinant human serum albumin (rHSA), demonstrate that its molecular structure and molecular weight are identical to those of pHSA. Preclinical pharmacodynamic studies suggest that rHSA can significantly reduce ascites volume in animals with cirrhosis; it also shows an effect of increasing plasma colloid osmotic pressure in both model animals and normal animals, and exhibits a certain dose-related effect. Its preclinical pharmacodynamic characteristics are similar to those of commercially available active control pHSA.<br>The Phase Ia clinical trial of the investigational drug in healthy individuals has been completed. This study was conducted as a single ascending dose (SAD) study across 6 dose groups (2 g, 5 g, 10 g, 20 g, 30 g, and 40 g). participants in each group were randomized at a 3:1 ratio to receive the investigational drug (rHSA) or the placebo (sodium chloride injection). The study results demonstrated that the investigational drug at doses of 2 to 40 g was safe and well-tolerated in healthy participants. Adverse events that occurred were all manageable, and immunogenicity was low. Serum albumin levels increased after administration compared to pre-dose levels. Within the dose range of 5 to 40 g, the magnitude of increase in serum albumin level was greater in higher dose groups, and the investigational drug increased plasma colloid osmotic pressure (COP) in the human body. Generally, within 0 to 72 hours post-dose, higher serum albumin levels were associated with higher plasma COP, and the results of plasma COP showed a certain correlation with PK results.<br>A Phase Ib clinical study of the investigational drug in patients with cirrhosis and Grade 1–2 ascites has also been completed. The study involved dose escalation across 3 dose groups (10 g/day, 20 g/day, and 30 g/day), where participants were randomized to receive the investigational drug (rHSA) or the comparator (pHSA). The results showed that the investigational drug had a favorable safety and efficacy profile at doses of 10 g/day to 30 g/day. Safety results indicated no significant difference in the incidence of TEAEs between the investigational drug group (rHSA) and the comparator group (pHSA), and no investigational product-related SAEs was reported

	during the entire study period. Efficacy and PD analysis results showed that the investigational drug effectively increased serum albumin level and COP in patients with cirrhotic ascites, and reduced abdominal circumference, body weight, and ascites depth. Furthermore, at equivalent dose levels, no significant differences between the investigational drug group and the comparator group regarding the proportion of participants with serum albumin level ≥ 35 g/L and the duration were observed within 14 days after the first intravenous administration. The trend of change in plasma COP over time was also similar between the two groups. In conclusion, rHSA demonstrated good comparability with pHSA, as well as a favorable safety and efficacy profile in patients with cirrhosis and Grade 1–2 ascites.<br>This study will use the change in serum albumin levels from baseline at the end of treatment as the primary efficacy endpoint. To minimize the impact of procedures for refractory ascites, such as paracentesis, on the evaluation of the investigational drug's efficacy, this study requires enrolled patients with cirrhotic ascites to have Grade 1–2 ascites, and serum albumin < 30 g/L at screening.<br><br>**The selection and basis of the dosing regimen:**<br>Hypoalbuminemia is a significant pathophysiological attribute of cirrhosis and a critical contributor to cirrhotic ascites. According to the package insert of marketed plasma-derived human serum albumin (pHSA) injection, for the treatment of patients with hypoalbuminemia, the dosage should be administered as needed based on the degree of albumin deficiency. However, the maximum daily safe dose should not exceed 2 g of albumin per kg of body weight; for instance, for an adult weighing 60 kg, the daily albumin infusion dose should not exceed 120 g. According to the *Rapid Advice Guideline on Plasma-derived Human Serum Albumin in the Treatment of Cirrhosis*, pHSA is recommended for patients without ascites whose plasma ALB level is below 25 g/L, or for patients with ascites whose plasma ALB level is below 30 g/L. The appropriate dosage of pHSA may be selected within the range of 10 g to 40 g/day based on the patient's actual clinical condition.<br>A Phase Ib multiple ascending dose (MAD) clinical study of the investigational drug has been completed in patients with cirrhosis and Grade 1–2 ascites. The study included three dose groups (10 g/day, 20 g/day, and 30 g/day), with doses increasing in ascending order. participants were randomly assigned in a 2:1 ratio to receive either the investigational drug (rHSA) or the comparator (pHSA), administered intravenously once daily for 14 consecutive days or until serum albumin reached ≥35 g/L (whichever occurred first). The study results demonstrated that the investigational drug (rHSA) exhibited favorable safety and efficacy in patients with cirrhotic ascites

	and showed good comparability with the comparator (pHSA). Safety results showed that at doses ranging from 10 g/day to 30 g/day, no significant difference in the incidence of TEAEs was observed between the investigational drug group (rHSA) and the comparator group (pHSA). Furthermore, no significant differences in TEAE incidence were observed between the test and comparators across the different dose cohorts. No SAEs related to the investigational product occurred during the entire study period. Efficacy and pharmacodynamic analysis results indicated that at doses of 10 g/day to 30 g/day, there were no significant differences between the investigational drug group and the comparator group regarding the proportion of participants achieving serum albumin level ≥ 35 g/L within 14 days after the first intravenous administration, nor were there significant differences in the median time to achieve this level. Both drugs effectively increased serum albumin levels and reduced abdominal circumference, body weight, and ascites depth. Additionally, the trends in plasma colloid osmotic pressure changes over time were similar between the two groups. At a dose of 10 g/day, the investigational drug already demonstrated favorable therapeutic efficacy. At this dose level, the majority of participants (66.7%) received treatment for 14 days. At the completion of the last dose, the mean increase in serum albumin level from baseline in the investigational drug group was 11.0 g/L. Abdominal circumference, body weight, and ascites depth were all significantly reduced from baseline. Specifically, the maximum reductions from baseline in abdominal circumference and body weight during the treatment period were 3.46 cm and 2.5 kg, respectively. By Day 14 after the first dose, the ascites depth in the investigational drug group decreased by 25.13 mm from baseline. By Day 17 after the first dose, plasma COP increased by 6.00 mmHg from baseline. At a dose of 20 g/day, within the Full Analysis Set (FAS), the proportion of participants in the investigational drug group achieving a serum albumin level ≥ 35 g/L was 6 participants (75%), with a median time of 8 days. At the completion of the last dose, the mean increase in serum albumin level from baseline was 9.2 g/L. The maximum reductions from baseline in abdominal circumference and body weight in the investigational drug group during the treatment period were 6.55 cm and 6 kg, respectively. By Day 14 after the first dose, ascites depth decreased by 27.63 mm from baseline, and plasma COP increased by 4.25 mmHg from baseline. At a dose of 30 g/day, the median time to achieve a serum albumin level ≥ 35 g/L was 6 days for the investigational drug group and 4 days for the comparator group. Due to premature discontinuation of dosing by participants, the actual total administered dose in the investigational drug group at this dose level was not significantly higher than that in other dose cohorts, resulting in efficacy and PD parameters that were not superior to the other two dose cohorts.

	Based on the clinical use of pHSA, two dosing regimens were established for the investigational drug and comparator groups in the Phase II stage of this study: 10 g/day for 14 consecutive days, and 20 g/day for 7 consecutive days. The study results demonstrated a favorable safety profile for the investigational drug. There were no significant differences in the incidence of TEAEs and TRAEs between the investigational drug and comparator groups, nor was there any significant dose correlation. Compared to the 10 g/day dose group, the 20 g/day dose group showed a more significant change in serum albumin levels from baseline at the completion of the final intravenous dose. Additionally, participants in the 20 g/day cohort achieved serum albumin levels ≥ 35 g/L faster and in a higher proportion. To rapidly correct hypoalbuminemia in patients with cirrhotic ascites and shorten the duration of hospitalization, the Phase III stage planned to select 20 g/day administered for 7 consecutive days as the dosing regimen.<br><br>**Selection and Rationale for Primary and Key Secondary Endpoints in Phase III**<br>Currently, human albumin injection products on the market have not undergone strictly defined registrational confirmatory clinical trials. There are few clinically relevant study results available for reference, and there are no existing regulations or guiding principles to direct the selection of primary endpoints for pivotal clinical trials of recombinant albumin products. According to the *Rapid Recommendation Guidelines for the Use of Human Albumin in the Treatment of Cirrhosis* and other relevant clinical guidelines, the direct purpose of administering human albumin in patients with cirrhotic ascites and hypoalbuminemia is to increase serum albumin level. Currently, there is a widely applied and recognized formula for calculating albumin dosage in clinical practice: Required dose (g) = (Target albumin level - Current level) (g/L) * 2 * Plasma volume (L). Through this calculation formula, it is evident that the change in serum albumin level from baseline can most intuitively and reliably evaluate the equivalence between the investigational drug and the comparator. Additionally, other recombinant albumin drugs currently undergoing Phase II or III clinical trials in China also use changes in albumin levels from baseline as the primary efficacy evaluation indicator. Therefore, in Phase III of this study, the change in serum albumin level from baseline, confirmed by immediate albumin testing after the final intravenous administration (based on central laboratory test results), will be used as the primary endpoint to evaluate whether the investigational drug is equivalent to the comparator.<br>Ascites is a common and serious complication in patients with decompensated cirrhosis and an important marker of the natural progression of cirrhosis. Once ascites

	occurs, the 1-year mortality rate is about 15%, and the 5-year mortality rate is about 44% to 85%. Therefore, controlling ascites is the most important treatment goal for these patients. This study will enroll patients with cirrhotic ascites. The therapeutic objectives of the investigational drug include not only correcting the patients' albumin levels but also improving their clinical symptoms, such as resolution of ascites. Clinical measurements of abdominal circumference and body weight can be influenced by various factors (such as breathing, eating status, bowel movements, etc.), potentially resulting in significant discrepancies from actual values, and both can only indirectly reflect the resolution of ascites. Changes in ascites depth measured by abdominal ultrasound can directly and objectively reflect whether patients with cirrhotic ascites derive clinical benefit from ascites resolution; therefore, in Phase III of this study, the ascites improvement rate will be used as a key secondary efficacy endpoint.<br>Considering that there are currently no clinical guidelines or expert consensus defining clinically meaningful criteria for ascites improvement, and that this study includes patients with grade 1 or 2 ascites, the extent and speed of ascites resolution after treatment may vary between patients with different grades of ascites. Therefore, this study will use the rate of ascites improvement at the completion of the final intravenous administration (defined as resolution of ascites in patients with baseline grade 1 ascites, downgrading of ascites in patients with baseline grade 2 ascites, or a reduction in ascites depth by ≥ 25% compared to baseline) as a key secondary endpoint to evaluate the clinical benefit to patients.<br>During the study, each research center should conduct abdominal ultrasound examinations and ascites depth measurements in accordance with the unified standard operating procedures to minimize measurement errors. For detailed requirements, please refer to the document *SOP for Ascites Assessment by Abdominal Ultrasound*.<br><br>**Pharmacodynamics (PD) Study:**<br>Albumin has multiple physiological functions, including maintaining plasma colloid osmotic pressure. When pHSA is used in patients with cirrhosis and ascites, it increases plasma colloid osmotic pressure, thereby promoting reabsorption of ascites and preventing further fluid leakage, indirectly achieving the therapeutic goal of controlling ascites progression.<br>In the Phase Ib clinical trial of multiple ascending doses of the investigational product in patients with liver cirrhosis and Grade 1–2 ascites, participants in both the investigational product group and the comparator group had increases from baseline in plasma colloid osmotic pressure at doses of 10 g/day and 20 g/day, and the

	time-profile trends were similar between the two groups. At a dose of 10 g/day, the colloid osmotic pressure before intravenous administration on Day 14 (D14) was 17.78 and 15.20 mmHg in the investigational drug group and comparator group, respectively. At this time, serum albumin levels were 29.9 and 27.7 g/L, respectively. Immediately after intravenous administration on D14, colloid osmotic pressure was 17.16 and 15.30 mmHg, respectively, with serum albumin levels of 31.1 and 28.5 g/L, respectively. At the 20 g/day dose, participants in the investigational product group and comparator group had plasma colloid osmotic pressures of 12.15 and 19.40 mmHg, respectively, prior to the start of intravenous administration on Day 7 (D7), with corresponding serum albumin levels of 20.7 and 31.6 g/L. Immediately after completion of intravenous administration on D7, plasma colloid osmotic pressures in the two groups were 13.00 and 18.60 mmHg, respectively, with corresponding serum albumin levels of 24.1 and 32.8 g/L. The above study results indicate that colloid osmotic pressure is correlated with serum albumin level, and colloid osmotic pressure increases with rising serum albumin level.<br>Therefore, to further explore the correlation between plasma colloid osmotic pressure and serum albumin levels, this trial will evaluate pharmacodynamic (PD) characteristics by measuring plasma colloid osmotic pressure and its change from baseline on Day 1, Day 7 (Day 7 only for participants in the 20 g/day group), and Day 14 (Day 14 only for participants in the 10 g/day group).The specific time points and windows for PD blood sample collection are described in Section 6.7.<br>During the trial, the PD blood sample collection time points may be adjusted based on the PD results obtained.<br><br>**Immunogenicity Study:**<br>In the Phase II part of this trial, all participants who received at least one dose of the investigational product must have blood samples collected for immunogenicity assessment at the following time points: prior to the start of investigational product intravenous infusion on Day 1, Day 8, Day 15 (Day 15 only for participants in the 10 g/day group), Day 29, Day 57, and the early termination visit (if applicable).<br>In Phase III of this trial, all participants who received at least one dose of the investigational product must undergo blood sampling for immunogenicity assessment at the following time points: prior to the start of intravenous infusion of the investigational product on Day 1, Day 90, or the early termination visit (if applicable). The sampling window for anti-drug antibodies (ADA) is the same as the corresponding visit window. Immunogenicity will be assessed by evaluating the incidence and titers of anti-rhHSA and anti-host protein (HCP) antibodies (if

	applicable).<br><br>**Establishment of IDMC:**<br>An Independent Data Monitoring Committee (IDMC) will be established during the Phase III stage of this trial to conduct interim analyses and regularly review the progress, safety data, and other important information from the clinical trial. The IDMC shall have a written operating procedure and retain all meeting records, as detailed in the IDMC Charter.
**Trial Population**	The trial population of this study consists of patients with cirrhotic ascites. Ascites grade must be 1–2, and serum albumin (ALB) level must be < 30 g/L.
**Sample Size**	**Phase II:**<br>Phase II of this study is a dose-exploratory phase and does not involve formal hypothesis testing. Currently, there are no efficacy data or reference literature for similar drugs. Based on preliminary data accumulated from the target patient population, a total of 92 participants are planned to be enrolled in the Phase II stage. Participants will be stratified by baseline serum albumin levels (< 25 or ≥ 25 g/L) and randomly allocated in a 1:1:1:1 ratio to the investigational drug 10 g/day group (23 cases), investigational drug 20 g/day group (23 cases), comparator drug 10 g/day group (23 cases), and comparator drug 20 g/day group (23 cases).<br><br>**Phase III:**<br>In the Phase III stage of this trial, the primary efficacy endpoint will be the change from baseline in serum albumin level, as confirmed by an immediate albumin assessment following completion of the final intravenous administration (based on central laboratory results). Referring to experience from studies of similar products, Phase II data of the investigational drug, and expert opinion, the equivalence margin for this Phase III study is set at –2 to 2 g/L. The standard deviation of the primary efficacy endpoint is expected to be no greater than 5 g/L, and the investigational drug is anticipated to have similar efficacy to the comparator. Under a two one-sided test (TOST) procedure with a significance level of 0.025 per side, a total sample size of 328 participants (164:164) will provide > 90% power.<br>In Phase III of this study, the ascites improvement rate at completion of the final intravenous administration will be used as a key secondary efficacy endpoint, with a non-inferiority assessment conducted. Ascites improvement at the individual level is defined as: resolution of ascites in participants with Grade 1 ascites at baseline; or downgrading of ascites grade or a reduction in measured ascites depth of ≥ 25% from baseline in participants with Grade 2 ascites at baseline. The ascites improvement rate is defined as the proportion of participants in the investigational drug group or the

	comparator group who meet the predefined criteria for ascites improvement. At a one-sided significance level of 0.025, -10% is proposed as the non-inferiority margin. Results from the Phase II study of the investigational drug showed that the ascites improvement rates at completion of the final intravenous administration were 60.87% and 50.00% in the 20 g/day investigational drug group and the 20 g/day comparator group, respectively. The between-group difference in ascites improvement rate (investigational drug group minus comparator group; without adjustment for covariates, using Fisher's exact test) was 10.9%. When adjusted for baseline ALB levels (< 25 g/L or ≥ 25 g/L), baseline ascites depth (< 3 cm; 3–6 cm [including 3, excluding 6]; or 6–10 cm [including both 6 and 10]), and baseline use of diuretics (yes or no) as covariates, the between-group difference in ascites improvement rate (investigational drug group minus comparator group) was 5.8%. Therefore, considering the results of similar products and the Phase II study of the investigational drug, assuming a 50% ascites improvement rate in the comparator group and an estimated between-group difference in ascites improvement rate (investigational drug group - comparator group) of 5%, a sample size of 348 for participants would achieve 80% power. By accounting for a 10% dropout rate, a total of 388 participants (194:194) were planned to be enrolled.<br>This Phase III study is planned to conduct one unblinded interim analysis when at least 328 participants have completed the D7 visit. The interim analysis will be performed by the IDMC, while the entire project team remains blinded. Further details regarding the interim analysis will be provided in the IDMC Charter and Statistical Analysis Plan (SAP).
**Enrollment Criteria**	**Inclusion criteria:**<br>**Patients meeting all the following criteria may be considered for enrollment:**<br>1) Agreeing to comply with the treatment regimen and visit schedule, willing to be enrolled and signing the Informed Consent Form (ICF).<br>2) Aged ≥ 18 years and ≤ 70 years on the date of signing the informed consent form, regardless of gender; body mass index (BMI) within the range of 17.0 to 29.0 kg/m^2 (inclusive of boundary values).<br>3) Patients clinically diagnosed with decompensated cirrhotic ascites, with ascites graded as level 1–2 confirmed by ultrasound abdomen during the screening period, and meeting the criterion of serum albumin (ALB) < 30 g/L.<br>4) Men and women of childbearing potential (women of childbearing potential include premenopausal women and those within 2 years of menopause) who are willing to use highly effective contraception (condom, contraceptive sponge, gel,

	film, intrauterine device, oral or injected contraceptives, subcutaneous implants, etc.) from signing the informed consent form through 3 months after the last dose of the investigational drug; women of childbearing potential must have a negative pregnancy test ≤ 7 days before the first dose of the investigational product.<br><br>**Exclusion criteria:**<br>**Patients meeting any of the following criteria should be excluded:**<br>1) Individuals with a known history of allergy/allergic reaction to yeast or yeast-derived products, or allergy to any component of the investigational formulation; those with an allergic predisposition (multiple drug or food allergies), a history of allergy to biological products, a history of severe systemic allergic reactions due to other causes, and considered by the investigator to be unsuitable for treatment with the investigational drug.<br>2) Severe digestive system diseases and complications present at screening that, in the investigator's opinion, render the patient unsuitable for participation in this study, including but not limited to malignant ascites, hepatic encephalopathy diagnosed as Grade 3 or 4 according to the West-Haven criteria, portal vein tumor thrombus/thrombosis, post-paracentesis circulatory dysfunction, biliary obstructive disease confirmed by ultrasound or other imaging examinations, hemorrhage of digestive tract with hemostasis achieved for less than 10 days following treatment, failure to achieve effective hemostasis via endoscopic variceal ligation, or conditions assessed by the investigator as posing a high risk of bleeding during the trial (e.g., endoscopy performed within 3 months prior to screening indicating severe esophageal and gastric varices with positive red color sign).<br>3) Active cardiovascular disease or history thereof at screening, or other conditions judged by the investigator as unsuitable for treatment with human blood albumin, specifically including but not limited to hypertension (systolic blood pressure > 140 mmHg or diastolic blood pressure > 90 mmHg, except where judged by the investigator to be well-controlled and stable), severe anemia, acute heart disorder , severe cardiopulmonary or structural heart disorder, severe arrhythmia, decompensated cardiac failure (with normovolemia or hypervolemia), unstable angina, infarct myocardial within 6 months prior to screening, tachycardia/bradycardia requiring pharmacological treatment, atrioventricular block third degree, etc.<br>4) Active metabolic system disease or history thereof at screening (excluding

patients with stable glycaemia control as judged by the investigator), or concurrent renal impairment considered by the investigator to be unsuitable for treatment with serum albumin.

5) Severe pre-existing disease present at screening that, in the investigator’s opinion, render the patient unsuitable for participation in this study, including but not limited to active malignancies (including hepatocellular carcinoma [HCC]), pulmonary edema, bleeding tendency or active bleeding disorders, uncontrolled infections (including active spontaneous bacterial peritonitis [SBP]), thyroid function abnormal (Grade ≥ 3 according to the National Cancer Institute Common Terminology Criteria for Adverse Events[NCI CTCAE] version 5.0), and effusion pleural that, as judged by the investigator, may require treatment or is participant to change throughout the trial.
6) The patient had the following laboratory test abnormality:
   - ✓ Bone marrow function: absolute neutrophil count (ANC) < 1.0×10^9/L; platelet count (PLT) < 20×10^9/L; hemoglobin (HGB) < 70 g/L;
   - ✓ Liver function: Alanine aminotransferase (ALT) > 5 × ULN (Upper Limit of Normal); Aspartate aminotransferase (AST) > 5 × ULN; Serum total bilirubin (TBIL) > 4 × ULN, or judged by the investigator as unsuitable for participation in the trial.
   - ✓ Renal function: serum creatinine > 3 × ULN, protein urine positive, and judged by the investigator as unsuitable for participation in the trial.
   - ✓ Coagulation function: prothrombin time (PT) prolonged > 5 s.
7) Individuals who have received human plasma products (including pHSA) within 7 days prior to the first administration of the investigational product; those with a history of organ transplant; those who require or plan to undergo interventional invasive examinations or treatments during the study period.
8) Those who have participated in another clinical trial of a new drug or medical device within 30 days prior to screening, or are currently participating in such a trial at the time of screening, and have received the investigational drug/investigational treatment.
9) Individuals with a positive test for human immunodeficiency virus (HIV) antibodies.
10) Pregnant or lactating women;
11) Any other reason judged by the investigator as unsuitable for participation in this study.

**Criteria for Treatment Termination**	During the trial, participants may withdraw consent and request to discontinue study treatment. When a participant requests to withdraw from the study treatment, every effort should be made to have them complete the early termination visit. Additionally, the investigator may discontinue the investigational product treatment for the participant in the following circumstances:<br>➢ The investigator believes that participants should terminate the study treatment due to safety concerns (such as the occurrence of intolerable adverse events).<br>➢ During the treatment period, the participant's serum albumin level meets the discontinuation criteria (ALB ≥ 40 g/L); or during the re-treatment period within the follow-up phase, the participant's serum albumin level meets the discontinuation criterion (ALB ≥ 35 g/L).<br>➢ The participant is pregnant;<br>➢ The participant has a serious protocol violation, which, in the opinion of the investigator, has seriously affected the evaluation of the primary endpoint of the study, and the investigator considers that the participant's treatment should be discontinued.<br>➢ Other circumstances where the investigator deems that the participant's treatment should be terminated.
**Study Grouping**	In the Phase II stage, participants will be stratified during the screening period based on baseline serum albumin (< 25 or ≥ 25 g/L) and randomized in a 1:1:1:1 ratio to the following four groups:<br>➢ Group 1: Investigational drug 10 g/day, administered continuously for 14 days;<br>➢ Group 2: Investigational drug 20 g/day, administered continuously for 7 days;<br>➢ Group 3: Comparator drug 10 g/day, administered continuously for 14 days;<br>➢ Group 4: Comparator drug 20 g/day, administered continuously for 7 days.<br>Based on the Phase II study results and investigator opinions, participants in the Phase III stage will be stratified during the screening period based on baseline human serum albumin level (< 25 or ≥ 25 g/L), baseline ascites grade (< 3 cm; 3-6 cm [inclusive of 3, exclusive of 6]; 6-10 cm [inclusive of 6 and 10]), and baseline diuretic use (yes or no), and randomized in a 1:1 ratio to the following two groups:<br>➢ Group A: Investigational drug 20 g/day, administered continuously for 7 days;<br>➢ Group B: Comparator drug 20 g/day, administered continuously for 7 days.

**Investigational drugs**	**1)Investigational drug**<br>**Drug**: Recombinant Human Serum Albumin Injection<br>**Specification**: 10 g/vial (20%, 50 mL)<br>**Administration method**: The investigational drug is not diluted. Calculate the volume according to the dose required by the participant and perform intravenous infusion. The intravenous infusion time for 10 g of investigational drug is 30 min (± 5 min) (excluding flushing time after infusion). The intravenous infusion time for 20 g of investigational drug is 1 h (± 10 min) (excluding flushing time after infusion). Flush the line after intravenous infusion ends; flushing time is 3–6 min. The entire intravenous administration is completed when flushing ends. The specific dosing regimens are shown in the "Drug Management Manual".<br>**Storage condition:** 2–8°C, protected from light<br>**Validity period:** 24 months<br>**Provider:** Shenzhen Protgen Co., Ltd.<br>**2) Comparator:**<br>**Drug: Plasma-derived Human Serum Albumin Injection**<br>**Specification:** 10 g/vial (20%, 50 mL)<br>**Administration method:** The comparator is not diluted. Calculate the volume according to the dose required by the participant and perform intravenous infusion. The intravenous infusion time for 10 g of comparator is 30 min (±5 min) (excluding flushing time after infusion). The intravenous infusion time for 20 g of comparator is 1 h (±10 miadminin) (excluding flushing time after infusion). Flush the line after intravenous infusion ends; flushing time is 3–6 min. The entire intravenous administration is completed when flushing ends. The specific dosing regimens are shown in the "Drug Management Manual".<br>**Storage condition:** 2–8°C, protected from light<br>**Validity period:** 60 months<br>**Provider:** Shenzhen Protgen Co., Ltd.
**EVALUATION ENDPOINTS**	**Efficacy Endpoints**<br>**Phase II:**<br>➢ **Primary efficacy endpoints:**<br>Change from baseline in serum albumin level, as confirmed by albumin test immediately after the completion of the final intravenous administration.<br>➢ **Secondary efficacy endpoints:**<br>1) Change from baseline in ascites depth after administration.<br>2) Proportion of participants with serum albumin level ≥ 35 g/L, confirmed by albumin test at the completion of the final intravenous administration.

	3) Time to achieve serum albumin level ≥ 35 g/L.<br>4) Change from baseline in abdominal circumference after administration.<br>5) Change from baseline in body weight after administration.<br>➢ **Exploratory endpoints:** Change from baseline in glycated albumin levels after administration.<br>**Phase III:**<br>➢ **Primary efficacy endpoint:**<br>Change from baseline in serum albumin level, as confirmed by albumin test immediately after the completion of the final intravenous administration (based on central laboratory test results).<br>➢ **Key secondary efficacy endpoints:**<br>Ascites improvement rate at the completion of the final intravenous administration, with ascites improvement defined as: resolution of ascites for participants with Grade 1 ascites at baseline, or downgrading of ascites in participants with Grade 2 ascites at baseline, or a ≥ 25% reduction in ascites depth measurement from baseline.<br>➢ **Secondary efficacy endpoints:**<br>1) Change from baseline in serum albumin level, as confirmed by local laboratory test immediately after the completion of the final intravenous administration.<br>2) Change from baseline in ascites depth at the completion of the final intravenous administration.<br>3) Proportion of participants with serum albumin level ≥ 35 g/L, confirmed by albumin test at the completion of the final intravenous administration.<br>4) Time to achieve serum albumin level ≥ 35 g/L.<br>5) Change from baseline in abdominal circumference at the completion of the final intravenous administration.<br>6) Change from baseline in body weight at the completion of the final intravenous administration.<br>For participants who receive retreatment with the investigational product from Day 29 to Day 76, efficacy endpoints also include:<br>1) Change from baseline before retreatment in serum albumin level confirmed by albumin test immediately after completion of the final intravenous administration during the retreatment period.<br>2) Ascites improvement rate at the completion of the final intravenous administration during the retreatment period, with ascites improvement during the retreatment period defined as: resolution of ascites in participants with Grade

	1 ascites at baseline of the retreatment period, or downgrading of ascites in participants with Grade 2 ascites at baseline of the retreatment period, or a ≥ 25% reduction in ascites depth measurement from baseline.<br>3) Change from baseline before retreatment in ascites depth at the completion of the final intravenous administration during the retreatment period.<br>4) Proportion of participants with serum albumin level ≥ 35 g/L as confirmed by albumin test during the retreatment period.<br>5) Time to serum albumin level ≥ 35 g/L confirmed by albumin test during the retreatment period.<br>6) Change from baseline before retreatment in abdominal circumference at the completion of the final intravenous administration during the retreatment period.<br>7) Change from baseline before retreatment in body weight at the completion of the final intravenous administration during the retreatment period.<br>➢ **Exploratory endpoints:** Change from baseline in glycated albumin levels and glycated hemoglobin levels after administration.<br><br>**Safety Endpoints**<br>The safety endpoints of this trial include the type, incidence, and severity of adverse events. Adverse events will be graded according to the National Cancer Institute Common Terminology Criteria for Adverse Events (NCI CTCAE) Version 5.0. The relationship of adverse events to the investigational product will be assessed by the investigator in accordance with the protocol-specified attribution assessment criteria. During the trial, participants are required to undergo safety assessments at specified time points, including laboratory tests (hematology, urinalysis, blood chemistry, coagulation function tests, etc.), vital signs, 12-lead electrocardiogram (ECG), physical examination, fundus examination, thyroid function tests, thyroid ultrasound, and iodine uptake test (applicable only to Phase III), among others.<br>Specific safety endpoints include, but are not limited to, the following:<br>1) Any reported and all directly observed adverse events;<br>2) Any abnormal changes in vital signs and physical examinations;<br>3) Treatment-emergent clinically significant abnormal laboratory test results, clinically significant abnormal hematology test results, etc.<br>**PD Endpoints**<br>The PD endpoint of this trial is plasma colloid osmotic pressure (COP). PD characteristics will be evaluated by assessing plasma COP and its change from baseline following intravenous administration of the investigational product.<br>**Immunogenicity Endpoints**

The immunogenicity endpoints of this trial are the incidence and titers (if applicable) of anti-drug antibodies (ADA) against recombinant human serum albumin (rHSA) and against host cell proteins (HCP). Immunogenicity will be evaluated by assessing the incidence and titers (if applicable) of ADAs following administration of the investigational product.

ESTIMAND

Primary Estimand

- **Clinical question of primary interest:** whether the change from baseline in serum albumin level immediately after completion of the final intravenous administration of rHSA (based on central laboratory test results) is equivalent to that of plasma-derived human serum albumin (pHSA) in patients with cirrhotic ascites and hypoalbuminemia.
- **Attributes of the Primary Estimand**
  - **Target population:** patients with cirrhotic ascites and hypoalbuminemia who meet the inclusion criteria and none of the exclusion criteria.
  - **Target variable:** change from baseline in serum albumin level immediately after completion of the final intravenous administration.
  - **Treatment:** participants are randomized to receive either rHSA or pHSA at 20 g/day for 7 consecutive days or until serum albumin level reaches the discontinuation criterion (ALB ≥ 40 g/L), whichever occurs first.
  - **Intercurrent Events and Addressing Strategies**

Intercurrent event	Addressing strategies	Note
Premature treatment discontinuation due to inadequate increase in albumin level after treatment	Composite strategy	Data collected after treatment discontinuation are not used in the primary estimand analysis. For the resulting missing primary efficacy endpoint data, the multiple imputation (MI) method will be applied to impute values before reanalysis, using data from more than 5% of participants with the worst on-treatment efficacy (based on the change from baseline in serum albumin) within each corresponding treatment group. The minimum sample size required for model convergence will be determined based on actual data, with the number of participants increased in steps of 5
Premature treatment discontinuation due to adverse event	Treatment strategy	Data collected after premature treatment discontinuation will be included in the primary estimand analysis
Premature treatment discontinuation due to other reasons, e.g., participant withdrawal of informed consent	Treatment strategy	Data collected after premature treatment discontinuation will be included in the primary estimand analysis

Increase in baseline dose of diuretics during the treatment period; concomitant use of other albumin, blood products, or prohibited medications/treatments; death	Composite strategy	Data collected after treatment discontinuation are not used in the primary estimand analysis. For the resulting missing primary efficacy endpoint data, the multiple imputation (MI) method will be applied to impute values before reanalysis, using data from more than 5% of participants with the worst on-treatment efficacy (based on the change from baseline in serum albumin) within each corresponding treatment group. The minimum sample size required for model convergence will be determined based on actual data, with the number of participants increased in steps of 5

- **Population-level summary:** The difference between groups in the least squares mean of the change from baseline in serum albumin level, along with its 95% confidence interval (CI). The analysis will be based on a Covariance model, with baseline serum albumin as a covariate and including randomization stratification factors.

Key Secondary Estimand

➢ **Clinical question of interest:** Whether the ascites improvement rate at the completion of the final intravenous administration of rHSA is non-inferior to that of pHSA in patients with cirrhotic ascites and hypoalbuminemia.

➢ **Attributes of the Estimand**

- **Target population:** patients with cirrhotic ascites and hypoalbuminemia who meet the inclusion criteria and none of the exclusion criteria.
- **Target variable:** ascites improvement rate at the completion of the final intravenous administration.
- **Treatment:** participants are randomized to receive either rHSA or pHSA at 20 g/day for 7 consecutive days or until serum albumin level reaches the discontinuation criterion (ALB ≥ 40 g/L), whichever occurs first.
- **Intercurrent Events and Addressing Strategies**

Intercurrent event	Addressing strategies	Note
Premature treatment discontinuation due to inadequate improvement in ascites after treatment	Composite strategy	Considered as no improvement in ascites depth
Premature treatment discontinuation due to adverse event	Treatment strategy	Data collected after premature treatment discontinuation will be included in the primary estimand analysis
Premature treatment discontinuation due to other reasons, e.g., participant withdrawal of informed consent	Treatment strategy	Data collected after premature treatment discontinuation will be

<table>
<tr><td></td><td>
<table>
<tr><td></td><td></td><td>included in the primary estimand analysis</td></tr>
<tr><td>Increase in baseline dose of diuretics during the treatment period; concomitant use of other albumin, blood products, or prohibited medications/treatments; death</td><td>Composite strategy</td><td>Considered as no improvement in ascites depth</td></tr>
</table>
Population-level summary: The difference in the ascites improvement rate between groups (investigational drug group and comparator group) along with its 95% CI.
Other Efficacy Endpoints
Statistical analyses will be performed in accordance with the general principles of statistical analysis, as detailed in the Statistical Analysis Plan (SAP).
</td></tr>
<tr><td>Statistical Hypotheses and Sample Size Estimation</td><td>
The Phase II stage of this trial will enroll approximately 92 participants. Participants will be randomized in a 1:1:1:1 ratio into two dose groups of the investigational product and two dose groups of the comparator product, using block randomization stratified by baseline serum albumin (< 25 or ≥ 25 g/L). The Phase II stage will evaluate the efficacy of rHSA in increasing serum albumin levels in patients with cirrhotic ascites, as well as the dose–response relationship. The final sample size will be based on actual enrollment.
An equivalence hypothesis test will be employed for the Phase III stage. The primary efficacy endpoint is the change from baseline in serum albumin level (as determined by the central laboratory) immediately after the completion of the final intravenous administration, to verify the clinical equivalence of investigational drug (rHSA) to the comparator product (pHSA). Based on Phase III clinical trial designs of similar products both domestically and internationally, and expert recommendations, the equivalence margin is set at ± 2 g/L.
Based on prior experience with similar products and Phase II data of the investigational product, the standard deviation of the primary efficacy endpoint is expected not to exceed 5 g/L for both the investigational and reference products, and the efficacy of the investigational product is anticipated to be similar to that of the comparator. Under a two one-sided tests procedure at a one-sided significance level of 0.025, a sample size of 328 participants (164 per group) will provide 90% power.
In Phase III of this study, the ascites improvement rate at completion of the final intravenous administration will be used as a key secondary efficacy endpoint, with a non-inferiority assessment conducted. Ascites improvement at the individual level is defined as: resolution of ascites in participants with Grade 1 ascites at baseline; or downgrading of ascites grade or a reduction in measured ascites depth of ≥ 25% from
</td></tr>
</table>

	baseline in participants with Grade 2 ascites at baseline. The ascites improvement rate is defined as the proportion of participants in the investigational drug group or the comparator group who meet the predefined criteria for ascites improvement. At a one-sided 0.025 significance level, -10% is proposed as the non-inferiority margin. Results from the Phase II study of the investigational drug showed that the ascites improvement rates at completion of the final intravenous administration were 60.87% and 50.00% in the 20 g/day investigational drug group and the 20 g/day comparator group, respectively. The between-group difference in ascites improvement rate (investigational drug group minus comparator group; without adjustment for covariates, using Fisher's exact test) was 10.9%. When adjusted for baseline ALB levels (< 25 g/L or ≥ 25 g/L), baseline ascites depth (< 3 cm; 3-6 cm [inclusive of 3, exclusive of 6]; or 6-10 cm [inclusive of 6 and 10]), and baseline use of diuretics (yes or no) as covariates, the between-group difference in ascites improvement rate (investigational drug group minus comparator group) was 5.8%. Therefore, after comprehensive consideration of data from similar products and the Phase II study results of the investigational drug, and assuming an ascites improvement rate of 50% in the control group, a conservatively estimated difference in ascites improvement rates (experimental group minus control group) of 5%, and a dropout rate of 10%, a total of 388 participants are required to achieve a power of more than 84%.<br>The methodology for sample size re-estimation is described in detail in the Interim Analysis section.
**Interim Analysis**	This Phase III trial is planned to conduct one unblinded interim analysis when at least 328 participants have completed the D7 visit (i.e., when data for the primary efficacy endpoint are collected). The interim analysis will perform an equivalence hypothesis test on the primary efficacy endpoint and conduct sample size re-estimation based on the effect size of the secondary key efficacy endpoint(s). The interim analysis will be executed by the IDMC. Details will be specified in the Statistical Analysis section and the IDMC Charter.<br>The decision for sample size re-estimation will be made following the method described by Cyrus R. Mehta in the referenced literature, as detailed below:<br>Assume the maximum allowable total sample size (n_{max}) for the entire trial is set to twice the initial sample size (388 participants), i.e., 776 participants (388 per treatment group). According to literature, the Minimal Conditional Power (CP_{min}) set to 0.36 and the Unconditional Power (UP) to 80% (where UP = 1-β, β being the type II error

	rate). The values of CP_{min} and UP divide the interval [0, 1] into three zones:<br>•Unfavorable Zone: The Conditional Power (CP) calculated based on the interim analysis results is lower than CP_{min}. In this case, the sample size will not be adjusted.<br>•Promising Zone: The CP calculated based on the interim analysis results is greater than CP_{min} but less than UP. In this case, the sample size will be increased based on the interim results to achieve a final analysis power of at least 80%, but the final adjusted sample size will not exceed n_{max}.<br>•Favorable Zone: The CP calculated based on the interim analysis results is greater than UP. In this case, the sample size will not be adjusted, and the trial will continue with the initial sample size.
**Statistical Analyses**	All statistical analyses will be performed using SAS version 9.4 or higher. In general, continuous variables will be described statistically using the number of participants, mean, median, standard deviation, minimum, and maximum values. Categorical and ordinal variables will be described statistically using the frequency and percentage of each category or rank. Unless otherwise specified, missing values will not be included in the calculation of percentages.<br>No formal hypothesis testing will be performed in Phase II; however, hypothesis testing will be conducted for exploratory purposes, employing various statistical methods for exploratory data analysis to enhance understanding. Unless otherwise stated, all statistical tests will utilize a two-sided confidence level of $\alpha = 0.05$, with the calculation of two-sided 95% confidence intervals (CIs).<br>For the primary efficacy endpoint in Phase III, an equivalence hypothesis test will be employed, with the change in serum albumin level from baseline immediately after the completion of the final intravenous administration as the primary efficacy endpoint. This test aims to verify that the clinical efficacy of the investigational drug (rHSA) is equivalent to that of the comparator (pHSA). The proposed equivalence range is -2 to 2 g/L. Additionally, during Phase III of this study, a non-inferiority assessment will be conducted on the key secondary efficacy endpoint, which is the improvement rate of ascites at the completion of the final intravenous administration. A non-inferiority margin of -10% is proposed for this assessment.<br>Apart from the primary and key secondary endpoints, the analysis of other efficacy endpoints will be based on observed data (i.e., missing data will not be imputed). Detailed statistical analysis methods for this study are outlined in the Statistical Analysis Plan (SAP).<br>**Statistical analysis population**<br>➢ **Full Analysis Set (FAS):** All randomized participants who have received at least

	one dose of the investigational product will be included in the FAS. This analysis set will be utilized for the primary analysis of patient distribution, demographic, and baseline characteristics, as well as for the primary efficacy analysis in both Phase II and Phase III.<br>➢ **Per-Protocol Set (PPS):** The PPS comprises all randomized participants who have received at least one dose of the investigational product, have not committed major protocol violations, and have not received treatments that could significantly affect the efficacy assessment. The PPS will be employed for supplementary efficacy analyses in both Phase II and Phase III.<br>➢ **Safety Set (SS):** All randomized participants who have received at least one dose of the investigational product and have undergone at least one post-dose safety evaluation will be included in the SS. The SS represents the safety evaluation population for this trial.<br>➢ **Pharmacodynamics (PD) Analysis Set:** All randomized participants who have received at least one dose of the investigational product and have at least one post-dose effective plasma colloid osmotic pressure data point will be included in the PD analysis set. This set will be utilized for pharmacodynamic analyses.<br>➢ **Immunogenicity Analysis Set:** All randomized participants who received at least one dose of the investigational product and have at least one post-dose immunogenicity assessment will be included in the Immunogenicity Analysis Set. This set will be used for immunogenicity analysis.<br>➢ **Retreatment Analysis Set:** All Phase III participants who received retreatment with the investigational product will be included in the Retreatment Analysis Set. This set will be used for safety and efficacy evaluations of retreatment.<br><br>**Demographic data and other baseline characteristics**<br>Descriptive statistics will be employed to analyze demographic data and other baseline characteristics (including disease characteristics, treatment history, medication history, etc.).<br>**Efficacy analysis**<br>During Phase II of this trial, descriptive statistics will be utilized to assess efficacy. The changes in serum albumin level from baseline were summarized immediately after the final intravenous administration of the investigational drug and the comparator at each dose, as confirmed by albumin testing. Additionally, changes in ascites depth, abdominal girth, and body weight from baseline at each post-initial intravenous administration visit will be summarized for comparisons between the investigational drug and comparator groups at each dose level. The proportions of

participants achieving serum albumin levels of 35 g/L or higher at the last dose, along with their corresponding confidence intervals (CIs), will be calculated separately for the two dose groups of the investigational drug, the two dose groups of the comparator, and for comparisons between the investigational drug and comparator groups at each dose level. The difference in proportions and its associated two-sided 95% confidence interval will also be computed, and a bar chart illustrating these proportions will be generated. The Kaplan-Meier method will be used to generate plots illustrating the time to achieve serum albumin levels of 35 g/L or higher for the investigational drug and comparator groups, as well as for comparisons between these groups at each dose level. The median, first quartile, and third quartile values for these times will also be calculated.

Phase III Primary Estimand Analysis:

- **Primary Estimation Method:** in Phase III of this trial, the primary endpoint is the change in serum albumin from baseline measured immediately after the completion of the final intravenous administration, with equivalence margins set at -2 g/L and 2 g/L. An analysis of covariance (ANCOVA) model will be employed, with the change in serum albumin from baseline as the dependent variable, treatment group as a fixed effect, and ALB level (continuous outcome), baseline ascites depth (< 3 cm; 3-6 cm [inclusive of 3, exclusive of 6]; or 6-10 cm [inclusive of 6 and 10]), and whether diuretics were used at baseline (yes or no) as covariates for analysis. The least squares means (LSM) and standard errors (SE) for the change in serum albumin from baseline immediately after the final intravenous administration, along with their 95% CIs, will be calculated for both groups. Additionally, the difference in LSMs between the groups (investigational drug group – comparator group) and its corresponding 95% CI will be computed.
- **Sensitivity analysis, supplementary analysis, and subgroup analysis:**

Category	Description of analytical procedure
Sensitivity Analysis 1	An ANOVA model will be employed, with the treatment group as a fixed effect and without considering covariates; all other aspects will align with the primary estimation analysis method.
Supplementary Analysis 1	For the intercurrent events—premature treatment discontinuation due to poor efficacy or adverse events, death, increased baseline diuretic dose during the treatment period, or concurrent administration of other albumin, blood products, or prohibited medications/therapies during the treatment period—a hypothetical strategy shall be applied. Under this approach, efficacy data collected after the occurrence of an intercurrent

	event will not be used in the analysis, and the resulting missing data will not be imputed. The analysis will instead adopt a Mixed-effects Model for Repeated Measures (MMRM).
Supplementary Analysis 2	With respect to intercurrent events—encompassing premature treatment discontinuation due to lack of efficacy or adverse events, increase in the basal dose of concomitant diuretics during treatment, or concurrent administration of other albumin products, blood products, or prohibited medications/therapies during the treatment period—a treatment policy strategy shall be applied. Under this paradigm, efficacy data collected subsequent to the occurrence of intercurrent events are retained and incorporated into the analysis.
Supplementary Analysis 3	Analyses will be conducted on the PPS, with all other methodological aspects remaining identical to those of the primary estimand analysis.
Subgroup analysis	Subgroup analyses will be conducted according to randomization stratification factors and salient baseline characteristics—including, but not limited to, baseline serum albumin level (< 25 or ≥ 25 g/L), baseline ascites depth (< 3 cm; 3 to < 6 cm; or 6 to 10 cm [inclusive of 6 and 10]), and baseline diuretic use (yes or no)—following the same analytical methodology as that employed for the primary estimand analysis.

Phase III Key Secondary Estimand Analysis

- **Primary Estimation Method:**The key secondary efficacy endpoint for Phase III of this trial is defined as the improvement rate of ascites upon completion of the final intravenous administration, for which a non-inferiority margin of –10% has been prespecified. Between-group comparisons will be adjusted for randomization stratification factors, including baseline serum albumin level (< 25 or ≥ 25 g/L), baseline ascites depth (< 3 cm, 3 to < 6 cm; or 6 to 10 cm [inclusive of 6 and 10]), and baseline diuretic use (yes or no). The difference in response rates between treatment groups, together with its two-sided 95% confidence interval, will be estimated using the Newcombe method.
- **Sensitivity analysis, supplementary analysis, and subgroup analysis:**

**Category**	**Description of analytical procedure**
Sensitivity Analysis 1	Analyses will be performed without correction for stratification factors, while all other methodological components remain

	congruent with those delineated in the primary estimand analysis.
Supplementary Analysis 1	Analyses will be conducted on the PPS, with all other methodological aspects remaining identical to those of the primary estimand analysis.
Subgroup analysis	Subgroup analyses will be conducted contingent upon randomization stratification factors and salient baseline characteristics—encompassing, though not limited to, baseline serum albumin level (< 25 or ≥ 25 g/L), baseline ascites depth (< 3 cm; 3-6 cm [inclusive of 3, exclusive of 6]; or 6-10 cm [inclusive of 6 and 10]), and baseline diuretic use (yes or no).

<u>Phase III Secondary Efficacy Endpoint Analyses</u>

The changes from baseline in ascites depth, abdominal circumference, and body weight following administration—will be evaluated using the same primary estimation methodology (ANCOVA) as specified for the primary estimand. For each endpoint, the change from baseline will be designated as the dependent variable, with treatment group entered as a fixed effect. Covariates will encompass the baseline value of the respective endpoint, baseline serum albumin level (< 25 or ⩾ 25 g/L), baseline ascites depth (< 3 cm; 3-6 cm [inclusive of 3, exclusive of 6]; or 6-10 cm [inclusive of 6 and 10]), and baseline diuretic use (yes or no). In the analysis of ascites depth, baseline ascites depth will be excluded from the covariate set. Temporal trajectories of these endpoints will be graphically depicted. Additionally, the proportion of participants attaining a serum albumin level ≥ 35 g/L, as confirmed by laboratory assessment at the completion of the final intravenous administration, will be computed along with its corresponding confidence interval. The between-group difference in this proportion (investigational drug group-comaprator group) and its associated two-sided 95% CI will likewise be estimated. A bar chart illustrating these proportions will be constructed. Time to the first achievement of serum albumin level ≥ 35 g/L (confirmed by laboratory assessment) will be analysed using the Kaplan-Meier (KM) method, and the corresponding KM curves will be provided.

For the retreatment period, the change from pre-retreatment baseline in serum albumin level at the completion of the final intravenous administration will be assessed using an analysis of covariance (ANCOVA) model. The change from pre-retreatment baseline in serum albumin will serve as the dependent variable, treatment group as a fixed effect, and pre-retreatment baseline serum albumin level as a covariate. For each group, the least squares mean (LSM) and standard error (SE) of the change will be estimated, along with the between-group difference in the change from pre-retreatment baseline and its two-sided 95% confidence interval. Statistical

	analyses for other efficacy endpoints during the retreatment period will be conducted in a manner consistent with those employed during the primary treatment period.<br><br>**Safety Analysis**<br>Adverse events will be encoded using the Medical Dictionary for Regulatory Activities (MedDRA). All analyses of adverse events will be predicated upon treatment-emergent adverse events (TEAEs). All TEAEs, investigational drug-related TEAEs, and serious adverse events (SAEs) were summarized and analyzed by system organ classification (SOC) and preferred term (PT), and the number of occurrences and incidence rates were calculated. Descriptive statistics were used to summarize changes in vital signs, ECG parameters, laboratory tests, thyroid tests, etc., and their changes relative to baseline. Shift tables were used to describe the changes in their clinical significance relative to baseline. Descriptive statistics will be used to summarize changes in iodine uptake testing (if applicable, only for Phase III) and its relative to the initial test, and corresponding shift tables will be generated to illustrate clinically meaningful deviations relative to the initial measurement.<br>**Pharmacodynamic analysis**<br>For the Phase II study, descriptive statistical analyses will be undertaken to summarise plasma colloid osmotic pressure (COP) values, as well as changes from baseline in COP after the first intravenous administration. These analyses will be performed separately for both dose levels of the investigational drug and both dose levels of the comparator, and additionally, at each dose level, for the comparison between the investigational product and the comparator.<br>In the Phase III study, descriptive statistical analyses will be conducted to summarise plasma colloid osmotic pressure (COP) values and their corresponding changes from baseline after the first intravenous administration, comparing the investigational product group with the comparator group.<br>**Immunogenicity Analysis**<br>The incidence and titers of anti-rHSA and anti-HCP antibodies will be summarized (if applicable). If applicable, the impact of ADAs on the PD, effectiveness, and safety of rHSA will be assessed.<br>**Exploratory Endpoint Analysis**<br>In Phase II, descriptive statistical analyses will be performed on the change from baseline in glycated albumin for the two dose levels of the investigational product and the two dose levels of the comparator, as well as for the comparison between the investigational product and the comparator at each dose level.<br>In Phase III, descriptive statistical analyses will be performed on the change from

	baseline in glycated albumin and glycated hemoglobin for the investigational product group (Group A) and the comparator group (Group B).

Trial flowchart (Phase II 10 g/day dose groups [Group 1 and Group 3])

Study day/procedures	Screening period	Treatment Period (D1–D14)						Follow-up period (D15~D57)			Early Discontinuation Visit[N]
	D-28–D-1	D1	D2	D3	D7	D8	D14/EOT[M]	D15	D29	D57	
**Window**	/	/	/	/	/	/	/	/	± 3 days	±5 days	+7
**Visit**	1	2	3	4	5	6	7	8	9	10	/
Signing of the Informed Consent Form	X										
Demographic information collection (including height, weight, etc.)	X										
Medical history collection	X										
Treatment History Collection	X										
Inclusion/Exclusion Criteria Assessment	X										
Infectious Disease Screening [A]	X[C]										
Pregnancy Test (Females of Childbearing Potential Only)	X[C]									X	X
Participant Randomization[B]	X										
Investigational product Dosing		X[E]									

Study day/procedures	Screening period	Treatment Period (D1–D14)						Follow-up period (D15~D57)			Early Discontinuation Visit[N]
	D-28–D-1	D1	D2	D3	D7	D8	D14/EOT[M]	D15	D29	D57	
**Window**	/	/	/	/	/	/	/	/	± 3 days	±5 days	+7
**Visit**	1	2	3	4	5	6	7	8	9	10	/
Albumin Assessment[D]	X[C]	X[H]									
vital signs	X[C]	X[F]							X	X	X
Waist circumference and weight measurement		X[G]							X	X	X
Abdominal Ultrasound Assessment	X[C]			X	X		X		X	X	X
Physical examinations	X[C]		X		X		X		X	X	X
Hematology[I]	X[C]		X	X	X		X		X	X	X
Blood Chemistry[I]	X[C]		X	X[G]	X[G]		X[G]		X	X	X
Coagulation Assay[I]	X[C]			X	X		X		X	X	X
Hemoglobin A1c (HbA1c) test	X[C]						X[C]				
Glycated Albumin test		X[C]					X[C]				
Urinalysis[I]	X[C]			X	X		X		X	X	X
Stool routine[I]	X[C]										
12-lead ECG	X[C]			X	X		X		X	X	X
ECG	X[C]										
Fundus examination	X[C]									X	X
Thyroid function tests[J]	X[C]						X		X	X	X
Thyroid Ultrasound	X[C]						X		X	X	X
PD blood sampling[k]		X					X				
ADA blood sample		X				X		X	X	X	X

Study day/procedures	Screening period	Treatment Period (D1–D14)						Follow-up period (D15~D57)			Early Discontinuation Visit[N]
	D-28–D-1	D1	D2	D3	D7	D8	D14/EOT[M]	D15	D29	D57	
**Window**	/	/	/	/	/	/	/	/	± 3 days	±5 days	+7
**Visit**	1	2	3	4	5	6	7	8	9	10	/
sampling[L]											
Recording of concomitant medications	X	X						X			
Record of adverse events	X	X						X			

Note:

A. All participants undergoing screening shall undergo infectious disease screening during the screening period. The assessments include hepatitis B panel (HBsAg, HBsAb, HBeAg, HBcAb, HBeAb; not required for participants with a documented history of hepatitis B), quantitative HBV DNA (to be performed only in participants positive for hepatitis B surface antigen or with a documented history of hepatitis B), HCV antibody testing (not required for participants with a documented history of hepatitis C), HCV RNA testing (to be performed only in participants positive for HCV antibody or with a documented history of hepatitis C), HIV antibody testing, and syphilis testing.

B. On Day -1, the inclusion/exclusion criteria will be reviewed. Participants who meet the eligibility requirements will be randomized using stratified block randomization.

C. For abdominal ultrasound, hematology, blood chemistry, coagulation assays, urinalysis, stool routine, 12-lead ECG, pregnancy test, albumin assessment, HbA1c, and echocardiography performed during the screening period, results obtained within 1 week prior to the start of the first intravenous administration of the investigational product are acceptable (including results obtained prior to the signing of informed consent up to 1 week before the first dose); otherwise, these assessments must be repeated within the 1-week window prior to dosing. For all other safety assessments—vital signs, physical examination, infectious disease screening (including HBsAg, HBsAb, HBeAg, HBcAb, HBeAb, quantitative HBV DNA, HCV antibody, HCV RNA, HIV antibody, syphilis testing), fundoscopy, thyroid function tests, and thyroid ultrasound—results obtained within 28 days prior to the first intravenous administration of the investigational product are acceptable (including results obtained prior to the signing of informed consent up to 28 days before the first dose). HbA1c and glycated albumin assessments are applicable solely to Phase II and will be collected during the screening period and on Day 1 of the treatment period prior to the first dose.

D. During screening, serum albumin (ALB) level will be monitored. A participant is deemed eligible for enrolment if the ALB value is below 30 g/L. Once the participant enters the treatment period, no further assessment of inclusion/exclusion criteria will be performed; therefore, if the ALB level is ≥ 30 g/L on Day 1

prior to dosing, this does not constitute screening failure, and the participant shall proceed to dosing in the treatment period. During the treatment period, commencing from Day 1, blood samples for albumin assessment will be collected within 1 hour prior to each intravenous administration and immediately after completion of the final intravenous administration (+ 3 minutes). If a study site does not offer individual albumin testing, the investigator may select an alternative laboratory panel that includes albumin assessment.

E. All randomized participants will receive daily intravenous doses of either the investigational product or the comparator at the specified dose levels, once daily for 14 consecutive days. During infusion, special attention shall be paid to whether participants experience infusion-related adverse reactions or clinical signs and symptoms associated with cardiovascular volume overload (e.g., headache, jugular vein distension, dyspnoea). Based on the treatment discontinuation criteria and the actual clinical situation, the investigator shall determine whether the infusion should be temporarily interrupted or permanently discontinued (see Section 5.4.1 "Dosage and Administration of the investigational product").

F. On each day of investigational product infusion, vital signs shall be monitored during the infusion period, with assessments conducted once within 2 hours prior to infusion and once at 30 minutes after completion of infusion (+30 minutes).

G. Prior to the first administration of the investigational product on D1, abdominal circumference and body weight shall be measured three times, and the results shall be recorded as baseline values. During the treatment period, abdominal circumference and body weight measurements shall be performed after completion of each intravenous administration. For participants who do not complete the 14-day treatment period (including those who meet the criteria for treatment discontinuation or who terminate treatment early), abdominal circumference and body weight measurements shall be performed at the D14 visit.

H. Eligibility for dosing during the treatment period shall be determined based on albumin (ALB) test results. If ALB < 40 g/L, the participant shall receive the planned intravenous administration; otherwise, dosing shall not be performed. It is not necessary to wait for the albumin test result obtained on the same day to determine whether treatment discontinuation criteria have been met before deciding whether to administer the dose on that day. Once it is known that the participant's serum albumin level has reached the treatment discontinuation criterion, no further dosing shall be administered. If the investigator determines that the albumin test result is abnormal (potentially due to systematic instrument error, sampling error, or transient increases or decreases in albumin levels related to clinical signs or disease conditions), one repeat test is permitted, and the repeat result shall prevail.

I. During the screening period, if any laboratory test value (except for albumin) exceeds the exclusion criteria, but the investigator determines that the abnormality may be related to systematic instrument error or sampling error (e.g., failure to maintain fasting status on the day of testing), one repeat test is permitted. If the repeat value still exceeds the exclusion criteria, the participant shall be considered a screening failure and will not meet the inclusion criteria for this study. Based on the investigator's judgment, the participant may undergo re-screening after receiving appropriate symptomatic treatment. For details, see Section 4.5 "Screening Failure and Re-screening".

J. Thyroid function assessments primarily include thyroxine (T4), triiodothyronine (T3), thyroid-stimulating hormone (TSH), free T3, and free T4.

K. Multiple pharmacodynamic (PD) blood sample collections will be conducted in this study. Detailed sampling time points and time windows are described in Section 6.7 "PD Assessments".

L. Immunogenicity blood samples will be collected at the following time points: prior to the start of intravenous administration of the investigational product on D1, and on D8, D15, D29, D57, and at the early discontinuation visit (if applicable). The sampling time windows shall be consistent with the corresponding visit time windows.

M. If a participant discontinues treatment prematurely during the treatment period, an End of Treatment (EOT) visit shall be completed within 24 hours after the investigator's decision to discontinue treatment. The assessments and procedures at the EOT visit shall be the same as those at the D14 visit. participants who complete the EOT visit are not required to undergo duplicate assessments at D14.

N. For all randomized participants who have received at least one intravenous administration of the investigational product, if the participant withdraws from the study prematurely, an early discontinuation visit shall be conducted within 7 days after the decision to withdraw. Based on the investigator's judgment, assessment results obtained within 3 days prior to the decision to withdraw may be used as early discontinuation visit data, and repeat assessments are not required.

Study flow diagram (phase II, 20 g/day group [group 2 and group 4])

Study day/procedures	Screening period	Treatment period (D1-D7)				Follow-up period (D8-D57)				Early Discontinuation Visit[N]
	D-28–D-1	D1	D2	D3	D7/EOT[M]	D8	D14	D29	D57	
**Window**	/	/	/	/	/	/	/	± 3 days	±5 days	+7
**Visit**	1	2	3	4	5	6	7	8	9	/
Signing of the Informed Consent Form	X									
Demographic information collection (including height, weight, etc.)	X									
Medical history collection	X									
Treatment History Collection	X									
Inclusion/Exclusion Criteria Assessment	X									
Infectious Disease Screening [A]	X[C]									
Pregnancy Test (Females of Childbearing Potential Only)	X[C]								X	X
Participant Randomization[B]	X									
Investigational product Dosing		X[E]								
Albumin Assessment[D]	X[C]	X[H]								
vital signs	X	X[F]					X	X	X	X
Waist circumference and weight measurement		X[G]						X	X	X
Ultrasound abdomen (ascites depth assessment)	X[C]			X	X			X	X	X
Physical examinations	X		X		X		X	X	X	X
Hematology[I]	X[C]		X	X	X		X	X	X	X
Blood Chemistry[I]	X[C]		X	X[G]	X[G]		X	X	X	X
Coagulation Assay[I]	X[C]			X	X			X	X	X

Study day/procedures	Screening period	Treatment period (D1-D7)				Follow-up period (D8-D57)				Early Discontinuation Visit[N]
	D-28–D-1	D1	D2	D3	D7/EOT[M]	D8	D14	D29	D57	
**Window**	/	/	/	/	/	/	/	± 3 days	±5 days	+7
**Visit**	1	2	3	4	5	6	7	8	9	/
Hemoglobin A1c (HbA1c) test		X[C]						X	X	X
Glycated Albumin test		X[C]			X					
Urinalysis[I]	X[C]			X	X			X	X	X
Stool routine[I]	X[C]									
12-lead ECG	X[C]			X	X			X	X	X
ECG	X[C]									
Fundus examination	X[C]								X	X
Thyroid function tests[J]	X[C]				X			X	X	X
Thyroid Ultrasound	X[C]				X			X	X	X
PD blood sampling[k]		X			X					
ADA blood sample sampling[L]		X[L]				X		X	X[L]	X[L]
Recording of concomitant medications	X	X								X
Record of adverse events	X	X								X

Note:

A. All participants undergoing screening shall undergo infectious disease screening during the screening period. The assessments include hepatitis B panel (HBsAg, HBsAb, HBeAg, HBcAb, HBeAb; not required for participants with a documented history of hepatitis B), quantitative HBV DNA (to be performed only in participants positive for hepatitis B surface antigen or with a documented history of hepatitis B), HCV antibody testing (not required for participants with a documented history of hepatitis C), HCV RNA testing (to be performed only in participants positive for HCV antibody or with a documented history of hepatitis C), HIV antibody testing, and syphilis testing.

B. On Day -1, the inclusion/exclusion criteria will be reviewed. Participants who meet the eligibility requirements will be randomized using stratified block randomization.

C. During the screening period, results of ultrasound abdomen, hematology, blood biochemistry, coagulation tests, urinalysis, stool routine, 12-lead ECG, pregnancy test, albumin test, glycosylated hemoglobin test, and echocardiography obtained within 1 week prior to the first intravenous administration of the investigational

product (including results obtained before signing the informed consent form up to 1 week prior to first dosing of investigational product) are acceptable; otherwise, these assessments must be repeated within 1 week prior to dosing. Other safety assessments (including vital signs, physical examination, infectious disease screening [HBsAg, HBsAb, HBeAg, HBcAb, HBeAb, HBV DNA quantification, HCV antibody, HCV RNA, HIV antibody, and syphilis testing], fundi examination, thyroid function tests, and ultrasound thyroid) may use results obtained within 28 days prior to the first intravenous administration of the investigational product (including results obtained before signing the informed consent form up to 28 days prior to first dosing).

D. During the screening period, albumin (ALB) levels will be monitored. If the monitored ALB value is < 30 g/L, the participant meets the enrollment criteria. After entering the treatment period, participants will no longer be evaluated against inclusion or exclusion criteria. Therefore, if a participant has an albumin level ≥ 30 g/L prior to dosing on D1, this does not constitute a screening failure, and the participant shall enter the treatment period and receive dosing. During the treatment period, starting from D1, samples for albumin testing will be collected within 1 hour prior to each intravenous administration and immediately after completion of the final intravenous administration (+ 3 minutes).

E. All randomized participants will receive intravenous administration of the investigational drug or comparator at the specified dose, once daily for 7 consecutive days. During infusion, special attention shall be paid to whether participants experience infusion-related adverse reactions or clinical signs and symptoms associated with cardiovascular volume overload (e.g., headache, jugular vein distension, dyspnea). Based on the treatment discontinuation criteria and the actual clinical situation, the investigator shall determine whether the infusion should be temporarily interrupted or permanently discontinued (see Section 5.4.1 "Dosage and Administration of the investigational product").

F. On each day of investigational product infusion, vital signs shall be monitored during the infusion period, with assessments conducted once within 2 hours prior to infusion and once at 30 minutes after completion of infusion (+ 30 minutes).

G. Prior to the first administration of the investigational product on D1, abdominal circumference and body weight shall be measured three times, with an interval of more than 1 minute between measurements, and the results shall be recorded as baseline values. During the treatment period, abdominal circumference and body weight measurements shall be performed after completion of each intravenous administration. For participants who do not complete the 7-day treatment period (including those who meet the criteria for treatment discontinuation or who terminate treatment early), abdominal circumference and body weight measurements shall be performed at the D7 visit.

H. Eligibility for dosing during the treatment period shall be determined based on albumin (ALB) test results. If ALB < 40 g/L, the participant shall receive the planned intravenous administration; otherwise, dosing shall not be performed. It is not necessary to wait for the albumin test result obtained on the same day to determine whether treatment discontinuation criteria have been met before deciding whether to administer the dose on that day. Once it is known that the participant's serum albumin level has reached the treatment discontinuation criterion, no further dosing shall be administered. If the investigator determines that the albumin test result is abnormal (potentially due to systematic instrument error, sampling error, or transient increases or decreases in albumin levels related to clinical signs or disease conditions), one repeat test is permitted, and the repeat result shall prevail.

I. During the screening period, if any laboratory test value (except for albumin) exceeds the exclusion criteria, but the investigator determines that the abnormality

may be related to systematic instrument error or sampling error (e.g., failure to maintain fasting status on the day of testing), one repeat test is permitted. If the repeat value still exceeds the exclusion criteria, the participant shall be considered a screening failure and will not meet the inclusion criteria for this study. Based on the investigator's judgment, the participant may undergo re-screening after receiving appropriate symptomatic treatment. For details, see Section 4.5 "Screening Failure and Re-screening".

J. Thyroid function assessments primarily include thyroxine (T4), triiodothyronine (T3), thyroid-stimulating hormone (TSH), free T3, and free T4.

K. Multiple pharmacodynamic (PD) blood sample collections will be conducted in this study. Detailed sampling time points and time windows are described in Section 6.7 "PD Assessments".

L. During the Phase II stage of this study, immunogenicity blood samples will be collected at the following time points: prior to the start of intravenous administration of the investigational product on D1, and on D8, D29, D57, and at the early discontinuation visit (if applicable). The sampling time windows shall be consistent with the corresponding visit time windows.

M. If a participant discontinues treatment prematurely during the treatment period, an End of Treatment (EOT) visit shall be completed within 24 hours after the investigator's decision to discontinue treatment. The assessments and procedures at the EOT visit shall be the same as those at the D7 visit. participants who complete the EOT visit are not required to undergo duplicate assessments at D7.

N. For all randomized participants who have received at least one intravenous administration of the investigational product, if the participant withdraws from the study prematurely, an early discontinuation visit shall be conducted within 7 days after the decision to withdraw. Based on the investigator's judgment, assessment results obtained within 3 days prior to the decision to withdraw may be used as early discontinuation visit data, and repeat assessments are not required.

Trial Flowchart (Phase III, Group A and Group B)

**Study day/procedures**	**Screening period**	**Treatment period (D1-D7)**				**Follow-up Period (D8~D90)**				**Early Discontinuation Visit[N]**
	**D-28–D-1**	**D1**	**D2**	**D3**	**D7/EOT[M]**	**D14**	**D29**	**D57**	**D90**	
**Window**	/	/	/	/	/	/	**± 3 days**	**±5 days**	**± 7 days**	**+7**
**Visit**	**1**	**2**	**3**	**4**	**5**	**6**	**7**	**8**	**9**	**/**
Signing of the Informed Consent Form	X									
Demographic information collection (including height, weight, etc.)	X									
Medical history collection	X									
Treatment History Collection	X									
Inclusion/Exclusion Criteria Assessment	X									
Infectious Disease Screening [A]	X[C]									
Pregnancy Test (Females of Childbearing Potential Only)	X[C]								X	X
Participant Randomization[B]	X									
Investigational product Dosing		X[E]								
Albumin Assessment[D]	X[C]	X[H]								
vital signs	X	X[F]				X	X	X	X	X
Waist circumference and weight measurement		X[G]					X	X	X	X
Ultrasound abdomen (ascites depth assessment)	X[C]			X	X		X	X	X	X
Physical examinations	X		X		X	X	X	X	X	X
Hematology[I]	X[C]		X	X	X	X	X	X	X	X
Blood Chemistry[I]	X[C]		X	X	X[G]	X	X	X	X	X

Study day/procedures	Screening period	Treatment period (D1-D7)				Follow-up Period (D8~D90)				Early Discontinuation Visit[N]
	D-28–D-1	D1	D2	D3	D7/EOT[M]	D14	D29	D57	D90	
Window	/	/	/	/	/	/	± 3 days	±5 days	± 7 days	+7
Visit	1	2	3	4	5	6	7	8	9	/
Coagulation Assay[I]	X[C]			X	X		X	X	X	X
Hemoglobin A1c (HbA1c) test		X[C]					X		X	X
Glycated Albumin test		X[C]			X					
Urinalysis[I]	X[C]			X	X		X	X	X	X
Stool routine[I]	X[C]									
12-lead ECG	X[C]			X	X		X	X	X	X
ECG	X[C]									
Fundus examination	X[C]								X	X
Thyroid function tests[J]	X[C]				X		X	X	X	X
Thyroid Ultrasound	X[C]				X		X	X	X	X
Iodine intake test [O]	X[O]				X[O]		X[O]	X[O]	X[O]	X[O]
PD blood sampling[k]		X			X					
ADA blood sample sampling[L]		X[L]							X[L]	X[L]
Judgment of retreatment [P]								X		
Recording of concomitant medications	X					X[P]				X
Record of adverse events	X					X				X

Note:

A. All participants undergoing screening shall undergo infectious disease screening during the screening period. The assessments include hepatitis B panel (HBsAg, HBsAb, HBeAg, HBcAb, HBeAb; not required for participants with a documented history of hepatitis B), quantitative HBV DNA (to be performed only in participants positive for hepatitis B surface antigen or with a documented history of hepatitis B), HCV antibody testing (not required for participants with a documented history of hepatitis C), HCV RNA testing (to be performed only in participants positive for HCV antibody or with a documented history of hepatitis

C), HIV antibody testing, and syphilis testing.

B. On Day -1, the inclusion/exclusion criteria will be reviewed. Participants who meet the eligibility requirements will be randomized using stratified block randomization.

C. Abdominal ultrasound, hematology, blood chemistry, coagulation function, urinalysis, stool routine, 12-lead electrocardiogram, pregnancy test, albumin test, and echocardiogram during the screening period are acceptable if the results are within one week before the first intravenous administration of the investigation product (including results from before signing the informed consent form to one week before the first administration of the investigational drug). Otherwise, the tests need to be repeated within one week before administration. Other safety assessments (including vital signs, physical examination, infectious disease screening [HBsAg, HBsAb, HBeAg, HBcAb, HBeAb, HBV DNA quantification, HCV antibody, HCV RNA, HIV antibody, and syphilis testing], fundi examination, thyroid function tests, and ultrasound thyroid) may use results obtained within 28 days prior to the first intravenous administration of the investigational product (including results obtained before signing the informed consent form up to 28 days prior to first dosing). Glycated hemoglobin (GHbA1c) testing and baseline GHbA1c testing were performed before the first administration on day 1 of treatment.

D. During the screening period, albumin (ALB) levels will be monitored. If the monitored ALB value is < 30 g/L, the participant meets the enrollment criteria. After entering the treatment period, participants will no longer be evaluated against inclusion or exclusion criteria. Therefore, if a participant has an albumin level ≥ 30 g/L prior to dosing on D1, this does not constitute a screening failure, and the participant shall enter the treatment period and receive dosing. During the treatment period, starting from D1, samples for albumin testing will be collected within 1 hour prior to each intravenous administration and immediately after completion of the final intravenous administration (+ 3 minutes). In addition to albumin testing in local laboratories, samples need to be collected before administration on D1, immediately after administration on D7, or during an EOT visit and sent to the central laboratory for albumin testing. If a study site does not offer individual albumin testing, the investigator may select an alternative laboratory panel that includes albumin assessment.

E. All randomized participants will receive intravenous administration of the investigational drug or comparator at the specified dose, once daily for 7 consecutive days. During infusion, special attention should be paid to whether the participant develops infusion-related adverse reactions or clinical symptoms and manifestations related to cardiovascular overload (such as headache, carotid artery engorgement, dyspnea, etc.), and the infusion should be suspended or stopped based on the discontinuation criteria and the actual situation (see Section 5.4.1 "Dosage and administration of the investigational drug" for details).

F. On the day of each infusion of the investigational drug, vital signs monitoring must be performed during the infusion. Specifically, the monitoring should be performed once within 2 hours (h) before the infusion and once 30 minutes (+ 30 minutes) after the infusion is completed.

G. Prior to the first administration of the investigational product on D1, abdominal circumference and body weight shall be measured three times, with an interval of more than 1 minute between measurements, and the results shall be recorded as baseline values. During the treatment period, abdominal circumference and body weight measurements shall be performed after completion of each intravenous administration. For participants who do not complete the 7-day treatment period (including those who meet the criteria for treatment discontinuation or who terminate treatment early), abdominal circumference and body weight measurements shall be performed at the D7 visit.

H. Eligibility for dosing during the treatment period shall be determined based on albumin (ALB) test results. If ALB < 40 g/L, the participant shall receive the planned intravenous administration; otherwise, dosing shall not be performed. It is not necessary to wait for the albumin test result obtained on the same day to determine whether treatment discontinuation criteria have been met before deciding whether to administer the dose on that day. Once it is known that the participant's serum albumin level has reached the treatment discontinuation criterion, no further dosing shall be administered. If the investigator determines that the albumin test result is abnormal (potentially due to systematic instrument error, sampling error, or transient increases or decreases in albumin levels related to clinical signs or disease conditions), one repeat test is permitted, and the repeat result shall prevail.

I. During the screening period, if a laboratory test value (except for albumin) exceeds the exclusion criteria, but the investigator determines that the abnormality may be related to systematic instrument error or sampling error (e.g., the patient did not fast as required on the day of the test), a retest is allowed. If the retest value still exceeds the exclusion criteria, the participant does not meet the inclusion criteria for this trial and fails the screening. Depending on the investigator's judgment, the participant may participate in the screening again after receiving certain symptomatic treatment. See Section 4.5 "Requirements for screening failure and rescreening" for details.

J. Thyroid function assessments primarily include thyroxine (T4), triiodothyronine (T3), thyroid-stimulating hormone (TSH), free T3, and free T4.

K. This study will involve multiple blood sample collections for PD. For details on the specific sample collection time points and time window settings, please refer to Section 6.7 **错误!未找到引用源。**, "PD study".

L. Immunogenic blood samples were collected at the following times: D1 before the start of intravenous administration of the investigational drug, D90, and early discontinuation from the visit (if applicable). The sampling time windows shall be consistent with the corresponding visit time windows.

M. If a participant discontinues treatment prematurely during the treatment period, an End of Treatment (EOT) visit shall be completed within 24 hours after the investigator's decision to discontinue treatment. The assessments and procedures at the EOT visit shall be the same as those at the D7 visit. participants who complete the EOT visit are not required to undergo duplicate assessments at D7.

N. For all randomized participants who have received at least one intravenous administration of the investigational product, if the participant withdraws from the study prematurely, an early discontinuation visit shall be conducted within 7 days after the decision to withdraw. Based on the investigator's judgment, assessment results obtained within 3 days prior to the decision to withdraw may be used as early discontinuation visit data, and repeat assessments are not required.

O. Iodine uptake testing is conducted only when the investigator deems it necessary, and the specific testing methods (such as thyroid 131I uptake test, serum iodine and 24-hour urinary iodine) are determined by the investigator based on the actual situation of the center. Iodine uptake test may be omitted if no adverse thyroid events occur in the participant or if the investigator determines that it is unsuitable and/or unnecessary to conduct the test. If our site is unable to perform iodine intake testing, we can accept testing from other hospitals.

P. During the Phase III follow-up period (D29–D76), if a participant's serum albumin level drops to < 30 g/L and is accompanied by grade 2 ascites, or if the investigator determines that re-supplementation of pHSA is necessary, the participant may be allowed to receive retreatment with the investigational drug (rHSA or pHSA) in a blinded manner, based on the randomization assigned during the initial treatment. The dosing regimen is 20 g/day, administered intravenously once

daily for 7 consecutive days, or until serum albumin ≥ 35 g/L (whichever occurs first). The retreatment visit is conducted independently and does not affect the original scheduled follow-up visit. If the retreatment visit overlaps with the original scheduled follow-up visit, the same examination will not be repeated. For details, please refer to the retreatment visit flowchart.

A.

Trial flowchart (Phase III - Retreatment period during follow-up)

**Study day/procedures**	**Retreatment period (D29~D76) [A]**		
	**Day 1 of retreatment (RD1)**	**Day 3 of retreatment (RD3)**	**Day 7 of retreatment (RD7) /Last day of retreatment [H]**
**Window**	/	/	/
Albumin test [B]	X		
Vital signs [C]	X		
Waist circumference and weight measurement [D]	X		
Abdominal ultrasound examination (ascites depth assessment) [F]	X	X	X[E]
Physical examinations		X[E]	X[E]
Hematology		X[E]	X[E]
Blood chemistry		X[E]	X[E]
Coagulation function test		X[E]	X[E]
Urinalysis		X[E]	X[E]
12-lead ECG		X[E]	X[E]
Dosage of investigation product [G]	X		
Recording of concomitant medications	X		
Record of adverse events	X		

Note:

A. If a participant's serum albumin level drops to <30 g/L during the follow-up period, and is accompanied by grade 2 ascites or if the investigator determines that pHSA needs to be supplemented again, the participant may be allowed to receive the investigation product (rHSA or pHSA) again during the follow-up period from D29 to D76, based on the randomization results at the time of the first treatment. Retreatment visits are conducted independently and do not affect the scheduled follow-up visits. If a retreatment visit overlaps with a scheduled follow-up visit, the same tests will not be repeated.

B. During the retreatment period, participants should have their albumin levels checked 1 hour before each administration of the investigation product (including before administration of RD1, which serves as the baseline for albumin changes during the retreatment period) and immediately after the final intravenous

administration. In the event that a study site does not offer a individual albumin test, the investigator may select an alternative laboratory panel that includes albumin assessment. If the serum albumin level is ≥ 35 g/L, the medication will not be continued (it is not necessary to wait for the albumin test results of the day to determine whether the discontinuation criteria have been met before deciding whether to administer the medication that day; once it is known that the participant's serum albumin level has reached the discontinuation criteria, the medication will not be continued). Participants who meet the criteria for early discontinuation of medication and no longer wish to continue taking the medication do not need to undergo albumin testing after discontinuation.

C. On each day of investigational product infusion, vital signs shall be monitored during the infusion period, with assessments conducted once within 2 hours prior to infusion and once at 30 minutes after completion of infusion (+30 minutes). Participants who meet the criteria for early discontinuation of medication and no longer require vital sign monitoring during the retreatment treatment period after discontinuation of medication.

D. Before administering RD1 during the second treatment period, participants should have their waist circumference and weight measured three times, and the average of the three measurements should be recorded as the baseline value for the retreatment. During the retreatment period, starting from RD2, after each intravenous administration, abdominal circumference and weight should be measured 3 times, and the average of the 3 measurements should be recorded. The measurement requirements are the same as during the treatment period. Participants who meet the criteria for early discontinuation of medication and no longer require weight and abdominal circumference measurements during the retreatment period after discontinuation of medication.

E. During the retreatment period, physical examination, hematology, blood chemistry, coagulation function tests, urinalysis, and 12-lead electrocardiogram should be performed at RD3, RD7, or the last day of retreatment. The above safety tests during the RD3 and RD7 visits are only applicable to participants who received the investigation product on that day. Participants who meet the criteria for early discontinuation and do not continue medication will have a retreatment end visit within 24 hours after early discontinuation (as close as possible to the time of completion of the last dose). It is not necessary to continue physical examination, hematology, blood chemistry, coagulation function tests, urinalysis, and 12-lead electrocardiogram during the retreatment period after discontinuation. Investigators determined that the results of examinations conducted within one day prior to the end of treatment could be recorded as the results of follow-up visits after retreatment, and that repeat examinations were not necessary.

F. Abdominal ultrasound examinations will be performed before the first dose of RD1 (baseline), RD3, RD7, or the last day of retreatment. Abdominal ultrasound examinations during RD3 and RD7 visits are only applicable to participants who receive medication on the same day (participants who do not receive medication on the same day do not need to undergo this examination), and must be performed after medication is administered. The time window for abdominal ultrasound examinations during the retreatment period is 1 day. If a retreatment visit overlaps with a follow-up visit, the same tests will not be repeated.

G. Participants will be blinded and re-treated with the investigation product (rHSA or pHSA) according to their randomization at the time of initial treatment, with a dosing regimen of 20 g/day, administered intravenously once daily for 7 days or until serum albumin ≥ 35 g/L (whichever comes first).

H. Participants need to have an end-of-treatment (EOT) visit on the day of the last dose during the retreatment period. If the participant takes the medication as planned for 7 days, the visit should be conducted on the 7th day of the retreatment period. If the participant discontinues the medication early during the retreatment period, the visit should be conducted within 24 hours of the early discontinuation (as close as possible to the time of the last dose completion). No other

visits/procedures are required during the retreatment period.

Clinical Trial Protocol — Main Text

1 STUDY BACKGROUND

1.1 Introduction to Hepatic Cirrhotic Ascites

Hepatic cirrhosis results from various chronic liver injuries leading to structural and functional abnormalities of the liver, primarily characterized by hepatic dysfunction and portal hypertension. Patients with compensated hepatic cirrhosis may remain asymptomatic, whereas those with decompensated cirrhosis can develop serious complications such as ascites, spontaneous bacterial peritonitis (SBP), hepatorenal syndrome (HRS), hemorrhage of digestive tract, hepatic encephalopathy (HE), and sepsis, ultimately leading to death [1,2]. In the Asia-Pacific region, the primary etiology of hepatic cirrhosis is hepatitis B virus (HBV, 51.3%), followed by alcoholic liver disease (ALD, 20.8%), hepatitis C virus (HCV, 15.7%), while metabolic dysfunction-associated fatty liver disease (MAFLD) and other causes account for 12.1% [3]. Currently, China has approximately 70 million chronic HBV carriers [4], about 10 million chronic HCV carriers [5], an estimated 173–338 million people affected by MAFLD, and at least 62 miALD million individuals affected by ALD [6]. Based on these figures, it is estimated that there are approximately 7 million patients with hepatic cirrhosis in China [6].

Ascites is a critical milestone in the natural progression of hepatic cirrhosis and represents the most common and severe complication in patients with decompensated hepatic cirrhosis. The onset of ascites in patients with hepatic cirrhosis can directly or indirectly trigger other severe complications, further reducing patient survival rates. The 1-year, 2-year, and 5-year mortality rates for patients with hepatic cirrhotic ascites are approximately 30%, 50%, and 70%, respectively [7,8].

The formation of ascites in hepatic cirrhosis often results from the interplay of multiple factors. Portal hypertension is the primary cause and initiating factor in ascites formation. Additionally, renin-angiotensin-aldosterone system (RAAS) imbalance and hypoproteinaemia secondary to hepatic cirrhosis also play significant roles in the development of ascites [9]. Based on the volume of ascites, clinical practice classifies ascites into Grade 1 (mild), Grade 2 (moderate),

and Grade 3 (severe). Treatment strategies are selected according to the ascites grade and concomitant disease status. Patients with Grade 1 or 2 ascites, where fluid does not extend beyond the mid-abdomen and ultrasound typically shows a depth of less than 10 cm, generally respond well to sodium restriction or diuretic therapy. These patients may receive either inpatient or outpatient treatment depending on the status of comorbid conditions. Patients with Grade 3 ascites present with significant abdominal distension, with fluid often occupying the entire abdominal cavity and ultrasound showing a depth exceeding 10 cm; such patients require mandatory hospitalization. Currently, commonly used therapeutic approaches for hepatic cirrhotic ascites in clinical practice include etiological treatment, appropriate sodium restriction, diuretic therapy, vasoactive drug therapy, pHSA supplementation following large-volume paracentesis (LVP), and transjugular intrahepatic portosystemic shunt (TIPS) [9]. In recent years, with improved understanding of the pathophysiological mechanisms of decompensated hepatic cirrhosis, pHSA has been utilized not only for the prevention and treatment of post-LVP circulatory dysfunction but also, when administered via long-term or short-term infusion combined with diuretics, for the treatment of hepatic cirrhotic ascites [10].

1.2 Introduction to Plasma-derived Human Serum Albumin (pHSA)

Albumin (ALB) is a moderately sized, non-glycosylated, charged serum protein synthesized primarily in the liver. It is secreted by hepatocytes into the sinusoids and enters the bloodstream, with a half-life of 15–20 days, and is ultimately degraded in muscles, the liver, and kidneys[11]. Under physiological conditions, ALB is the most abundant protein in extracellular fluid, with the plasma level ranging from 35 to 50 g/L, which accounts for approximately 60% of total plasma protein content. ALB exerts multiple biological functions in the human body, primarily including: (1) maintaining plasma colloid osmotic pressure and preserving intravascular–extravascular fluid balance; (2) transporting, binding, and carrying various ions, lipids, and metabolic products; and (3) maintaining capillary permeability, exerting anti-inflammatory and antioxidant effects, and regulating coagulation function [12]. Plasma-derived Human Serum Albumin (pHSA) is the pharmaceutical preparation of ALB and is widely used in clinical practice for the treatment of diseases such as hepatic cirrhotic ascites, hypovolemia, hypoproteinaemia, and acute respiratory distress syndrome.

1.2.1 Application of pHSA in the treatment of hepatic cirrhotic ascites

Hypoproteinaemia is a key pathophysiological feature of hepatic cirrhosis and a significant contributing factor to the development of cirrhotic ascites. Based on albumin's role in maintaining plasma colloid osmotic pressure and its other biological functions, albumin supplementation is considered effective in improving circulatory failure and renal dysfunction, thereby increasing survival rates in patients with hepatic cirrhosis. Currently, multiple clinical guidelines recommend pHSA for the prevention and treatment of post-large-volume paracentesis (LVP) circulatory dysfunction, short-term infusion therapy in hospitalized patients with hepatic cirrhotic ascites, and long-term treatment in discharged patients.

According to the *Rapid Advice Guideline on Plasma-derived Human Serum Albumin in the Treatment of Cirrhosis*[10], pHSA is recommended for the treatment of hepatic cirrhosis and its complications in patients without ascites when plasma ALB level is below 25 g/L, and in patients with ascites when plasma ALB level is below 30 g/L. The dose of pHSA should be individualized based on the patient's actual condition. For patients with hepatic cirrhosis complicated by Grade 2–3 ascites and ALB level below 30 g/L, a daily dose of 10 to 40 g of pHSA combined with diuretics is recommended. In a systematic review [13] including 8 randomized controlled trials (RCTs), the dose range of pHSA was 5–12.5 g/day (5 RCTs) or 20–30 g/day (3 RCTs). The findings demonstrated that, in patients with Grade 2 cirrhotic ascites, infusion of pHSA combined with diuretics, compared with diuretics alone or fluid resuscitation agents alone, significantly improved treatment response rate (RR = 3.43; 95% CI: 1.84–6.38), shortened ascites resolution time (MD = -4.04; 95% CI: -4.58 to -3.50), increased urinary sodium excretion, and reduced serum creatinine level. No statistically significant differences were observed in the incidence of adverse reactions or 24-h urine volume.

As a specialized medical resource, pHSA has not been widely utilized for long-term treatment in patients with hepatic cirrhotic ascites due to resource constraints. However, a recent multi-center, randomized, parallel, unblinded, pragmatic study conducted across 33 hospitals in Italy (the ANSWER study) [14] has provided robust evidence supporting the use of long-term pHSA infusion to improve outcomes in patients with decompensated cirrhotic ascites. For the study, 440 patients with hepatic cirrhosis complicated by mild ascites were enrolled and randomly

assigned to receive either standard medical therapy (SMT, comprising aldosterone receptor antagonists and furosemide) or SMT combined with pHSA. The results showed that in the SMT combined with pHSA group, mean serum albumin levels increased significantly within 1–2 months, rising from a baseline of 31 to approximately 40 g/L. This intervention also significantly improved the 18-month overall survival rate (77% vs. 66%; P = 0.028), corresponding to a 38% reduction in mortality risk (HR = 0.62; 95% CI: 0.40–0.95), without increasing the incidence of adverse events (AEs). A post hoc analysis[15] of this study further revealed that patients with serum albumin levels > 41 g/L at one month of treatment had a survival rate of 93%, whereas those with serum albumin levels ≤ 41 g/L at one month had a survival rate of 70%. These findings suggest that long-term albumin infusion effectively improves serum albumin levels, and that post-treatment serum albumin levels are correlated with prognosis.

In summary, both short-term and long-term administration of pHSA in combination with diuretics can significantly improve therapeutic outcomes in patients with hepatic cirrhotic ascites, with a favorable safety profile.

1.2.2 Market overview of plasma-derived human serum albumin (pHSA)

In addition to its applications in patients with hepatic cirrhotic ascites as described above, pHSA is also used clinically for a variety of acute conditions. Its indications include, but are not limited to, shock caused by hemorrhagic trauma or burns, elevated intracranial pressure due to brain oedema or injury, oedema or ascites secondary to nephropathy, prevention and treatment of hypoproteinaemia, hyperbilirubinaemia neonatal, cardiopulmonary bypass, adjunctive therapy in haemodialysis, and adult respiratory distress syndrome [16].

At present, all injectable-grade pHSA used in clinical practice is derived from human plasma. However, China has long faced a severe shortage of plasma supply. Increasing clinical demand has led to a supply–demand imbalance for pHSA, necessitating heavy reliance on imports. In recent years, both the number of batch releases and the proportion of imported pHSA in China have continued to rise. By 2020, the volume of batch-released pHSA reached 51.526 million vials, of which imports accounted for 60% [17], reflecting substantial clinical demand.

Injectable-grade rHSA can effectively reduce the risk of viral infection while offering ultra-

high purity and consistent quality, thereby ensuring clinical medication safety. Studies have confirmed the similarity between rHSA and pHSA in terms of molecular structure, physicochemical properties, immunology, and physiological functions [18,19]. It is anticipated that the development of rHSA will help bridge the clinical gap in pHSA supply in China, reduce import dependence, and provide additional treatment options for patients requiring pHSA.

1.3 Research and Development Progress of Injection-grade Recombinant Human Serum Albumin

Globally, no injection-grade rHSA biological product has been approved for marketing. In 2008, Mitsubishi Tanabe Pharma Corporation of Japan launched rHSA (Medway) as an injectable product; however, in March 2009, the Ministry of Health, Labour and Welfare issued an order suspending its manufacturing operations [20].

Currently, Tonghua Anrate Biopharmaceutical Co., Ltd. and Wuhan Healthgen Biotechnology Corp. each have one injection-grade rHSA product in the clinical research stage. Tonghua Anrate Biopharmaceutical Co., Ltd. has completed a Phase I clinical trial of its recombinant human albumin (rHA) injection in healthy participants, including both single-ascending-dose and multiple-ascending-dose studies [21]. The study results demonstrated that rHA has a favorable safety profile, and the changes from baseline in serum albumin levels post-administration were comparable between the rHA cohort and the HSA cohort. A multiple-ascending-dose Phase Ib clinical trial in patients with hepatic cirrhotic ascites has also been completed. A multiple-dose Phase Ic clinical trial and a Phase II/III clinical trial in patients with hepatic cirrhotic ascites are currently ongoing. Wuhan Healthgen Biotechnology Corp. has completed a Phase I clinical trial of its plant-derived rHSA therapeutic biological product (OsrHSA) expressed in rice seeds. The dose-escalation study in healthy participants demonstrated that the investigational drug was safe and well tolerated, with no participants testing positive for ADA throughout the trial period [22]. A multi-dose cohort Phase II clinical trial and a Phase III clinical trial in patients with hepatic cirrhosis have also been completed.

1.4 About the Investigational Drug

1.4.1 Introduction to the investigational drug recombinant human serum albumin (rHSA)

Recombinant human serum albumin (rHSA), developed independently by Shenzhen Protgen Co., Ltd. ("Protgen") through an innovative production process, is the participant of patent application PCT/CN2017/083079: "Construction of Engineered Bacteria for High-Efficiency Expression of Recombinant Human Serum Albumin."

rHSA is a single-chain polypeptide of 585 amino acid residues. Its tertiary structure is heart-shaped and contains 35 cysteine residues that form 17 disulfide bonds, with a single free thiol at Cys34. The protein is theoretically non-glycosylated (it contains no consensus glycosylation sites). Its amino-acid sequence and overall structure are identical to natural human serum albumin.

To date, Protgen's rHSA has completed pharmaceutical development and preclinical studies, a Phase Ia (single-ascending-dose) clinical trial in healthy volunteers, and a Phase Ib (multiple-ascending-dose) clinical trial in patients with cirrhotic ascites. Relevant preclinical and clinical study information is summarized below.

1.4.2 Phase Ia (single-ascending-dose) clinical trial in healthy volunteers

Registered as CTR20210413, this single-ascending-dose Phase Ia clinical trial in healthy volunteers evaluated the safety, tolerability, immunogenicity, and pharmacokinetic/pharmacodynamic (PK/PD) profiles of rHSA across multiple dose levels. The study comprised six sequential dose cohorts (2, 5, 10, 20, 30, and 40 g) in a single-ascending-dose design. Eligible participants were enrolled cohort-by-cohort and randomized to receive a single dose of rHSA or placebo (normal saline). Serial blood samples were collected, and safety was monitored over a long-term follow-up period.

The safety results demonstrated the safety profile was favorable across the 2–40 g dose range. The overall incidence of AEs was slightly higher in the investigational drug group than in the placebo group; however, the severity of events were similar between groups, and no clear dose–response relationship in AE incidence was observed. In the safety analysis set, the investigational drug group reported 97 treatment-emergent adverse events (TEAEs) in 33 participants (91.7%), of which 84 TEAEs in 30 participants (83.3%) were judged related to the

study treatment. In the placebo group, 9 participants (75.0%) experienced 19 TEAEs, of which 17 TEAEs in 7 participants (58.3%) were judged related to the study treatment. All TEAEs observed during the trial were mild or moderate in intensity (CTCAE grades 1–2). No participant experienced a grade ≥3 TEAE, and no TEAE led to dose interruption, study-drug discontinuation, or withdrawal from the study. TEAEs judged related to the investigational drug (TRAEs) and occurring in ≥ 10% of participants in the investigational drug group included: Bilirubin conjugated increased (27.8%), neutrophil count decreased (19.4%), white blood cell count increased (13.9%), blood thyroid stimulating hormone increased (13.9%), blood bilirubin increased (3.9%), blood triglycerides increased (13.9%), white blood cell count decreased (11.1%), neutrophil count increased (11.1%), blood calcium decreased (11.1%), and blood uric acid increased (11.1%).

Pharmacokinetic analysis showed that serum albumin level increased after administration of the investigational drug relative to baseline, with C_{max}/C_0 ranging from 1.1444 to 1.3341. Across the 5–40 g dose range, higher doses were associated with larger increases in serum albumin. Within one week after dosing, baseline-corrected serum albumin levels were higher in all investigational-drug dose groups than in the placebo group. The study enrolled healthy participants with relatively high baseline serum albumin levels. Homeostatic physiological processes tended to restore albumin toward the normal range. Consequently, across the 2–40 g dose range, baseline-corrected human serum albumin $AUC_{0\text{–}tlast}$ and C_{max} did not exhibit a dose-dependent relationship.

Pharmacokinetic analysis indicated that administration of the investigational drug was associated with increases in plasma colloid osmotic pressure, ranging from 0.016 to 5.158 mmHg. Over the 0–72 h post-dose period, plasma colloid osmotic pressure generally increased with higher serum albumin levels, and these pressure changes were correlated with PK exposure metrics.

Immunogenicity assessments indicated low immunogenicity of the investigational drug: No participants tested positive for anti-rHSA or anti-HCP antibodies during the study period.

1.4.3 Phase Ib (multiple-ascending-dose) clinical trial in patients with cirrhotic ascites

This study (Registration No. CTR20212509) is a phase Ib, multiple ascending-dose (dose-escalation) clinical trial in patients with cirrhotic ascites, designed to evaluate the safety, tolerability, immunogenicity, PK and PD profiles, and the preliminary efficacy of rHSA across

different dose levels. The study comprised three dose cohorts receiving rHSA at 10 g/day, 20 g/day, or 30 g/day, with dose escalation proceeding sequentially between cohorts. Each dose cohort was planned to enroll 12 participants, allocated 2:1 such that 8 participants would receive the investigational drug and 4 would receive the comparator. The investigational product was administered intravenously once daily for up to 14 days or until serum albumin reached ≥ 35 g/L, whichever occurred first.

Safety results indicated that the investigational drug had a favorable safety profile, and there was no statistically significant difference in the incidence of TEAEs between the investigational drug and comparator groups. In the investigational drug group, 15 participants (62.5%) reported a total of 35 TEAEs considered related to investigational product (TRAEs). TRAEs with an incidence ≥ 5% included: Headache (3 cases [12.5%]), anaemia (3 cases [12.5%]), white blood cell count decreased (2 cases [8.3%]), hyperuricaemia (2 cases [8.3%]), pyrexia (2 cases [8.3%]), and hypotension (2 cases [8.3%]). Only one case of severe TEAE (grade 3 anaemia) related to the investigational drug occurred during the entire trial. No SAE was related to the investigational drug, no treatment-related adverse event (TEAE) led to the discontinuation of the investigational drug, no TEAE led to the withdrawal of participants from the study, and no AE led to the death of participants during the entire trial period.

Efficacy results showed that, at doses of 10–30 g/day, serum albumin levels in both the investigational drug group and the comparator group were higher than baseline immediately after the final intravenous administration. Furthermore, at the same dosage level, there was no significant difference in the proportion and time of participants with serum albumin level ≥ 35 g/L within 14 days after intravenous administration in the investigational drug group and the comparator group. At a dose of 10 g/day, the vast majority of participants (66.7%) received the drug for 14 days. At a dose of 20 g/day, the number of participants in the investigational drug group and the comparator group who achieved a serum albumin level of ≥ 35 g/L were 6 (75%) and 3 (75%) respectively and the median time was 6 days and 8 days respectively. At a dose of 30 g/day, the serum albumin level of all participants in the investigational drug group and the comparator group was ≥35 g/L within 14 days after the first intravenous administration in the PPS, with median times of 6 days and 4 days, respectively. Furthermore, at doses of 10–30 g/day, participants in both the investigational drug group and the comparator group showed significant reductions in abdominal circumference, body weight, and ascites depth compared to

baseline within 14 days after the first intravenous administration.

Pharmacokinetic analysis results showed that, at the same dose level, the changes in serum albumin level over time in participants in the investigational drug group and the comparator group at D1 and D14, as well as the changes in the ratio of serum albumin level to C0 over time, were quite similar between the two groups.

Pharmacokinetic analysis showed that after the participants received the investigation product, the colloid osmotic pressure increased, and at the same dose level, the colloid osmotic pressure changes over time were similar between the investigational drug group and the comparator group.

Immunogenicity analysis showed that all participants included in the analysis had negative rHSA ADA test results, with only 2 participants having positive HCP ADA (positive at baseline), and antibody titers did not change significantly during the study period.

1.5 Risk-Benefit Assessment

1.5.1 Potential risks

The investigational drug was recombinant human serum albumin (rHSA) expressed in yeast, which had essentially the same physicochemical properties and tertiary structure as plasma-derived human serum albumin (pHSA). The results of completed non-clinical studies show that recombinant pHSA has essentially the same PK, PD, and toxicological characteristics as pHSA. The results of the completed Phase Ia clinical trial with a single dose in healthy participants and the Phase Ib clinical trial with multiple doses in patients with cirrhotic ascites showed that the investigational drug rHSA has good safety and similar PK, PD and efficacy characteristics compared with pHSA. Therefore, considering the similarities between the investigational drugs rHSA and pHSA in various aspects, the common adverse reactions of pHSA are of reference value for predicting the safety risks of rHSA. According to reports from domestic and international clinical applications, the adverse reactions of intravenous pHSA administered by the Center for Drug Evaluation (CDE) of the National Medical Products Administration[16] are listed in descending order of importance as follows: anaphylactic shock, hypersensitivity, laryngeal oedema, bronchial asthma, dyspnea, cardiac failure, pulmonary oedema, myocardial infarction, arrhythmia such as atrial fibrillation, tachycardia or bradycardia, hypertension, hypotension, mental agitation, hyperactivity, gastrointestinal bleeding, nausea, vomiting,

hepatic function abnormal, hematuria and oliguria caused by haemolytic reaction, back pain, and in severe cases, kidney damage, acute hemolysis, loss of consciousness, syncope, headache, dizziness, taste disorder, urticaria, angioedema, rash, flushing, itching, pompholyx, fever, chills. In addition, pharmacovigilance data collected from 10 major global pHSA suppliers between 1998 and 2000[22] showed that in 1.62×10^7 clinical applications of pHSA, a total of 198 non-fatal SAEs and 13 fatal SAEs were reported, but no deaths of patients could be clearly attributed to the use of pHSA. The three fatal SAEs that may have been related to pHSA were anaphylactic shock, toxicoderma and dyspnoea, and cardiac arrest and asphyxia.

The Phase Ia clinical trial of the investigational drug in healthy individuals has been completed, with a total of 6 dose groups (2 g, 5 g, 10 g, 20 g, 30 g, and 40 g) for dose escalation. Safety results showed that the investigational drug had good safety profile, and the incidence of AEs was not significantly dose-dependent. In the safety analysis, 97 TEAEs occurred in 33 participants (91.7%) in the investigational drug group, of which 84 TEAEs in 30 participants (83.3%) were determined to be related to the investigation product. All TEAEs observed during the trial were mild or moderate in intensity (CTCAE grades 1–2). No participant experienced a grade ≥ 3 TEAE, and no TEAE led to dose interruption, study-drug discontinuation, or withdrawal from the study. TEAEs judged related to the investigational drug (TRAEs) and occurring in ≥ 10% of participants in the investigational drug group included: Bilirubin conjugated increased (27.8%), neutrophil count decreased (19.4%), white blood cell count increased (13.9%), blood thyroid stimulating hormone increased (13.9%), blood bilirubin increased (3.9%), blood triglycerides increased (13.9%), white blood cell count decreased (11.1%), neutrophil count increased (11.1%), blood calcium decreased (11.1%), and blood uric acid increased (11.1%).

The investigational drug has undergone a phase Ib clinical study in patients with cirrhotic ascites with multiple doses and escalation. Three dose groups (10 g/day, 20 g/day, and 30 g/day) were set up for dose escalation. The safety results showed that the investigational drug has good safety, and there was no significant difference in the incidence of TEAE between the investigational drug group and the comparator group. In the investigational drug group, 15 participants (62.5%) reported 35 TEAEs related to the investigation product. The TRAEs with a high incidence (≥5%) included: headache (3 cases [12.5%]), anaemia (3 cases [12.5%]), white blood cell count decreased (2 cases [8.3%]), hyperuricaemia (2 cases [8.3%]), pyrexia (2 cases

[8.3%]), and hypotension (2 cases [8.3%]). Only one case of severe TEAE (grade 3 anemia) related to the investigational drug occurred.

1.5.2 Potential benefits

Pplasma-derived human serum albumin (pHSA) is widely used in clinical practice. Its indications include, but are not limited to, shock caused by blood loss and trauma, shock caused by burns, brain oedema and increased intracranial pressure caused by injury, oedema or ascites caused by hepatic cirrhosis and nephropathy, prevention and treatment of hypoproteinemia, hyperbilirubinaemia neonatal, cardiopulmonary bypass, adjunctive treatment of haemodialysis and adult respiratory distress syndrome, etc[16]. In patients with cirrhotic ascites, multiple guidelines recommend that pHSA can be used for the prevention and treatment of circulatory dysfunction after large-volume paracentesis (LVP), short-term infusion therapy for hospitalized patients with hepatic cirrhosis or ascites, and long-term treatment for discharged patients [9,10]. Previous studies have suggested[13,14] that short-term (10–40 g/day) and long-term treatment with pHSA combined with diuretics can significantly improve patient outcomes compared with other standard therapies, and there is no significant difference in the incidence of adverse reactions.

However, all injectable pHSA currently used in clinical practice is extracted and prepared from human plasma. For many years, my country has faced a severe shortage of plasma supply, and the increase in clinical usage has led to a shortage of pHSA, requiring a large reliance on imports. The presence of infectious viruses such as HIV and hepatitis not only increases the risks associated with the use of pHSA, but also exacerbates the clinical shortage of pHSA, greatly limiting its clinical use.

The results of the structural elucidation study of the investigational drug recombinant human serum albumin (rHSA) showed that its molecular structure and molecular weight were consistent with those of pHSA. Furthermore, yeast-expressed injectable recombinant pHSA can effectively reduce the risk of viral infection, while the product has extremely high purity and stable quality, ensuring the safety of clinical medication. The Phase Ia clinical trial of the investigational drug in healthy individuals has been completed. Within the dosage range of 2 g to 40 g, serum albumin level increased after administration of the investigational drug compared to before administration, with a larger increase in serum albumin level in the higher dose group. The results of plasma colloid osmotic pressure are correlated with serum albumin level.

Generally, within 0–72 h after drug administration, the higher the serum albumin level, the higher the plasma colloid osmotic pressure. The results of the Phase Ib clinical trial of the investigational drug in patients with cirrhosis and Grade 1–2 ascites showed that the investigational drug had good safety and efficacy at doses of 10 to 30 g/day. The safety results showed that there was no significant difference in the incidence of TEAEs between the investigational drug group (rHSA) and the comparator group (pHSA), and no SAEs related to the investigation product occurred during the entire trial period. Efficacy and PD analysis results showed that the investigational drug could effectively increase serum albumin level and colloid osmotic pressure and reduce abdominal circumference, weight and ascites depth. Furthermore, at equivalent dose levels, no significant differences between the investigational drug group and the comparator group regarding the proportion of participants with serum albumin level ≥ 35 g/L and the duration were observed within 14 days after the first intravenous administration. The trend of change in plasma COP over time was also similar between the two groups. Therefore, in summary, rHSA and pHSA are highly comparable and have good safety and efficacy in patients with cirrhosis and Grade 1–2 ascites.

In summary, the investigational drug rHSA is expected to provide a new treatment option for patients with cirrhotic ascites who require pHSA.

1.5.3 Potential risks and benefit assessment

The investigational drug rHSA has the same molecular weight and molecular structure as pHSA. Clinical study results show that rHSA and pHSA have similar pharmacokinetic, PD and efficacy characteristics. It is expected that rHSA can produce a similar therapeutic effect to pHSA, providing a new treatment option for patients with cirrhotic ascites who need pHSA treatment. The investigational drug rHSA has not yet been approved for marketing by the Chinese drug regulatory authorities, and there are no similar drugs approved for marketing. Therefore, participating in clinical trials may pose some risks to the participants. During the clinical trial, measures such as selecting sites with emergency rescue procedures and equipment, assigning experienced clinicians, and designing reasonable safety checks will be taken to closely monitor the safety of the participants and to make rapid and appropriate interventions in the event of AEs.

In conclusion, this study may provide a safer new treatment option for patients with cirrhotic

ascites who require pHSA therapy.

2 STUDY OBJECTIVES

2.1 Phase II

- **Primary objectives:**

  To evaluate the efficacy of rHSA in increasing serum albumin levels in patients with cirrhotic ascites, and the relationship between dosage and efficacy.

- **Secondary objectives:**
  - To evaluate the safety of Recombinant Human Serum Albumin in patients with cirrhotic ascites.
  - To evaluate the pharmacodynamics of Recombinant Human Serum Albumin in patients with cirrhotic ascites.
  - To evaluate the immunogenicity of rHSA in cirrhotic participants with ascites.
- **Exploratory objective:** To evaluate the effect of rHSA on glycated albumin in patients with cirrhotic ascites.

2.2 Phase III

- **Primary objectives:**

  To evaluate the efficacy of Recombinant Human Serum Albumin in patients with cirrhotic ascites, as indicated by the change from baseline in serum albumin level immediately after completion of the final intravenous administration.

- **Secondary objectives:**
  - The efficacy of Recombinant Human Serum Albumin in patients with cirrhotic ascites based on additional efficacy endpoints.
  - To evaluate the safety of Recombinant Human Serum Albumin in patients with cirrhotic ascites.
  - To evaluate the pharmacodynamics of Recombinant Human Serum Albumin in patients with cirrhotic ascites.
  - To evaluate the immunogenicity of rHSA in patients with cirrhotic ascites.
- **Exploratory objective:** To evaluate the effect of rHSA on glycated albumin and glycated

hemoglobin in patients with cirrhotic ascites.

3 STUDY DESIGN

3.1 Overall Design

This study is a randomized, double-blind, active-controlled, parallel-group phase II/III clinical trial that tests recombinant pHSA (rHSA) in patients with cirrhotic ascites. It is divided into Phase II and Phase III. Phase II will use pHSA as a control and different doses and treatment durations to evaluate the efficacy of rHSA in increasing serum albumin levels in patients with cirrhotic ascites, and to determine the dose-efficacy relationship, thus providing a basis for the dosing regimen and sample size calculation in the Phase III study. Phase III will use the change in serum albumin level from baseline immediately after the final intravenous administration as the primary indicator to evaluate the efficacy of rHSA, and further evaluate its safety, disease progression (PD) characteristics, and immunogenicity.

This Phase II study plans to enroll 92 participants. Using baseline pHSA (< 25 or ⩾ 25 g/L) as the stratification factor, participants will be randomized to the following four groups (23 participants in each group) in a 1:1:1:1 ratio:

- Group 1: Investigational drug 10 g/day, administered continuously for 14 days.
- Group 2: Investigational drug 20 g/day, administered continuously for 7 days.
- Group 3: Comparator drug 10 g/day, administered continuously for 14 days.
- Group 4: Comparator drug 20 g/day, administered continuously for 7 days.

The Phase II phase of this study plans to conduct statistical analysis after all participants in the Phase II study have completed their final visit, and to determine the statistical hypotheses and sample size to be used in Phase III based on the results of Phase II.

Phase III will use the change in serum albumin level from baseline immediately after the final intravenous administration (based on central laboratory tests) as the primary efficacy endpoint to verify the equivalence hypothesis between the investigational drug and the comparator. Phase III will also assess the clinical benefit of albumin supplementation in participants based on ascites improvement. Individual-level ascites improvement is defined as: ascites regression in patients with baseline grade 1 ascites, ascites downgrade in patients with baseline grade 2 ascites, or a decrease in ascites depth of 25% or more from baseline. The ascites improvement

rate at the completion of the final intravenous administration (i.e., the proportion of participants in each group who meet the ascites improvement criteria) will be used as the key secondary efficacy endpoint for non-inferiority assessment, in order to further evaluate the efficacy of the investigational drug (rHSA) in patients with cirrhotic ascites. The results of the Phase II study showed that different serum albumin levels, ascites depth, and whether or not diuretics were used at baseline may affect the changes in serum albumin level and ascites depth at the end of the final intravenous administration compared to baseline. Therefore, in the Phase III study, baseline serum albumin level (< 25 or ⩾ 25 g/L), baseline ascites depth (< 3 cm; 3-6 cm [inclusive of 3, exclusive of 6]; or 6-10 cm [inclusive of 6 and 10]), and whether or not diuretics were used at baseline (yes or no) were used as stratification factors. In Phase III, a total of 388 participants are planned to be enrolled and randomly assigned in a 1:1 ratio to the following two groups (194 participants per group).

- Group A: Investigational drug 20 g/day, administered continuously for 7 days.
- Group B: Comparator drug 20 g/day, administered continuously for 7 days.

The Phase II study includes a screening period, a treatment period, and a follow-up period. Each participant will enter the follow-up period after completing 7 or 14 consecutive days of dosing according to their assigned dosing regimen. All participants will undergo the final visit on D57 ± 5 during the follow-up period.

The Phase III study includes a screening period, a treatment period, and a follow-up period. Participants will enter the follow-up period after completing 7 consecutive days of dosing and undergo the final visit on D90 ± 7 during the follow-up period. If, during the follow-up period from day 29 to day 76, the participant's serum albumin level drops to < 30 g/L and is accompanied by Grade 2 ascites, or if the investigator determines that pHSA needs to be supplemented again, the participant will be treated again. The participant will remain blinded and re-treated with the investigational product (rHSA or pHSA) according to their randomization at the time of initial treatment, with a dosing regimen of 20 g/day, administered intravenously once daily for 7 days or until serum albumin ≥ 35 g/L (whichever comes first). If a participant undergoes retreatment, relevant safety and efficacy tests should be completed during the retreatment period (i.e., the period of re-dosing, not exceeding 7 days), including on

day 1 of retreatment (RD1), RD3 (if dosing is received on the same day), RD7 (if dosing is received on the same day), or the last day of retreatment. Visits during the re-treatment period are conducted independently and do not affect the visits scheduled for the follow-up period. All participants received their last visit on D90 ± 7.

This Phase III study is planned to conduct one unblinded interim analysis when at least 328 participants have completed the D7 visit. The interim analysis will be performed by the Independent Data Monitoring Committee (IDMC), while the entire project team remains blinded. Further details regarding the interim analysis will be provided in the IDMC Charter and Statistical Analysis Plan (SAP).

During the trial, participants will have blood samples collected at specified time points for PD and immunogenicity analysis.

3.2 Selection of Study Population and Rationale

Hepatic cirrhosis is a chronic condition characterized by diffuse hepatic fibrosis and the formation of regenerative nodules resulting from various types of chronic liver injury, primarily manifesting as hepatocellular dysfunction and portal hypertension. Patients with decompensated cirrhosis may experience complications such as ascites, esophageal and gastric variceal bleeding, and hepatic encephalopathy. Ascites is the most common complication in patients with cirrhosis, and it can further lead to serious complications such as spontaneous bacterial peritonitis (SBP) and hepatorenal syndrome (HRS), and even death. The most common mechanism of ascites in cirrhosis is portal hypertension. Other mechanisms include low albumin and decreased plasma colloid osmotic pressure due to liver synthetic dysfunction and neuroendocrine system abnormalities [9].

Albumin (ALB) is synthesized by hepatocytes and accounts for 50% to 60% of the total plasma protein content. It possesses the physical properties of increasing circulating blood volume and maintaining plasma colloid osmotic pressure (COP) as well as a variety of biological functions [23]. Plasma-derived Human Serum Albumin (pHSA), the medicinal preparation of ALB, is widely used in clinical practice. It is utilized not only for the management of patients with various acute and critical illnesses but also frequently for the treatment of ascites, cerebral edema, and hypoalbuminemia.

pHSA is widely used in patients with cirrhotic ascites. Multiple guidelines recommend the use of pHSA for the prevention and treatment of post-paracentesis circulatory dysfunction (PPCD) following large-volume paracentesis (LVP), short-term infusion therapy for hospitalized patients with cirrhotic ascites, and long-term treatment for outpatients. According to the *Rapid Recommendation Guidelines for the Use of Plasma-derived Human Serum Albumin in the Treatment of Cirrhosis* [10], human albumin is recommended for the treatment of patients without ascites with plasma ALB levels below 25 g/L or patients with ascites with plasma ALB levels below 30 g/L. The dosage of human albumin should be individualized based on the patient's actual condition. For patients with severe grade 3 ascites (mostly refractory ascites), paracentesis is a rapid and effective treatment to relieve abdominal distension. Guidelines recommend that continuous large-volume paracentesis (4–6 L/day) combined with pHSA (8 g/1000 mL ascites) is more effective and has fewer complications than diuretics alone.

Results from the structural characterization studies of the investigational drug, recombinant human serum albumin (rHSA), demonstrate that its molecular structure and molecular weight are identical to those of pHSA. Preclinical pharmacodynamic studies suggest that rHSA can significantly reduce ascites volume in animals with cirrhosis; it also shows an effect of increasing plasma colloid osmotic pressure in both model animals and normal animals, and exhibits a certain dose-related effect. Its preclinical pharmacodynamic characteristics are similar to those of commercially available active control pHSA.

Phase Ia studies of the investigational drug in healthy individuals have been completed. This study was conducted as a single ascending dose (SAD) study across 6 dose groups (2 g, 5 g, 10 g, 20 g, 30 g, and 40 g). participants in each group were randomized at a 3:1 ratio to receive the investigational drug (rHSA) or the placebo (sodium chloride injection). The results showed that the test drug at doses of 2–40 g was safe and well-tolerated in healthy participants, with manageable AEs and low immunogenicity. Serum albumin level increased after administration of the test drug compared to pre-administration levels. Within the dosage range of 5–40 g of the test drug, the higher dose group showed a greater increase in serum albumin level, and the test drug also increased plasma colloid osmotic pressure. In general, within 0–72 h after administration, an overall trend was observed that the higher the serum albumin level, the

higher the plasma colloid osmotic pressure, indicating a certain correlation between plasma colloid osmotic pressure and PK results.

A Phase Ib clinical study of the investigational drug in patients with cirrhosis and Grade 1–2 ascites has also been completed. The study involved dose escalation across 3 dose groups (10 g/day, 20 g/day, and 30 g/day), where participants were randomized to receive the investigational drug (rHSA) or the comparator (pHSA). The results showed that the investigational drug had a favorable safety and efficacy profile at doses of 10–30 g/day. Safety results indicated no significant difference in the incidence of TEAEs between the investigational drug group (rHSA) and the comparator group (pHSA), and no investigational product-related SAEs was reported during the entire study period. Efficacy and PD analysis results showed that the test drug effectively increased serum albumin level and colloid osmotic pressure in patients with cirrhotic ascites, and reduced abdominal circumference, body weight, and ascites depth. At equivalent dose levels, there was no significant difference between the test drug group and the comparator group in the proportion and duration of participants with serum albumin level ≥ 35 g/L within 14 days after the first intravenous administration, and the time-course trends of plasma colloid osmotic pressure were also similar between the two groups. Therefore, in summary, rHSA and pHSA are highly comparable, and both demonstrate good safety and efficacy in patients with cirrhosis and Grade 1–2 ascites.

This study will use the change in serum albumin levels from baseline at the end of treatment as the primary efficacy endpoint. To minimize the impact of procedures for refractory ascites, such as paracentesis, on the evaluation of the investigational drug's efficacy, this study requires enrolled patients with cirrhotic ascites to have Grade 1–2 ascites, and serum albumin < 30 g/L at screening.

3.3 Selection and Rationale for the Dosing Regimen

Hypoalbuminemia is an important pathophysiological feature of cirrhosis and a key contributing factor to cirrhotic ascites. According to the approved product information for plasma-derived human serum albumin (pHSA) injection[24], when treating patients with hypoalbuminemia, the dosage should be administered based on the degree of albumin deficiency as needed; however, the daily safe dose should not exceed 2 g of albumin per

kilogram of body weight, meaning that for an adult weighing 60 kg, the daily albumin infusion dose should not exceed 120 g. According to the *Rapid Recommendation Guidelines for the Use of Plasma-derived Human Serum Albumin in the Treatment of Cirrhosis*[10], plasma-derived human serum albumin is recommended for treating patients without ascites with plasma ALB level below 25 g/L, or patients with ascites with plasma ALB level below 30 g/L. The appropriate dose of human serum albumin can be selected within the range of 10–40 g per day based on the patient's actual condition.

A Phase Ib multiple ascending dose (MAD) clinical study of the investigational drug has been completed in patients with cirrhosis and Grade 1–2 ascites. The study included three dose groups (10 g/day, 20 g/day, and 30 g/day), with doses increasing in ascending order. participants were randomly assigned in a 2:1 ratio to receive either the investigational drug (rHSA) or the comparator (pHSA), administered intravenously once daily for 14 consecutive days or until serum albumin reached ≥ 35 g/L (whichever occurred first). The study results demonstrated that the investigational drug (rHSA) exhibited favorable safety and efficacy in patients with cirrhotic ascites and showed good comparability with the comparator (pHSA). Safety results showed that at doses ranging from 10 to 30 g/day, no significant difference in the incidence of TEAEs was observed between the investigational drug group (rHSA) and the comparator group (pHSA). Furthermore, no significant differences in TEAE incidence were observed between the test and comparators across the different dose cohorts. No SAEs related to the investigational product occurred during the entire study period. Efficacy and pharmacodynamic analysis results indicated that at doses of 10 to 30 g/day, there were no significant differences between the investigational drug group and the comparator group regarding the proportion of participants achieving serum albumin level ≥ 35 g/L within 14 days after the first intravenous administration, nor were there significant differences in the median time to achieve this level. Both drugs effectively increased serum albumin levels and reduced abdominal circumference, body weight, and ascites depth. Additionally, the trends in plasma colloid osmotic pressure changes over time were similar between the two groups. At a dose of 10 g/day, tthe investigational drug already demonstrated favorable therapeutic efficacy; at this dose level, most participants (66.7%) received treatment for 14 days. At the completion of the final

administration, the mean increase in serum albumin level from baseline in the investigational drug group was 11.0 g/L. Abdominal circumference, body weight, and ascites depth were all markedly reduced compared to baseline. During the treatment period, the maximum reductions from baseline in abdominal circumference and body weight were 3.46 cm and 2.5 kg, respectively. By day 14 after the first administration, ascites depth in the investigational drug group had decreased by 25.13 mm from baseline. On day 17 after the first administration, colloid osmotic pressure increased by 6.00 mmHg from baseline. At the 20 g/day dose, in the full analysis set, 6 participants (75%) in the investigational drug group achieved a serum albumin level of ≥ 35 g/L, with a median time to achievement of 8 days. At completion of the final administration, the mean increase in serum albumin level from baseline was 9.2 g/L. During the treatment period, the maximum reductions from baseline in abdominal circumference and body weight in the investigational drug group were 6.55 cm and 6 kg, respectively. By day 14 after the first administration, ascites depth had decreased by 27.63 mm from baseline, and colloid osmotic pressure increased by 4.25 mmHg. At a dose of 30 g/day, the median time to achieve a serum albumin level ≥ 35 g/L was 6 days for the investigational drug group and 4 days for the comparator group. Due to premature discontinuation of dosing by participants, the actual total administered dose in the investigational drug group at this dose level was not significantly higher than that in other dose cohorts, resulting in efficacy and PD parameters that were not superior to the other two dose cohorts. Based on the above clinical use of plasma-derived human serum albumin, two dosing regimens were designed for both the investigational drug and comparator groups in the Phase II stage of this study: 10 g/day for 14 consecutive days, and 20 g/day for 7 consecutive days. The study results demonstrated a favorable safety profile for the investigational drug. There were no significant differences in the incidence of TEAEs and TRAEs between the investigational drug and comparator groups, nor was there any significant dose correlation. Compared to the 10 g/day dose group, the 20 g/day dose group showed a more significant change in serum albumin levels from baseline at the completion of the final intravenous dose. Additionally, participants in the 20 g/day cohort achieved serum albumin levels ≥ 35 g/L faster and in a higher proportion. To rapidly correct hypoalbuminemia in patients with cirrhotic ascites and shorten the duration of hospitalization,

the Phase III stage plans to select 20 g/day administered for 7 consecutive days as the dosing regimen.

3.4 Selection and Rationale for Primary and Key Secondary Endpoints in Phase III

To date, no strictly defined registrational confirmatory clinical trials has been conducted for any currently marketed plasma-derived human serum albumin injections; consequently, there are few clinical study results available for reference, and no existing regulations or guidance documents specifically address the selection of primary endpoints for pivotal clinical trials of recombinant albumin-based products. According to the "Rapid Recommendation Guidelines for the Use of Plasma-derived Human Serum Albumin in the Treatment of Cirrhosis" [10] and other relevant diagnosis and treatment guidelines, the direct objective of using plasma-derived human serum albumin in patients with cirrhosis ascites accompanied by hypoproteinemia is to increase serum albumin level. Currently, there is a widely applied and recognized formula for calculating albumin dosage in clinical practice: Required dose (g) = (Target albumin level - Current level) (g/L) * 2 * Plasma volume (L). Through this calculation formula, it is evident that the change in serum albumin level from baseline can most intuitively and reliably evaluate the equivalence between the investigational drug and the comparator. Additionally, other recombinant albumin drugs currently undergoing Phase II or III clinical trials in China also use changes in albumin levels from baseline as the primary efficacy evaluation indicator. Therefore, in Phase III of this study, the change in serum albumin level from baseline, confirmed by immediate albumin testing after the final intravenous administration (based on central laboratory test results), will be used as the primary endpoint to evaluate whether the investigational drug is equivalent to the comparator.

Ascites is one of the common and serious complications in patients with decompensated cirrhosis and is also an important marker of the progression of the natural history of cirrhosis; once ascites appears, the 1-year mortality rate is approximately 15%, and the 5-year mortality rate is 44%–85% [9]; therefore, controlling ascites is the most important treatment goal for this patient population. This study will enroll patients with cirrhosis ascites, and the therapeutic objectives of the investigational product include not only correcting the patients' albumin levels but also improving their clinical symptoms, such as the resolution of ascites. Clinical measurements of abdominal circumference and body weight can be influenced by various factors (such as breathing, eating status, bowel movements, etc.), potentially resulting in

significant discrepancies from actual values, and both can only indirectly reflect the resolution of ascites. The change in ascites depth measured by ultrasound abdomen can most directly and objectively reflect whether patients with cirrhosis ascites have the clinical benefit of ascites resolution; therefore, the Phase III stage of this study will use the ascites improvement rate as a key secondary efficacy endpoints.

Considering that there are currently no clinical guidelines or expert consensus defining a clinically meaningful standard for ascites improvement, and this study enrolls patients with grade 1 or grade 2 ascites where the degree and rate of ascites resolution after treatment may vary among patients with different grades of ascites, this study will therefore use the ascites improvement rate at the completion of the final intravenous administration (improvement is defined as: resolution of ascites for those with baseline grade 1 ascites; downgrading of ascites or a decrease in measured ascites depth of 25% from baseline for those with baseline Grade 2 ascites) as a key secondary endpoint to evaluate patient clinical benefit. During the execution of the study, each research center shall conduct ultrasound abdomen and ascites depth measurements in accordance with unified standard operating procedures to minimize measurement errors as much as possible.

3.5 Provisions for Re-treatment during the Follow-up Period of the Phase III Stage

All participants in the Phase III stage of this study will be followed up until 90 days after the first administration of the investigational product (D90 ± 7 days). Due to the long duration of follow-up period, participants may need to receive additional supplementation of plasma-derived human serum albumin during the follow-up. The regulations regarding the allowable time frame and dosing regimen for re-treatment are as follows:

This study enrolls patients with Grade 1–2 cirrhosis ascites accompanied by hypoalbuminaemia; it is expected that normal or near-normal albumin levels can be maintained for approximately 3 weeks following albumin infusion during the treatment period, meaning the likelihood of requiring re-medication within the follow-up period from D8 to D28 is very low. Therefore, participants should not use the investigational product again prior to D29 during the follow-up period.

To ensure that participants can undergo a sufficiently comprehensive safety evaluation following re-treatment, the use of the investigational product is not permitted during D77–D90 in this study. If the investigator determines that the participant requires further plasma-derived

human serum albumin, the participant is permitted to use commercially available albumin in accordance with clinical routine.

Based on the considerations above, re-use of the investigational product is permitted during D29–D76 of the follow-up period.

During D29–D76 of the follow-up period, if the participant's serum albumin level decreases to <30 g/L accompanied by grade 2 ascites, or if the investigator determines that re-supplementation of albumin is necessary, the participant will receive re-treatment with the corresponding investigational product (rHSA or pHSA) under blinded conditions based on the randomization assignment at the initial treatment; the dosing regimen is 20 g/day, administered intravenously once daily, for 7 consecutive days or until serum albumin ≥ 35 g/L (whichever occurs first). Participants in the re-treatment period are required to undergo corresponding efficacy and safety evaluations; details regarding visits and examinations/procedures during the re-treatment period are provided in Section 6.4.

3.6 PD Study

Albumin has multiple physiological functions, including maintaining plasma colloid osmotic pressure. When pHSA is used in patients with cirrhosis and ascites, it increases plasma colloid osmotic pressure, thereby promoting reabsorption of ascites and preventing further fluid leakage, indirectly achieving the therapeutic goal of controlling ascites progression.

A Phase Ib clinical study of multiple-dose, dose-escalation administration of the investigational drug in patients with cirrhosis with Grade 1–2 ascites demonstrated that at doses of 10 g/day and 20 g/day, colloid osmotic pressure increased from baseline in both the investigational drug group and comparator drug group, and the trends over time were generally similar between the two groups. At the 10 g/day dose, colloid osmotic pressure in the investigational drug group and comparator drug group was 17.78 and 15.20 mmHg, respectively, prior to the start of intravenous administration on Day 14 (D14), with corresponding serum albumin levels of 29.9 and 27.7 g/L. Immediately after completion of intravenous administration on D14, colloid osmotic pressure was 17.16 and 15.30 mmHg in the two groups, respectively, with corresponding serum albumin levels of 31.1 and 28.5 g/L. At the 20 g/day dose, colloid osmotic pressure in the investigational drug group and comparator group was 12.15 and 19.40 mmHg, respectively, prior to the start of intravenous administration on D7, with corresponding serum

albumin levels of 20.7 and 31.6 g/L. Immediately after completion of intravenous administration on D7, colloid osmotic pressure was 13.00 and 18.60 mmHg in the two groups, respectively, with corresponding serum albumin levels of 24.1 and 32.8 g/L. The above study results indicate that colloid osmotic pressure is correlated with serum albumin level, and colloid osmotic pressure increases with rising serum albumin level.

Therefore, to further explore the correlation between colloid osmotic pressure and serum albumin levels, the pharmacodynamic (PD) profile will be evaluated in this study by measuring plasma colloid osmotic pressure and its change from baseline in participants on D1, D7 (applicable only to participants in the 20 g/day group), and D14 (applicable only to participants in the 10 g/day group). The specific time points and windows for PD blood sample collection are described in Section 6.7.

During the trial, the PD blood sample collection time points may be adjusted based on the PD results obtained.

3.7 Immunogenicity Study

Following the administration of investigational drug, participants may develop anti-drug antibodies (ADA), which could affect the safety, efficacy, and pharmacodynamic (PD) profile of the investigational product.

In the Phase II study, blood samples will be collected at the following time points from all participants who have received at least one dose of the investigational product: on D1 (prior to the start of intravenous administration), D8, D15 (applicable only to participants in the 10 g/day group), D29, D57, and at early discontinuation visit (if applicable).

In the Phase III study, blood samples will be collected at the following time points from all participants who have received at least one dose of the investigational product: on D1 (prior to the start of intravenous administration), on D90, and at early discontinuation visit (if applicable). The sampling time windows shall be consistent with the corresponding visit time windows. Immunogenicity will be assessed by evaluating the incidence and titers of anti-rhHSA and anti-host protein (HCP) antibodies (if applicable).

3.8 Interim Analysis

This Phase III study is planned to conduct one unblinded interim analysis when at least 328 participants have completed the D7 visit. The interim analysis will be performed by the Independent Data Monitoring Committee (IDMC), while the entire project team remains blinded. Further details regarding the interim analysis will be provided in the IDMC Charter and Statistical Analysis Plan (SAP).

3.9 Establishment of IDMC

The IDMC will be established during the Phase III stage of this trial to conduct interim analyses and regularly review the progress, safety data, and other important information from the clinical trial. The IDMC shall have a written operating procedure and retain all meeting records, as detailed in the IDMC Charter.

3.10 Trial Suspension or Termination

Trial termination includes both completion of the trial and early termination. If either situation occurs, the trial will be stopped, and all participants must be withdrawn and will no longer receive the investigational product.

Trial completion is defined as the time when the last participant completes the last visit.

Early termination/suspension of the trial refers to the situation where the clinical trial has not been completed for all participants according to the protocol, and the entire trial or a part of it is stopped/suspended midway. During the drug clinical trial, the Sponsor has the right to terminate/suspend the trial early at any time point for reasonable cause. The main purpose is to protect the rights and interests of participants, ensure trial quality, and avoid unnecessary economic losses.

Generally, the trial will not be terminated/suspended arbitrarily. However, the entire trial may be terminated/suspended early if any of the following situations occur:

1) The investigator cannot follow the protocol or GCP, etc.
2) New information is obtained leading to an unfavorable risk-benefit assessment of the investigational drug, or there is sufficient evidence suggesting a lack of efficacy or unacceptable safety.

3) The Sponsor believes it is inappropriate to continue the clinical trial for medical, ethical, or commercial reasons.
4) Participant enrollment is slow, making it impossible to complete the study within an acceptable timeframe.
5) The National Medical Products Administration (NMPA) or the Ethics Committee orders the termination of the trial for some reason.

In case of early termination or suspension of the clinical trial, all parties involved in the study (Sponsor, investigator, Ethics Committee, clinical trial institution, and administrative authorities) should be notified in writing in a timely manner.

4 POPULATION SELECTION

4.1 Inclusion Criteria

Patients meeting all the following criteria may be considered for enrollment:

1) Agreeing to comply with the treatment regimen and visit schedule, willing to be enrolled and sign the Informed Consent Form (ICF).
2) Aged ≥ 18 years and ≤ 70 years on the date of signing the informed consent form, regardless of gender; body mass index (BMI) within the range of 17.0 to 29.0 kg/m^2 (inclusive of boundary values).
3) Patients clinically diagnosed with decompensated cirrhotic ascites, with ascites graded as level 1–2 confirmed by ultrasound abdomen during the screening period, and meeting the criterion of serum albumin (ALB) < 30 g/L.
4) Men and women of childbearing potential (women of childbearing potential include premenopausal women and those within 2 years of menopause) who are willing to use highly effective contraception (condom, contraceptive sponge, gel, film, intrauterine device, oral or injected contraceptives, subcutaneous implants, etc.) from signing the informed consent form through 3 months after the last dose of the investigational drug; women of childbearing potential must have a negative pregnancy test ≤ 7 days before the first dose of the investigational product.

4.2 Exclusion Criteria

Patients meeting any of the following criteria should be excluded:

1) Individuals with a known history of allergy/allergic reaction to yeast or yeast-derived products, or allergy to any component of the investigational formulation; those with an allergic predisposition (multiple drug or food allergies), a history of allergy to biological products, a history of severe systemic allergic reactions due to other causes, and considered by the investigator to be unsuitable for treatment with the investigational drug.
2) Severe digestive system diseases and complications present at screening that, in the investigator's opinion, render the patient unsuitable for participation in this study, including but not limited to malignant ascites, hepatic encephalopathy diagnosed as Grade 3 or 4 according to the West-Haven criteria, portal vein tumor thrombus/thrombosis, post-paracentesis circulatory dysfunction, biliary obstructive disease confirmed by ultrasound or other imaging examinations, hemorrhage of digestive tract with hemostasis achieved for less than 10 days following treatment, failure to achieve effective hemostasis via endoscopic variceal ligation, or conditions assessed by the investigator as posing a high risk of bleeding during the trial (e.g., endoscopy performed within 3 months prior to screening indicating severe esophageal and gastric varices with positive red color sign).
3) Active cardiovascular disease or history thereof at screening, or other conditions judged by the investigator as unsuitable for treatment with human blood albumin, specifically including but not limited to hypertension (systolic blood pressure > 140 mmHg or diastolic blood pressure > 90 mmHg, except where judged by the investigator to be well-controlled and stable), severe anemia, acute heart disorder , severe cardiopulmonary or structural heart disorder, severe arrhythmia, decompensated cardiac failure (with normovolemia or hypervolemia), unstable angina, infarct myocardial within 6 months prior to screening, tachycardia/bradycardia requiring pharmacological treatment, atrioventricular block third degree, etc.
4) Active metabolic system disease or history thereof at screening (excluding patients with stable glycaemia control as judged by the investigator), or concurrent renal impairment considered by the investigator to be unsuitable for treatment with serum albumin.
5) Severe pre-existing disease present at screening that, in the investigator's opinion, render the patient unsuitable for participation in this study, including but not limited to active

malignancies (including hepatocellular carcinoma [HCC]), pulmonary edema, bleeding tendency or active bleeding disorders, uncontrolled infections (including active spontaneous bacterial peritonitis [SBP]), thyroid function abnormal (Grade ≥3 according to the National Cancer Institute Common Terminology Criteria for Adverse Events[NCI CTCAE] version 5.0), and effusion pleural that, as judged by the investigator, may require treatment or is participant to change throughout the trial.

6) The patient had the following laboratory test abnormality:
   - ✓ Liver function: Alanine aminotransferase (ALT) > 5 × ULN (Upper Limit of Normal); Aspartate aminotransferase (AST) > 5 × ULN; Serum total bilirubin (TBIL) > 4 × ULN, or judged by the investigator as unsuitable for participation in the trial.
   - ✓ Renal function: serum creatinine > 3 × ULN, protein urine positive, and judged by the investigator as unsuitable for participation in the trial.
   - ✓ Bone marrow function: absolute neutrophil count (ANC) < 1.0×10^{9}/L; platelet count (PLT) < 20×10^{9}/L; hemoglobin (HGB) < 70 g/L.
   - ✓ Coagulation function: prothrombin time (PT) prolonged > 5 s.
7) Individuals who have received human plasma products (including pHSA) within 7 days prior to the first administration of the investigational product; those with a history of organ transplant; and those who require or plan to undergo interventional invasive examinations or treatments during the study period.
8) Those who have participated in another clinical trial of a new drug or medical device within 30 days prior to screening, or are currently participating in such a trial at the time of screening, and have received the investigational drug/investigational treatment.
9) Individuals with a positive test for human immunodeficiency virus (HIV) antibodies.
10) Pregnant or lactating women.
11) Any other reason judged by the investigator as unsuitable for participation in this study.

4.3 Criteria for Treatment Termination

During the trial, participants may withdraw consent and request to discontinue study treatment. When a participant requests to withdraw from the study treatment, every effort should be made to have the participant complete the early termination visit. Additionally, the investigator may discontinue the investigational product treatment for the participant in the following

circumstances:

- The investigator believes that participants should terminate the study treatment due to safety concerns (such as the occurrence of intolerable AEs).
- During the treatment period, the participant's serum albumin level meets the discontinuation criteria (ALB ≥ 40 g/L), during the re-treatment period within the follow-up phase, the participant's serum albumin level meets the discontinuation criterion (ALB ≥ 35 g/L).
- The participant is pregnant.
- The participant has a serious protocol violation, which, in the opinion of the investigator, has seriously affected the evaluation of the primary endpoint of the study, and the investigator considers that the participant’s treatment should be discontinued.
- Other circumstances where the investigator deems that the participant should terminate treatment.

4.4 Early Withdrawal Criteria

Participants meeting the following criteria need to be withdrawn from the study early:

- Poor compliance, unable to follow the study protocol.
- Being lost for follow-up.
- Participant requests to withdraw from the study and subsequent follow-up.
- Other situations assessed by the investigator as unsuitable for continuing participation in the study.

During the trial, participants may decide to withdraw from the trial treatment and subsequent follow-up at any time for any reason. After the participant confirms withdrawal, the investigator should record the participant's withdrawal event information, including at least the date and reason for withdrawal. Lost to follow-up is defined as the inability to contact the participant after 3 attempts via telephone or other means such as WeChat/email/registered mail/express mail, etc. The investigator should record the specific circumstances of the participant being lost to follow-up, including at least the dates and methods of attempted contact.

4.5 Screening Failure and Rescreening Regulations

Screening failure is defined as a participant who has consented to participate in the clinical trial but was not randomized or did not participate in the study. If a participant withdraws from the study before randomization, it is considered a screening failure. Information to be recorded for screening failure participants includes demographics, reason for screening failure, participant eligibility criteria, and any SAE.

After a patient fails screening for the first time, the research center can provide symptomatic treatment for the cause of screening failure, such as hepatoprotective or diuretic treatment. After receiving corresponding symptomatic treatment, the patient can be followed up according to their condition. During the follow-up period, the patient can participate in screening one more time. If the participant fails screening again, they should not participate in the clinical trial. Corresponding examinations completed during the follow-up period within 1 week prior to the first intravenous administration of the investigational product (including results of examinations from before signing the informed consent form to within 1 week prior to the first administration) do not need to be repeated.

Rescreened participants should be assigned a new screening number.

4.6 Randomization

In the Phase II stage, participants will be stratified during the screening period based on baseline human serum albumin < 25 or ≥ 25 g/L and randomized in a 1:1:1:1 ratio to the following four groups (23 participants per group):

- Group 1: Investigational drug 10 g/day, administered continuously for 14 days.
- Group 2: Investigational drug 20 g/day, administered continuously for 7 days.
- Group 3: Comparator drug 10 g/day, administered continuously for 14 days.
- Group 4: Comparator drug 20 g/day, administered continuously for 7 days.

Based on the Phase II study results and investigator opinions, participants in the Phase III stage will be stratified during the screening period based on baseline serum albumin level (< 25 or ≥ 25 g/L), baseline ascites depth (< 3 cm; 3-6 cm [inclusive of 3, exclusive of 6]; or 6-10 cm [inclusive of 6 and 10]), and baseline diuretic use (yes or no), and randomized in a 1:1 ratio to

the following two groups (194 participants per group):

Group A: Investigational drug 20 g/day, administered continuously for 7 days;

Group B: comparator 20 g/day, administered continuously for 7 days.

At screening, each participant will be identified using a screening number. Screening numbers of screening failure participants will not be reused.

The randomization statistician will use SAS (version 9.4 or above) to generate the randomization schedule. After a participant meets the inclusion criteria and passes screening, the investigator will log in to the Interactive Web Response System (IWRS) to apply for a randomization number according to the order in which the participant enters the trial, and dispense the drug according to the drug number assigned by the central randomization system.

4.7 Participant assignment and numbering

In the Phase II stage, after signing the informed consent form and before relevant study examinations, participants will be assigned a unique screening number in sequential order. The screening number consists of a capital letter "S" + 5 Arabic digits, wherein the first 2 digits are the "Center Number", and the last 3 digits are the "Screening Sequence Number". For example, the screening number for the 2nd screened participant at Center 01 would be "S01002".

In the Phase III stage, after signing the informed consent form and before relevant study examinations, participants will be assigned a unique screening code in sequential order. The screening code consists of capital letters "SC" + 5 Arabic digits, wherein the first 2 digits are the "Center Number", and the last 3 digits are the "Screening Sequence Number". For example, the screening number for the 2nd screened participant at Center 01 would be "SC01002".

At randomization, successfully screened participants will be assigned a randomization number. During the trial, participants will be identified by their randomization number. Once assigned, a participant's randomization number will not be reused. If a participant withdraws from this trial, their screening/randomization number cannot be reused, and they cannot re-enter this trial.

4.8 Blinding and Unblinding Regulations

4.8.1 Blinding execution

According to the standard operating procedures for double-blind clinical trials, appropriate

steps will be taken to ensure treatments are indistinguishable. Neither the participant nor the researchers will know the treatment received by the participant until unblinding after the end of the trial.

During interim analysis, unblinded data will be provided exclusively to an independent unblinded statistical team for data analysis. The unblinded analysis results will then be submitted to the IDMC for review, ensuring that the sponsor, investigators, and relevant research personnel remain blinded to the treatment assignments of trial participants.

4.8.2 Drug coding

This trial uses a double-blind design. The outer packaging of the investigational drug and the comparator should be ensured to be identical (see Section 5.2 "Packaging and labeling" for details). During the trial, neither the participant nor the investigator will know which group's drug is being used.

4.8.3 Routine unblinding

This trial will be unblinded after all participants complete treatment and the database is locked. When data entry is completed, the Case Report Form (CRF) is reviewed without errors, and after a blind review, the data will be locked, and unblinding will be performed by staff from the statistical unit. The blind codes will be stored at the statistical unit to ensure the double-blind status.

4.8.4 Emergency unblinding

In emergency situations where the investigator judges it necessary to know the participant's randomized group assignment for treatment, emergency unblinding can be performed in the IWRS. This must be reported by the principal investigator of the center to the Sponsor and the leading principal investigator. Overdose is not considered a reason for unblinding.

When a SAE is suspected to be related to the investigational product occurs, the investigator at the center should report to the Sponsor and the principal investigator of the leading unit to decide whether to unblind, and report to the relevant regulatory authorities as soon as possible. If unblinding occurs, the date, time, and reason for unblinding must be recorded. After unblinding by the investigator or designated personnel, the unblinded patient will stop

medication and enter the follow-up period.

5 INVESTIGATIONAL DRUGS

5.1 Basic Information of Investigational Product

1) Investigational Drug:

Drug: Recombinant Human Serum Albumin Injection

Specification: 10 g/vial (20%, 50 mL)

Administration method: The investigational drug is not diluted. Calculate the volume according to the dose required by the participant and perform intravenous infusion. The intravenous infusion time for 10 g of investigational drug is 30 min (± 5 min) (excluding flushing time after infusion). The intravenous infusion time for 20 g of investigational drug is 1 h (± 10 min) (excluding flushing time after infusion). Flush the line after intravenous infusion ends; flushing time is 3~6 min. The entire intravenous administration is completed when flushing ends. The specific dosing regimens are shown in the "Drug Management Manual".

Storage conditions: 2–8℃, protect from light

Validity period: 24 months

Provider: Shenzhen Protgen Co., Ltd.

2)Comparator:

Drug: Plasma-derived Human Serum Albumin Injection

Specification: 10 g/vial (20%, 50 mL)

Administration method: The comparator is not diluted. Calculate the volume according to the dose required by the participant and perform intravenous infusion. The intravenous infusion time for 10 g of comparator is 30 min (± 5 min) (excluding flushing time after infusion). The intravenous infusion time for 20 g of comparator is 1 h (± 10 min) (excluding flushing time after infusion). Flush the line after intravenous infusion ends; flushing time is 3–6 min. The entire intravenous administration is completed when flushing ends. The specific dosing regimens are shown in the "Drug Management Manual".

Storage conditions: 2–8℃, protect from light

Validity period: 60 months

Provider: Shenzhen Protgen Co., Ltd.

5.2 Packaging and Labeling

According to GCP and national regulatory requirements, Shenzhen Protgen Co., Ltd. is responsible for the packaging box and labeling of the investigational product.

The packaging of rHSA used in this trial is a 50 mL vial (10 g/vial). Uniform packaging will be used for the investigational product for all participants. The content of the label may include the Sponsor's name, packaging specification, batch number, dosage form, route of administration, quantity, drug number, trial code, usage and dosage, drug storage conditions, expiry date, etc. Specific packaging is participant to the actual product.

5.3 Investigational Product Management

The Sponsor is responsible for providing the investigational product to investigators and clinical trial institutions. The Sponsor shall not provide the investigational product to investigators and clinical trial institutions until the clinical trial has obtained the approval of the Ethics Committee and the permission or filing of the drug regulatory department.

The Sponsor shall provide written instructions for the investigational product to investigators and clinical trial institutions, clarifying the use, storage, and recording of the investigational product. The Sponsor is responsible for establishing procedures for the supply and management of the investigational product, including receipt, storage, dispensing, use, and recovery. Investigational products recovered from participants and unused by researchers shall be returned to the Sponsor or destroyed by the clinical trial institution upon authorization by the Sponsor.

The Sponsor shall ensure that the investigational product is delivered to investigators and clinical trial institutions in a timely manner to ensure timely use by participants, shall maintain records of transportation, receipt, dispensing, recovery, and destruction of the investigational product, shall establish a recovery management system for the investigational product to ensure the recall of defective products, recovery after the end of the trial, and recovery after expiration, and shall establish a destruction system for unused investigational products. There should be written records for the entire management process of the investigational product, with accurate

counting throughout.

The research center must ensure that all investigational products are used only for participants in this clinical trial, and their dosage and usage should follow the trial protocol; if there are issues with the quality or appearance of the investigational product, it should not be dispensed, and the Sponsor should be contacted immediately; unused remaining investigational products and empty packaging of used drugs should be recovered.

The investigator should designate research personnel to be responsible for the receipt, storage, dispensing, use, recovery, inventory, and recording of the investigational product, and records should be completed timely and accurately; a Drug Receipt Form must be signed upon drug receipt in duplicate, with one copy kept by the research center and one by the Sponsor.

The unblinded monitor is responsible for monitoring the receipt, storage, use, inventory, and recovery of remaining investigational products and empty packaging of used drugs. After counting the drugs during visits, situations such as medication errors, overdose, and loss of drugs need to be recorded in the electronic Case Report Form (eCRF) or relevant documents. After counting by the monitor, the remaining investigational products and empty packaging of used drugs will be recovered by the Sponsor, and both parties shall sign the Drug Recovery Form. At the end of the trial, the shipping records of the investigational product must correspond with the quantities used and destroyed/returned. Any discrepancies need to be recorded and the reason for the discrepancy noted.

The Sponsor shall take measures to ensure the stability of the investigational product during the trial. The retention period for retention samples of the investigational product, within the storage period of the investigational product, shall be until the end of clinical trial data analysis or the timeframe required by relevant regulations, whichever is longer.

5.4 Instructions for Use of Investigational Product by Participants

5.4.1 Usage and dosage of investigational product

Check the turbidity and discoloration of the investigational product before use. Do not use the solution if it is turbid or has precipitate.

The investigational product should not be diluted. Once opened, it should be used immediately.

Calculate the required volume according to the dose required by the participant and administer intravenously. The intravenous administration time for 10 g of investigational product is 30 min (± 5 min), and for 20 g of investigational product is 1 h (± 10 min). The specific dosing regimens are shown in the "Drug Management Manual".

Starting from D2, the start time of each administration should not exceed ± 1 hour compared to the start time of the first administration on D1.

Closely monitor changes in the participant's vital signs during infusion, and judge whether to pause or stop infusion based on the actual situation. If the participant experiences mild reactions such as flushing, rash, elevated body temperature, or nausea during administration, the study doctor may pause or stop the infusion based on the participant's condition. If the participant experiences clinical symptoms and manifestations related to cardiovascular overload, such as headache, dyspnea, etc., immediately judge whether infusion needs to be stopped. If the participant is suspected of having an allergy or anaphylactic reaction, such as infusion-related adverse reactions appearing at the infusion site, pruritus, hypotension, etc., the study doctor may pause or stop the infusion based on the situation. If the participant experiences severe anaphylactic reaction or shock, stop infusion immediately and give appropriate treatment. After infusion is complete, closely monitor the participant for any abnormal changes in vital signs or laboratory tests (e.g., electrolyte level, hematocrit/hemoglobin level, etc.).

If infusion is paused or stopped due to an infusion-related adverse reaction during infusion, when performing the next administration, the investigator should comprehensively judge whether to continue administration, pause administration once, or permanently stop medication based on the safety check results before administration.

Study doctors and nurses need to administer the drug according to the designated protocol mentioned above, truthfully record the date, time, and actual dose of each administration, and report to the investigator.

5.4.2 Dietary management for participants during investigational product administration

To prevent significant dietary influence on albumin levels, participants are required to maintain a light diet during the treatment period, with a dietary structure primarily based on

carbohydrates. Prior to each albumin test conducted before intravenous administration, participants must fast for at least 10 h.

5.4.3 Medication errors and management

Medication error is defined as any unintended deviation from the investigational product administration process, including but not limited to missed doses, incorrect dosing (e.g., overdose), incorrect administration timing, wrong medication, or use of expired, contaminated, or deteriorated investigational products. Overdose is defined as the actual administered dose exceeding the planned dose.

If a missed dose occurs or is suspected, no supplementary dose shall be administered. The sponsor and investigator shall determine, based on the actual circumstances, whether to administer the next scheduled dose. The specific reason for non-administration must be documented.

Any medication error shall be recorded as a protocol deviation and documented in detail in the eCRF and other clinical records. In the event of a medication error, regardless of whether it results in an AE and/or SAE, the investigator shall report it within 24 h to the sponsor or the safety department of the authorized Contract Research Organization (CRO). Appropriate measures shall be taken as warranted, such as delaying subsequent dosing or enhancing participant monitoring. If the medication error leads to an AE/SAE, the investigator shall manage the event according to the AE/SAE severity and report it as required.

5.5 Concomitant Medications and Treatments

During the study period, participants may receive standard diuretic therapy selected by the investigator as a concomitant treatment regimen.

In general, participants are not permitted to undergo paracentesis for ascites drainage. However, if the investigator determines that the participant's medical condition is unstable and there is an urgent need for treatment, paracentesis may be performed. For participants who undergo paracentesis during the study period, the decision regarding their inclusion in the Per Protocol Set will be made jointly by the investigator, sponsor, medical monitor, and statistician, taking into consideration the participant's specific circumstances.

During Phase III, the use of other pHSA products (other than the investigational product) is prohibited prior to D14 and not recommended prior to D29. The use of proteolytic enzymes, whole blood, and plasma preparations is prohibited prior to D29. Between D29 and D76, if a participant develops a condition that, in the investigator's judgment, requires re-treatment with pHSA (e.g., serum albumin < 30 g/L accompanied by Grade 2 or higher ascites), the participant may receive re-treatment with either rHSA (investigational drug) or commercially available pHSA (comparator) in accordance with the original randomization assignment. The dosing regimen is 20 g/day, administered once daily by intravenous infusion, for 7 consecutive days or until serum albumin reaches ≥ 35 g/L, whichever occurs first. Between D77 and D90, if a participant develops a condition that, in the investigator's judgment, requires re-treatment with pHSA, the participant may receive commercially available pHSA in accordance with routine clinical practice. The sponsor will not reimburse the cost of commercially available pHSA used during the D77–D90 period.

During the dosing period (Phase II: D1–D14 for the 10 g/day dose group, or D1–D7 for the 20 g/day dose group; Phase III: D1–D7) and during the re-treatment period, the investigational product must not be mixed with any drug other than the solvent, nor administered in combination with vasoconstrictors, injectable solutions containing proteolytic enzymes or alcohol-based solvents, whole blood, or packed red blood cells. During the treatment period (Phase II: D1–D14 for the 10 g/day dose group, or D1–D7 for the 20 g/day dose group; Phase III: D1–D7), the following medications that may interfere with the evaluation of the investigational drug are prohibited: sulfonamide antibiotics, penicillins, benzodiazepines, salicylates, and amino acids (except those used for nutritional supplementation). Fat emulsions are also prohibited. Corticosteroids should generally not be used as part of routine treatment regimens unless deemed essential by the investigator for the management of AEs or required in clinical emergencies to protect participant’s safety. To fully characterize the safety profile of the investigational drug, prophylactic medication for potential AEs associated with the investigational drug (e.g., electrolyte disturbances) is not permitted throughout the entire trial period. However, prophylactic medication for potential AEs related to concomitant medications is allowed.

During the trial, investigators may administer best supportive care or necessary adjunctive therapies based on the patient's condition and clinical needs. Supportive care may include, but is not limited to, diuretics, antiemetics, antihistamines, oxygen therapy, and correction of electrolyte disturbances. For medications not specified as prohibited in the protocol, investigators may use them as deemed appropriate and document accordingly. These may include hepatoprotective drugs, anti-hepatitis B virus agents, anti-hepatitis C virus agents, and others. For participants receiving diuretic therapy at screening, the diuretic regimen during the treatment period (D1–D7) should remain consistent with that at screening (baseline). Every effort should be made to maintain the diuretic regimen unchanged during the follow-up period (D8–D90). However, if the investigator determines that an adjustment to the diuretic regimen is necessary during the follow-up period, such adjustment may be made at the investigator's discretion. Should diuretic-related AEs (e.g., electrolyte disturbances) occur during the study, adjustments to the diuretic regimen may also be made at the investigator's discretion.

The concomitant medications used in this study must be recorded in detail and coded using the WHO Drug Dictionary. The reasons, doses, frequencies, start and end dates, etc. for all concomitant medications and treatments must be fully recorded on the electronic Case Report Form (eCRF).

6 TRIAL PROCEDURES

This trial is divided into the following periods: screening period (D-28 to D-1), treatment period (Phase II 10 g/day group: D1–D14; Phase II 20 g/day group and Phase III: D1–D7), follow-up period (Phase II 10 g/day group: D15–D57; Phase II 20 g/day group: D8–D57; Phase III: D8–D90), and re-treatment period during follow-up (if applicable, Phase III only). The safety assessments and operational procedures required for each period are as follows.

6.1 Screening Period (D-28 to D-1)

Participants and their guardians must sign the ICF in writing on a voluntary basis before participating in the screening. Participants who meet all inclusion criteria and do not meet any of the exclusion criteria can participate in the study.

During the screening period, participants are required to undergo relevant examinations or

observations to confirm the diagnosis, determine disease status, treatment history, etc. The main examinations and procedures include:

- Signing of informed consent form.
- Collection of demographic information (including height, weight, etc.);
- Collection of medical history.
- Collection of treatment history.
- Infectious disease screening.
- Pregnancy testing (for females of childbearing potential only [including premenopausal women and women within 2 years postmenopause], with blood or urine pregnancy test at the investigator's discretion).
- Physical examination.
- Vital signs.
- Hematology.
- Blood chemistry.
- Albumin testing (serum albumin levels will be monitored during the screening period via albumin testing; participants with albumin levels < 30 g/L meet the enrollment criteria);
- Coagulation function testing.
- Glycosylated haemoglobin testing (glycosylated haemoglobin testing during the screening period applies only to Phase II; baseline glycosylated haemoglobin testing for Phase III will be performed prior to the first dose of investigational drug on D1 of the treatment period);
- Urinalysis.
- Stool routine.
- 12-lead ECG.
- Echocardiography.
- Fundi examination.
- Thyroid function test.
- Thyroid ultrasound examination.
- Iodine uptake testing (applicable only to Phase III, and only when deemed necessary by the investigator; the specific testing method shall be determined by the investigator based on the actual conditions of the site);
- Abdominal ultrasound (ascites depth assessment, as baseline data);

- Assessment of inclusion and exclusion criteria (D-28 to D-2) and recheck (D-1);
- Recording of concomitant medications.
- Recording of AEs.

During the screening period, ALB level will be monitored via albumin assessment. A participant is deemed eligible for enrolment if the ALB value is below 30 g/L, and the serum albumin level obtained within 7 days prior to the first intravenous administration during screening will serve as the baseline value.

For abdominal ultrasound, hematology, blood chemistry, coagulation assays, urinalysis, stool routine, 12-lead ECG, pregnancy test, albumin assessment, HbA1c assessment—with the note that HbA1c assessment during screening is applicable solely to Phase II, whereas for Phase III the baseline HbA1c assessment is to be performed on Day 1 of the treatment period prior to the first dose of the investigational product—and echocardiography performed during the screening period, results obtained within 1 week prior to the first intravenous administration of the investigational product are acceptable (including results obtained prior to the signing of informed consent up to 1 week before the first dose); otherwise, these assessments must be repeated within the 1-week window prior to dosing. Other safety assessments (including vital signs, physical examination, infectious disease screening [HBsAg, HBsAb, HBeAg, HBcAb, HBeAb, HBV DNA quantification, HCV antibody, HCV RNA, HIV antibody, and syphilis testing], fundi examination, thyroid function tests, and ultrasound thyroid) may use results obtained within 28 days prior to the first intravenous administration of the investigational product (including results obtained before signing the informed consent form up to 28 days prior to first dosing).

During screening, if a laboratory parameter (excluding blood chemistry albumin assessment or individual albumin test) yields a value outside the exclusion criterion range or exhibits substantial fluctuation, yet the investigator judges the abnormality to be potentially attributable to systematic instrument error or sampling error (e.g., failure to maintain fasting status on the day of assessment), a single repeat test may be permitted upon review and agreement by both the investigator and the sponsor. Should the repeat test result still fall outside the exclusion criterion range, the participant shall be deemed to have failed screening for not meeting the entry criteria. Based on the investigator's judgment, the participant may be permitted to undergo

rescreening after receipt of appropriate symptomatic treatment; refer to Section 4.5, "Screening Failure and Rescreening Provisions," for further details.

On D–1, the inclusion and exclusion criteria shall be reviewed. This review does not require repetition of all screening assessments; rather, the results obtained during the screening period are to be verified against the eligibility criteria. Participants who do not meet all inclusion criteria or who meet any exclusion criteria are ineligible for participation in the study. Once it is confirmed that the participant meets all inclusion criteria and does not meet the exclusion criteria, the participant will be assigned a random number.

6.2 Treatment Period

During the treatment period, on each day of investigational product infusion, the following assessments and procedures are to be performed:

- During the treatment period, participants will receive the investigational product according to their randomization assignment. Participants in the Phase II 10 g/day dose group will receive 10 g/day for 14 consecutive days; participants in the Phase II 20 g/day dose group and all Phase III participants will receive 20 g/day for 7 consecutive days. For detailed instructions on dosage and administration, refer to Section 5.4.1. Throughout the infusion, particular attention shall be paid to the occurrence of infusion-related adverse reactions or clinical signs and symptoms suggestive of cardiovascular overload (e.g., headache, jugular vein distension, dyspnoea). Based on the clinical presentation, a decision to temporarily suspend or permanently discontinue the infusion shall be made at the investigator's discretion (see Section 5.4.1, "Dosage and Administration of Investigational Product," for further details).
- Albumin assessment:
  - During the treatment period, commencing on Day 1, albumin assessments are to be performed within 1 hour prior to each intravenous administration, immediately after completion of the final intravenous administration (+3 minutes; applicable only to Phase II 20 g/day dose group participants and Phase III participants who have completed 7 days of dosing, as well as Phase II 10 g/day dose group participants who have completed 14 days of dosing), and at the End of Treatment (EOT) visit

(applicable only to participants who prematurely discontinue treatment during the treatment period). If a study site does not offer a individual albumin test, the investigator may select an alternative laboratory panel that includes albumin assessment. Additionally, for Phase III participants, samples must be collected prior to dosing on Day 1, immediately after completion of dosing on Day 7 (or at the EOT visit), and shipped to a central laboratory for albumin assessment.

- Eligibility for dosing during the treatment period shall be determined based on albumin (ALB) test results. If ALB < 40 g/L, the participant shall receive the planned intravenous administration; otherwise, dosing shall not be performed. It is not necessary to wait for the albumin test result obtained on the same day to determine whether treatment discontinuation criteria have been met before deciding whether to administer the dose on that day. Once it is known that the participant's serum albumin level has reached the treatment discontinuation criterion, no further dosing shall be administered. If the investigator determines that the albumin test result is abnormal (potentially due to systematic instrument error, sampling error, or transient increases or decreases in albumin levels related to clinical signs or disease conditions), one repeat test is permitted, and the repeat result shall prevail.
- Once the participant enters the treatment period, no further assessment of inclusion/exclusion criteria will be performed; accordingly, if the albumin level is ≥ 30 g/L on Day 1 prior to dosing, this does not constitute screening failure, and the participant shall proceed to dosing in the treatment period.

➢ Vital sign monitoring during dosing: On each day of investigational product infusion, vital signs are to be assessed once within 2 hours prior to the start of infusion and once at 30 minutes (+ 30 minutes) after completion of infusion.

➢ Abdominal circumference and body weight measurements: Prior to the first dose of investigational product on Day 1, three measurements of abdominal circumference and body weight shall be performed (with an interval of more than 1 minute between each measurement), and the mean of the three measurements will be recorded as the baseline value. During the treatment period, after each intravenous administration, three

measurements of abdominal circumference and body weight shall be performed (with an interval of more than 1 minute between each measurement), and the mean of the three measurements will be recorded.

Other assessments and procedures to be conducted at each visit, in addition to the dosing-related activities described above, are delineated as follows.

6.2.1 10 g/day dose group (D1–D14)

6.2.1.1 D1 visit

On Day 1, all participants in the 10 g/day dose group are to undergo blood sample collection for ADA (see Section 6.8, "Immunogenicity Studies"), blood sample collection for PD (see Section 6.7, "Pharmacodynamic Studies"), and glycated albumin assessment prior to the start of the first intravenous administration.

6.2.1.2 D2 visit

If the participant receives intravenous administration on Day 2, the following assessments and procedures are to be performed:

- Physical examination.
- Hematology.
- Blood Chemistry.

6.2.1.3 D3 visit

If the participant meets the discontinuation criteria and does not receive administration on Day 3, an End of Treatment (EOT) visit shall be completed (see Section 6.2.1.7, "EOT Visit"), and the Day 3 visit procedures described below are not required. If the participant receives intravenous administration on Day 3, the following assessments and procedures are to be performed:

- Hematology.
- Blood chemistry.
- Coagulation function testing.
- Urinalysis.
- 12-lead ECG.
- Abdominal Ultrasound (to be performed after completion of administration, within a

1-day time window, for ascites depth assessment).

6.2.1.4 D7 visit

If the participant meets the discontinuation criteria and does not receive administration on Day 7, an EOT visit shall be completed (see Section 6.2.1.7, “EOT Visit”), and the Day 7 visit procedures described below are not required. If the participant receives intravenous administration on Day 7, the following assessments and procedures are to be performed:

- Physical examination.
- Hematology.
- Blood chemistry.
- Coagulation function testing.
- Urinalysis.
- Abdominal Ultrasound (to be performed after completion of administration, within a 1-day time window, for ascites depth assessment).
- 12-lead ECG.

6.2.1.5 D8 visit

Participants are to undergo blood sample collection for ADA at the Day 8 visit.

6.2.1.6 D14 visit

If the participant receives intravenous administration of the investigational product on Day 14, the following assessments and procedures are to be performed at the Day 14 visit (in the event of premature discontinuation, only the EOT visit shall be completed, and the Day 14 visit procedures are not required):

- Blood chemistry.
- Physical examination.
- Waist circumference and weight measurement.
- Abdominal Ultrasound (to be performed after administration, within a 1-day time window, for ascites depth assessment).
- Hematology.
- Coagulation function testing.
- Urinalysis.
- 12-Lead ECG.
- Thyroid function test.

- Thyroid ultrasound examination.
- Hemoglobin A1c (HbA1c) assessment.
- Glycated albumin assessment.
- Blood Sample Collection for PD Studies (see Section 6.7, “Pharmacodynamic Studies”).

Additionally, the occurrence of AEs and information on concomitant medications during the treatment period shall be continuously documented. Unless otherwise specified, no time windows are applied to assessments during the treatment period; any deviation from the protocol-specified timing of assessments shall be recorded. All AEs and concomitant medication information obtained during the treatment period must be recorded in the source documents.

6.2.1.7 EOT visit

If a participant prematurely discontinues treatment during the treatment period, the End of Treatment (EOT) visit shall be completed within 24 hours after the investigator’s decision to discontinue. The EOT visit requires the following assessment and procedure:

- Blood chemistry.
- Physical examination.
- Waist circumference and weight measurement.
- Abdominal Ultrasound (to be performed after administration, within a 1-day time window, for ascites depth assessment).
- Hematology.
- Coagulation function testing.
- Urinalysis.
- 12-Lead ECG.
- Thyroid function test.
- Thyroid ultrasound examination.
- Hemoglobin A1c (HbA1c) assessment.
- Glycated albumin assessment.
- Blood Sample Collection for Pharmacodynamic (PD) Studies (see Section 6.7, “Pharmacodynamic Studies”).

Additionally, the occurrence of AEs and information on concomitant medications during the treatment period shall be continuously documented.

6.2.2 20 g/day dose group (Phase II and Phase III (D1–D7))

6.2.2.1 D1 visit

At D1 visit, for the 20 g/day dose group in Phase II and all participants in Phase III, the following blood samples must be collected prior to the start of the first intravenous administration:ADA blood sample collection (see Section 6.8 "Immunogenicity Study" for details),PD blood samples (see Section 6.7 "Pharmacodynamic Study" for details),glycated hemoglobin and glycated albumin.

6.2.2.2 D2 visit

If the participant receives intravenous administration on Day 2, the following assessments and procedures are to be performed:

- Physical examination.
- Hematology.
- Blood Chemistry.

6.2.2.3 D3 visit

If a participant meets the discontinuation criteria on D3 and therefore does not receive the administration, the End of Treatment (EOT) visit must be completed (refer to Section 6.2.2.5 "EOT Visit"); in this case, the regular D3 visit procedures described below are not required.If the participant receives the intravenous administration on D3, the following examinations and procedures must be performed:

- Hematology.
- Blood chemistry.
- Coagulation function testing.
- Urinalysis.
- Abdominal Ultrasound (to be performed after completion of administration, within a 1-day time window, for ascites depth assessment).
- 12-lead ECG.

6.2.2.4 D7 visit

If the participant receives intravenous administration of the investigational product on D7, the following examinations and procedures should be performed at the D7 visit (if the participant discontinues treatment prematurely, only the EOT visit should be completed, and the D7 visit

is not required):

- Blood chemistry.
- Physical examination.
- Waist circumference and weight measurement.
- Abdominal Ultrasound (to be performed after administration, within a 1-day time window, for ascites depth assessment).
- Hematology.
- Coagulation function testing.
- Urinalysis.
- 12-lead ECG.
- Thyroid function test.
- Thyroid ultrasound examination.
- Iodine uptake testing (applicable only to Phase III, and only when deemed necessary by the investigator; the specific testing method shall be determined by the investigator based on the actual conditions of the site).
- Glycated hemoglobin testing (applicable only to the Phase II stage; not performed at the D7 visit in the Phase III stage).
- Glycated albumin assessment.
- PD blood sample collection (refer to Section 6.7 "Pharmacodynamic Study").

6.2.2.5 EOT visit

If the participant discontinues treatment prematurely during the treatment period, the EOT visit should be completed within 24 hours after the investigator's decision to discontinue treatment. Safety assessment results obtained within 2 days prior to the EOT visit that, in the investigator's judgment, can be recorded as the examination results do not need to be repeated. The following examinations and procedures should be performed during the EOT visit:

- Blood chemistry.
- Physical examination.
- Waist circumference and weight measurement.
- Abdominal ultrasound examination (to be performed after administration, time window ±1 day, for ascites depth assessment).
- Hematology.
- Coagulation function testing.

- Urinalysis.
- 12-Lead ECG.
- Thyroid function test.
- Thyroid ultrasound examination.
- Iodine uptake testing (applicable only to Phase III, and only when deemed necessary by the investigator; the specific testing method shall be determined by the investigator based on the actual conditions of the site).
- Glycated hemoglobin testing (applicable only to the Phase II stage; not performed during the EOT visit in the Phase III stage).
- Glycated albumin assessment.
- PD blood sample collection (refer to Section 6.7 "Pharmacodynamic Study").

Additionally, the occurrence of AEs and information on concomitant medications during the treatment period shall be continuously documented.

6.3 Follow-up Period

Following the completion of the treatment period, participants will enter the follow-up period. The required safety assessments and procedures at each follow-up visit are described below:

6.3.1 10 g/day Dose Group (Phase II) (D15–D57)

6.3.1.1 D15 visit

At the D15 visit, participants are required to undergo ADA blood sample collection.

6.3.1.2 D29 visit (± 3 days)

At the D29 visit, participants are required to undergo the following examinations and procedures:

- Vital signs.
- Physical examination.
- Waist circumference and weight measurement.
- Abdominal ultrasound examination (ascites depth assessment).
- Hematology.
- Blood chemistry.
- Coagulation function testing.
- Urinalysis.

- 12-Lead ECG.
- Thyroid function test.
- Thyroid ultrasound examination.
- ADA blood sample collection (See Section 6.8 "Immunogenicity Study" for details).

6.3.1.3 D57 visit (± 5 days)

At the D57 visit, participants are required to undergo the final visit examinations and procedures, including:

- Pregnancy test (applicable only to women of childbearing potential, with serum or urine pregnancy test at the discretion of the investigator);
- Vital signs.
- Physical examination.
- Waist circumference and weight measurement.
- Abdominal ultrasound examination (for ascites depth assessment).
- Hematology.
- Blood chemistry.
- Coagulation function testing.
- Urinalysis.
- 12-Lead ECG.
- Fundi examination.
- Thyroid function test.
- Thyroid ultrasound examination.
- ADA blood sample collection (see Section 6.8 "Immunogenicity Study" for details);

6.3.2 20 g/day Dose Group (Phase II) (D8–D57)

6.3.2.1 D8 visit

At the D8 visit, participants in the Phase II stage are required to undergo ADA blood sample collection. ADA blood sample collection is not performed at the D8 visit in the Phase III stage (see Section 6.8 "Immunogenicity Study" for details).

6.3.2.2 D14 visit

At the D14 visit, participants are required to undergo the following examinations and procedures:

- Vital signs.

- Physical examination.
- Hematology.
- Blood Chemistry.

6.3.2.3 D29 visit (± 3 days)

At the D29 visit, participants are required to undergo the following examinations and procedures:

- Vital signs.
- Physical examination.
- Waist circumference and weight measurement.
- Abdominal ultrasound examination (for ascites depth assessment) ;
- Hematology.
- Blood chemistry.
- Coagulation function testing.
- Urinalysis.
- 12-Lead ECG.
- Thyroid function test.
- Thyroid ultrasound examination.
- Hemoglobin A1c (HbA1c) assessment.
- ADA blood sample collection (applicable only to Phase II, see Section 6.8 "Immunogenicity Study" for details);

6.3.2.4 D57 visit (± 5 days)

At the D57 visit, participants are required to undergo the final visit examinations and procedures, including:

- Pregnancy test (applicable only to women of childbearing potential, with serum or urine pregnancy test at the discretion of the investigator).
- Vital signs.
- Physical examination.
- Waist circumference and weight measurement.
- Abdominal ultrasound examination (for ascites depth assessment);
- Hematology.
- Blood chemistry.

- Coagulation function testing.
- Urinalysis.
- 12-Lead ECG.
- Fundi examination.
- Thyroid function test.
- Thyroid ultrasound examination.
- Hemoglobin A1c (HbA1c) assessment.
- ADA blood sample collection (see Section 6.8 "Immunogenicity Study" for details);

6.3.3 Groups A and B (Phase III) (D8–D90)

6.3.3.1 D14 visit

At the D14 visit, participants are required to undergo the following examinations and procedures:

- Vital signs.
- Physical examination.
- Hematology.
- Blood Chemistry.

6.3.3.2 D29 visit (± 3 days)

At the D29 visit, participants are required to undergo the following examinations and procedures:

- Vital signs.
- Physical examination.
- Waist circumference and weight measurement.
- Abdominal ultrasound examination (for ascites depth assessment).
- Hematology.
- Blood chemistry.
- Coagulation function testing.
- Urinalysis.
- 12-Lead ECG.
- Thyroid function test.
- Thyroid ultrasound examination.
- Iodine uptake assessment (to be performed only when considered necessary by the

investigator; the specific assessment method will be determined by the investigator based on the actual conditions of the study site).

- Glycated hemoglobin test.

6.3.3.3 D57 visit (± 5 days)

At the D57 visit, participants are required to undergo the following examinations and procedures:

- Vital signs.
- Physical examination.
- Waist circumference and weight measurement.
- Abdominal ultrasound examination (for ascites depth assessment).
- Hematology.
- Blood chemistry.
- Coagulation function testing.
- Urinalysis.
- 12-Lead ECG.
- Thyroid function test.
- Thyroid ultrasound examination.
- Iodine uptake assessment (to be performed only when considered necessary by the investigator; the specific assessment method will be determined by the investigator based on the actual conditions of the study site).

6.3.3.4 D90 visit (± 7 days)

At the D90 visit, participants are required to undergo the final visit examinations and procedures, including:

- Pregnancy test (applicable only to women of childbearing potential, with serum or urine pregnancy test at the discretion of the investigator);
- Vital signs.
- Physical examination.
- Waist circumference and weight measurement.
- Abdominal ultrasound examination (for ascites depth assessment).
- Hematology.
- Blood chemistry.
- Coagulation function testing.

- Urinalysis.
- 12-Lead ECG.
- Fundi examination.
- Thyroid function test.
- Thyroid ultrasound examination.
- Iodine uptake assessment (to be performed only when considered necessary by the investigator; the specific assessment method will be determined by the investigator based on the actual conditions of the study site).
- Hemoglobin A1c (HbA1c) assessment.
- ADA blood sample collection (see Section 6.8 "Immunogenicity Study" for details).

In addition, AEs and concomitant medications should be continuously recorded throughout the follow-up period. Any information on AEs and concomitant medications obtained during the follow-up period must be documented in the electronic Case Report Form (eCRF). For patients who are ADA-positive at the end of the follow-up period, the sponsor and investigator will determine whether follow-up should continue until the ADA-positive result reverts to negative. Adverse events that persist at the end of the follow-up period must be followed until their outcome.

6.4 Retreatment Period during Follow-up (Applicable Only to Phase III)

During the follow-up period of Phase III, if a participant's serum albumin level drops to < 30 g/L accompanied by Grade 2 ascites, or if the investigator determines that repletion with plasma-derived human serum albumin is necessary, retreatment is permitted between D29 and D76 of the follow-up period. Participants will receive retreatment with the investigational product (rHSA or pHSA) under blinded conditions according to their randomized treatment assignment from the initial treatment period. The investigational product will be administered once daily by intravenous infusion for 7 consecutive days or until serum albumin reaches ≥ 35 g/L, whichever occurs first.

Retreatment visits are conducted independently and do not affect the scheduled follow-up visits. If a participant receives retreatment during the follow-up period, they must undergo all required examinations and procedures specified for the retreatment period, while continuing to complete other scheduled follow-up visits and procedures as originally planned. In the event of

overlapping visits or duplicative examinations between the retreatment period and the originally scheduled follow-up visits, the assessments need only be performed once; repeated visits or examinations are not required.

- On each day of investigational product infusion during the retreatment period, participants are required to undergo the following examinations and procedures:
  - Albumin assessment:
    - ✓ During the retreatment period, starting from RD1 (first day of retreatment), albumin test must be performed within 1 h prior to each intravenous administration (including prior to dosing on RD1, which serves as the baseline for albumin changes during the retreatment period), and immediately after completion of the final intravenous administration.(if a single albumin test is not available at the study site, the investigator may select another laboratory test that includes albumin as a component).
    - ✓ Albumin test results during the retreatment period will be used to determine whether the participant meets the dosing criteria: If ALB < 35 g/L, the participant will receive intravenous administration; otherwise, dosing will not be administered. It is not necessary to wait for the albumin test result obtained on the same day to determine whether treatment discontinuation criteria have been met before deciding whether to administer the dose on that day. Once it is known that the participant's serum albumin level has reached the treatment discontinuation criterion, no further dosing shall be administered. If the investigator determines that the albumin test result is abnormal (potentially due to systematic instrument error, sampling error, or transient increases or decreases in albumin levels related to clinical signs or disease conditions), one repeat test is permitted, and the repeat result shall prevail.
  - Vital signs monitoring: On each day of investigational product infusion during the retreatment period, vital signs should be monitored within 2 hours prior to infusion and 30 min (+ 30 min) after completion of infusion.
  - Abdominal circumference and body weight measurements: Prior to the first

administration of the investigational product on RD1 during the retreatment period, abdominal circumference and body weight should be measured three times, and the average of the three measurements will be recorded as the baseline value for the retreatment period. During the retreatment period, following each intravenous administration, abdominal circumference and body weight should be measured three times, and the average of the three measurements will be recorded.

Other assessments and procedures to be conducted at each visit, in addition to the dosing-related activities described above, are delineated as follows.

6.4.1 RD1 visit

- Baseline abdominal ultrasound examination (results obtained within 3 days prior to the first administration on RD1 of the retreatment period are acceptable).

6.4.2 RD3 visit

If the participant does not receive intravenous administration on RD3, the examinations and procedures for the RD3 visit are not required. If the participant receives intravenous administration on RD3, the following examinations and procedures should be performed:

- Physical examination.
- Hematology.
- Blood chemistry.
- Coagulation function testing.
- Urinalysis.
- 12-lead ECG.
- Abdominal Ultrasound (to be performed after completion of administration, within a 1-day time window, for ascites depth assessment).

6.4.3 RD7 Visit

If the participant does not receive intravenous administration on RD7, the examinations and procedures for the RD7 visit are not required. If the participant receives intravenous administration on RD7, the following examinations and procedures should be performed:

- Physical examination.

- Hematology.
- Blood chemistry.
- Coagulation function testing.
- Urinalysis.
- 12-lead ECG.
- Abdominal ultrasound examination (to be performed after administration, within a 1-day time window).

6.4.4 Re-treatment EOT visit

During the re-treatment period, participants need to have an end-of-treatment (EOT) visit on the day of the last dose. If the participant has taken the medication as planned for 7 days, the visit will be conducted on RD7 of the re-treatment period. If the participant discontinues the medication early during the re-treatment period, the visit will be conducted within 24 hours after the early discontinuation (as close as possible to the time of completion of the last administration). No other visits or procedures are required during the re-treatment period afterward. Physical examination, hematology, blood chemistry, coagulation function test, urinalysis, and 12-lead ECG results obtained within 1 day prior to treatment discontinuation, as judged by the investigator, may be documented as the EOT visit assessments for re-treatment without repeating the examinations. The specific examination and procedures to be performed during this visit are as follows:

- Physical examination.
- Hematology.
- Blood chemistry.
- Coagulation function testing.
- Urinalysis.
- Waist circumference and weight measurement.
- 12-lead ECG.
- Abdominal ultrasound examination (performed after drug administration, within a time window of ±1 day).

6.5 Unscheduled Visits

In consideration of participants' safety, additional visits or examinations may be requested by the investigator. Results of unscheduled visits or examinations should be documented in the electronic case report forms (eCRFs).

6.6 Early Discontinuation Visit

For all randomized participants who received at least one intravenous dose of the investigational product, the investigator may comprehensively consider whether the participant should continue to participate in all or part of the visits specified in the protocol based on the completeness of the participant's medication administration and the participant's willingness. If a participant withdraws from the study early during the treatment period, the EOT visit shall be completed first (safety assessments performed within 2 days before the EOT visit, as judged by the investigator, may be documented as the examinations for the early termination visit without repetition). The early discontinuation visit shall be completed within 7 days after the EOT visit. If a participant withdraws from the study early during the follow-up period, the early termination visit shall be performed within 7 days after the decision to withdraw early. The examinations for the early termination visit are the same as those for the Day 90 visit (examinations performed within 3 days before the decision to withdraw, as judged by the investigator, may be documented as the assessments for the early termination visit without repetition). The specific relevant examinations and procedures include:

- Vital signs.
- Physical examination.
- Waist circumference and weight measurement.
- Abdominal ultrasound examination (for ascites depth assessment).
- Hematology.
- Blood chemistry.
- Coagulation function testing.
- Urinalysis.
- 12-Lead ECG.
- Fundi examination.
- Thyroid function test.

- Thyroid ultrasound examination.
- Iodine uptake testing (applicable only to Phase III, and only when deemed necessary by the investigator; the specific testing method shall be determined by the investigator based on the actual conditions of the site).
- Hemoglobin A1c (HbA1c) assessment.
- Pregnancy test (applicable only to women of childbearing potential, with serum or urine pregnancy test at the discretion of the investigator).
- ADA blood sample collection.
- Recording of AEs.
- Concomitant medication log.

6.7 PD Study

This trial evaluates the drug's PD characteristics by measuring plasma COP after administration. For participants who have received at least one intravenous dose, PD blood samples should be collected at the following time points, or at the early discontiunation visit during the treatment period (if applicable). (No examination is required for early discontinuation during the follow-up period):

- **Phase II 10 g/day group:**
  - Within 1 hour prior to intravenous administration on D1.
  - Within 1 hour prior to intravenous administration on D14, and immediately after the completion of intravenous administration (+ 3 min).
  - Early discontinuation visit (within 24 hours of the decision to withdraw, as close as possible to the time of completion of the last administration).
- **Phase II 20 g/day group and phase III:**
  - Within 1 hour prior to intravenous administration on D1.
  - Within 1 hour prior to intravenous administration on D7, and immediately after the completion of intravenous administration (+ 3 min).
  - Early discontinuation visit (within 24 hours of the decision to withdraw, as close as possible to the time of completion of the last administration).

PD blood samples for this test must be stored in a freezer at -60 to -90℃ (detailed requirements are participant to the laboratory manual), and the samples will be transported to the central laboratory for unified testing. For details on the procedures for sample collection, processing,

preservation, and transportation, please refer to the laboratory standard operating procedures or relevant clinical operation manuals.

6.8 Immunogenicity Study

Blood samples of ADA were collected from all participants who received at least one intravenous administration in this trial. The specific time points and time windows for ADA blood sample collection are as follows:

- **Phase II, 10 g/day group:** Prior to the start of investigational product IV administration on Day 1, and on Days 8, 15, 29, 57, and at the early discontinuation visit (if applicable).
- **Phase II, 20 g/day group:** Prior to the start of investigational product IV administration on Day 1, and on Days 8, 29, 57, and at the early discontinuation visit (if applicable).
- **Phase III:** Prior to the start of investigational product IV administration on Day 1, and on Day 90, and at the early discontinuation visit (if applicable).

The sampling time windows shall be consistent with the corresponding visit time windows. Immunogenicity was assessed by evaluating the incidence and titer of anti-rHSA and anti-host protein (HCP) ADAs, if applicable.

The ADA blood samples for this study need to be stored in a refrigerator at -60 ~ -90℃ (for detailed requirements, refer to the laboratory manual), and the samples will be transported to the central laboratory for unified testing. For details on the procedures for sample collection, processing, preservation, transportation, etc., please refer to the laboratory standard operating procedures or relevant clinical operation manuals.

7 STUDY ASSESSMENTS

7.1 Efficacy Assessments

7.1.1 Waist circumference and weight measurement

Abdominal circumference and body weight will be measured before the start of the first administration of the investigational product on D1, after the completion of each administration

of the investigational product during the treatment period, at the EOT visit, at the follow-up visits of D29 (± 3 days), D57 (± 5 days), D90 (± 7 days, applicable only to Phase III), and at the early discontinuation visit (if any). In Phase III, if a participant receives re-treatment during the follow-up period, abdominal circumference and weight measurements are required before the first administration of the investigational product on RD1, after each administration of the investigational product during re-treatment, and at the re-treatment EOT visit.

The instructions for abdominal circumference measurement and the standards for physical measurement in this study are detailed in Appendix I and Appendix II.

7.1.2 Abdominal ultrasound assessment

Abdominal ultrasound examinations will be performed during the screening period, treatment period visits on Day 3 (ultrasound time window: 1 day), Day 7 (ultrasound time window: 1 day), Day 14 (only applicable to participants in the 10 g/day group; ultrasound time window: 1 day), EOT visit, follow-up period visits on Day 29 (± 3 days), Day 57 (± 5 days), Day 90 (± 7 days, only applicable to Phase III), and the early discontinuation visit (if any). In Phase III, if a participant receives re-treatment during the follow-up period, an abdominal ultrasound examination is required before the first administration of the investigational product on RD1, during re-treatment on RD3 (if administered on the same day), RD7 (if administered on the same day), or during the re-treatment EOT visit (+ 1 day).

The operation and grading standards for measuring peritoneal effusion by abdominal ultrasound in this study are detailed in the "SOP for Ascites Examination under Abdominal Ultrasound".

7.1.3 Albumin test

During the screening period, albumin (ALB) levels will be monitored. If ALB level is below 30 g/L, the participant will meet the enrollment requirements.

During the treatment period, starting from D1, participants are required to undergo albumin testing within 1 hour before each intravenous administration, immediately after completion of the final intravenous administration (+ 3 min; only for participants in the Phase II 20 g/day group, participants in Phase III who completed 7 days of administration, and participants in the Phase II 10 g/day group who completed 14 days of administration), and at the EOT visit (only for participants who discontinued treatment prematurely during the treatment period). If the study site does not offer standalone albumin testing, the investigator may select other laboratory

tests that include albumin as a parameter.

Eligibility for dosing during the treatment period shall be determined based on ALB test results. If ALB < 40 g/L, the participant shall receive the planned intravenous administration; otherwise, dosing shall not be performed. It is not necessary to wait for the albumin test result obtained on the same day to determine whether treatment discontinuation criteria have been met before deciding whether to administer the dose on that day. Once it is known that the participant's serum albumin level has reached the treatment discontinuation criterion, no further dosing shall be administered. If the investigator determines that the ALB test result is abnormal (potentially due to systematic instrument error, sampling error, or transient increases or decreases in albumin levels related to clinical signs or disease conditions), one repeat test is permitted, and the repeat result shall prevail.

During the Phase III follow-up period, changes in the participant's serum albumin level will be monitored. If the participant's serum albumin level drops to < 30 g/L and is accompanied by Grade 2 ascites, or if the investigator determines that plasma-derived human serum albumin needs to be supplemented again, re-treatment will be administered. During the retreatment period, participants should have their albumin levels checked 1 hour before each administration of the investigation product (including before administration of RD1, which serves as the baseline for albumin changes during the retreatment period) and immediately after the final intravenous administration. If a study site does not offer individual albumin testing, the investigator may select an alternative laboratory panel that includes albumin assessment. If the serum albumin level is ≥ 35 g/L, the medication will not be continued (it is not necessary to wait for the albumin test results of the day to determine whether the discontinuation criteria have been met before deciding whether to administer the medication that day; once it is known that the participant's serum albumin level has reached the discontinuation criteria, the medication will not be continued). Participants who meet the criteria for early discontinuation of medication and no longer wish to continue taking the medication do not need to undergo albumin testing after discontinuation.

7.1.4 Efficacy endpoints

The baseline values are the waist circumference, body weight measurements before the first administration of the investigational product on D1, and the ascites depth confirmed by abdominal ultrasound during the screening period. The preliminary evaluation of the

effectiveness of the investigational product in treating ascites will be conducted by analyzing the changes in indicators such as body weight, waist circumference, and ascites depth relative to baseline values.

Phase II efficacy endpoints include:

- **Primary efficacy endpoints:**

  The change in serum albumin level from baseline, as confirmed by albumin test immediately after the completion of the final intravenous administration.

- **Secondary efficacy endpoints:**

  1) Change from baseline in ascites depth after administration.
  2) Proportion of participants with serum albumin level ≥ 35 g/L, confirmed by albumin test at the completion of the final intravenous administration.
  3) Time to achieve serum albumin level ≥ 35 g/L.
  4) Change from baseline in abdominal circumference after administration.
  5) Change from baseline in body weight after administration.

- **Exploratory endpoints:** Change from baseline in glycated albumin levels after administration.

Phase III efficacy evaluation indicators include:

- **Primary efficacy endpoint:**

  The change in serum albumin level from baseline, confirmed by an albumin test immediately after the final intravenous administration (based on the results of central laboratory tests).

- **Key secondary efficacy endpoints:**

  The improvement rate of ascites at the completion of the final intravenous administration. The definition of ascites improvement is as follows: for those with baseline grade 1 ascites, the ascites resolves; for those with baseline Grade 2 ascites, the ascites grade is reduced, or the measured depth of ascites decreases by 25% compared with the baseline.

- **Secondary efficacy endpoints:**

  1) Change from baseline in serum albumin level, as confirmed by local laboratory test immediately after the completion of the final intravenous administration.
  2) Change from baseline in ascites depth at the completion of the final intravenous administration.

3) Proportion of participants with serum albumin level ≥ 35 g/L, confirmed by albumin test at the completion of the final intravenous administration.
4) Time to achieve serum albumin level ≥ 35 g/L.
5) Change from baseline in abdominal circumference at the completion of the final intravenous administration.
6) Change from baseline in body weight at the completion of the final intravenous administration.

For patients who receive the investigational product treatment again within the D29–D76 period, the efficacy evaluation indicators also include:

1) Change from baseline before retreatment in serum albumin level confirmed by albumin test immediately after completion of the final intravenous administration during the retreatment period.
2) Ascites improvement rate at the completion of the final intravenous administration during the retreatment period, with ascites improvement during the retreatment period defined as: resolution of ascites in participants with Grade 1 ascites at baseline of the retreatment period, or downgrading of ascites in participants with Grade 2 ascites at baseline of the retreatment period, or a ≥ 25% reduction in ascites depth measurement from baseline.
3) Change from baseline before retreatment in ascites depth at the completion of the final intravenous administration during the retreatment period.
4) Proportion of participants with serum albumin level ≥ 35 g/L as confirmed by albumin test during the retreatment period.
5) Time to serum albumin level ≥ 35 g/L confirmed by albumin test during the retreatment period.
6) Change from baseline before retreatment in abdominal circumference at the completion of the final intravenous administration during the retreatment period.
7) Change from baseline before retreatment in body weight at the completion of the final intravenous administration during the retreatment period.

- **Exploratory endpoints:** Change from baseline in glycated albumin levels and glycated hemoglobin levels after administration.

7.2 Safety Evaluation

The safety evaluation indicators of this trial include the type, incidence, and severity of AEs. AEs will be graded using the National Cancer Institute Common Terminology Criteria for Adverse Events (NCI CTCAE) Version 5.0. The relationship of AEs to the investigational product will be assessed by the investigator in accordance with the protocol-specified attribution assessment criteria.

During the trial, participants are required to undergo safety assessments at the scheduled time points, including laboratory tests (hematology, urinalysis, biochemistry, coagulation function tests, etc.), vital signs, 12-lead electrocardiogram (ECG), physical examination, fundoscopy, thyroid function tests, thyroid ultrasound, and iodine uptake test (only applicable to Phase III). AEs will be documented with their clinical characteristics, severity, onset time, end time, treatment measures, and outcome, and their correlation with the investigational product will be determined.

Specific safety endpoints include, but are not limited to, the following:

1) Any reported and all directly observed AEs.
2) Any abnormal changes in vital signs and physical examinations.
3) Treatment-emergent clinically significant abnormal laboratory test results, clinically significant abnormal hematology test results, etc.

The schedule and specific indicators for the clinical examinations related to safety evaluation in this trial are as follows (the specific indicators shall be participant to those commonly used in the investigator site's examination reports). For details, refer to the clinical trial flow chart.

7.2.1 Vital signs

Vital signs mainly include blood pressure, pulse, respiratory rate, and body temperature.

Vital signs will be assessed during the screening period, treatment period, and re-treatment period (if applicable), before and after each administration of the investigational product, at D14 (D14 is applicable to participants in the 10 g/day group during the treatment period and participants in the 20 g/day group during the follow-up period), D29 (± 3 days), D57 (± 5 days), D90 (± 7 days, only applicable to Phase III), and at the early termination visit (if applicable).

Vital signs during the screening period will be used to determine whether the participant is eligible to participate in the trial. Vital sign monitoring before and after each administration of the investigational product during the treatment period is used to monitor infusion-related adverse reactions. The specific examination time points are: once within 2 hours before the start of intravenous infusion, and once 30 minutes (+ 30 min) after the completion of intravenous infusion.

Systolic and diastolic blood pressure will be measured at each time point, and it is recommended that participants rest quietly for at least 5 minutes before measurement. All recordings will be performed using standard equipment; automated or manual measurements are acceptable, but the same method should be used for all assessments of an individual participant throughout the trial.

7.2.2 Physical examinations

Physical examination involves skin, lymph nodes, eyes, head and neck, chest, abdomen, spine, and musculoskeletal system, among others.

Physical examinations will be performed during the screening period, at the D2 visit, D7 visit, D14 visit (D14 is applicable to participants in the 10 g/day group during the treatment period, as well as participants in the 20 g/day group during the follow-up period of Phase II and all participants in Phase III), D29 (± 3 days), D57 (± 5 days), D90 (± 7 days, only applicable to Phase III) visits, and at the early termination visit (if applicable). If necessary, the frequency of physical examinations may be increased, and should be aligned with the investigational product administration schedule as much as possible. In Phase III, if a participant receives re-treatment during the follow-up period, a physical examination is required at RD3 (if the medication is administered on the same day), RD7 (if the medication is administered on the same day), or at the re-treatment EOT visit.

During the screening period, the results of physical examinations will be used to comprehensively determine whether the participants meet the inclusion criteria, and the investigator will record their prior medical history after making their determination. During the treatment and follow-up period, the results of physical examinations will be used to comprehensively determine whether the participants experience any AE.

7.2.3 12-lead ECG

A 12-lead ECG will be performed during the screening period, at D3 and D7 visits of the treatment period, at the D14 visit of the treatment period (only applicable to participants in the 10 g/day group), at the EOT visit, at follow-up visits D29 (± 3 days), D57 (± 5 days), D90 (± 7 days, only applicable to Phase III), and at the early termination visit (if applicable). In Phase III, if a participant receives re-treatment during the follow-up period, a 12-lead ECG is required at RD3 (if the medication is administered on the same day), RD7 (if the medication is administered on the same day), or at the re-treatment EOT visit.

The main indicators of an ECG examination include heart rate, PR interval, QRS duration, QT interval, and QTc interval.

During the screening period, the results of ECG examinations will be combined to comprehensively determine whether the participants meet the inclusion criteria, and the investigator will record their prior medical history after making their determination. During treatment and follow-up periods, the results of the 12-lead ECG will be used to comprehensively determine whether the participant experiences AE.

7.2.4 Thyroid function test

Thyroid function tests will be performed during the screening period, at the D7 visit of the treatment period (D7 only applicable to participants in the 20 g/day group of Phase II and all participants in Phase III) or the D14 visit of the treatment period (D14 only applicable to participants in the 10 g/day group), at the EOT visit, at follow-up visits D29 (± 3 days), D57 (± 5 days), D90 (± 7 days, only applicable in Phase III), and at the early termination visit (if applicable).

The examination indicators mainly include thyroxine (T4), triiodothyronine (T3), thyroid-stimulating hormone (TSH), free T3, and free T4. The results of the screening test will be used to determine whether the participants meet the inclusion criteria. During the treatment and follow-up periods, the results of thyroid function tests will be combined to comprehensively determine whether the participants' thyroid function has deteriorated or whether any related AEs have occurred. If the investigator site is unable to perform thyroid function tests, tests

performed at an external hospital will be acceptable.

7.2.5 Thyroid ultrasound

Thyroid ultrasound will be performed during the screening period, at the D7 visit of the treatment period (only applicable to participants in the 20 g/day group of Phase II and all participants in Phase III) or the D14 visit of the treatment period (only applicable to participants in the 10 g/day group), at the EOT visit, at follow-up visits D29 (± 3 days), D57 (± 5 days), D90 (± 7 days, only applicable in Phase III), and at the early termination visit (if applicable). The results of the screening examination will be used to comprehensively determine whether the participants meet the inclusion criteria. During the treatment and follow-up periods, the results of thyroid ultrasound examination will be used to comprehensively determine whether the participants' thyroid function has deteriorated and whether any related AEs have occurred. If the investigator site is unable to perform thyroid ultrasound examinations, examinations performed at an external hospital will be acceptable.

7.2.6 Iodine uptake examination

During the Phase III study, iodine uptake examinations may be performed at the screening period, D7 visit of the treatment period, follow-up visits D29, D57, D90, and the early termination visit (if applicable) if the investigator deems it necessary. The specific examination methods (such as thyroid iodine-131 uptake test, serum iodine, and 24-hour urinary iodine) will be determined by the investigator based on the actual conditions of the site. Iodine uptake test may be omitted if no adverse thyroid events occur in the participant or if the investigator determines that it is unsuitable and/or unnecessary to conduct the test. If our site is unable to perform iodine intake testing, we can accept testing from other hospitals.

7.2.7 Fundus examination

Fundus examinations will be performed during the screening period, at the follow-up visit D57 (± 5 days) (Phase II) or D90 (± 7 days) (Phase III), and at the withdrawal visit (if applicable). The results of the screening period will be used to determine whether the participants meet the inclusion criteria. During the follow-up period, the results of fundus examination will be used to comprehensively determine whether the participant will experience any AEs. If the

investigator site is unable to perform fundus examinations, examinations performed at an external hospital will be acceptable.

7.2.8 Routine laboratory analysis

Routine laboratory tests include hematology, biochemistry, urinalysis, coagulation function tests, and stool analysis.

Routine stool examinations will be performed during the screening period, and the results will be used to determine whether the participants meet the inclusion criteria.

Hematology, biochemistry, coagulation function, and urinalysis will be performed during the screening period, at D2 (only hematology and biochemistry are required at the D2 visit), D3, and D7 visits of the treatment period, at the D14 visit (D14 is applicable to participants in the 10 g/day group during the treatment period, as well as participants in the 20 g/day group during the follow-up period of Phase II and all participants in Phase III; among these, only hematology and biochemistry are required for participants in the 20 g/day group of Phase II and all participants in Phase III), at the EOT visit, at follow-up visits D29 (± 3 days), D57 (± 5 days), D90 (± 7 days, only applicable in Phase III), and at the withdrawal visit (if applicable). If a participant receives re-treatment during the Phase III follow-up period, hematology, biochemistry, urinalysis, and coagulation function tests are required at RD3 (if the medication is administered on the same day), RD7 (if the medication is administered on the same day), or at the EOT visit for re-treatment.

The indicators for routine laboratory tests are described below, the specific indicators at each site may be slightly adjusted (external hospital examinations are acceptable):

- Hematology examination parameters include white blood cell count, absolute lymphocyte count, absolute neutrophil count, absolute monocyte count, absolute eosinophil count, absolute basophil count, neutrophil percentage, lymphocyte percentage, red blood cell count, hemoglobin, hematocrit, mean corpuscular volume, mean corpuscular hemoglobin level, and platelets.
- Urinalysis includes: specific gravity (SG), pH, bilirubin, urine protein, glucose (Glu), ketone bodies (Ket), urobilinogen, nitrites, occult blood, RBC, and WBC.
- Blood biochemistry examination include: lactate dehydrogenase (LDH), aspartate

aminotransferase (AST), alanine aminotransferase (ALT), gamma-glutamyl transferase (GGT), creatine kinase, alkaline phosphatase, total bilirubin, direct bilirubin, indirect bilirubin, total protein, albumin (ALB), globulin, urea (blood urea nitrogen), creatinine, uric acid, total cholesterol, triglycerides, high-density lipoprotein (HDL), low-density lipoprotein (LDL), sodium, potassium, chloride, calcium, magnesium, and glucose.

- Coagulation function tests include: prothrombin time, prothrombin activity, activated partial thromboplastin time, thrombin time, and international normalized ratio (INR).
- Routine stool examinations include: microscopic examination (fecal red blood cells, fecal white blood cells), and fecal occult blood test.

7.2.9 Infectious disease screening

Infectious disease screening will be performed during the screening period, including hepatitis B surface antigen and antibody tests (HBsAg, HBsAb, HBeAg, HBcAb, HBeAb; this test is not required for those with a history of hepatitis B), HBV DNA quantification (only performed on participants who are positive for hepatitis B surface antigen or have a history of hepatitis B), HCV antibody testing (this test is not required for participants with a history of hepatitis C), HCV RNA testing (only performed on participants who are positive for HCV antibody or have a history of hepatitis C), HIV antibody testing, and syphilis testing. HIV testing during the screening period is used to determine whether a participant meets the inclusion criteria, and examinations performed at an external hospital are acceptable.

7.2.10 Pregnancy testing

Women of childbearing potential (defined as pre-menopausal women and women within 2 years of menopause) will undergo pregnancy tests (either serum or urine pregnancy tests, at the investigator's discretion) during the screening period, at the D57 (± 5 days, Phase II) or D90 (± 7 days, Phase III) follow-up visit, and at the withdrawal visit (if applicable). This test is used to determine the participant's pregnancy status.

7.2.11 ECG

Echocardiography will be performed during the screening period; it is not required under other

circumstances. During the screening period, the results of the ECG will be used to comprehensively determine whether participants meet the eligibility criteria.

7.3 PD Evaluation

The pharmacodynamic blood sample collection time points and time windows for this study are detailed in Section 6.7. The PD endpoint of this trial is plasma colloid osmotic pressure (COP). PD characteristics will be evaluated by assessing plasma COP and its change from baseline following intravenous administration of the investigational product.

7.4 Immunogenicity Evaluation

The time points and time windows for collecting immunogenic blood samples in this study are detailed in Section 6.8.

The immunogenicity endpoints of this trial are the incidence and titers (if applicable) of anti-drug antibodies (ADA) against recombinant human serum albumin (rHSA) and against host cell proteins (HCP). Immunogenicity will be evaluated by assessing the incidence and titers (if applicable) of ADAs following administration of the investigational product.

7.5 Other Evaluations

7.5.1 Demographic

Demographic data will be collected during the screening period, including age, date of birth, gender, height, weight, drinking history, and ethnicity etc. These data will be recorded in the source records, and the data that does not involve the participant's personal information or privacy will be recorded in the eCRF.

7.5.2 Medical history

The medical history should record information on prior or concomitant diseases (or signs and symptoms if a definitive diagnosis cannot be established; AEs before treatment are excluded, refers to Section 8.1 for details) that occurred within 6 months of the first administration of the investigational product, including: history of allergies, kidney disease, cardiovascular disease, digestive system disease, cirrhosis complications, ocular disease, etc. Medical history should be documented in the original records and eCRF during the screening period. If any disease or

symptom that, in the investigator's judgment, is not expected to occur in the short term (such as cysts) occurs during the study, it should be recorded in medical history. Participants with specific prior or current medical conditions are not eligible to participate in this clinical study (refer to the inclusion and exclusion criteria in Sections 4.1 and 4.2).

For a history of cirrhosis complications: if a relevant diagnosis is documented in the original records, it shall be recorded in the medical history. If no diagnosis is made in the medical history, and only cirrhosis-related symptoms and clinically significant abnormal laboratory findings related to cirrhosis are present (i.e., if an abnormal laboratory finding is not diagnosed but is judged by the investigator to be related to cirrhosis), there is no need to record it in the medical history or as an AE during the screening period.

7.5.3 Treatment history

The treatment history shall record the treatments received by the patient within 6 months before signing the informed consent form, including at least the use of human plasma preparations (including human albumin preparations) within 20 days before the first administration of the investigational product, and the history of prior organ transplantation, etc. Only medications administered for therapeutic purposes shall be recorded in the treatment history. Medications used for diagnostic assistance, lysozymes, and the like do not need to be collected.

7.5.4 Exploratory evaluation

Glycated hemoglobin testing will be performed during the screening period, at the D7 visit during the treatment period (D7 is only applicable to participants in the 20 g/day dose group), or at the D14 visit (D14 is only applicable to participants in the 10 g/day dose group) in Phase II.

Glycated albumin testing will be performed before the first administration of the investigational product on D1 of the treatment period, at the D7 visit (D7 only applicable to participants in the 20 g/day dose group), or at the D14 visit (D14 only applicable to participants in the 10 g/day dose group) in Phase II.

Glycated hemoglobin (HbA1c) testing will be performed before the first administration of the investigational product on D1 (baseline), D29 (± 3 days), and D90 (± 7 days), or at the early termination visit (if applicable) during the Phase III treatment period.

Glycated albumin testing will be performed before the first administration of the investigational drug on day 1 of the Phase III treatment period (baseline), during the day 7 visit, or during the EOT visit.

Blood samples for glycated albumin testing must be stored in a freezer at -60 to -90°C (detailed requirements are participant to the laboratory manual) and transported to the central laboratory for testing. For details on the procedures for sample collection, processing, preservation, and transportation, please refer to the laboratory standard operating procedures or relevant clinical operation manuals.

8 ADVERSE EVENT

8.1 Definition of Adverse Event

According to the GCP, an AE is defined as any untoward medical event that occurs after a participant receives the investigational product, which may be manifested as symptoms, signs, diseases, or laboratory abnormalities, but may not necessarily have a causal relationship with the investigational product.

In order to collect more adequate information, information on AEs will be collected once the ICF has been signed until the end of the follow-up period at the last visit (D90). Pre-treatment AEs refer to clinical AEs that occur after the signing of the informed consent form but before the first use of investigational products, and must meet one of the following conditions: injury/damage caused by any clinical laboratory test procedure; AEs caused by discontinuation of the drug related to the trial protocol. Other symptoms/signs not mentioned above are recorded in the eCRF as medical history/concomitant diseases, and are not recorded as AEs.

A treatment-emergent adverse event (TEAE) is any undesirable medical event not present prior to medical treatment, or an already present event that worsens following the treatment. Symptoms/signs that are present at screening and do not worsen after the patients are enrolled in the study are not reported as TEAEs; however, deterioration of existing symptoms/signs during the study should be recorded as TEAEs.

AEs include but are not limited to the following:

1) AEs routinely observed and those that are expected from the pharmacological action of the investigational product.

2) Abnormal laboratory tests that the investigator considers clinically significant and that are related to accompanying symptoms.
3) Unintentional injury, any cause leading to medical, nursing, or pharmacological consultation, hospitalization or surgery.

Participants should be closely monitored for AEs during the study. The monitoring period for AEs is defined as starting from the participant's enrollment (date of signing the first informed consent form) until the completion of the last follow-up visit (D90). SAEs related to the investigational drug during the follow-up period (for the determination of relevance, please refer to Section 8.6 "Criteria for Causal Judgment of Adverse Events") will be monitored.

The sources of AEs include:

- Participants' answers to questions about their health status (non-leading questions at each visit such as "How have you been feeling since your last visit?").
- symptoms reported by the participants.
- Changes or abnormal findings or examination results assessed as clinically significant by the investigator.
- Additional information known to the investigator that is related to the participant's health (e.g., hospitalization).

8.2 Recording of AEs

All AEs will be coded and described using the Medical Dictionary of Regulatory Activities (MedDRA). During the trial, the AE record form should be completed accurately, including:

- Name of the AE (signs, symptoms, diagnosis, or syndrome);
- Start date and end date.
- Severity (CTCAE level 1-5).
- The investigator's causality assessment between the event and the investigational drug (definitely related, probably related, possibly related, unlikely related, definitely unrelated), including a description of other suspect procedures in the trial.
- Relevant measures taken for investigational drug.
- Event outcome.
- Is it a SAE? (if so, list the specific criteria that define it as a SAE).

- Drug treatment and other interventions provided to treat AEs.

If possible, AEs should be recorded according to the definitive diagnosis. If exact diagnoses are not available, the signs and symptoms for each AE should be recorded individually. Each diagnosis/symptom should be recorded.

If the same AE occurs more than once in a participant and the participant has recovered between the two occurrences, each of the events should be recorded as an AE. If the severity of an AE changes during its onset, it will be re-recorded as a separate AE. When re-recording an AE, the end date of the previous AE must be recorded.

It must be noted that the action taken is not an AE, but the reason for the action is an AE. Hospitalization is not an AE, but the reason for hospitalization is an AE. Death is not an AE, but the cause of death is an AE (except for sudden death of an unknown cause).

The start date of an AE is the date when the first sign or symptom is first observed. If the AE is a clinically significant and abnormal laboratory test result, the start time will be the date of sampling. The start date of a SAE is the date when the first sign or symptom is observed.

The end time of an AE is the date of recovery (return to normal or baseline), stabilization and no further improvement, reasonable explanation, or loss to follow-up. The end time can be recorded when the AE has resolved or the condition has stabilized; otherwise, the end time should not be recorded (e.g., when the outcome of a SAE is recovering/resolving, the end time should not be recorded). If no end time was collected at the time of death and the AE that is not a direct cause of the "death" still persists, then the end time of this AE should be left blank and its status should be "ongoing"; if it is judged to be a direct or major cause of the "death", then the end time should be the time of death of the participant.

8.3 Follow-up of AEs

To ensure participant safety, the investigator must take appropriate measures to follow up on all AEs until resolution, or until any clinically relevant changes in laboratory findings have returned to baseline, or a reasonable explanation is established. For participants who are ADA-positive at the end of the follow-up period, the sponsor and investigator shall determine whether to continue follow-up until the ADA result turns negative.

To ensure participant safety, the investigator must take appropriate measures to follow up on

all AEs until resolution, or until any clinically relevant changes in laboratory findings have returned to baseline, or a reasonable explanation is established. This means that even after the last visit specified in the protocol, further follow-up may be required, and the monitoring team may request the investigator to conduct additional follow-up. For AEs caused by the progression of underlying diseases, the investigator can conduct a full safety assessment at the end of the follow-up period to determine whether the participant needs further follow-up observation. All relevant follow-up information must be reported to Shenzhen Protgen Co., Ltd. The outcomes of AEs can be described in the following ways:

- Complete recovery (no sequelae): record the "adverse event end date", and follow-up can be terminated at this time;
- Recovered/Resolved with sequelae: the participant has recovered from the AE, but long-term or lifelong sequelae persist (such as blindness caused by diabetes, hemiplegia caused by stroke). Record the "adverse event end date", and the follow-up can be terminated at the same time;
- Recovering/Resolving: the status of the AE has improved compared to before, and follow-up is required. When no further improvement is expected, the end date of the AE can be recorded (the end time is not recorded when a SAE is classified as 'Recovering/Resolving').
- Not Recovered/Not Resolved: the severity of the event has not changed, and it is ongoing, requiring continued follow-up (when a SAE is classified as 'Not Recovered/Not Resolved', the end time is not recorded).
- Fatal: when an AE results in death, the time of death must be recorded. If the AE is determined to be the direct or primary cause of death, the participant's time of death should be recorded as the "adverse event end date".
- Unknown: investigators are unable to understand AEs, such as participant loss to follow-up;
- Worsened: the severity of the event has worsened, requiring a new AE to be recorded. The "end time of this AE" should be recorded.

If an AE is classified as "recovered/resolved (without sequel)" or "unknown", the end time of the AE may be temporarily omitted. If an AE is classified as "recovered/resolved (without sequel)" or "recovered/resolved (with sequel)", the AE end time must be recorded.

8.4 Abnormal Laboratory Test Results

The following criteria are used to determine whether an abnormal objective test result should be reported as an AE:

1) The test results were related to the accompanying symptoms;
2) The test result requires additional diagnostic tests or therapeutic measures/surgical interventions;
3) The test results in a change in the dosage of the investigational drug or discontinuation from the study, requiring medication or other treatment;
4) The investigator or sponsor believes that the test result should be reported as an AE.

Any abnormal laboratory findings determined to be errors are not required to be reported as AEs.

8.5 Severity of AEs

The investigator will refer to the 5-grade criteria established by NCI CTCAE V5.0 to grade (or assess) the severity of each AE based on the description of clinical symptoms, as follows:

- Grade 1: Mild, asymptomatic or mild symptoms; clinical or diagnostic observations only; intervention not indicated.
- Grade 2: Minimal, local or non-invasive intervention required; limiting age-appropriate instrumental activities of daily living (ADL) *.
- Grade 3: Severe or medically significant but not immediately life-threatening; hospitalization or prolongation of hospitalization indicated; disabling; limiting self-care ADL **.
- Grade 4: Life-threatening consequences; urgent intervention indicated.
- Grade 5: Fatal due to AE.

* Instrumental ADL refers to preparing meals, shopping for groceries or clothes, using the telephone, and managing money, etc.

** Self-care ADL refers to bathing, dressing and undressing, feeding oneself, using the toilet, taking medications, and not being bedridden.

8.6 Criteria for Causality Assessment of AEs

All AEs occurring during clinical trials must be assessed for their causality to the investigational drug. The analysis of the causality between AEs and the investigational drug needs to take into account the following factors:

1) Whether there is a reasonable temporal sequence between the occurrence of the AE and the onset of action of the investigational product.
2) Did the AE disappear or lessen after the investigational drug was discontinued?
3) Whether the AE recurs or worsens upon re-challenge with the investigational product.
4) Whether the AE is consistent with the known pharmacological and toxicological knowledge of the Investigational drug; whether there are clinical symptoms, laboratory findings, or imaging results to support the causality assessment.
5) Whether the AE can be explained by the participant's clinical status, psychological factors, concomitant medications or therapies, or other environmental factors.

The investigator should assess the potential correlations between AEs and the investigational drug based on five-level classification criteria as follows:

1) Definitely related: The timing of the reaction is consistent with a reasonable temporal sequence of investigational product administration; the reaction is consistent with the known type of adverse reaction for the investigational product; it cannot be explained by the participant's clinical condition, psychological factors, concomitant medication or treatment, or other environmental factors; the reaction recurs or worsens upon re-challenge with the investigational product and lessens or disappears upon discontinuation; and there is literature evidence consistent with the known adverse reactions of the investigational product.
2) Probably related: An event that follows a reasonable temporal sequence from administration of the study intervention, that follows a known or expected response pattern to the suspected intervention, that is unlikely to be explained by the participant's clinical state, psychological factors, concomitant medications or treatments, and other environmental factors, and that is confirmed by improvement on stopping.

3) Possibly related: An event that follows a reasonable temporal sequence from administration of study intervention, that follows a known or expected response pattern to the suspected intervention, but that could readily have been produced by the participant’s clinical state, psychological factors, concomitant medications or treatments, and other environmental factors. After obtaining more information, further determine whether it is "probably related" or "definitely related" depending on the circumstances.
4) Unlikely related: An event that follows a reasonable temporal sequence from administration of study intervention, that does not follow a known or expected response pattern to the suspected intervention of the investigational drug, and that could have been readily explained by the participant’s clinical state, psychological factors, concomitant medications or treatments, and other environmental factors.
5) Not related: An event that neither follows a reasonable temporal sequence from administration of study intervention nor follows a known or expected response pattern to the suspected intervention, that could be readily explained by the participant’s clinical state, psychological factors, concomitant medications or treatments, or there are other clearly explainable reasons.

The above criteria are for reference only. The assessment of causality should also consider many other factors, such as the patient's general condition, past medical history, and medication use. The investigator should make a comprehensive clinical judgment in combination with the above criteria.

8.7 Definition of SAE

SAEs refer to any untoward medical events that occur after a participant receives any dose of the investigational product, including the following:

1) Leading to death.
2) Life-threatening: Immediate risk of death to the participant, excluding AEs that may potentially lead to death upon serious progression.
3) Is a congenital anomaly or birth defect.
4) Results in persistent or significant disability/incapacity.

5) Results in hospitalization or prolongation of hospitalization. In general, hospitalization means that the participant is not suitable for observation or treatment in an outpatient or emergency setting, but requires formal admission or emergency observation (more than 24 hours). Prolongation of hospitalization refers to an adverse event occurring during hospitalization that delays the planned or expected discharge date of the participant (usually an overnight stay in the hospital).
6) Other important medical events. These events may not immediately result in life-threatening situations, death, or hospitalization, but based on medical judgment, they may jeopardize the participant or require medical intervention to prevent one of the aforementioned outcomes (e.g., allergic broncho's requiring intensive care, blood dyspepsias, seizures not resulting in hospitalization, progression to drug dependency or abuse). These events are also typically considered serious and necessitate prompt reporting based on medical and scientific judgment.

The following conditions do not qualify as hospitalization or do not need to be reported as SAEs: emergency room visits; observation within 24 hours; hospitalization for routine examinations (less than 24 hours); hospitalization for social reasons (e.g., due to lack of care); hospitalization for surgery scheduled prior to the study; hospitalization for anticipated disease progression of cirrhotic ascites and intervention for its complications; if a participant has a pre-existing condition before participating in the trial and the condition does not worsen during the trial, planned pre-trial hospitalization and/or surgical treatment are not considered AEs.

An adverse drug reaction refers to any harmful or unintended response occurring during a clinical trial that may be associated with the investigational drug. There must be at least a reasonable possibility of a causal relationship between the investigational drug and the AE, meaning the association cannot be ruled out.

An unexpected adverse reaction refers to an adverse reaction whose nature, severity, frequency, or outcome is not consistent with existing drug information (e.g., for an investigational drug not yet approved, it means inconsistency with the description in the Investigator's Brochure). When no Investigator's Brochure is available, the information is inconsistent with the risk information described in the study protocol or application materials.

A Suspected Unexpected Serious Adverse Reaction (SUSAR) must meet both of the following conditions simultaneously:

- Related: based on the causality assessment by the investigator/sponsor, there is a reasonable causal relationship between the SAE and the use of the investigational drug.
- Unexpected: the nature, severity, frequency, or consequences of a SAE exceed the information available in the investigator's manual, the package insert for marketed drugs, or the product characteristics summary.

8.8 Reporting of SAEs and Suspected Unexpected Serious Adverse Reactions

Reporting of SAEs and follow-up reports shall comply with local GCP and relevant institutional or organizational standard operating procedures, as detailed in the Risk Management Plan. Investigators shall report AEs to the sponsor promptly (within 24 hours whenever possible). When reporting SAE, the participant's identification code used in the clinical trial should be provided, rather than the participant's real name, citizen identification number, address, or other personal identifying information. AEs and laboratory abnormalities specified in the study protocol that are important for safety evaluation shall be reported to the sponsor according to the requirements and timelines defined in the study protocol. The collection timeframe for SAEs is the same as that for AEs.

For reports involving death events, investigators shall provide additional required documents to the sponsor and the ethics committee, such as autopsy reports and final medical reports. Other reporting requirements may follow the standard operating procedures of the institution or organization.

Reports identified as Suspected Unexpected Serious Adverse Reactions (SUSARs) will be submitted by the sponsor or contract research organization (CRO) to regulatory authorities, all institutions and investigators involved in the clinical trial, within the statutory timeframes; simultaneously, investigators are required to report to the ethics committee.

After receiving relevant safety information about the clinical trial provided by the sponsor, investigators shall promptly acknowledge and read the information, consider whether adjustments to the participant's treatment are needed, and communicate with the participant as

early as possible if necessary.

8.9 Reporting of Pregnancy Events

A pregnancy event refers to a pregnancy occurring in a female participant or the female partner of a male participant during the course of this study. To ensure the safety of participants, each pregnancy and its outcome in participants using the investigational drug (or the female partner of a male participant) must be reported to the sponsor using an appropriate pregnancy reporting form within 24 hours of becoming aware of the pregnancy event. Abnormal pregnancy outcomes should be considered SAEs and reported using the serious adverse event report form. The collection timeframe for pregnancy events is the same as that for SAEs.

Pregnancy itself is not considered an AE, unless there is suspicion that the investigational drug may affect the efficacy of contraceptive medication. Congenital malformations/birth defects and spontaneous abortions should be reported and handled as SAEs. Elective abortions without complications are not to be treated as AEs. All pregnancy outcomes (spontaneous abortion, elective abortion, ectopic pregnancy, normal delivery, or congenital abnormalities) should be followed up and recorded, even if the participant has already withdrawn from the trial.

If a pregnancy event occurs during the trial period in a participant (or the female partner of a male participant), the investigator should promptly decide, based on the specific circumstances, whether to discontinue the participant's participation in the clinical trial. Investigators should communicate scientifically and rigorously with participants based on medication information, informing them of the potential effects and risks of the investigational drug on pregnant women and fetuses, and allowing the participant to make their own decision regarding whether to terminate or continue the pregnancy.

Within 24 hours of confirming a pregnancy event in a participant (or the female partner of a male participant), the investigator shall complete the initial pregnancy report form and report it to the sponsor, and if necessary, also report it to the hospital ethics committee.

If the participant (or the female partner of a male participant) decides to terminate the pregnancy, the investigator shall complete the relevant section of the pregnancy report form within 24 hours of being informed of the termination and report it to the sponsor, and if necessary, also report it to the hospital ethics committee.

If the participant (or the female partner of a male participant) decides to continue the pregnancy, follow-up visits for the pregnancy should be conducted every 3 months until delivery occurs

and the pregnancy outcome is known. The investigator should complete the tracking report of the pregnancy report form within 24 h of each follow-up visit and report it to the sponsor, and if necessary, to the hospital's ethics committee.

9 RISK CONTROL AND MANAGEMENT

Based on the results of single-dose escalation studies in healthy participants, multiple-dose escalation studies in patients with cirrhotic ascites, Phase III studies of a similar drug (recombinant human serum albumin) in patients with cirrhotic ascites, and post-marketing safety surveillance data for human serum albumin (pHSA), the potential risks for participants participating in this trial are detailed in Section 1.4.1 "Potential Risks". Participants in this study may experience one or more AEs, including but not limited to those mentioned above. In this study, if a participant experiences an adverse drug event, medication should be discontinued promptly in accordance with the protocol, and intervention should be provided in a timely manner according to routine clinical treatment measures.

The trial site must be equipped with necessary medical resuscitation equipment, emergency medicine, and emergency measures. An emergency medical event response team may be established if necessary to handle emergency medical events and accidental injuries in accordance with relevant standard operating procedures. The investigator should pay close attention to possible AEs, especially unexpected AEs, analyze and communicate in a timely manner, and record AEs in detail. Communication procedures for the transfer and care of participants in the intensive care units of hospitals should be established. Procedures for the communications between the investigator, the laboratory, and the sponsor should be established to ensure timely communications and handling of any possible AE.

This trial specifies the organ function (including liver, kidney, hematopoietic, and cardiac ejection function) of the participants in the inclusion/exclusion criteria, and excludes participants with heart disease, kidney disease, gastrointestinal bleeding, and other serious, uncontrolled systemic diseases to avoid exacerbation of such diseases during the trial. During the treatment period, safety checks will be conducted according to the protocol, including: ECG of vital signs monitoring, thyroid function tests, thyroid ultrasound, hematology, blood chemistry, urinalysis, and coagulation function tests. At the end of the treatment period, vital signs monitoring, physical examination, hematology, blood chemistry, urinalysis, coagulation

function test, pregnancy test, ECG, thyroid function test, and thyroid ultrasound examination were performed. These examinations and observations by the investigator are aimed to detect any AE early and provide timely and necessary interventions. During the follow-up period, if any AEs occur to the participant or if any concomitant medications or treatments are used, the participant must report to the investigator in a timely manner, and may come to the hospital for necessary intervention if necessary. If the investigational drug is discontinued due to an AE related to the drug, follow-up is required until the outcome is achieved.

10 STATISTICAL ANALYSES

All statistical analyses will be performed using SAS version 9.4 or higher. In general, continuous variables will be described statistically using the number of participants, mean, median, standard deviation, minimum, and maximum values. Categorical and ordinal variables will be described statistically using the frequency and percentage of each category or rank. Unless otherwise specified, missing values will not be included in the calculation of percentages. No formal hypothesis testing will be performed in Phase II. However, hypothesis testing will be conducted for exploratory purposes, and some statistical methods will be used to explore and analyze the data in order to better understand it. Unless otherwise stated, all statistical tests will utilize a two-sided confidence level of $\alpha = 0.05$, with the calculation of two-sided 95% confidence intervals (CIs).

The primary efficacy endpoint for Phase III trials was tested using the equivalence hypothesis. The primary efficacy endpoint was the change in serum albumin level from baseline immediately after the final intravenous administration (based on central laboratory test results), to verify that the clinical efficacy of the investigational drug (rHSA) was equivalent to that of the comparator (pHSA). The proposed equivalence range is -2 to 2 g/L. Additionally, during Phase III of this study, a non-inferiority assessment will be conducted on the key secondary efficacy endpoint, which is the improvement rate of ascites at the completion of the final intravenous administration. A non-inferiority margin of -10% is proposed for this assessment.

Except for the primary endpoint and key secondary endpoints, other efficacy endpoints will be analyzed based on observational data (i.e., missing data will not be imputed). For details of the specific statistical analysis methods used in this study, please refer to the Statistical Analysis Plan (SAP).

10.1 Estimand

10.1.1 Primary estimand

- **Clinical question of primary interest:** whether the change from baseline in serum albumin level immediately after completion of the final intravenous administration of rHSA (based on central laboratory test results) is equivalent to that of plasma-derived human serum albumin (pHSA) in patients with cirrhotic ascites and hypoalbuminemia.
- **Attributes of the Primary Estimand**
  - **Target population:** patients with cirrhotic ascites and hypoalbuminemia who meet the inclusion criteria but not the exclusion criteria.
  - **Target variable:** change from baseline in serum albumin level immediately after completion of the final intravenous administration.
  - **Treatment:** participants are randomized to receive either rHSA or pHSA, administered at 20 g/day for 7 consecutive days, or until serum albumin level reaches the discontinuation criterion (ALB ≥ 40 g/L), whichever occurs first.
  - **The intercurrent events and their handling strategies are shown in Table1:**

Table 1. Intercurrent Events and Their Handling Strategies for the Primary Estimand

Intercurrent event	Addressing strategies	Note
Premature treatment discontinuation due to inadequate increase in albumin level after treatment	Composite strategy	Data collected after treatment discontinuation are not used in the primary estimand analysis. For the resulting missing primary efficacy endpoint data, the multiple imputation (MI) method will be applied to impute values before reanalysis, using data from more than 5% of participants with the worst on-treatment efficacy (based on the change from baseline in serum albumin) within each corresponding treatment group. The minimum sample size required for model convergence will be determined based on actual data, with the number of participants increased in steps of 5.
Premature treatment discontinuation due to adverse event	Treatment strategy	Data collected after premature treatment discontinuation will be included in the primary estimand analysis
Premature treatment discontinuation due to other reasons, e.g., participant withdrawal of informed consent	Treatment strategy	Data collected after premature treatment discontinuation will be included in the primary estimand analysis
Increase in baseline dose of diuretics during the	Composite strategy	Data collected after treatment discontinuation are not used in the primary estimand analysis. For the

Intercurrent event	Addressing strategies	Note
treatment period; concomitant use of other albumin, blood products, or prohibited medications/treatments; death		resulting missing primary efficacy endpoint data, the multiple imputation (MI) method will be applied to impute values before reanalysis, using data from more than 5% of participants with the worst on-treatment efficacy (based on the change from baseline in serum albumin) within each corresponding treatment group. The minimum sample size required for model convergence will be determined based on actual data, with the number of participants increased in steps of 5.

- **Population-level summary:** The difference between groups in the least squares mean of the change from baseline in serum albumin level, along with its 95% confidence interval (CI). The analysis will be based on a Covariance model, with baseline serum albumin as a covariate and including randomization stratification factors.

10.1.2 Key secondary estimand

- **Clinical question of interest:** Whether the ascites improvement rate at the completion of the final intravenous administration of rHSA is non-inferior to that of pHSA in patients with cirrhotic ascites and hypoalbuminemia.
- **Attributes of the Estimand**
  - **Target population:** Patients with cirrhotic ascites and hypoalbuminemia who meet the inclusion criteria but not the exclusion criteria.
  - **Target variable:** Ascites improvement rate at the completion of the final intravenous administration.
  - **Treatment:** Participants are randomized to receive either rHSA or pHSA, administered at 20 g/day for 7 consecutive days, or until serum albumin level reaches the discontinuation criterion (ALB ≥ 40 g/L), whichever occurs first.
  - **The intercurrent events and their handling strategies are shown in Table 2**:

Table 2. Intercurrent Events and Handling Strategies for Key Secondary Estimand

Intercurrent event	Addressing strategies	Note
Premature treatment discontinuation due to inadequate improvement in ascites after treatment	Composite strategy	Considered as no improvement in ascites depth

Intercurrent event	Addressing strategies	Note
Premature treatment discontinuation due to adverse event	Treatment strategy	Data collected after premature treatment discontinuation will be included in the primary estimand analysis
Premature treatment discontinuation due to other reasons, e.g., participant withdrawal of informed consent	Treatment strategy	Data collected after premature treatment discontinuation will be included in the primary estimand analysis
Increase in baseline dose of diuretics during the treatment period; concomitant use of other albumin, blood products, or prohibited medications/treatments; death	Composite strategy	Considered as no improvement in ascites depth

- **Population-level summary:** The difference between groups in the ascites improvement rate (investigational drug group - comparator group) along with its 95% CI.

10.1.3 Other efficacy endpoints

Statistical analyses will be performed in accordance with the general principles of statistical analysis, as detailed in the Statistical Analysis Plan (SAP).

10.2 Statistical Assumptions and Sample Size Estimation

This Phase II study is an exploratory phase and does not involve formal hypothesis testing. Currently, there is no efficacy data or references for similar drugs. Based on preliminary data from the target population, a total of 92 participants are planned to be enrolled in Phase II. Participants are stratified by baseline pHSA (< 25 or ≥ 25 g/L) and randomized in a 1:1:1:1 ratio to the investigational drug group (10 g/day, n = 23), the investigational drug group (20 g/day, n = 23), the comparator group (10 g/day, n = 23), and the comparator group (20 g/day, n = 23). The sample size for Phase III will be determined based on the results of Phase II study.

In the Phase III phase of this trial, the equivalence hypothesis was tested. The change in serum albumin level from baseline immediately after the final intravenous administration (based on the results of central laboratory tests) was used as the primary efficacy endpoint to verify that the clinical efficacy of the investigational drug (rHSA) was equivalent to that of the comparator (pHSA). Based on the Phase III trial design of similar domestic products and relevant expert recommendations, ± 2 g/L is proposed as the upper and lower limits of equivalence.

Based on research experience with similar products and Phase II data of the investigational drug, the standard deviation of the primary efficacy endpoint between the investigational drug and the comparator is expected to be no more than 5 g/L, and the efficacy of the investigational drug and the comparator is similar. At a significance level of 0.025 for both one-sided and two-sided studies, a 90% confidence level can be achieved in 328 cases (164:164).

In the Phase III of this study, the ascites improvement rate at the completion of the final intravenous administration (ascites improvement at the individual level is defined as: ascites reduction in patients with baseline grade 1 ascites, ascites downgrading in patients with baseline grade 2 ascites, or a decrease in ascites depth of 25% or more from baseline; ascites improvement rate is defined as the proportion of participants in the investigational drug group/comparator group who meet the ascites improvement criteria) will be used as the key secondary efficacy endpoint, and non-inferiority will be assessed. At a one-sided significance level of 0.025, -10% is proposed as the non-inferiority margin. Results from the Phase II study of the investigational drug showed that the ascites improvement rates at completion of the final intravenous administration were 60.87% and 50.00% in the 20 g/day investigational drug group and the 20 g/day comparator group, respectively. The between-group difference in ascites improvement rate (investigational drug group minus comparator group; without adjustment for covariates, using Fisher's exact test) was 10.9%. When adjusted for baseline ALB levels (< 25 or ≥ 25 g/L), baseline ascites depth (< 3 cm; 3–6 cm [including 3, excluding 6]; or 6–10 cm [including both 6 and 10]), and baseline use of diuretics (yes or no) as covariates, the between-group difference in ascites improvement rate (investigational drug group minus comparator group) was 5.8%. Therefore, after comprehensive consideration of data from similar products and the Phase II study results of the investigational drug, and assuming an ascites improvement rate of 50% in the control group, a conservatively estimated difference in ascites improvement rates (experimental group minus control group) of 5%, and a dropout rate of 10%, a total of 388 participants are required to achieve a power of more than 84%.

The methodology for sample size re-estimation is described in detail in the Interim Analysis section.

10.3 Interim Analysis

This Phase III trial is planned to conduct one unblinded interim analysis when at least 328 subjects have completed the D7 visit (i.e., when data for the primary efficacy endpoint are collected). The interim analysis will perform an equivalence hypothesis test on the primary efficacy endpoint and conduct sample size re-estimation based on the effect size of the secondary key efficacy endpoint(s). The interim analysis will be executed by the IDMC. Details will be specified in the Statistical Analysis section and the IDMC Charter.

The decision for sample size re-estimation will be made following the method described by Cyrus R. Mehta in the referenced literature [25], as detailed below:

Assume the maximum allowable total sample size (n_{max}) for the entire trial is set to twice the initial sample size (388 subjects), i.e., 776 subjects (388 per treatment group). According to literature [25], set the Minimal Conditional Power (CP_{min}) to 0.36 and the Unconditional Power (UP) to 80% (where UP = 1-β, β being the type II error rate). The values of CP_{min} and UP divide the interval [0, 1] into three zones:

- Unfavorable Zone: The Conditional Power (CP) calculated based on the interim analysis results is lower than CP_{min}. In this case, the sample size will not be adjusted.
- Promising Zone: The CP calculated based on the interim analysis results is greater than CP_{min} but less than UP. In this case, the sample size will be increased based on the interim results to achieve a final analysis power of at least 80%, but the final adjusted sample size will not exceed n_{max}.
- Favorable Zone: The CP calculated based on the interim analysis results is greater than UP. In this case, the sample size will not be adjusted, and the trial will continue with the initial sample size. Further details regarding the interim analysis will be provided in the IDMC Charter and the Statistical Analysis Plan (SAP).

10.4 Statistical Analysis Population

10.4.1 Full analysis dataset (FAS)

All participants who were randomized and received at least one dose of the investigation product will be included in the full analysis dataset. This dataset will be used for the primary

analysis of patient distribution, demographics, and baseline characteristics, as well as for the primary analysis of efficacy in Phase II and Phase III trials.

10.4.2 Per protocol set (PPS)

The eligible participants in this trial consist of all participants who were randomized to receive at least one dose of the investigation product, did not commit any major protocol violations, and did not receive any treatment that could significantly affect the assessment of efficacy. The compliant protocol set will be used for supplementary analysis of the effectiveness of Phase II and Phase III.

10.4.3 Safety set (SS)

Participants who were randomized to receive at least one dose of the investigation product and who underwent at least one safety evaluation after administration were included in the safety set. The SS represents the safety evaluation population for this trial.

10.4.4 PD analysis set

All participants who were randomized and received at least one dose of the investigation product, and who had at least one effective plasma colloid osmotic pressure data after dosing, were included in the PD analysis set of this study. The PD analysis set will be used for pharmacodynamic analysis.

10.4.5 Immunogenicity analysis set

All participants who were randomized and received at least one dose of the investigation product and had immunogenicity data after at least one dose were included in the immunogenicity analysis set, which will be used for immunogenicity analysis.

10.4.6 Retreatment analysis set

All participants who received retreatment with the investigation product in Phase III trials were included in the retreatment analysis set, which will be used to evaluate the safety and efficacy of retreatment.

10.5 Statistical Analysis Method

10.5.1 Demographic data and other baseline characteristics

Descriptive statistics were used to tabulate and descriptively analyze demographic data and other baseline characteristics (treatment history, medication history, etc.).

10.5.2 Efficacy analysis

During Phase II of this trial, descriptive statistics will be utilized to assess efficacy. The changes in serum albumin level from baseline were summarized immediately after the final intravenous administration of the investigational drug and the comparator at each dose, as confirmed by albumin testing. Additionally, changes in ascites depth, abdominal girth, and body weight from baseline at each post-initial intravenous administration visit will be summarized for comparisons between the investigational drug and comparator groups at each dose level. The proportions of participants achieving serum albumin levels of 35 g/L or higher at the last dose, along with their corresponding confidence intervals (CIs), will be calculated separately for the two dose groups of the investigational drug, the two dose groups of the comparator, and for comparisons between the investigational drug and comparator groups at each dose level. The difference in proportions and its associated two-sided 95% confidence interval will also be computed, and a bar chart illustrating these proportions will be generated. The Kaplan-Meier method will be used to generate plots illustrating the time to achieve serum albumin levels of 35 g/L or higher for the investigational drug and comparator groups, as well as for comparisons between these groups at each dose level. The median, first quartile, and third quartile values for these times will also be calculated.

Analysis of key estimation targets in Phase III:

- **Primary Estimation Method:** in Phase III of this trial, the primary endpoint is the change in serum albumin from baseline measured immediately after the completion of the final intravenous administration, with equivalence margins set at -2 and 2 g/L. The ANCOVA model will be used, with the change in serum albumin from baseline as the dependent variable, the treatment group as a fixed effect, and the baseline ALB level (continuous result), the baseline ascites depth (< 3 cm; 3-6 cm [inclusive of 3, exclusive of 6]; or 6-10

cm [inclusive of 6 and 10]), and whether diuretics were used at baseline (yes or no) as covariates for analysis. The least squares means (LSM) and standard errors (SE) for the change in serum albumin from baseline immediately after the final intravenous administration, along with their 95% CIs, will be calculated for both groups. Additionally, the difference in LSMs between the groups (investigational drug group – comparator group) and its corresponding 95% CI will be computed.

- **Sensitivity analysis, supplementary analysis, and subgroup analysis are presented in Table 3 :**

Table 3 Sensitivity, Supplementary, and Subgroup Analysis for the Primary Estimand in the Phase III Stage

Category	Description of analytical procedure
Sensitivity Analysis 1	An ANOVA model will be employed, with the treatment group as a fixed effect and without considering covariates; all other aspects will align with the primary estimation analysis method.
Supplementary Analysis 1	For intercurrent events, including premature treatment termination due to lack of efficacy or adverse events, death, change in concomitant baseline diuretic therapy during the treatment period, and use of other albumin or blood products or prohibited medications/therapies during treatment, a hypothetical strategy will be applied. Specifically, efficacy data collected after the occurrence of these intercurrent events will not be used in the analysis. The resulting missing data will not be imputed. Instead, the analysis will be conducted using a Mixed Model for Repeated Measures (MMRM).
Supplementary Analysis 2	For intercurrent events including premature treatment termination due to lack of efficacy or adverse events, change in concomitant baseline diuretic therapy during the treatment period, and use of other albumin or blood products or prohibited medications/therapies during treatment, a treatment policy strategy will be applied. Specifically, efficacy data collected after the occurrence of these intercurrent events will be used in the analysis.
Supplementary Analysis 3	Analyses will be conducted on the PPS, with all other methodological aspects remaining identical to those of the primary estimand analysis.

Category	Description of analytical procedure
Subgroup analysis	Subgroup analyses will be performed based on randomization stratification factors and key baseline characteristics, including but not limited to baseline serum albumin level (< 25 or ⩾ 25 g/L), baseline ascites depth (< 3 cm; 3 to < 6 cm [inclusive of 3, exclusive of 6]; or 6–10 cm [inclusive of 6 and 10]), and baseline use of diuretics (yes or no). Analyses will be conducted using the primary estimand analysis method

Phase III Key Secondary Estimand Analysis

- **Primary Estimation Method:** the key secondary efficacy endpoint for Phase III of this trial is defined as the improvement rate of ascites upon completion of the final intravenous administration, for which a non-inferiority margin of –10% has been prespecified. Between-group comparisons will be adjusted for randomization stratification factors, including baseline serum human albumin level (< 25 or ⩾ 25 g/L), baseline ascites depth (< 3 cm; 3 to < 6 cm [inclusive of 3, exclusive of 6]; or 6-10 cm [inclusive of 6 and 10]), and baseline use of diuretics (yes or no). The between-group difference in proportions and the corresponding 95% confidence interval will be calculated using the Newcombe method.
- **Sensitivity analysis, supplementary analysis, and subgroup analysis are presented in Table 4 :**

Table 4 Sensitivity, Supplementary, and Subgroup Analysis for the Key Secondary Estimand in the Phase III Stage

Category	Description of analytical procedure
Sensitivity Analysis 1	Analyses will be performed without correction for stratification factors, while all other methodological components remain congruent with those delineated in the primary estimand analysis.
Supplementary Analysis 1	Analyses will be conducted on the PPS, with all other methodological aspects remaining identical to those of the primary estimand analysis.
Subgroup analysis	Subgroup analyses will be conducted contingent upon randomization stratification factors and salient baseline characteristics—encompassing, though not limited to, baseline serum albumin level (< 25 or ≥ 25 g/L), baseline ascites depth (< 3 cm; 3 to < 6 cm [inclusive of 3, exclusive of 6]; or 6-10 cm [inclusive of 6 and 10]), and baseline diuretic use (yes or no).

Phase III Secondary Efficacy Endpoints Analyses

Changes from baseline in ascites depth, abdominal circumference, and body weight after dosing will be analyzed using the same primary estimand analysis method (ANCOVA) as specified for the primary estimand. For each endpoint, the change from baseline will be used as the dependent variable, with treatment group included as a fixed effect. Covariates will include the baseline value of the respective endpoint, baseline serum albumin level (< 25 or ⩾ 25 g/L), baseline ascites depth (< 3 cm, 3 to < 6 cm [inclusive of 3, exclusive of 6]; or 6-10 cm [inclusive of 6 and 10]), and baseline use of diuretics (yes or no). For the analysis of ascites depth, baseline ascites depth will not be included as a covariate. Time-course plots of these endpoints will also be generated. Additionally, the proportion of participants attaining a serum albumin level ≥ 35 g/L, as confirmed by laboratory assessment at the completion of the final intravenous administration, will be computed along with its corresponding confidence interval. The between-group difference in this proportion (investigational drug group-comaprator group) and its associated two-sided 95% CI will likewise be estimated. A bar chart illustrating these proportions will be constructed. Time to the first achievement of serum albumin level ≥ 35 g/L (confirmed by laboratory assessment) will be analysed using the Kaplan-Meier (KM) method, and the corresponding KM curves will be provided.

During the retreatment period, the change from pre-retreatment baseline in serum albumin level at the completion of the final intravenous administration will be analyzed using an analysis of covariance (ANCOVA) model. The change from pre-retreatment baseline in serum albumin will be used as the dependent variable, with treatment group included as a fixed effect and pre-retreatment baseline serum albumin level included as a covariate. For each treatment group, the least squares mean (LSM) and standard error (SE) of the change will be estimated, along with the between-group difference in the change from pre-retreatment baseline and the corresponding two-sided 95% confidence interval (CI). Statistical analyses for other efficacy endpoints during the retreatment period will be conducted in a manner consistent with those employed during the primary treatment period.

10.5.3 Safety analysis

AEs will be encoded using the Medical Dictionary for Regulatory Activities (MedDRA). All analyses of AEs will be predicated upon treatment-emergent adverse events (TEAEs). All

TEAEs, investigational drug-related TEAEs, and SAEs were summarized and analyzed by system organ classification (SOC) and preferred term (PT), and the number of occurrences and incidence rates were calculated. Descriptive statistics will be used to summarize vital signs, ECG parameters, laboratory assessments, and thyroid function tests, as well as their changes relative to baseline. Crosstabs will be used to describe the clinical significance of changes relative to baseline. Descriptive statistics will be used to summarize changes in iodine uptake testing (if applicable, only for Phase III) and its relative to the initial test, and corresponding shift tables will be generated to illustrate clinically meaningful deviations relative to the initial measurement.

10.5.4 Pharmacodynamic analysis

For the Phase II study, descriptive statistical analyses will be undertaken to summarise plasma colloid osmotic pressure (COP) values, as well as changes from baseline in COP after the first intravenous administration. These analyses will be performed separately for both dose levels of the investigational drug and both dose levels of the comparator, and additionally, at each dose level, for the comparison between the investigational product and the comparator.

In the Phase III study, descriptive statistical analyses will be conducted to summarise plasma colloid osmotic pressure (COP) values and their corresponding changes from baseline after the first intravenous administration, comparing the investigational product group with the comparator group.

10.5.5 Immunogenicity analysis

The incidence and titers of anti-rHSA and anti-HCP antibodies will be summarized (if applicable). If applicable, the impact of ADAs on the PD, effectiveness, and safety of rHSA will be assessed.

10.5.6 Exploratory endpoint analysis

In Phase II, descriptive statistical analyses will be performed on the change from baseline in glycated albumin for the two dose levels of the investigational product and the two dose levels of the comparator, as well as for the comparison between the investigational product and the comparator at each dose level.

In Phase III, descriptive statistical analyses will be performed on the change from baseline in

glycated albumin and glycated hemoglobin for the investigational product group (Group A) and the comparator group (Group B).

11 DATA PROCESSING

11.1 Source Data and Source Document

Source data

Source data refers to all information recorded in the original records or verified copies in a clinical trial, including clinical findings, observations, and other relevant records necessary to reconstruct and evaluate the conduct of the clinical trial.

Source document

Source documents refer to the original records, documents, and data generated during a clinical trial, such as hospital medical records, medical images, laboratory records, memos, participants' diaries or assessment forms, drug dispensing records, data recorded by automated instruments, microfilm, photographic negatives, magnetic media, X-rays, participant files, and clinical trial–related documents and records maintained by the pharmacy, laboratory, and medical technology departments, including verified copies. Source documents containing source data, may be available in paper or electronic form.

CRF

This study uses electronic case report forms (eCRFs), which are to be completed by the investigator or their authorized personnel via the electronic data capture and management system (EDC). The CRFs will be set up in the EDC system prior to the start of the study, and an account will be assigned to each investigator and/or their authorized personnel responsible for CRF completion at the study site.

1) For all participants who have signed the ICF, each item in the CRF shall be completed carefully and in detail per the CRF completion instructions.
2) All data in the CRF should be checked against the participant's source documents to ensure accuracy.
3) Data that are significantly high or outside the clinically acceptable range must be verified and, if necessary, reviewed and confirmed by the investigator.

4) For further details, refer to the eCRF Completion Guidelines.

The completed eCRF is first reviewed by the CRA and then verified and managed by the data manager. Once data collection and cleaning are completed, the CRF is electronically signed by the investigator for confirmation.

Source data required by the protocol should be entered into the CRF accurately, completely, legibly, and in a timely manner.

The completed CRFs are the sole property of the sponsor and shall not be disclosed to any third party in any form without the sponsor's written permission, except to authorized representatives of relevant regulatory authorities or ethics committee (EC).

11.2 Data Management

1) An electronic data management system will be used to collect data in this study. Establishment of eCRF: The data administrator will establish eCRFs according to the study protocol.
2) Access assignment: The data manager is responsible for creating user accounts and assigning access privileges according to personnel roles, such as data entry personnel, investigators, and clinical monitors. Data entry personnel have permissions for data entry, modification, and query feedback. Investigators have permissions for modification, viewing, query feedback, and review. The CRA have permissions for viewing and issuing queries. Data managers have permissions for viewing, issuing queries, and data locking.
3) Data entry: Clinical researchers or data entry personnel designated by the investigator (clinical coordinators) shall accurately and timely enter data from the research medical records into the eCRF.
4) Issuance and resolution of queries: Monitors and data managers send all queries through the eCRF. Data entry personnel or investigators answer questions and correct erroneous data. Queries can be resent if necessary. All records are saved in the eCRF.
5) Data modification and review: Data entry personnel or investigators can modify data after verifying it. Reasons for modification must be filled in on the eCRF for any modified data. The investigator has the authority to review all final data.
6) Data Locking and Export: After all data is reviewed and confirmed to be error-free, the data manager locks the data. If modifications are needed after data locking, they can only be executed after confirmation and signature by the Sponsor, data

management personnel, and statistician. All data is ultimately exported by data management personnel and handed over to statistical personnel for analysis.

11.3 Storage of Documents

Essential documents for clinical trials refer to documents that can be used individually or collectively to evaluate the conduct of the clinical trial and the quality of trial data. Essential documents for clinical trials are used to demonstrate that the investigator, Sponsor, and monitor have complied with GCP and relevant laws and regulations for drug clinical trials during the clinical trial process.

Essential documents are important content for Sponsor audits and inspections of clinical trials by drug regulatory authorities, and serve as the basis for confirming the authenticity of clinical trial conduct and the integrity of collected data.

The Sponsor, investigator, and clinical trial institution shall confirm that they have places and conditions for storing essential documents for clinical trials. The equipment conditions for storing documents should be capable of preventing direct light exposure, water, fire, etc., which is conducive to the long-term preservation of documents. Standard operating procedures for document management should be established. Stored documents need to be easy to identify, locate, retrieve, and return. Media used to store clinical trial data should ensure that source data or their certified copies are preserved intact and readable during the retention period, and the ability to restore reading should be tested or checked regularly to prevent intentional or unintentional alteration or loss.

For some documents generated during the implementation of clinical trials, if they are not listed in the management catalog of essential documents for clinical trials, the Sponsor, investigator, and clinical trial institution may also include them in their respective essential document archives for preservation based on necessity and relevance.

For clinical trials used to apply for drug registration, essential documents should be preserved for at least 5 years after the investigational drug is approved for marketing. For clinical trials not used to apply for drug registration, essential documents should be preserved for at least 5 years after the termination of the clinical trial. All data of this clinical research belongs to Shenzhen Protgen Co., Ltd. Except as required by the National Health Commission, the investigator shall not provide it to any third party in any form without the written consent of the Sponsor.

The Sponsor shall ensure that the investigator can always access and enter/correct data in the Case Report Form reported to the Sponsor during the trial, and such data should not be controlled solely by the Sponsor.

The Sponsor shall ensure that the investigator can retain the Case Report Form data that has been submitted to the Sponsor. Copies used as source documents should meet the requirements for certified copies.

At the beginning of the clinical trial, both the investigator/clinical trial institution and the Sponsor shall establish archive management for essential documents. At the end of the clinical trial, the monitor shall review and confirm the essential documents of the investigator/clinical trial institution and the Sponsor. These documents should be properly preserved in their respective clinical trial archive files.

12 CLINICAL MONITORING

To ensure the rights and interests of participants in the trial, ensure the accuracy and integrity of trial records and reported data, and ensure that the trial complies with the approved protocol, GCP, and relevant regulations, the Sponsor shall appoint qualified monitors who have received appropriate training and possess the scientific and clinical knowledge required for clinical trial monitoring to monitor the clinical trial. The main responsibilities of the monitor are:

1) The monitor should be familiar with relevant knowledge of the investigational product, the clinical trial protocol, the informed consent form, and other written materials provided to participants, as well as standard operating procedures for clinical trials and relevant regulations such as GCP.
2) The monitor shall conscientiously fulfill monitoring duties according to the Sponsor's requirements to ensure that the clinical trial is conducted and recorded correctly according to the trial protocol.
3) The monitor is the main contact person between the Sponsor and the investigator. Before the clinical trial, confirm that the investigator has sufficient qualifications and resources to complete the trial, and that the clinical trial institution has appropriate conditions to complete the trial, including staffing and training, complete laboratory equipment in good working order, and various examination conditions related to the trial.

4) The monitor shall verify that during the clinical trial, the investigational product is within the validity period, storage conditions are acceptable, and supply is sufficient; the investigational product is provided only to eligible participants according to the dosage specified in the trial protocol; participants receive instructions on the correct use, handling, storage, and return of the investigational product; the clinical trial institution has appropriate controls and records for the receipt, use, and return of the investigational product; and the disposal of unused investigational products by the clinical trial institution complies with relevant laws, regulations, and Sponsor requirements.
5) The monitor verifies the investigator's execution of the trial protocol during the implementation of the clinical trial; confirms that all participants or their guardians have signed the informed consent form before the trial; ensures that the investigator receives the latest version of the Investigator's Brochure, all trial-related documents, and essential trial supplies, and implements them according to the requirements of relevant laws and regulations; and ensures that researchers have a full understanding of the clinical trial.
6) The monitor verifies that researchers fulfill the responsibilities specified in the trial protocol and contract, and whether these responsibilities are delegated to unauthorized personnel; confirms that enrolled participants are eligible and reports the enrollment rate and progress of the clinical trial; confirms that data recording and reporting are correct and complete, and trial records and documents are updated in real-time and well-preserved; verifies that all medical reports, records, and documents provided by the investigator are traceable, clear, synchronously recorded, original, accurate, complete, and marked with dates and trial numbers.
7) The monitor checks the accuracy and integrity of Case Report Form entries and compares them with source documents. The monitor shall pay attention to checking that data specified in the trial protocol are accurately recorded in the Case Report Form and are consistent with source documents; confirm that participant dose changes, treatment changes, AEs, concomitant medications, complications, loss to follow-up, missed examinations, etc., are all recorded in the Case Report Form; confirm that follow-ups not achieved by the investigator, unimplemented trials, unperformed

examinations, and whether corrections to errors or omissions have been made are all recorded in the Case Report Form; and verify that the withdrawal and loss to follow-up of enrolled participants have been recorded and explained in the Case Report Form.

8) The monitor shall notify the investigator of any errors, omissions, or illegible handwriting in the Case Report Form; the monitor shall ensure that any corrections, additions, or deletions made are operated by the investigator or authorized personnel, and have the modifier's signature and date, with reasons for modification explained if necessary.
9) The monitor confirms that AEs have been reported within the specified timeframe according to the requirements of relevant laws and regulations, the trial protocol, the Ethics Committee, and the Sponsor.
10) The monitor confirms whether the investigator has preserved essential documents according to GCP.
11) For deviations from the trial protocol, standard operating procedures, and relevant laws and regulations, the monitor should communicate with the investigator in a timely manner and take appropriate measures to prevent recurrence.
12) After each monitoring visit, the monitor shall submit a written report to the Sponsor in a timely manner; the report shall state the monitoring date, location, monitor's name, names of investigators and other personnel contacted by the monitor, problems found during monitoring, etc.; the report shall include a summary of monitoring work, statements of problems and facts found in the clinical trial, deviations and deficiencies from the clinical trial protocol, and monitoring conclusions; the report shall also explain corrective measures taken or to be taken for problems found during monitoring, and suggestions for ensuring trial compliance with the protocol; the monitoring report should provide sufficient details for inspection and audit of compliance with the monitoring plan. Centralized monitoring reports can be submitted separately from on-site monitoring reports. The Sponsor shall review and follow up on issues in the monitoring report and document them for preservation.

13 QUALITY CONTROL AND ASSURANCE

1) The Sponsor and/or the Contract Research Organization (CRO) entrusted by it are responsible for formulating, implementing, and updating standard operating procedures for the clinical trial quality assurance and quality control systems in a timely manner to ensure that the conduct of the clinical trial and the generation, recording, and reporting of data all comply with the requirements of the trial protocol, GCP, and relevant laws and regulations. The Sponsor may entrust part or all of the work and tasks of its clinical trial to a CRO, but the Sponsor remains the ultimate person responsible for the quality and reliability of clinical trial data and shall supervise the various tasks undertaken by the CRO. The CRO shall implement quality assurance and quality control.
2) The entire process of clinical trials and laboratory testing must be strictly carried out in accordance with quality management standard operating procedures. There is quality control at every stage of data processing to ensure that all data is reliable and the data processing process is correct. Trial participants receive training on the trial protocol, current GCP, and relevant standard operating procedures currently in force for this study before the trial starts, so that researchers have a full understanding and recognition of the clinical trial protocol and the specific connotations of its various indicators.
3) The Sponsor shall sign contracts with all relevant units participating in the clinical trial, such as investigators and clinical trial institutions, to clarify the responsibilities of each party.
4) The contracts signed by the Sponsor with relevant units shall state that the Sponsor's monitoring and auditing, and the inspection by drug regulatory authorities can go directly to the trial site to consult source data, source documents, and reports.

14 PROTOCOL COMPLIANCE AND DEVIATION

The investigator shall conduct the trial in accordance with the clinical trial protocol approved by the Ethics Committee. Without the consent of the Sponsor and the Ethics Committee, the investigator shall not modify or deviate from the trial protocol, except for changes involving

only clinical trial management aspects such as eliminating immediate hazards to participants or changing monitors, telephone numbers, etc. The investigator or designated researchers shall record and explain deviations from the trial protocol. To eliminate immediate hazards to participants, if the investigator modifies or deviates from the trial protocol without obtaining the consent of the Ethics Committee, they shall report to the Ethics Committee and the Sponsor in a timely manner, explain the reasons, and report to the drug regulatory department if necessary.

The investigator shall take measures to avoid the use of concomitant medications prohibited by the trial protocol. The clinical trial institution and the principal investigator should take measures to note relevant clinical trial information on the participant's paper and electronic medical records, in addition to strengthening researcher training; they should also use information systems to impose necessary restrictions on prohibited drugs specified in the protocol and provide reminders for drugs to be used with caution. At the same time, inform participants that if they seek medical attention at other hospitals during the trial, they should inform the attending doctor of their participation in the clinical trial, especially their medication status.

For deviations from the trial protocol, standard operating procedures, and relevant laws and regulations, the monitor shall communicate with the investigator in a timely manner and take appropriate measures to prevent recurrence.

The Sponsor and/or the CRO entrusted by it are responsible for formulating, implementing, and updating SOPs for the clinical trial quality assurance and quality control systems in a timely manner to ensure that the conduct of the clinical trial and the generation, recording, and reporting of data all comply with the requirements of the trial protocol, GCP, and relevant laws and regulations. If it is found that personnel of the investigator, clinical trial institution, or Sponsor do not comply with the trial protocol, SOPs, GCP, or relevant laws and regulations during the clinical trial, which may have a significant impact on participant safety and rights or the reliability of clinical trial data, the Sponsor shall conduct a root cause analysis in a timely manner and take appropriate corrective and preventive measures. If the violation of the trial protocol or GCP is serious, the Sponsor may hold relevant personnel accountable and report to the drug regulatory department.

15 REPORTING AND PUBLISHING

15.1 Clinical Trial Report

Data and information collected during this trial will be used by Shenzhen Protgen Co., Ltd. or its authorized party to write a clinical trial report, which will be submitted to the signatory investigator for review and signature.

15.2 Confidentiality and Ownership of Trial Data

Any confidential information related to the investigational drug or this trial, including any data and results derived from this trial, is the proprietary property of Shenzhen Protgen Co., Ltd. The investigators and any other personnels participating in this trial should protect the confidentiality of such proprietary information belonging to Shenzhen Protgen Co., Ltd.

15.3 Publication and Public Disclosure

15.3.1 Publication policy

At the end of the trial, the investigators may collaborate with Shenzhen Progene Pharmaceutical Technology Co., Ltd. in writing one or more articles for publication, with both investigators and Shenzhen Progene Pharmaceutical Technology Co., Ltd. acting as first authors and corresponding authors.

Copyright determination is based on International Committee of Medical Journal Editors (ICMJE) standards (see http://www.ICMJE.org for the current official version). The total number of authors is based on the guidelines of the relevant journal or congress. In case of any discrepancies in the publication, the viewpoints of the investigator and Shenzhen Protgen Co., Ltd. will be represented fairly and comprehensively.

Any external contract research organization (CRO) or laboratory participating in the conduct of this trial has no right to the publication of this trial.

If the investigator wishes to independently publish/present any results regarding the trial, a draft manuscript/publication must be submitted in writing to Shenzhen Protgen Co., Ltd. for written comments prior to submission. Shenzhen Progene Pharmaceutical Technology Co., Ltd. will provide comments within 4 weeks of receipt of the draft manuscript. This statement does not

grant Shenzhen Protgen Co., Ltd. any editorial rights over the published content, except for limiting the disclosure of its intellectual property rights. If Shenzhen Progene Pharmaceutical Technology Co., Ltd. believes that the content of the publication is patentable, it is not permissible to present it in a scientific publication until the corresponding patent application has been published. Under such conditions, the publication will be modified or delayed at the investigator's discretion to allow sufficient time for Shenzhen Protgen Co., Ltd. to seek patent protection for the invention.

15.3.2 Public disclosure policy

ICMJE member journals have adopted the trial registration policy as the condition for publication. This policy requires all clinical studies to be registered online in an open clinical trial registration network. Therefore, Shenzhen Progene Pharmaceutical Technology Co., Ltd. will be responsible for registering this trial on the appropriate public registration network designated by the NMPA (i.e., http://www.chinadrugtrials.org.cn/).

16 ETHICAL CONSIDERATIONS AND INFORMED CONSENT

16.1 EC Review and Approval

The trial will be conducted in accordance with the trial protocol, GCP, and relevant laws and regulations. The sponsor shall obtain the approval of the EC before conducting the clinical trial. The sponsor should obtain from the investigator and clinical trial institution the name and address of the EC, the list of EC members participating in the project review, the review statement of compliance with GCP and relevant laws and regulations, as well as the documents and other relevant data reviewed and approved by the EC.

The documents that the EC shall review include the trial protocol and its amendments, ICF and its updates, participant recruitment methods and information, other written materials provided to the participants, Investigator's Brochure (IB), available safety information, a document containing information on participant compensation, certification of the qualifications of investigators, and any other documents that the EC may need to fulfil its responsibilities. The EC shall ensure that the ICF and other written materials provided to the participants explain the information on compensation to the participants, including the method, amount, and schedule

of compensation.

The review opinions of the EC may be "agree", "agree upon necessary modifications", "disagree", and "terminate or suspend the approved study". The review opinions should state what needs to be modified, or the reasons for denial.

If the review opinion of the EC is "agree upon necessary modifications", such as modifying the protocol, ICF, documents provided to participant, and/or other relevant documents, the sponsor should consult with the investigator and the clinical trial institution, modify relevant documents, and submit them to the EC. If the review opinion of the EC is "disagree", the sponsor and the investigator should modify the relevant issues of the clinical trial and submit them to the EC for re-review.

16.2 Informed Consent of Participants

Regarding informed consent, the investigator should adhere to the ethical principles of the Declaration of Helsinki and the following requirements:

1) The investigator should provide the participants with the latest informed consent and any additional information that have been agreed upon by the EC. During the clinical trial, the participants should sign the informed consent again, if necessary.
2) When obtaining informed consent from participants, investigators must comply with the regulatory requirements of drug regulatory authorities, GCP, and the ethical principles of the Declaration of Helsinki.
3) During the clinical trial, if there is any new information that may affect the participants' continuous participation in the trial, the investigator should promptly inform the participants or their guardian) and record accordingly.
4) The investigator should not use improper methods such as coercion or inducement to affect participants' participation or continuation of the clinical trial.
5) The investigator or designed study personnel should ensure that the participants fully understand all the matters concerned with the clinical trial, including the written information and the consent of the EC.
6) Oral and written materials provided to participants, provided to participants, such as ICFs, should be in plain language and presented in a manner that is easily understood by the participants or their guardians and witnesses.

7) Prior to the signing of the ICF, the investigator or designated study staff should provide the participants or their guardians with sufficient time and opportunity to know about details of the clinical trial and answer all the questions related to the trial raised by the participants or their guardians in detail.
8) The participant or his/her guardian, and the investigator implementing the informed consent should sign and date the ICF respectively. If the participant is unable to sign personally, the relationship between the participant and the individual signing on his/her behalf should be clearly stated.
9) If the participant or his/her guardian is illiterate, an impartial witness should be on site to witness the whole informed consent process. The investigator should thoroughly explain the content of the ICF and other text information to the participant or his/her guardian, and the witness. If the participant or his/her guardian provides oral consent to participate in the trial and can sign, every effort should be made to obtain their signature on the ICF. Additionally, the witness should sign and date the ICF to certify that the participant or his/her guardian has been fully explained the ICF and other text information by the investigator, understood the relevant content, and agreed to participate in the clinical trial.
10) The participant and his/her guardian should be provided with the original signed and dated ICF or a copy, along with other relevant written information, including the original updated ICF or a copy and any revised editions of other written information.
11) For a participant who has no capacity for civil conduct, the written informed consent should be obtained from his/her guardian; for a participant who has limited civil act capacity, written informed consent should be obtained from both the participant and his/her guardian. When the guardian consent on behalf of the participant, the guardian should inform the participant about the information regarding the clinical trial to the extent that the participant can understand, and try to get the participant to sign the ICF and indicate the date in person.
12) In case of emergency when the informed consent of the participant cannot be obtained before his/her participation in the clinical trial, the participant's guardian may give informed consent on behalf of the participant. If the guardian is also unavailable, the participant's inclusion mode should be clarified in the trial protocol and other documents, with the written consent of the ethics committee required. At the same time,

the informed consent of the participant or his/her guardian to continue participation should be obtained as soon as possible.

13) The specific time and personnel involved in the participant 's informed consent should be recorded in the medical history record.

17 LIABILITY AND INSURANCE

17.1 Participant Benefits and Privacy Protection

Participants in this trial will not experience direct health benefits, but the findings may be beneficial to patients later in life. All examinations or other trial-related operations are free of charge for the participants. Participants enrolled in this trial will be provided with transportation compensation for each visit to the hospital, and participants who have pharmacodynamic and immunogenicity-related blood samples collected will be given nutritional compensation. Transportation compensation and nutritional compensation will be paid to the participants in multiple installments based on their progress in completing the trial.

We will keep confidential all medical records and trial materials that can identify participants by name, in accordance with legal requirements. When reporting AEs in participants and other trial-related data, the investigator should use corresponding codes instead of the name of the participants so as to protect their privacy. The personal information of patients participating in the trial, including medical history and case records, will be strictly kept confidential. Within the scope permitted by laws and regulations, representatives of the sponsor, IEC members, and government regulatory authorities may review medical records, provided that confidentiality is not violated, to verify the authenticity, accuracy, and reliability of the study data.

17.2 Liability and Insurance

The responsibilities of Shenzhen Protgen Co., Ltd., its monitors, and investigators are consistent with GCP, relevant guidelines, and relevant regulatory requirements in China. The investigator shall perform the investigator's responsibilities as set out in the GCP and dispense the investigational products in accordance with the trial protocol approved by the EC or its signed amendment; the sponsor shall ensure that the investigation products are delivered to the investigator and the clinical trial institution in time, and that the investigational products are

safely stored and disposed of throughout the clinical trial.

Shenzhen Progene Pharmaceutical Technology Co., Ltd. has purchased clinical trial liability insurance for this clinical trial in accordance with the relevant Chinese laws. The cost of treatment for AEs related to the investigational drug or participation in the trial will be covered by the sponsor/insurer.

17.3 Documentation

17.3.1 Investigator documentation

The investigator and clinical trial institution should properly keep trial documents in accordance with the "Necessary Documents for the Clinical Trial" and requirements of drug regulatory departments. The sponsor should specify in the contract with the investigator and the clinical trial institution the storage time, cost, and handling of the necessary documents after expiration. The investigator shall complete and maintain a confidential participant identification code form. The participant identification code is a unique code assigned to participants in a clinical trial for identification. When reporting AEs in participants and other trial-related data, the investigator should use this code instead of the name of the participants so as to protect their privacy.

The trial documents shall not be destroyed without the written authorization of Shenzhen Protgen Co., Ltd. and the investigator. If the investigator retires or the trial unit is unable to continue to keep the trial documents, the trial documents may be transferred to a qualified third party for safekeeping.

17.3.2 Essential documents of clinical trial

Shenzhen Protgen Co., Ltd. will archive the essential documents of the clinical trial in accordance with GCP and applicable regulatory requirements.

17.4 Responsibilities of Each Party and Other Relevant Provisions

1) Sponsor

According to the GCP, the sponsor shall conscientiously perform the following duties:

- Responsible for initiating, managing, and providing funding for the clinical trial.
- The sponsor shall take protecting the rights and safety of participant s and the

authenticity and reliability of clinical trial results as basic considerations for the clinical trial.

- The sponsor shall select clinical trial institutions and investigators and confirm their qualifications and conditions to ensure the completion of the trial.
- Prior to the participation of all parties in the clinical trial, the sponsor shall clarify their responsibilities and indicate them in the signed contract.
- The contract signed by the sponsor, the investigator, and the clinical trial institution shall specify the responsibilities, rights, and interests of all parties, as well as the possible conflicts of interest that all parties should avoid. The study funds specified in the contract should be reasonable and conform to market rules. The sponsor, investigator, and clinical trial institutions shall sign and confirm the contract.
- When formulating the clinical trial protocol, the sponsor shall have sufficient safety and efficacy data to support the route, dose, and duration of administration. The sponsor shall promptly update the IB when important new information becomes available.
- Before the clinical trial, the sponsor shall submit relevant clinical trial materials to the drug regulatory authority and obtain permission for the clinical trial or complete filing. The submitted documents shall indicate the version number and date.
- The sponsor should obtain from the investigator and clinical trial institution the name and address of the EC, the list of EC members participating in the project review, the review statement of compliance with GCP and relevant laws and regulations, as well as the documents and other relevant data reviewed and approved by the EC.
- Sponsors shall select qualified biostatisticians, clinical pharmacologists, and clinicians to participate in the trial for designing the trial protocol and case report form, formulating a statistical analysis plan, analyzing data, and writing the final trial summary report.
- The sponsor shall provide investigators with investigational products that are easily identifiable, correctly coded, specially labeled, and of acceptable quality. Investigational products shall be properly packaged and stored according to the

requirements of the trial protocol. The sponsor shall establish a management system and record system for investigational products.

- The sponsor is responsible for the safety assessment of the investigational products during the trial. The sponsor shall promptly notify the investigator, clinical trial institutions, and drug regulatory authority of any issues identified during the clinical trial that may affect the safety of the participants, the implementation of the clinical trial, or the consent of the EC.
- The sponsor shall appoint a qualified CRA who is acceptable to the investigator.
- Sponsors and/or their contracted research organizations should establish quality control and quality assurance systems for clinical trials and may organize trial audits to ensure quality.
- The sponsor shall clearly define the authority to review the trial records. The sponsor shall clearly state in the trial protocol or contract that the investigator and clinical trial institution allow CRAs, auditors, reviewers of the EC, and inspectors of the drug regulatory authority to have direct access to source data and source documents related to the clinical trial. The sponsor should confirm that each participant has given written consent for the CRAs, auditors, ethics committee reviewers and regulatory authority inspectors to have direct access to his original medical records related to the clinical trial.
- The sponsor shall designate competent medical experts to provide timely consultation on relevant medical issues of the clinical trial.
- The sponsor shall report ADRs in accordance with the requirements and time limits.
- The sponsor shall immediately inform the investigator, clinical trial institution, and rug regulatory authority of the early termination or suspension of the clinical trial, and explain the reasons.
- The sponsor shall take appropriate measures to ensure that compensation or indemnity can be provided to the participants and investigators. The sponsor shall provide the investigators and the clinical trial institution with legal and economic insurance or guarantee related to the clinical trial, which shall match the nature and extent of the

risk of the clinical trial, excluding damages caused due to faults of the investigator and the clinical trial institution. The sponsor shall bear the medical expenses and corresponding compensation for the damage or death of the participants related to the clinical trial. The sponsor and the investigator shall promptly pay the compensation or indemnity to the participants. The sponsor shall provide compensation to the participants in a manner that complies with relevant laws and regulations. The sponsor shall provide investigational products to the participants free of charge and pay the medical test costs related to the clinical trial.

- The sponsor shall ensure compliance of the clinical trial. The sponsor shall terminate the continued participation of the investigator or the clinical trial institution in the trial after finding that they have non-compliance issues that are serious or have not been corrected after persuasion, and promptly report in writing to the drug regulatory authority. At the same time, the sponsor and the investigator shall take appropriate emergency safety measures to protect the safety and rights of participants.
- When the clinical trial is completed or terminated early, the sponsor shall submit a clinical trial report to the drug regulatory authority in accordance with relevant laws and regulations. The final clinical trial report shall comprehensively, completely, and accurately reflect the clinical trial results, and the safety and efficacy data in the report shall be consistent with the clinical trial source data.

2) Clinical trial institutions and investigators

According to the GCP, the investigator and the clinical trial institution shall conscientiously perform the following duties:

- The investigator and the clinical trial institution shall possess the qualifications required by GCP.
- The investigator and the clinical trial institution shall have the necessary conditions required to complete the clinical trial.
- The investigator shall provide participants with appropriate medical treatment.
- The investigator shall communicate with the EC.
- The investigator shall comply with the trial protocol.

- The investigator and the clinical trial institution are responsible for managing the investigational products provided by the sponsor.
- The investigator shall comply with the randomization procedure of the clinical trial.
- The investigator shall implement informed consent in accordance with the ethical principles of the Declaration of Helsinki and GCP requirements, and provide ICF and other materials.
- The records and reports of the trial shall comply with GCP requirements.
- The safety report of the investigator shall comply with GCP requirements.
- When the ethics committee decides to terminate a clinical trial early, the investigators should immediately report to the trial institution and the sponsor, and provide a detailed explanation in writing.
- When the clinical trial is terminated or suspended prematurely, the investigator shall notify the participants in a timely manner, provide them with appropriate treatment and follow-up, and complete other matters required by GCP.
- The investigator shall provide trial progress reports.

3) Biological specimen testing unit

The biological specimen testing unit shall carry out the biological specimen testing conscientiously in accordance with the requirements of the clinical trial. A bioanalytical laboratory in China will be responsible for testing and preserving the biological specimens, ensuring the reliability of the testing data, analyzing the data according to the trial results, and issuing reports.

4) Biostatistical analysis (data management/statistical unit)

The unit shall conscientiously fulfill its responsibilities for the management and statistics of the trial data in accordance with the GCP. At the end of the trial, the unit shall manage data, statistically analyze the data according to the established statistical plan, and issue a statistical analysis report.

17.5 Study Institutions and Participants

17.5.1 Sponsor

Sponsor Name: Shenzhen Protgen Co., Ltd.

Contact Person: An Yajun

Address: Room 1088, 10th Floor, Investment Building, Longcheng Street, Longgang District, Shenzhen, Guangdong Province, China

Telephone: 18310566756

E-mail: anyajun@protgen.com

17.5.2 Research unit

Main research unit: Beijing Friendship Hospital, Capital Medical University

Principal Investigator: Professor Jia Jidong

Address: No. 95, Yong'an Road, Xicheng District, Beijing, China

Telephone: 13501378269

E-mail: jiamd@263.com

17.5.3 Data management and statistical analysis unit

Company Name: Pharmaron (Chengdu) Clinical Research Service Co., Ltd.

17.5.4 Clinical audit units

Company Name: Pharmaron (Chengdu) Clinical Research Service Co., Ltd.

17.5.5 Biological sample testing unit

Company Name: United-Power Pharma Tech Co., Ltd.

18 REFERENCES

1. Moore, C. M. & Van Thiel, D. H. Cirrhotic ascites review: Pathophysiology, diagnosis and management. *World J. Hepatol.* **5**, 251–263 (2013).
2. Poordad, F. Presentation and complications associated with cirrhosis of the liver. *Curr. Med. Res. Opin.* **31**, 925–937 (2015).
3. Sarin, S. K. *et al.* Liver diseases in the Asia-Pacific region: a Lancet Gastroenterology & Hepatology Commission. *Lancet Gastroenterol. Hepatol.* **5,** 167–228 (2020).
4. Chinese Society of Infectious Diseases, Chinese Medical Association & Chinese Society of Hepatology, Chinese Medical Association. Guidelines for the prevention and treatment of chronic hepatitis B (version 2019). *J. Clin. Hepatol.* **35**, 2648–2669 (2019).
5. Chinese Society of Infectious Diseases, Chinese Medical Association & Chinese Society of Hepatology, Chinese Medical Association. Guidelines for the prevention and treatment of hepatitis C (2019 edition). *J. Clin. Hepatol.* **35**, 2670–2686 (2019).
6. Xiao, J. *et al.* Global liver disease burdens and research trends: Analysis from a Chinese perspective. *J. Hepatol.* **71,** 212–221 (2019).
7. European Association for the Study of the Liver. EASL clinical practice guidelines on the management of ascites, spontaneous bacterial peritonitis, and hepatorenal syndrome in cirrhosis. *J. Hepatol.* **53**, 397–417 (2010).
8. D'Amico, G., Garcia-Tsao, G. & Pagliaro, L. Natural history and prognostic indicators of survival in cirrhosis: A systematic review of 118 studies. *J. Hepatol.* **44**, 217–231 (2006).
9. Chinese Society of Hepatology, Chinese Medical Association. Guidelines for the diagnosis and treatment of cirrhotic ascites and related complications. *Pract. Hepatol. J.* **21**, 21–31 (2018).
10. Li, H. *et al.* Interpretation of the rapid recommendation guidelines for the use of plasma-derived human serum albumin in the treatment of liver cirrhosis. *J. Clin. Drug Ther.* **16**, 10–16 (2018).
11. Duan, H., Chai, J. & Deng, H. Functions of plasma-derived human serum albumin and its application in the treatment of critical illnesses. *PLA Med. J.* **37**, 926–929 (2012).
12. Rabbani, G. & Ahn, S. N. Structure, enzymatic activities, glycation and therapeutic potential of human serum albumin: A natural cargo. *Int. J. Biol. Macromol.* **123**, 979–990 (2019).
13. Chen, L. Systematic review of plasma-derived human serum albumin in patients with cirrhosis and ascites. PhD thesis, Hunan Normal Univ. (2014).

14. Caraceni, P. *et al.* Long-term albumin administration in decompensated cirrhosis (ANSWER): an open-label randomised trial. *Lancet* **391**, 2417–2429 (2018).
15. Caraceni, P. & Tufoni, M. On-treatment serum albumin level can guide long-term treatment in patients with cirrhosis and uncomplicated ascites. *J. Hepatol.* **74**, 340–349 (2021).
16. Center for Drug Evaluation, China Food and Drug Administration. *Instructions for Use: Plasma-derived Human Serum Albumin (Draft for Public Comment)* (2018).
17. Zhu, J. & Zhang, N. *Summary of Blood Product Batch Releases in 2021*. Zhongtai Securities Pharmaceutical and Biotechnology Research Report (2022).
18. Wang, M. & Tang, Z. A brief discussion on the progress of recombinant human serum albumin. *Biotechnol. World.* **4**, 116–117 (2015).
19. Wen, Z. *et al.* Research progress on recombinant human serum albumin. *China Med. Guide.* **9**, 222–224 (2011).
20. Mitsubishi Tanabe Pharma Corporation. *Withdrawal of Marketing Authorization for Recombinant Human Serum Albumin Preparation "Medway Injection 5%" and Voluntary Recall of "Medway Injection 5%" and "Medway Injection 25%"* (2009).
21. Li, C. *et al.* A randomized dose-escalation study on the safety, tolerability, immunogenicity, pharmacokinetics and pharmacodynamics of a novel recombinant human albumin in healthy subjects. *Eur. J. Pharm. Sci.* **165**, 105923 (2021).
22. Healthgen Biotechnology Corp. A study of OsrHSA in adult healthy male and female volunteers. *ClinicalTrials.gov* (2020).
23. Bernardi, M., Ricci, C. S. & Zaccherini, G. Role of human albumin in the management of complications of liver cirrhosis. *J. Clin. Exp. Hepatol.* **4**, 302–311 (2014).
24. Baxter AG. *Instructions for Use: Plasma-derived Human Serum Albumin* (Approval No. S20140025).
25. Mehta, C. R. & Pocock, S. J. Adaptive increase in sample size when interim results are promising: A practical guide with examples. *Stat. Med.* **30**, 3267–3284 (2011).

19 APPENDIX

19.1 Appendix I Instructions for Waist Circumference Measurement

Step 1: The participant lies flat after urinating;

Step 2: Take measurements while the participant is breathing calmly;

Step 3: The examiner measure the abdominal circumference by wrapping it snugly against the participant's skin instead of compressing it, circumferentially around the abdomen and passing through the navel for one full circle;

Step 4: Measure the values to the millimeter.

19.2 Appendix II Standard Procedures for Anthropometric Measurement

Height measurement:

- **Preparations:**

Instrument inspection: The column is perpendicular to the pedal and placed against the wall on a flat ground; the column is perpendicular to the sliding side plate and slides freely.

- **Test steps:**

1) Instruct the participant to remove shoes and take off headwear and hats (lighter headdresses worn for religious reasons may remain on, but the participant should wear them at each patient visit to measure weight);
2) Instruct the participant to stand on a calibrated height measuring board (range meter), or against a wall with heels together, toes apart at about 60 degrees, knees together and straight, and heels close to the backboard, range meter or wall;
3) Instruct the participant to stand at attention, look straight ahead, not raise their head, straighten their chest and pull in their abdomen, with their arms hanging naturally at their sides;
4) Instruct the participant to inhale and stand upright, with eyes looking straight ahead at eye level during measurement. Ensure the participant's lower edge of the orbit and upper edge of the earlobe at the same level. The participant's heels, buttocks, and the area between the shoulder blades should all touch the upright column at the same time, with the head remaining in an upright position.

- **Precautions:**

1) The measurer holds the sliding plate and gently slides it downwards until the bottom surface contacts the top of the skull; after observing and confirming that the posture is correct, the measurer reads the number shown on the ground column of the sliding plate;
2) When taking the reading, the measurer should keep his eyes on the same horizontal plane as the sliding plate
3) Record the results in centimeters, to one decimal place (it is recommended to keep them consistent with the data entered into the system).

Body weight measurement:

- **Weighing scale placement requirements:**

Quiet and spacious, with a level and solid surface.

- **Measurement steps:**

1) Instruct the patient to remove their shoes and headwear (lighter headwear worn for religious reasons may remain on, but the patient should wear it during each visit to have their weight measured);
2) Make sure to place the scale on a solid, flat surface (do not place it on a carpet, or on a sloping or rough surface);
3) Instruct the patient to stand calmly on the scale and step up and down gently. Place both feet symmetrically on either side of the scale, with the body upright, arms hanging naturally and relaxed at the sides of the body, head upright, and eyes looking straight ahead;
4) After the scale reading stabilizes, the measurer instructs the participant to remain upright and records the reading (to one decimal place). The unit is kilograms (kg).

- **Precautions:**

1) Each visit should be conducted after the participant has urinated, using the same scale calibrated for subsequent measurements of the participant's total weight;
2) During weight measurement, the patient should remove their shoes, hat, and coat, wear only a single layer of clothing, and take out their personal belongings, such as wallet and mobile phone. Each time the body weight is measured, the participant

should wear similar clothing each time.
