# Supplementary material for: A randomized, double-blind, active-controlled phase 3 multicenter trial of rHSA for cirrhotic ascites
Source: Signal Transduct Target Ther. 2026 Sep 10;11:390. doi: 10.1038/s41392-026-02951-7 (PMC13588796; doi:10.1038/s41392-026-02951-7)
Supplement: Supplementary file 2 [file 41392_2026_2951_MOESM3_ESM.pdf]

Statistical Analysis Plan (Phase 3)

A Randomized, Double-Blind, Parallel-Group, Phase 2/3 Clinical Trial Evaluating the Efficacy and Safety of Recombinant Human Serum Albumin compared to Human Serum Albumin in Patients with Cirrhotic Ascites

rHSA 2020-3

Investigational drug:	Recombinant Human Serum Albumin Injection
Comparator:	Human Serum Albumin Injection
Indications:	Cirrhotic Ascites
Study phase:	Phase 3
File type:	Statistical Analysis Plan (Phase 3)
Version:	V1.0
Release date:	9 July 2025

Table of contents

1. Overview of the Trial....5
1.1 Purpose of the trial....5
1.1.1 Primary Objective....5
1.1.2 Secondary objectives....5
1.1.3 Exploratory Objective....5
1.2 Trial endpoints....5
1.2.1 Efficacy evaluation endpoints....5
1.2.2 Safety....7
1.2.3 Pharmacodynamic (PD)....7
1.2.4 Immunogenicity....7
1.3 Study Design....7
1.3.1 Overall Study Design....7
1.3.2 Sample Size Determination....15
1.3.3 Randomization and blinding....15
2 Estimands....16
2.1 Primary Estimand....16
2.3 Other efficacy indicators....18
3 General Considerations for Statistical Analysis....18
3.1 General Principles....18
3.2 Analysis Populations....20
3.3 Multicenter Studies....20
3.4 Covariate Adjustment....20
3.5 Multiplicity....20
3.6 Subgroup Analysis....21
4 Data Processing Rules....21
4.1 Derivative Variables....21
4.1.1 Definition of the Baseline and Change from Baseline....21
4.1.2 Definition of Baseline for Re-treatment and Change from Baseline for Re-treatment....21
4.1.3 Treatment Day....22
4.1.4 Re-treatment Day....22
4.2 Missing Data....22
4.2.1 Missing or Incomplete Dates....22
4.3 Visit Windows....23
5 Statistical Analysis....27
5.1 Study Participants....27
5.1.1 Participant Disposition....27
5.1.2 Protocol Deviations....27
5.1.3 Demographic and Baseline Characteristics....28
5.1.4 Medical History and Surgical History....30
5.1.5 Previous and Concomitant Medications....30
5.1.6 Prior and Concomitant Non-Drug Therapies....31

5.2 Efficacy Analysis 31
5.2.1 Analysis of the Primary Esitmand 31
5.2.2 Key Secondary Efficacy Endpoint Analysis..33
5.2.3 Secondary Efficacy Endpoint Analysis 35
5.2.4 Exploratory Analysis 41
5.3 Safety Analysis 41
5.3.1 Extent of Drug Exposure and Compliance 41
5.3.2 Adverse Events 42
5.3.3 Laboratory Assessments 45
5.3.4 Vital Signs...46
5.3.5 12-Lead Electrocardiogram (ECG)..46
5.3.6 Physical Examination...47
5.3.7 Fundus examination 47
5.3.8 Thyroid function test..47
5.3.9 Thyroid Ultrasonography..48
5.3.10 Iodine Uptake Test..48
5.3.11 Other Safety Assessments...48
5.4 PD Analysis 48
5.5 Immunogenicity Analysis 49
6 Planned Analyses 49
6.1 IDMC 49
6.2 Final Analysis 49
7 Deviations from Planned Analyses 49
8 Templates for Statistical Tables, Listings, and Figures 50

List of Abbreviations

Abbreviation	Explanation
ADA	Anti-drug Antibody
AE	Adverse Events
ANCOVA	Analysis of Covariance
ANOVA	Analysis of Variance
ATC	Anatomical Therapeutic Chemical
BMI	Body mass Index
BQL	Below the Quantization Limit
CI	Confidence Interval
COP	Plasma Colloid Osmotic Pressure
CRF	Case Report Form
CTCAE	Common Terminology Criteria for Adverse Events
CV	Coefficient of Variation
ECG	12-Lead Electrocardiogram
eCRF	Electronic Case Report Form
FAS	Full Analysis Set
FCS	Full Compliance Set
HCP	Host Cell Protein
HR	Hazard Ratio
IDMC	Independent Data Monitoring Committee
IWRS	Interactive Web Response System
LSM	Least Squares Mean
MAR	Missing at Random
MedDRA	Medical Dictionary for Regulatory Activities
MMRM	Repeated measurement linear mixed-effect model
PD	Pharmacodynamics
PPS	Per-Protocol Set
PT	Preferred Term
Q1	First Quarter
Q3	Third Quartile
RD	Retreatment
rHSA	Recombinant Human Serum Albumin
SAE	Serious Adverse Events
SAP	Statistical Analysis Plan
SE	Standard Error
SOC	Classification of System Organs
SS	Safety Set
TEAE	Treatment Emergent Adverse Event
TRAE	Treatment-Related Adverse Event

1. Overview of the Trial

This Statistical Analysis Plan (SAP) has been developed for the Phase 3 study in accordance with the clinical trial titled *"A Randomized, Double-Blind, Parallel-Group, Phase 2/3 Clinical Trial Evaluating the Efficacy and Safety of Recombinant Human Serum Albumin Versus Human Serum Albumin in Patients with Cirrhotic Ascites"* (Protocol No.: rHSA 2020-3), sponsored by Shenzhen Protgen Co., Ltd. This document provides a detailed description of the statistical analysis methods and data processing principles for the phase 3 component of the study.

The preparation of this SAP is based on Clinical Trial Protocol rHSA 2020-3 **(Version 3.1, dated April 15, 2025).**

1.1 Purpose of the trial

1.1.1 Primary Objective

To evaluate the efficacy of recombinant human serum albumin (rHSA) in patients with cirrhotic ascites, as measured by the change from baseline in serum albumin level immediately after the final intravenous administration.

1.1.2 Secondary objectives

To evaluate the efficacy of recombinant human serum albumin in patients with cirrhotic ascites based on additional efficacy endpoints;

To evaluate the safety of recombinant human serum albumin in patients with cirrhotic ascites;

To evaluate the pharmacodynamic of recombinant human serum albumin in patients with cirrhotic ascites;

To evaluate the immunogenicity of recombinant human serum albumin in patients with cirrhotic ascites.

1.1.3 Exploratory Objective

To evaluate the effect of recombinant human serum albumin on glycated albumin and glycated hemoglobin in patients with cirrhotic ascites.

1.2 Trial endpoints

1.2.1 Efficacy evaluation endpoints

1.2.1.1 Primary endpoints

The change in serum albumin levels measured at the central laboratory from baseline to immediately after the final intravenous administration.

1.2.1.2 Key secondary endpoint

The rate of ascites improvement after the final administration. Ascites improvement was defined using grade-specific criteria: for patients with grade 1 ascites, complete resolution of ascites; for those with grade 2, reduction in ascites grade or a decrease ≥ 25% and > 1 cm in maximum depth of ascitic fluid.

1.2.1.3 Secondary endpoints

- Change from baseline in serum albumin level, as confirmed by local laboratory albumin assessment immediately following the completion of the final intravenous infusion;
- Change from baseline in ascites depth at the completion of the final intravenous infusion;
- Proportion of participants achieving serum albumin level ≥ 35 g/L, as confirmed by albumin assessment at the completion of the final intravenous infusion;
- Time to achieve serum albumin level ≥ 35 g/L;
- Change from baseline in abdominal circumference at the completion of the final intravenous infusion;
- Change from baseline in body weight at the completion of the final intravenous infusion.

For patients receiving re-treatment with investigational medicinal product from Day 29 to Day 76, the following efficacy endpoints will also be assessed:

- Change from retreatment baseline in serum albumin level, as confirmed by albumin assessment immediately following the completion of the final intravenous infusion during the re-treatment period;
- Ascites improvement rate at the completion of the final intravenous infusion during the re-treatment period, with ascites improvement defined as: resolution of ascites for participants with Grade 1 ascites at re-treatment baseline, or downgrading of ascites or ≥ 25% reduction in ascites depth measurement with absolute decrease > 1 cm for participants with Grade 2 ascites at re-treatment baseline;
- Change from re-treatment baseline in ascites depth at the completion of the final intravenous infusion during the re-treatment period;
- Proportion of participants achieving serum albumin level ≥ 35 g/L, as confirmed by albumin assessment during the re-treatment period;
- Time to achieve serum albumin level ≥ 35 g/L during the re-treatment period;
- Change from re-treatment baseline in abdominal circumference at the completion of the final intravenous infusion during the re-treatment period;
- Change from re-treatment baseline in body weight at the completion of the final intravenous infusion during the re-treatment period.

1.2.1.4 Exploratory endpoint

Changes in glycated albumin and glycated hemoglobin from baseline after drug administration.

1.2.2 Safety

Safety will be assessed by monitoring the incidence and severity of adverse events (AEs), graded according to the National Cancer Institute Common Terminology Criteria for Adverse Events (NCI-CTCAE) Version 5.0, and investigator-assessed causality relative to the investigational product. Assessments will include laboratory evaluations (hematology, serum chemistry, urinalysis, and coagulation parameters), vital signs, 12-lead electrocardiogram (ECG), physical examination, and thyroid function tests (TFTs) at predefined timepoints. Additional assessments include thyroid ultrasound and radioactive iodine uptake (RAIU) testing, as well as ophthalmologic examination (funduscopy).

Specific safety endpoints include:

- Incidence of treatment-emergent adverse events (TEAEs)
- Clinically significant changes in vital signs or physical examination findings
- Clinically significant laboratory abnormalities

1.2.3 Pharmacodynamic (PD)

The pharmacodynamic (PD) endpoint in this trial is plasma colloid osmotic pressure (COP). PD characteristics will be evaluated by assessing plasma COP and its change from baseline following intravenous administration of the investigational product.

1.2.4 Immunogenicity

The immunogenicity endpoints in this trial are the incidence and titers (if applicable) of anti-drug antibodies (ADAs) against rHSA and host cell proteins (HCP). Immunogenicity will be evaluated by assessing the incidence and titers (if applicable) of ADAs following administration of the investigational product.

1.3 Study Design

1.3.1 Overall Study Design

This study is a randomized, double-blind, active-controlled, parallel-group phase 2/3 clinical trial of Recombinant Human Serum Albumin in patients with cirrhotic ascites. This study consists of two parts: phase 2 and phase 3. In the phase 2, pHSA will be used as a comparator to evaluate the efficacy of rHSA in increasing serum albumin levels in patients with cirrhotic ascites at different doses and treatment courses, and to establish the dose–response relationship, thereby providing a basis for the dosing regimen and sample size calculation for the phase 3 study. In the phase 3, the change from baseline in serum albumin level immediately after completion of the final intravenous administration will be used as the primary endpoint to evaluate the efficacy of rHSA, and its safety, pharmacodynamic (PD) profile, and immunogenicity will be further evaluated. This SAP applies to phase 3 only.

In phase 3, the change in serum albumin level from baseline immediately after the final intravenous administration (based on central laboratory tests) will be evaluated as the primary efficacy endpoint to verify the equivalence hypothesis between the investigational drug and the comparator drug. In phase 3, the clinical benefit of albumin supplementation in participants will also be assessed based on ascites improvement. At the individual level, ascites improvement is defined as: resolution of ascites in participants with Grade 1 ascites at baseline; or downgrading of ascites, or a ≥25% reduction in ascitic fluid depth from baseline with a decrease of >1 cm, in participants with Grade 2 ascites at baseline. The ascites improvement rate at the time of completion of the final intravenous administration (i.e., the proportion of participants in each group meeting the criteria for ascites improvement) will be used as a key secondary efficacy endpoint for non-inferiority testing, to further evaluate the efficacy of the investigational drug (rHSA) in patients with cirrhotic ascites. The results from phase 2 indicated that the change from baseline in serum albumin level and ascitic fluid depth at the time of completion of the final intravenous administration may be influenced by serum albumin level at baseline, grade of ascites, and diuretic use. Therefore, in phase 3, the baseline serum albumin level (<25 g/L or ≥25 g/L), baseline ascitic fluid depth (< 3 cm; 3-6 cm [inclusive of 3, exclusive of 6]; 6-10 cm [inclusive of 6 and 10]), and baseline diuretic use (yes; no) will be used as stratification factors. In phase 3, a total of 388 participants are planned to be enrolled and randomly assigned in a 1:1 ratio to the following two groups (194 participants per group).

- Group A: Investigational drug 20 g/day, administered continuously for 7 days;
- Group B: Comparator drug 20 g/day, administered continuously for 7 days.

The phase 3 study includes a screening period, a treatment period, and a follow-up period. Participants will enter the follow-up period after completing 7 consecutive days of dosing and undergo the final visit on D90±7 during the follow-up period. If a participant's serum albumin level drops to <30 g/L during the follow-up period (D29 to D76), accompanied by Grade 2 ascites or judged by the investigator as requiring re-supplementation with human serum albumin, re-treatment will be administered. The participant will remain blinded and re-treated with the investigational product (rHSA or pHSA) according to their randomization at the time of initial treatment, with a dosing regimen of 20 g/day, administered intravenously once daily for 7 days or until serum albumin ≥35 g/L (whichever comes first). If a participant undergoes re-treatment, relevant safety and efficacy tests should be completed during the re-treatment period (i.e., the period of re-dosing, not exceeding 7 days), including on day 1 of re-treatment (RD1), RD3 (if dosing is received on the same day), RD7 (if dosing is received on the same day), or the last day of re-treatment. Visits during the re-treatment period are conducted independently and do not affect the visits scheduled for the follow-up period. All participants received their last visit on D90±7.

Blood samples for PD and immunogenicity assessments will be collected at specified timepoints during the trial.

Detailed study flowcharts are presented in Table 1 and Table 2.

Table 1 Trial Flow chart (Phase 3, Group A and Group B)

Study day/procedures	Screening period	Treatment period (D1-D7)				Follow-up Period (D8~D90)				Early Discontinuation Visit [N]
	D-28~D-1	D1	D2	D3	D7/EOT[M]	D14	D29	D57	D90	
**Window**	/	/	/	/	/	/	± 3 days	±5 days	± 7 days	+7
**Visit**	1	2	3	4	5	6	7	8	9	/
Signing of the Informed Consent Form	X									
Demographic information collection (including height, weight, etc.)	X									
Medical history collection	X									
Treatment History Collection	X									
Inclusion/Exclusion Criteria Assessment	X									
Infectious Disease Screening [A]	X[C]									
Pregnancy Test (Females of Childbearing Potential Only)	X[C]								X	X
Participant Randomization [B]	X									
Investigational product Dosing		X[E]								
Albumin Assessment [D]	X[C]	X[H]								
vital signs	X	X[F]				X	X	X	X	X
Waist circumference and weight measurement		X[G]					X	X	X	X
Ultrasound abdomen (ascites depth assessment)	X[C]			X	X		X	X	X	X
Physical examinations	X		X		X	X	X	X	X	X
Hematology [I]	X[C]		X	X	X	X	X	X	X	X
Blood Chemistry [I]	X[C]		X	X	X[G]	X	X	X	X	X
Coagulation Assay [I]	X[C]			X	X		X	X	X	X
Hemoglobin A1c (HbA1c) test		X[C]					X		X	X
Glycated Albumin test		X[C]			X					
Urinalysis [I]	X[C]			X	X		X	X	X	X
Stool routine [I]	X[C]									
12-lead ECG	X[C]			X	X		X	X	X	X

Study day/procedures	Screening period	Treatment period (D1-D7)				Follow-up Period (D8~D90)				Early Discontinuation Visit [N]
	D-28~D-1	D1	D2	D3	D7/EOT[M]	D14	D29	D57	D90	
**Window**	/	/	/	/	/	/	± 3 days	±5 days	± 7 days	+7
**Visit**	1	2	3	4	5	6	7	8	9	/
ECG	X[C]									
Fundus examination	X[C]								X	X
Thyroid function tests [J]	X[C]				X		X	X	X	X
Thyroid Ultrasound	X[C]				X		X	X	X	X
Iodine intake test [O]	X[O]				X[O]		X[O]	X[O]	X[O]	X[O]
PD blood sampling [k]		X			X					
ADA blood sample sampling [L]		X[L]							X[L]	X[L]
Judgment of retreatment [P]								X		
Recording of concomitant medications	X					X[P]				X
Record of adverse events	X					X				X

Note:

A. All participants undergoing screening shall undergo infectious disease screening during the screening period. The assessments include hepatitis B panel (HBsAg, HBsAb, HBeAg, HBcAb, HBeAb; not required for participants with a documented history of hepatitis B), quantitative HBV DNA (to be performed only in participants positive for hepatitis B surface antigen or with a documented history of hepatitis B), HCV antibody testing (not required for participants with a documented history of hepatitis C), HCV RNA testing (to be performed only in participants positive for HCV antibody or with a documented history of hepatitis C), HIV antibody testing, and syphilis testing.

B. On Day –1, the inclusion/exclusion criteria will be reviewed. Participants who meet the eligibility requirements will be randomized using stratified block randomization.

C. Abdominal ultrasound, hematology, blood chemistry, coagulation function, urinalysis, stool routine, 12-lead electrocardiogram, pregnancy test, albumin test, and echocardiogram during the screening period are acceptable if the results are within one week before the first intravenous administration of the investigation product (including results from before signing the informed consent form to one week before the first administration of the investigational drug). Otherwise, the tests need to be repeated within one week before administration. Other safety assessments (including vital signs, physical examination, infectious disease screening [HBsAg, HBsAb, HBeAg, HBcAb, HBeAb, HBV DNA quantification, HCV antibody, HCV RNA, HIV antibody, and syphilis testing], fundi examination, thyroid function tests, and ultrasound thyroid) may use results obtained within 28 days prior to the first intravenous administration of the investigational product (including results obtained before signing the informed consent form up to 28 days prior to first dosing). Glycated hemoglobin (GHbA1c) testing (within 7 days prior to administration) and baseline GHbA1c testing were performed before the first administration on day 1 of treatment.

D. During the screening period, albumin (ALB) levels will be monitored. If the monitored ALB value is <30 g/L, the participant meets the enrollment criteria. After entering the treatment period, participants will no longer be evaluated against inclusion or exclusion criteria. Therefore, if a participant has an albumin level ≥30 g/L prior to dosing on D1, this does not constitute a screening failure, and the participant shall enter the treatment period and receive dosing. During the treatment period, starting from D1, samples for albumin testing will be collected within 1 hour prior to each intravenous administration and immediately after completion of the final intravenous administration (+3 minutes). In addition to albumin testing in local laboratories, samples need to be collected before administration on D1, immediately after administration on D7, or during an EOT visit and sent to the central laboratory for albumin testing. In the event that a study site does not offer a individual albumin test, the investigator may select an alternative laboratory panel that includes albumin assessment.

E. All randomized participants will receive intravenous administration of the investigational drug or comparator at the specified dose, once daily for 7 consecutive days. During infusion, special attention should be paid to whether the participant develops infusion-related adverse reactions or clinical symptoms and manifestations related to cardiovascular overload (such as headache, carotid artery engorgement, dyspnea, etc.), and the infusion should be suspended or stopped based on the discontinuation criteria and the actual situation.

F. On the day of each infusion of the investigational drug, vital signs monitoring must be performed during the infusion. Specifically, the monitoring should be performed once within 2 hours (h) before the infusion and once 30 minutes (+30 minutes, excluding 30 minutes) after the infusion is completed.

G. Prior to the first administration of the investigational product on D1, abdominal circumference and body weight shall be measured three times, with an interval of more than 1 minute between measurements, and the results shall be recorded as baseline values. During the treatment period, abdominal circumference and body weight measurements shall be performed after completion of each intravenous administration. For participants who do not complete the 7-day treatment period (including those who meet the criteria for treatment discontinuation or who terminate treatment early), abdominal circumference and body weight measurements shall be performed at the D7 visit.

H. Eligibility for dosing during the treatment period shall be determined based on albumin (ALB) test results. If ALB < 40 g/L, the participant shall receive the planned intravenous administration; otherwise, dosing shall not be performed. It is not necessary to wait for the albumin test result obtained on the same day to determine whether treatment discontinuation criteria have been met before deciding whether to administer the dose on that day. Once it is known that the participant's serum albumin level has reached the treatment discontinuation criterion, no further dosing shall be administered. If the investigator determines that the albumin test result is abnormal (potentially due to systematic instrument error, sampling error, or transient increases or decreases in albumin levels related to clinical signs or disease conditions), one repeat test is permitted, and the repeat result shall prevail.

I. During the screening period, if a laboratory test value (except for albumin) exceeds the exclusion criteria, but the investigator determines that the abnormality may be related to systematic instrument error or sampling error (e.g., the patient did not fast as required on the day of the test), a retest is allowed. If the retest value still exceeds the exclusion criteria, the participant does not meet the inclusion criteria for this trial and fails the screening. Depending on the investigator's judgment, the participant may participate in the screening again after receiving certain symptomatic treatment.

J. Thyroid function assessments primarily include thyroxine (T4), triiodothyronine (T3), thyroid-stimulating hormone (TSH), free T3, and free T4.

K. This study will involve multiple blood sample collections for PD. For details on the specific sample collection time points and time window settings.

L. Immunogenic blood samples were collected at the following times: D1 before the start of intravenous administration of the investigational drug, D90, and early discontinuation from the visit (if applicable). The sampling time windows shall be consistent with the corresponding visit time windows.

M. If a participant discontinues treatment prematurely during the treatment period, an End of Treatment (EOT) visit shall be completed within 24 hours after the investigator's decision to discontinue treatment. The assessments and procedures at the EOT visit shall be the same as those at the D7 visit. participants who complete the EOT visit are not required to undergo duplicate assessments at D7.

N. For all randomized participants who have received at least one intravenous administration of the investigational product, if the participant withdraws from the study prematurely, an early discontinuation visit shall be conducted within 7 days after the decision to withdraw. Based on the investigator's judgment, assessment results obtained within 3 days prior to the decision to withdraw may be used as early discontinuation visit data, and repeat assessments are not required.

O. Iodine uptake testing is conducted only when the investigator deems it necessary, and the specific testing methods (such as thyroid 131I uptake test, serum iodine and 24-hour urinary iodine) are determined by the investigator based on the actual situation of the center. Iodine uptake test may be omitted if no adverse thyroid events occur in the participant or if the investigator determines that it is unsuitable and/or unnecessary to conduct the test. If our site is unable to perform iodine intake testing, we can accept testing from other hospitals.

P. During the phase 3 follow-up period (D29–D76), if a participant's serum albumin level drops to <30 g/L and is accompanied by grade 2 ascites, or if the investigator determines that re-supplementation of pHSA is necessary, the participant may be allowed to receive retreatment with the investigational drug (rHSA or pHSA) in a blinded manner, based on the randomization assigned during the initial treatment. The dosing regimen is 20 g/day, administered intravenously once daily for 7 consecutive days, or until serum albumin ≥35 g/L (whichever occurs first). The retreatment visit is conducted independently and does not affect the original scheduled follow-up visit. If the retreatment visit overlaps with the original scheduled follow-up visit, the same examination will not be repeated. For details, please refer to the retreatment visit flowchart.

Table 2 Trial Flow chart (Phase 3 - Re-treatment period during3 follow-up)

Study day/procedures	Retreatment period (D29~D76) [A]		
	Day 1 of retreatment (RD1)	Day 3 of retreatment (RD3)	Day 7 of retreatment (RD7) /Last day of retreatment [H]
**Window**	/	/	/
Albumin test [B]	X		
Vital signs [C]	X		
Waist circumference and weight measurement [D]	X		
Abdominal ultrasound examination (ascites depth assessment) [F]	X	X	X[E]
Physical examinations		X[E]	X[E]
Hematology		X[E]	X[E]
Blood chemistry		X[E]	X[E]
Coagulation function test		X[E]	X[E]
Urinalysis		X[E]	X[E]
12-lead ECG		X[E]	X[E]
Dosage of investigation product [G]	X		
Recording of concomitant medications	X		
Record of adverse events	X		

Note:

A. If a participant's serum albumin level drops to <30 g/L during the follow-up period, and is accompanied by grade 2 ascites or if the investigator determines that pHSA needs to be supplemented again, the participant may be allowed to receive the investigation product (rHSA or pHSA) again during the follow-up period from D29 to D76, based on the randomization results at the time of the first treatment. Retreatment visits are conducted independently and do not affect the scheduled follow-up visits. If a retreatment visit overlaps with a scheduled follow-up visit, the same tests will not be repeated.

B. During the retreatment period, participants should have their albumin levels checked 1 hour before each administration of the investigation product (including before administration of RD1, which serves as the baseline for albumin changes during the retreatment period) and immediately after the final intravenous administration. In the event that a study site does not offer a individual albumin test, the investigator may select an alternative laboratory panel that includes albumin assessment. If the serum albumin level is ≥35 g/L, the medication will not be continued (it is not necessary to wait for the albumin test results of the day to determine whether the discontinuation criteria have been met before deciding whether to administer the medication that day; once it is known that the

participant's serum albumin level has reached the discontinuation criteria, the medication will not be continued). Participants who meet the criteria for early discontinuation of medication and no longer wish to continue taking the medication do not need to undergo albumin testing after discontinuation.

C. On each day of investigational product infusion, vital signs shall be monitored during the infusion period, with assessments conducted once within 2 hours prior to infusion and once at 30 minutes after completion of infusion (+30 minutes). Participants who meet the criteria for early discontinuation of medication and no longer require vital sign monitoring during the retreatment treatment period after discontinuation of medication.

D. Before administering RD1 during the second treatment period, participants should have their waist circumference and weight measured three times, and the average of the three measurements should be recorded as the baseline value for the retreatment. During the retreatment period, starting from RD2, after each intravenous administration, abdominal circumference and weight should be measured 3 times, and the average of the 3 measurements should be recorded. The measurement requirements are the same as during the treatment period. Participants who meet the criteria for early discontinuation of medication and no longer require weight and abdominal circumference measurements during the retreatment period after discontinuation of medication.

E. During the retreatment period, physical examination, hematology, blood chemistry, coagulation function tests, urinalysis, and 12-lead electrocardiogram should be performed at RD3, RD7, or the last day of retreatment. The above safety tests during the RD3 and RD7 visits are only applicable to participants who received the investigation product on that day. Participants who meet the criteria for early discontinuation and do not continue medication will have a retreatment end visit within 24 hours after early discontinuation (as close as possible to the time of completion of the last dose). It is not necessary to continue physical examination, hematology, blood chemistry, coagulation function tests, urinalysis, and 12-lead electrocardiogram during the retreatment period after discontinuation. Investigators determined that the results of examinations conducted within one day prior to the end of treatment could be recorded as the results of follow-up visits after retreatment, and that repeat examinations were not necessary.

F. Abdominal ultrasound examinations will be performed before the first dose of RD1 (baseline), RD3, RD7, or the last day of retreatment. Abdominal ultrasound examinations during RD3 and RD7 visits are only applicable to participants who receive medication on the same day (participants who do not receive medication on the same day do not need to undergo this examination), and must be performed after medication is administered. The time window for abdominal ultrasound examinations during the retreatment period is +1 day. If a retreatment visit overlaps with a follow-up visit, the same tests will not be repeated.

G. Participants will be blinded and re-treated with the investigation product (rHSA or pHSA) according to their randomization at the time of initial treatment, with a dosing regimen of 20 g/day, administered intravenously once daily for 7 days or until serum albumin ≥35 g/L (whichever comes first).

H. Participants need to have an end-of-treatment (EOT) visit on the day of the last dose during the retreatment period. If the participant takes the medication as planned for 7 days, the visit should be conducted on the 7th day of the retreatment period. If the participant discontinues the medication early during the retreatment period, the visit should be conducted within 24 hours of the early discontinuation (as close as possible to the time of the last dose completion). No other visits/procedures are required during the retreatment period.

1.3.2 Sample Size Determination

An equivalence hypothesis test will be employed for the phase 3 stage. The primary efficacy endpoint is the change from baseline in serum albumin level (as determined by the central laboratory) immediately after the completion of the final intravenous administration, to verify the clinical equivalence of investigational drug (rHSA) to the comparator product (pHSA). Based on phase 3 clinical trial designs of similar products both domestically and internationally, and expert recommendations, the equivalence margin is set at ± 2 g/L.

Based on prior experience with similar products and phase 2 data of the investigational product, the standard deviation of the primary efficacy endpoint is expected not to exceed 5 g/L for both the investigational and reference products, and the efficacy of the investigational product is anticipated to be similar to that of the comparator. Under a two one-sided tests procedure at a one-sided significance level of 0.025, a sample size of 328 participants (164 per group) will provide 90% power.

In phase 3 of this study, the ascites improvement rate at completion of the final intravenous administration will be used as a key secondary efficacy endpoint, with a non-inferiority assessment conducted. Ascites improvement at the individual level is defined as: resolution of ascites in participants with Grade 1 ascites at baseline; or downgrading of ascites grade or a reduction in measured ascites depth of ≥ 25% from baseline and an absolute decrease of >1 cm in participants with Grade 2 ascites at baseline. The ascites improvement rate is defined as the proportion of participants in the investigational drug group or the comparator group who meet the predefined criteria for ascites improvement. At a one-sided 0.025 significance level, -10% is proposed as the non-inferiority margin.

Results from the phase 2 study of the investigational drug showed that the ascites improvement rates at completion of the final intravenous administration were 60.87% and 50.00% in the 20 g/day investigational drug group and the 20 g/day comparator group, respectively. The between-group difference in ascites improvement rate (investigational drug group minus comparator group; without adjustment for covariates, using Fisher's exact test) was 10.9%. When adjusted for baseline ALB levels (< 25 g/L; ≥ 25 g/L), baseline ascites depth (< 3 cm; 3–6 cm [including 3, excluding 6]; 6–10 cm [including both 6 and 10]), and baseline use of diuretics (yes; no) as covariates, the between-group difference in ascites improvement rate (investigational drug group minus comparator group) was 5.8%.

Therefore, considering the results from similar products and the phase 2 study of the investigational product, an ascites improvement rate of 50% is assumed in the comparator group, with an estimated between-group difference (rHSA – pHSA) of 5%. A sample size of 348 participants will provide 80% power. Accounting for a 10% dropout rate, a total of 388 participants (194 per group) is planned for enrollment.

1.3.3 Randomization and blinding

Based on the Phase 2 study results and investigator input, participants enrolled during the screening period of the Phase 3 study will be stratified by baseline human serum albumin level (< 25 g/L; ≥ 25 g/L), baseline ascites depth (< 3 cm; 3–6 cm [including 3, excluding 6]; 6–10 cm [including 6 and 10]),

and baseline diuretic use (yes; no), and randomly assigned in a 1:1 ratio to one of two treatment groups (194 participants per group).

This study is conducted as a double-blind trial. In accordance with standard operating procedures for double-blind clinical trials, appropriate measures will be implemented to ensure that the investigational treatments remain indistinguishable. Both participants and study personnel will remain blinded to treatment assignment until formal unblinding occurs upon study completion.

2 Estimands

2.1 Primary Estimand

Clinical Question of Primary Interest: Whether the change from baseline in serum albumin level immediately after completion of the last final intravenous administration of rHSA (based on central laboratory test results) is equivalent to that of plasma-derived human serum albumin (pHSA) in patients with cirrhotic ascites and hypoalbuminemia.

Attributes of the Primary Estimand:

- **Target Population:** Patients with cirrhotic ascites and hypoproteinemia who meet the inclusion criteria and none of the exclusion criteria.
- **Target Variable:** Change from baseline in serum albumin level immediately after the completion of the final intravenous administration.
- **Treatment:** patients are randomized to receive either rHSA or pHSA, administered at 20 g/day for 7 consecutive days, or until serum albumin level reaches the discontinuation criterion (ALB ≥40 g/L), whichever occurs first.

Intercurrent Events and Their Addressing Strategies (Table 3).

Table 3 Intercurrent Events and Addressing Strategies

Intercurrent event	Addressing strategies	Note
Premature treatment discontinuation due to inadequate increase in albumin level after treatment	Composite strategy	Data collected after treatment discontinuation are not used in the primary estimand analysis. Resulting missing values for the primary efficacy endpoint will be handled via Multiple Imputation (MI) based on data from the worst 5% of participants (in terms of change from baseline in serum albumin) within the corresponding group. If the model fails to converge, imputation will be based on the central tendency (e.g., mean or median, depending on data distribution) of these 5% participants.

Premature treatment discontinuation due to adverse event	Treatment strategy	Data collected after premature treatment discontinuation will be included in the primary estimand analysis
Premature treatment discontinuation due to other reasons, e.g., participant withdrawal of informed consent	Hypothetical strategy	Data collected after treatment discontinuation will not be used for the primary estimand analysis. Resulting missing values for the primary efficacy endpoint will be handled via Multiple Imputation (MI) based on participants within the corresponding treatment group
Increase in baseline dose of diuretics during the treatment period; concomitant use of other albumin, blood products, or prohibited medications/treatments; death	Composite strategy	Data collected after the occurrence of an intercurrent event (except death) will not be used for the primary estimand analysis. Missing values for the primary efficacy endpoint due to these events or death will be handled via Multiple Imputation (MI) based on data from the worst 5% of participants (in terms of change from baseline in serum albumin) within the corresponding group. If the model fails to converge, imputation will be based on the central tendency (e.g., mean or median) of these 5% participants.

Where a participant experiences multiple concomitant events, the determination shall be made by the data review meeting.

Population-level summary: The difference between groups in the least squares mean of the change from baseline in serum albumin level, along with its 95% CI. The analysis will be based on a Covariance model, with baseline serum albumin as a covariate and including randomization stratification factors.2.2 Key Secondary Estimand

Key Secondary Estimand:

- **Clinical Question of Interest:** Whether the ascites improvement rate at the completion of the final intravenous administration of rHSA is non-inferior to that of pHSA in patients with cirrhotic ascites and hypoalbuminemia.
- **Target Population:** Patients with cirrhotic ascites and hypoalbuminemia who meet the inclusion criteria and do none of the exclusion criteria.
- **Target Variable:** Ascites improvement rate at completion of the final intravenous infusion.
- **Treatment:** Patients are randomized to receive recombinant human serum albumin (rHSA) or plasma-derived human serum albumin (pHSA) at a dose of 20 g/day for 7 consecutive days, or until the serum albumin level reaches the discontinuation criterion (ALB ≥ 40 g/L), whichever occurs first.

Intercurrent Events and Their Addressing Strategies (Table 4).

Table 4 Intercurrent Events and Addressing Strategies

Intercurrent event	Addressing strategies	Note
Premature treatment discontinuation due to inadequate improvement in ascites after treatment	Composite strategy	Considered as no improvement in ascites depth
Premature treatment discontinuation due to adverse event	Treatment strategy	Data collected after premature treatment discontinuation will be included in the primary estimand analysis
Premature treatment discontinuation due to other reasons, e.g., participant withdrawal of informed consent	Hypothetical strategy	Data collected after treatment discontinuation will not be used for the primary estimand analysis. Resulting missing values for the primary efficacy endpoint will be handled via Multiple Imputation (MI) based on participants within the corresponding treatment group
Increase in baseline dose of diuretics during the treatment period; concomitant use of other albumin, blood products, or prohibited medications/treatments; death	Composite strategy	Considered as no improvement in ascites depth

Where a participant experiences multiple concomitant events, the determination shall be made by the data review meeting.

Population-level summary: The difference between groups in the ascites improvement rate (investigational drug group - comparator group) along with its 95% CI.

2.3 Other efficacy indicators

Statistical analysis was conducted using the general principles of statistical analysis.

3 General Considerations for Statistical Analysis

3.1 General Principles

All statistical analyses will be performed using SAS® version 9.4 or higher.

Trial data will be summarized and tabulated by treatment group and visit/time point (as applicable). In general, continuous variables will be described using the number of participants, mean, median, standard deviation, minimum, and maximum; categorical and ordinal variables will be described using the frequency and percentage of each category or level. Unless otherwise specified, percentages will be calculated based on the total number of participants in the corresponding analysis set. Survival variables will be analyzed using the Kaplan-Meier method.

For the Phase 3 study, an equivalence hypothesis test will be conducted for the primary efficacy endpoint. The change from baseline in serum albumin level immediately following completion of the

final intravenous infusion (based on central laboratory results) will serve as the primary efficacy endpoint to demonstrate that the clinical efficacy of the investigational product (rHSA) is equivalent to that of the control product (pHSA). The pre-specified equivalence margin is -2 g/L to 2 g/L. Additionally, a non-inferiority assessment will be performed for the key secondary efficacy endpoint of ascites improvement rate at completion of the final intravenous infusion, with a non-inferiority margin of -10%.

Except for the primary and key secondary efficacy endpoints, hypothesis testing for other efficacy endpoints will be conducted for exploratory purposes, employing statistical methods to explore the data for better understanding. Unless otherwise stated, all statistical tests will use a two-sided significance level of $\alpha = 0.05$, and two-sided 95% confidence intervals (CIs) will be calculated.

Unless otherwise specified, the rules for retaining decimal places in statistical analysis results will follow Table 5. In the table below, if the rule for retaining decimal places is a numeric value, it indicates that the number of decimal places to be retained is that value; if it is in the form of X + a number, it indicates that the actual number of decimal places to be retained is the number of decimal places in the original data plus that number.

Table 5 Rules for Retaining Decimal Places

**Statistics**	**Rules for retaining decimal places**
The number of participants in the analysis set (N)	0
The number of participants in a certain category (n)	0
Percentage (%	If the percentage calculation result is not 100, keep one decimal place. If it is 100, no decimal places will be displayed. If the number of cases is 0, the percentage result will not be displayed.
Mean, least squares mean	X + 1
Standard deviation, standard error	X + 2
Minimum value	X
Median, first quartile (Q1), third quartile (Q3)	X + 1
Maximum value	X
95% CI	X + 1
"P value	4. If the p value is less than 0.0001, then it is expressed as < Let 0.0001 represent it

Note: All statistical quantities should be rounded to no more than four decimal places.

3.2 Analysis Populations

Full Analysis Set (FAS): All randomized participants who have received at least one dose of the investigational product will be included in the FAS. This analysis set will be utilized for the primary analysis of patient distribution, demographic, and baseline characteristics, as well as for the primary efficacy analysis in phase 3.

Per-Protocol Set (PPS): All randomized participants who have received at least one dose of the investigational product, have not committed major protocol violations, and have not received treatments that could significantly affect the efficacy assessment. The PPS will be employed for supplementary efficacy analyses in Phase 3.

Safety Set (SS): All randomized participants who have received at least one dose of the investigational product and have undergone at least one post-dose safety evaluation will be included in the SS. The SS represents the safety evaluation population for this trial.

Pharmacodynamic (PD) Analysis Set: All randomized participants who have received at least one dose of the investigational product and have at least one post-dose effective plasma colloid osmotic pressure data point will be included in the PD analysis set. This set will be utilized for pharmacodynamic analysis.

Immunogenicity Analysis Set: All randomized participants who received at least one dose of the investigational product and have at least one post-dose immunogenicity assessment will be included in the Immunogenicity Analysis Set. This set will be used for immunogenicity analyses.

Re-treatment Analysis Set: All phase 3 participants who received retreatment with the investigational product will be included in the Retreatment Analysis Set. This set will be used for safety and efficacy evaluations of retreatment.

3.3 Multicenter Studies

Data from all centers are planned to be pooled for analysis.

3.4 Covariate Adjustment

In model analyses of change from baseline or percent change from baseline for efficacy endpoints, the corresponding baseline value will be included as a covariate.

3.5 Multiplicity

The primary efficacy endpoint for the Phase 3 study is the change from baseline in serum albumin level immediately after completion of the final intravenous administration (based on central laboratory results), which will be evaluated using an equivalence hypothesis test. A non-inferiority test will be performed for the key secondary efficacy endpoint—ascites improvement rate at completion of the

final intravenous administration—at a one-sided significance level of 0.025. The key secondary efficacy endpoint will be considered met if the non-inferiority hypothesis is achieved.

Analyses of all other efficacy endpoints will be regarded as supportive, and statistical results for these endpoints will be interpreted with caution.

3.6 Subgroup Analysis

If data permit, descriptive analyses of the primary efficacy endpoint will be conducted for the following subgroups:

- Sex (Male, Female)
- Age (< 60 years [< 40 years, ≥ 40 and < 60 years], ≥ 60 years)
- Baseline serum albumin (< 25 g/L, ≥ 25 g/L) (derived from IWRS and screening serum albumin level)
- Baseline ascites depth (< 3 cm; 3–6 cm [including 3, excluding 6]; 6–10 cm [including 6 and 10]) (derived from IWRS and maximum ascites depth at screening)
- Baseline diuretic use (Yes; No) (based on IWRS and CRF)
- Baseline abdominal circumference (< median abdominal circumference [cm], ≥ median abdominal circumference [cm])
- Baseline body weight (< median body weight [kg], ≥ median body weight [kg])

4 Data Processing Rules

4.1 Derivative Variables

4.1.1 Definition of the Baseline and Change from Baseline

Except for the analyses of abdominal circumference and body weight, baseline is defined as the last non-missing assessment/examination prior to the first administration of the investigational medicinal product. For the analyses of abdominal circumference and body weight, baseline is defined as the mean of three measurements obtained prior to the first administration of the investigational medicinal product on Day 1. Change from baseline is calculated as the post-baseline assessment/examination value minus the baseline value.

4.1.2 Definition of Baseline for Re-treatment and Change from Baseline for Re-treatment

Except for the analyses of abdominal circumference and body weight, the baseline prior to re-treatment is defined as the last non-missing assessment/examination prior to the first administration of the investigational medicinal product during the re-treatment period. For the analyses of abdominal circumference and body weight, the baseline prior to re-treatment is defined as the mean of three measurements obtained prior to the first administration of the investigational medicinal product on Re-treatment Day 1 (RD1) of the first re-treatment. Change from baseline for re-treatment is calculated as the post-baseline assessment/examination value minus the baseline value.

4.1.3 Treatment Day

Treatment day is defined as the number of days relative to the reference date for efficacy/safety/pharmacodynamic (PD)/immunogenicity assessments/examinations. The date of the first administration of the investigational medicinal product is designated as the reference date. The reference date itself is recorded as Day 1.

• Treatment Day = Assessment/Examination Date – Reference Date + 1, if the assessment/examination occurs after the reference date

or

• Treatment Day = Assessment/Examination Date – Reference Date, if the assessment/examination occurs prior to the reference date.

4.1.4 Re-treatment Day

Re-treatment day is defined as the number of days relative to the reference date for efficacy/safety/pharmacodynamic (PD)/immunogenicity assessments/examinations. The date of the first administration of the investigational medicinal product during the re-treatment period is designated as the reference date. The reference date itself is recorded as Day 1.

• Re-treatment Day = Assessment/Examination Date – Reference Date + 1, if the assessment/examination occurs after the reference date

or

• Re-treatment Day = Assessment/Examination Date – Reference Date, if the assessment/examination occurs prior to the reference date.

In addition, corresponding forest plots will be generated.

4.2 Missing Data

For participants who withdraw prematurely from the study, all available data will be listed and included in the respective analyses where applicable.

The specific principles for handling missing efficacy data are detailed in Section 5.2. Unless otherwise specified, other missing data will be treated as missing and will not be imputed with any assumed values.

4.2.1 Missing or Incomplete Dates

If a date is completely missing, no imputation will be performed.

Adverse Events and Concomitant Medications

- If only the year and month are recorded for the start date of an adverse event or concomitant medication, and both the year and month are identical to those of the first dose date of the investigational medicinal product, the start date will be imputed as the first dose date (if the imputed start date is later than the end date, the start date will be set equal to the end date); otherwise, the start date will be imputed as the first day of the month in which the adverse event or concomitant medication started.
- If only the year is recorded for the start date of an adverse event or concomitant medication, and the year is identical to that of the first dose date of the investigational medicinal product, the start date will be imputed as the first dose date (if the imputed start date is later than the end date, the start date will be set equal to the end date); otherwise, the start date will be imputed as January 1 of the year in which the adverse event or concomitant medication started.
- If the end date of an adverse event or concomitant medication is incomplete, the end date will be imputed as the last day of the month or December 31 of the year in which the adverse event or concomitant medication ended. If the imputed end date is later than the study completion date, the end date will be set equal to the study completion date.

4.3 Visit Windows

To maximize data inclusion accounting for potential deviations from protocol-specified visit windows, out-of-window visits, unscheduled visits, early treatment discontinuation visits, or early termination visits will be remapped to corresponding protocol-scheduled visits based on the actual assessment date relative to the study day following the first intravenous administration per Table 6 for inclusion in the summary analysis. If remapped visits overlap with existing protocol-scheduled visits, only the scheduled timepoint will be summarized.

Table 6. Protocol-Specified Visit Windows (Phase 3, Groups A and B)

Inspection items	"Visit"	The time window (day) based on the treatment day
Vital signs	Baseline	≤ 1
	D14	≥ 8 ~ < 22
	D29	≥ 22 ~ < 43
	D57	≥ 43 ~ <74
	D90	≥ 74
Abdominal circumference, weight	Baseline	≤ 1

**Inspection items**	**"Visit"**	**The time window (day) based on the treatment day**
	D29	≥ 15 ~ < 43
	D57	≥ 43 ~ < 74
	D90	≥ 74
Abdominal ultrasound examination	Baseline	≤ 1
	D3	> 1 ~ < 5
	D7	≥ 5 ~ < 18
	D29	≥ 18 ~ < 43
	D57	≥ 43 ~ < 74
	D90	≥ 74
Physical examination	Baseline	≤ 1
	D2	> 1 ~ < 3
	D7	≥ 3 ~ < 11
	D14	≥ 11 ~ < 22
	D29	≥ 22 ~ < 43
	D57	≥ 43 ~ < 74
	D90	≥ 74
Blood routine, blood biochemistry	Baseline	≤1
	D2	>1 ~ <3
	D3	≥3 ~ <5

**Inspection items**	**"Visit"**	**The time window (day) based on the treatment day**
	D7	≥5 ~ <11
	D14	≥11 ~ <22
	D29	≥22 ~ <43
	D57	≥ 43 ~ < 74
	D90	≥ 74
Coagulation function, urine routine, 12-lead electrocardiogram	Baseline	≤ 1
	D3	> 1 ~ < 5
	D7	≥ 5 ~ < 18
	D29	≥ 18 ~ < 43
	D57	≥ 43 ~ < 74
	D90	≥ 74
Glycated hemoglobin test	Baseline	≤ 1
	D29	≥ 15 ~ < 74
	D90	≥ 74
Glycated albumin test	Baseline	≤ 1
	D7	> 1
Thyroid function tests, thyroid ultrasound examinations, iodine intake tests	Baseline	≤ 1
	D7	> 1 ~ < 18
	D29	≥ 18 ~ < 43

Inspection items	"Visit"	The time window (day) based on the treatment day
	D57	≥ 43 ~ < 74
	D90	≥ 74
Fundus examination	D1	≤ 1
	D90	> 1
PD	Baseline	≤ 1
	D7	> 1
ADA	Baseline	≤ 1
	D90	> 1

For analyses related to the first retreatment, out-of-window visits, unscheduled visits, early treatment discontinuation visits, or early withdrawal visits will be remapped to the corresponding protocol-planned visits based on actual assessment dates relative to the study day following the first intravenous administration in the retreatment period per Table 7 for inclusion in summary analyses. Should such visits overlap with existing protocol-planned visits after remapping, only the planned time points will be included in summary analyses.

Table 7. Protocol-Defined Visit Windows for First Retreatment During the Phase 3 Follow-up Period

Inspection items	"Visit"	Based on the time window (days) of the re-treatment day
Abdominal ultrasound examination	Re-treatment baseline	≤ 1
	RD3	> 1 ~ < 5
	RD7	≥ 5
Physical examination, blood routine, blood biochemistry, coagulation function test, urine routine, 12-lead electrocardiogram	Re-treatment baseline	≤ 1
	RD3	> 1 ~ < 5
	RD7	≥ 5

5 Statistical Analysis

5.1 Study Participants

5.1.1 Participant Disposition

All screened participants will be included in the analysis.

Participants who withdraw from the study prior to randomization will be considered screen failures. The total number and reasons for screen failure will be reported, with reasons for screen failure listed in tabular format. Screen-failed participants will not be included in any subsequent analyses.

The number and percentage of participants randomized, who received at least one dose of investigational product during the Treatment Period, who completed the Treatment Period dosing, who prematurely discontinued treatment during the Treatment Period, who received at least one dose of investigational product during the First Retreatment Period, who completed the First Retreatment Period dosing, who prematurely discontinued treatment during the First Retreatment Period, who completed the study, and who prematurely withdrew from the study will be summarized by treatment group. Primary reasons for treatment discontinuation and premature study withdrawal will also be summarized. The primary reasons for treatment discontinuation during the Treatment Period/First Retreatment Period and premature study withdrawal will be presented in tables according to the order recorded in the electronic Case Report Form (eCRF). A data listing will be provided for participants who discontinued treatment or prematurely withdrew from the study during the Treatment Period/Retreatment Period, including the date and reason for treatment discontinuation or premature withdrawal during the respective period.

The number and percentage of participants in each analysis dataset will be summarized by treatment group, using the number of all randomized participants as the denominator for percentage calculations. In addition, the distribution of participants across analysis datasets and reasons for exclusion from analysis datasets will be listed.

Furthermore, the number and percentage of participants randomized, who received at least one dose of investigational product during the Treatment Period, who completed the Treatment Period dosing, who prematurely discontinued treatment during the Treatment Period, who received at least one dose of investigational product during the Retreatment Period, who completed the First Retreatment Period dosing, who prematurely discontinued treatment during the First Retreatment Period, who completed the study, who prematurely withdrew from the study, and who were included in each analysis set will be summarized by treatment group and study center.

5.1.2 Protocol Deviations

Protocol deviations will be classified as Non-Important or Important.

The investigator and sponsor will evaluate and reach consensus on the classification of protocol deviations as important or non-important during data review prior to database lock. Final determination of Important Protocol Deviations will be confirmed and documented prior to database lock.

Important Protocol Deviations for all randomized participants will be summarized by treatment group, with separate listings provided for Important Protocol Deviations and all protocol deviations.

5.1.3 Demographic and Baseline Characteristics

Based on the FAS, the following demographic and baseline characteristics will be summarized by treatment group in tabular format. Additionally, a listing of demographic and baseline characteristics will be provided for all randomized participants.

5.1.3.1 Demographics

·• Age (years)

• Age categories (< 60 years [< 40 years, ≥ 40 and < 60 years], ≥ 60 years)

• Sex (male, female)

• Ethnicity (Han, other)

• Height (cm)

• Weight (kg)

• BMI (kg/m^2)

Note: Summaries will be based on data from the Demographics domain in the EDC.

5.1.3.2 Baseline Characteristics

• Baseline serum albumin (< 25 g/L, ≥ 25 g/L) (derived from IWRS and screening serum albumin level)

• Screening serum albumin level (g/L)

• Baseline serum albumin level (g/L)

• Baseline ascites depth (< 3 cm; 3-6 cm [inclusive of 3, exclusive of 6]; 6-10 cm [inclusive of 6 and 10]) (derived from IWRS and screening maximum ascites depth)

• Screening maximum/total ascites depth (mm)

• Baseline maximum/total ascites depth (mm)

• Baseline ascites grade (Grade 1, Grade 2)

• Baseline diuretic use (yes; no) (based on IWRS and CRF)

• Baseline abdominal circumference (cm)

• Baseline abdominal circumference category (< median abdominal circumference cm, ≥ median abdominal circumference cm)

• Baseline body weight (kg)

• Baseline body weight category (< median body weight kg, ≥ median body weight kg)

• Baseline hepatitis B screening result

• Baseline hepatitis C screening result·

Note: Baseline serum albumin (< 25 g/L, ≥ 25 g/L) (IWRS) is defined as the stratification factor selected in IWRS at randomization; baseline serum albumin (< 25 g/L, ≥ 25 g/L) (derived from screening serum albumin level) is defined as the categorical variable derived from the last serum albumin level recorded in the EDC prior to the date of randomization. Screening serum albumin level (g/L) is defined as the last serum albumin level recorded in the EDC prior to the date of randomization. Baseline serum albumin level (g/L) is defined as the last non-missing local laboratory serum albumin level prior to the first administration of investigational product. Baseline ascites depth (< 3 cm; 3–6 cm [inclusive of 3, exclusive of 6]; 6–10 cm [inclusive of 6 and 10]) (IWRS) is defined as the stratification factor selected in IWRS at randomization; baseline ascites depth (< 3 cm; 3–6 cm [inclusive of 3, exclusive of 6]; 6–10 cm [inclusive of 6 and 10]) (derived from screening maximum ascites depth) is defined as the categorical variable derived from the last maximum ascites depth recorded in the EDC prior to the date of randomization. Screening maximum/total ascites depth (mm) is defined as the last maximum/total ascites depth recorded in the EDC prior to the date of randomization. Baseline maximum/total ascites depth (mm) is defined as the last non-missing maximum/total ascites depth prior to the first administration of investigational product. Baseline ascites grade (Grade 1, Grade 2) is defined as the last non-missing ascites grade prior to the first administration of investigational product. Baseline diuretic use (yes; no) (IWRS) is defined as the stratification factor selected in IWRS at randomization; baseline diuretic use (yes; no) (CRF) is defined as the baseline diuretic use recorded in the EDC. Baseline abdominal circumference (cm)/body weight (kg) is defined as the mean of three measurements of abdominal circumference/body weight taken prior to administration of investigational product on Day 1. Baseline abdominal circumference category (< median abdominal circumference cm, ≥ median abdominal circumference cm)/baseline body weight category (< median body weight kg, ≥ median body weight kg) is defined as the derived categorical variable of baseline abdominal circumference (cm)/baseline body weight (kg). Baseline hepatitis B/hepatitis C screening result is defined as the last non-missing hepatitis B/hepatitis C screening result prior to the first administration of investigational product.

5.1.3.3 Other Baseline Assessments

Other baseline characteristics (alcohol consumption history, allergy history, infectious disease screening, routine stool test, echocardiography, etc.) will be listed by treatment group.

5.1.4 Medical History and Surgical History

Medical history was coded using the Medical Dictionary for Regulatory Activities (MedDRA) version 27.0 or higher.

In the FAS, medical histories for each treatment group were summarized by System Organ Class (SOC) and Preferred Term (PT), with the number and percentage of participants reported.

Medical histories and surgical histories were listed for all randomized participants by treatment group.

5.1.5 Previous and Concomitant Medications

Medications will be coded using the WHO Drug Dictionary (WHO Drug Global) version March 1, 2024 or later. Both the Preferred Name (PN) and the Anatomical Therapeutic Chemical (ATC) classification will be provided.

Based on the FAS, prior and concomitant medications will be summarized by treatment group according to ATC Level 2 and PN, with the number and percentage of participants calculated as follows:

- **Prior medications**: Medications that were discontinued prior to the first dose of investigational product.
- **Concomitant medications**: Medications used after the first dose of investigational product, including medications that were ongoing at the time of the first dose of investigational product and medications that were initiated after the first dose of investigational product.

Based on the Retreatment Analysis Set, concomitant medications during the retreatment period will be summarized by treatment group according to ATC Level 2 and PN, with the number and percentage of participants calculated.

- Concomitant medications during the retreatment period are defined as medications administered after the first dose of investigational product during the retreatment period, including medications that were initiated prior to but continued after the first dose, and medications initiated after the first dose.

If the timing of medication use relative to the first dose of investigational product cannot be determined, the medication will be considered concomitant.

Listings of prior and concomitant medications will be provided for all randomized participants by treatment group.

5.1.6 Prior and Concomitant Non-Drug Therapies

Listings of prior and concomitant non-drug therapies will be provided for all randomized participants by treatment group.

5.2 Efficacy Analysis

5.2.1 Analysis of the Primary Esitmand

The primary efficacy endpoint is defined as the change from baseline in serum albumin level, confirmed by albumin testing immediately after completion of the final intravenous infusion, based on central laboratory results.

5.2.1.1 Hypothesis Testing

The following hypotheses are specified:

Null Hypothesis (H_0):$\mu_{A\ group} - \mu_{B\ group} \leq -2$ or $\mu_{A\ group} - \mu_{B\ group} \geq 2$

Alternative Hypothesis (H_1):$-2 < \mu_{A\ group} - \mu_{B\ group} < 2$

where $\mu_{A\ group}$ and $\mu_{B\ group}$ represent the mean change from baseline in serum albumin level, confirmed by albumin testing immediately after completion of the final intravenous infusion (based on central laboratory results), for Group A (investigational product 20 g/day for 7 consecutive days) and Group B (comparator 20 g/day for 7 consecutive days), respectively.

5.2.1.2 Primary Analysis

Based on the FAS, serum albumin level values and changes from baseline measured immediately following completion of the final intravenous infusion (confirmed by albumin laboratory assessments) will be summarized by treatment group (number of participants, mean, median, standard deviation, standard error [as appropriate], minimum, and maximum).

An ANCOVA model will be fitted with change from baseline in serum albumin level measured immediately following completion of the final intravenous infusion (confirmed by albumin laboratory assessments) as the response variable, treatment group as a fixed effect, and baseline serum albumin level (continuous outcome), baseline ascites depth (< 3 cm; 3-6 cm [inclusive of 3, exclusive of 6]; 6-10 cm [inclusive of 6 and 10]) (IWRS), and baseline diuretic use (yes; no) (IWRS) as covariates. Least squares mean (LSM), standard error (SE), and corresponding 95% CIs for change from baseline in serum albumin level measured immediately following completion of the final intravenous infusion (confirmed by albumin laboratory assessments) will be reported for each treatment group. Additionally, the difference in LSMs between treatment groups (Group A – Group B), its SE, and corresponding 95% CI for change from baseline in serum albumin level measured immediately following completion of the final intravenous infusion (confirmed by albumin laboratory assessments) will be reported.

If intercurrent events occur, multiple imputation will be performed according to the corresponding intercurrent event handling strategies. Multiple imputation will be conducted using SAS PROC MI, employing the fully conditional specification (FCS) method to generate 20 complete datasets with a seed value of 954371. The ANCOVA model described above will be applied to each of the 20 complete datasets, and the results will be pooled using SAS PROC MIANALYZE to obtain the final primary efficacy endpoint analysis results.

Serum albumin levels from the central laboratory will be listed for all randomized participants by treatment group.

5.2.1.3 Sensitivity Analysis

Sensitivity analysis 1:

An ANOVA model will be used for the analysis. The change from baseline in serum albumin level confirmed by the albumin assessment immediately following completion of the final intravenous infusion will serve as the dependent variable, with treatment group as a fixed effect. The LSM, SE, and corresponding 95% CI for the change from baseline in serum albumin level confirmed by the albumin assessment immediately following completion of the final intravenous infusion will be reported for each treatment group. Additionally, the difference in LSMs between treatment groups (Group A minus Group B), along with its SE and corresponding 95% CI, will be reported.

If a participant experiences an intercurrent event, the pooled analysis will be conducted using the imputed dataset as described in the primary analysis. The ANOVA model described above will be applied separately to each of the 20 complete datasets, and the results will be combined using the SAS procedure PROC MIANALYZE to obtain the final analysis results for the primary efficacy endpoint.

Sensitivity Analysis 2:

An ANCOVA model will be employed, with the change from baseline in serum albumin level (as confirmed by albumin assessment immediately following completion of the final intravenous administration) as the dependent variable, treatment group as a fixed effect, and baseline serum albumin level (continuous) as a covariate. LSM, SE, and corresponding 95% CI for the change from baseline will be reported for each treatment group. Additionally, the difference in LSM between treatment groups (Group A minus Group B), together with its SE and corresponding 95% CI, will be reported.

In the presence of intercurrent events, a pooled analysis will be conducted using datasets imputed according to the methods described in the primary analysis. The ANCOVA model described above will be applied to each of the 20 complete datasets, and the results will be combined using SAS PROC MIANALYZE to yield the final analysis results for the primary efficacy endpoint.

5.2.1.4 Supplementary Analysis

Supplementary analysis 1:

For intercurrent events including premature treatment discontinuation due to lack of efficacy or adverse events, increased concomitant diuretic dose during treatment, and use of concurrent albumin, blood products, or prohibited medications/treatments during the treatment period, a hypothetical strategy will be applied. Efficacy data collected after the occurrence of intercurrent events will not be included in the analysis, and missing data resulting from this approach will not be imputed. Analysis will be performed using the ANCOVA method described in the primary analysis based on available participant data.

If intercurrent events occur, multiple imputation will be conducted according to the handling strategies for corresponding intercurrent events described in this section and the primary analysis (as applicable). Multiple imputation will be performed using SAS PROC MI, employing fully conditional specification (FCS) to generate 20 complete datasets with a seed number of 954371. The ANCOVA model described above will be applied to each of the 20 complete datasets, and the results will be combined using SAS PROC MIANALYZE to obtain the final analysis results for the primary efficacy endpoint.

Supplementary Analysis 2:

For intercurrent events including premature treatment discontinuation due to lack of efficacy or adverse events, increased concomitant diuretic dose during treatment, and use of concurrent albumin, blood products, or prohibited medications/treatments during the treatment period, a treatment policy strategy will be applied. Efficacy data collected after the occurrence of intercurrent events will be included in the analysis. The same analytical method as the primary analysis will be used for Supplementary Analysis 2.

Supplementary Analysis 3:

Supplementary Analysis 3 will be conducted using the same analytical method as the primary analysis based on the PPS.

5.2.1.5 Subgroup Analysis

Subgroup analyses will be performed on the FAS to evaluate the consistency of treatment effects across subgroups defined in Section 3.6, repeating the primary analyses within each subgroup. Corresponding forest plots will also be generated.

5.2.2 Key Secondary Efficacy Endpoint Analysis

The key secondary efficacy endpoint is defined as the ascites improvement rate at the completion of the final intravenous administration.

5.2.2.1 Hypothesis testing

The hypotheses established are as follows:

Null hypothesis (H_0): $\mathbf{P}_{\text{A group}} - \mathbf{P}_{\text{B group}} \leq -0.01$

Alternative hypothesis (H_1): $\mathbf{P}_{A\ group}$ -$\mathbf{P}_{B\ group}$ > -0.01

Where $\mathbf{P}_{A\ group}$ and $\mathbf{P}_{B\ group}$ represent the ascites improvement rates at the completion of the final intravenous administration for A Group (investigational drug 20 g/day for 7 consecutive days) and B Group (comparator 20 g/day for 7 consecutive days), respectively.

5.2.2.2 Primary Analysis

Based on the FAS, calculate the response rate of ascites improvement at the time of completion of the final intravenous infusion and its corresponding 95% CI (based on the Clopper-Pearson method) by treatment group. Between-group comparisons will be stratified by randomization stratification factors: baseline human serum albumin level (< 25 g/L; ≥ 25 g/L) (IWRS), baseline ascites depth (< 3 cm; 3 to < 6 cm; 6 to 10 cm) (IWRS), and baseline diuretic use (yes; no) (IWRS). The between-group (A Group – B Group) difference in response rates and the corresponding 95% CI will be calculated using the stratified Newcombe method.

If intercurrent events occur, the corresponding intercurrent event handling strategies will be applied to impute missing data for ascites results at completion of the final intravenous infusion using multiple imputation (MI). The MI will be performed using the SAS procedure PROC MI with the fully conditional specification (FCS) method to generate 20 imputed datasets, with the seed set to 954371. Based on these 20 datasets, the determination of whether a participant achieved ascites improvement at completion of the final intravenous infusion will be made. By treatment group, the proportion of participants with ascites improvement at completion of the final intravenous infusion and the corresponding 95% CI will be summarized across the 20 datasets and pooled using the Wilson-Confidence-Interval method. Based on the 20 imputed datasets, the proportion of improvement and the corresponding 95% CI will be calculated for each treatment group within each stratum (adjusted randomization stratification factors: baseline human serum albumin level [< 25 g/L; ≥ 25 g/L] [IWRS], baseline ascites depth [< 3 cm; 3 to < 6 cm; 6 to 10 cm] [IWRS], baseline diuretic use [yes; no] [IWRS]), pooled using the stratified Wilson-Confidence-Interval method, and the between-treatment difference in rates and the corresponding 95% CI will be derived using the stratified Newcombe method.

Bar charts will be provided to present the proportion of participants with ascites improvement at completion of the final intravenous infusion by treatment group.

A listing of ascites depth for all randomized participants will be provided by treatment group.

5.2.2.3 Sensitivity Analysis

Sensitivity analysis 1:

The Newcombe method (without stratification factor adjustment) was employed to calculate the rate difference between treatment groups (Group A minus Group B) and its corresponding 95% CI.

In the event of concomitant events occurring in some participants, the dataset was imputed using the same method described in the primary analysis. Based on 20 imputed datasets, the improvement rates

for each group and their corresponding 95% CI were calculated separately, then pooled using the Wilson confidence interval method. Subsequently, the rate difference between treatment groups and its corresponding 95% CI were derived using the Newcombe method.

5.2.2.4 Supplementary Analysis

Supplementary Analysis 1:

Based on the PPS, supplementary analysis 2 was conducted using the same analytical method as that in the main analysis.

5.2.2.5 Subgroup Analysis

Based on the FAS, repeat the main analysis according to the subgroups defined in 3.6 to evaluate the treatment consistency among different subgroups. In addition, the corresponding forest plots will be drawn.

5.2.3 Secondary Efficacy Endpoint Analysis

The analysis of secondary efficacy endpoints will be based on observed data (i.e., missing data will not be imputed).

5.2.3.1 Change from Baseline in Serum Albumin Level Confirmed by Local Laboratory Albumin Assessment Immediately After Completion of Last Intravenous Dose

Based on the FAS, tabulate the observed values and change from baseline in serum albumin level confirmed by local laboratory albumin assessment immediately after completion of the final intravenous dose by treatment group (number of participants, mean, median, standard deviation, minimum, maximum).

An ANCOVA model will be used, with change from baseline in serum albumin level confirmed by local laboratory albumin assessment immediately after completion of the final intravenous dose as the dependent variable, treatment group as a fixed effect, and baseline serum albumin level (continuous), baseline ascites depth (< 3 cm; 3-6 cm [inclusive of 3, exclusive of 6]; 6-10 cm [inclusive of 6 and 10]) (IWRS), and baseline diuretic use (Yes; No) (IWRS) as covariates. Report the LSM, SE, and corresponding 95% CI for the change from baseline in serum albumin level confirmed by local laboratory albumin assessment immediately after completion of the final intravenous dose for each treatment group. In addition, report the difference in LSM between treatment groups (Group A – Group B), SE, and corresponding 95% CI for the change from baseline in serum albumin level confirmed by local laboratory albumin assessment immediately after completion of the final intravenous dose.

Based on the PPS, repeat the above statistical methods for supplementary analysis.

List the serum albumin levels examined by local laboratories for all randomly selected participants according to the treatment groups.

5.2.3.2 Change from Baseline in Ascites Depth at Completion of Last Intravenous Administration

Based on the FAS, Tabulate the observed values and change from baseline in ascites depth at completion of last intravenous administration by treatment group (number of participants, mean, median, standard deviation, minimum, maximum).

An ANCOVA model shall be employed, with change from baseline in ascites depth at completion of last intravenous administration as the dependent variable, treatment group as a fixed effect, and baseline ascites depth (continuous), baseline human serum albumin level (< 25 g/L; ≥ 25 g/L) (IWRS), and baseline diuretic use (Yes; No) (IWRS) as covariates. The LSM, SE, and corresponding 95% CI for change from baseline in ascites depth at completion of last intravenous administration shall be reported for each treatment group. Additionally, the difference in LSM between treatment groups (Group A – Group B), SE, and corresponding 95% CI for change from baseline in ascites depth at completion of last intravenous administration shall be reported.

Based on the PPS, Repeat the above statistical methods as a supplementary analysis.

List ascites depth for all randomized participants by treatment group.

5.2.3.3 Proportion of Participants with Serum Albumin Level ≥ 35 g/L Confirmed by Albumin Assessment at Completion of Last Intravenous Administration

Based on the FAS, calculate the proportion of participants with serum albumin level ≥ 35 g/L confirmed by albumin assessment at completion of last intravenous administration and its corresponding 95% CI (based on Clopper-Pearson method) by treatment group. For between-group comparison, adjust for randomization stratification factors: baseline human albumin level (< 25 g/L; ≥ 25 g/L) (IWRS), baseline ascites depth (< 3 cm; 3-6 cm [inclusive of 3, exclusive of 6]; 6-10 cm [inclusive of 6 and 10]) (IWRS), baseline diuretic use (Yes; No) (IWRS). Calculate the between-group (Group A - Group B) rate difference and its corresponding 95% CI based on stratified Newcombe method.

Provide a bar chart of the proportion of participants with serum albumin level ≥ 35 g/L confirmed by albumin assessment at completion of last intravenous administration for each treatment group.

Based on the PPS, repeat the above statistical methods for supplementary analysis.

5.2.3.4 Time to Serum Albumin Level ≥ 35 g/L

Time to serum albumin level ≥ 35 g/L is defined as the time from first intravenous administration to the first occurrence of serum albumin level ≥ 35 g/L during the treatment period.

If a participant does not achieve serum albumin level ≥ 35 g/L following first intravenous administration, the participant will be censored at the date of serum albumin assessment at the actual Day 7 visit or End of Treatment (EOT) visit. Participants who do not have any serum albumin

assessment after first intravenous administration will be censored at the date of first intravenous administration.

Based on the FAS, the Kaplan-Meier method will be used to estimate the time to serum albumin level ≥ 35 g/L for each treatment group, with Q1, median, and Q3 reported. Two-sided 95% CI for these estimates will be constructed using the Brookmeyer-Crowley method with log-log transformation. Kaplan-Meier curves will also be plotted.

A stratified Log-rank test will be used for between-group comparisons, with stratification factors including: baseline human albumin level (< 25 g/L; ≥ 25 g/L) (IWRS), baseline ascites depth (< 3 cm; 3 to < 6 cm; 6 to 10 cm) (IWRS), and baseline diuretic use (yes; no) (IWRS). A stratified Cox proportional hazards model will be used to estimate the hazard ratio (HR) of the test drug relative to the comparator, along with its 95% CI and associated p-value; the model will include treatment group and the randomization stratification factors (baseline human albumin level [< 25 g/L; ≥ 25 g/L] [IWRS], baseline ascites depth [< 3 cm; 3 to < 6 cm; 6 to 10 cm] [IWRS], and baseline diuretic use [yes; no] [IWRS]).

Based on the PPS, repeat the above statistical methods for supplementary analysis.

A listing of time to serum albumin level ≥ 35 g/L during the treatment period will be provided for all randomized participants by treatment group.

5.2.3.5 Change from Baseline in Abdominal Circumference at Completion of Last Intravenous Dosing

Based on the FAS, tabulate the observed values and changes from baseline in abdominal circumference at completion of last intravenous administration by treatment group (number of participants, mean, median, standard deviation, minimum, maximum).

An ANCOVA model was adopted, with the change in abdominal circumference from baseline at the completion of the final intravenous administration as the dependent variable, the treatment group as the fixed effect, and the following covariates: baseline abdominal circumference (continuous), baseline human serum albumin level (< 25 g/L; ≥ 25 g/L) (IWRS), baseline ascites depth (< 3 cm; 3-6 cm [inclusive of 3, exclusive of 6]; 6-10 cm [inclusive of 6 and 10]) (IWRS), and baseline diuretic use (yes; no) (IWRS). The LSM, SE, and corresponding 95% CI for the change in abdominal circumference from baseline at the completion of the final intravenous administration were reported for each treatment group. Additionally, the between-group differences in LSM (Group A minus Group B), SE, and corresponding 95% CI were reported.

Based on the PPS, repeat the above statistical methods for supplementary analysis.

List the abdominal circumference measurements of all randomized participants by treatment group.

5.2.3.6 Change from baseline in Body Weight at the completion of the final intravenous administration

Based on the FAS, tabulate the body weight observations at the completion of the final intravenous administration and the changes from the baseline (number of participants, mean, median, standard deviation, minimum, maximum) according to the treatment groups.

An analysis of covariance (ANCOVA) model will be employed with change from baseline in body weight at completion of last intravenous administration as the response variable, treatment group as a fixed effect, and baseline body weight (continuous), baseline serum albumin level (< 25 g/L; ≥ 25 g/L) (IWRS), baseline ascites depth (< 3 cm; 3-6 cm [inclusive of 3, exclusive of 6]; 6-10 cm [inclusive of 6 and 10]) (IWRS), and baseline diuretic use (yes; no) (IWRS) as covariates. The LSM, SE, and corresponding 95% CI for change from baseline in body weight at completion of last intravenous administration will be reported by treatment group. In addition, the difference in LSM between treatment groups (Group A - Group B) for change from baseline in body weight at completion of last intravenous administration, along with its SE and corresponding 95% CI, will be reported.

Based on the PPS, repeat the above statistical methods for supplementary analysis.

Listings of body weight measurements for all randomized participants will be provided by treatment group.

5.2.3.7 Change from Baseline in Serum Albumin Level at the End of Re-treatment

This endpoint applies to participants who received re-treatment with the investigational medicinal product from Day 29 to Day 76. Only data from the first re-treatment period will be analyzed.

For the Re-treatment Analysis Set, descriptive statistics (n, mean, median, standard deviation, minimum, and maximum) will be provided for the observed serum albumin level at the end of the re-treatment period and for the change from baseline prior to re-treatment, by treatment group.

An ANCOVA model will be used, with the change from baseline in serum albumin level at the end of the re-treatment period as the dependent variable, treatment group as a fixed factor, and the baseline serum albumin level prior to re-treatment as a continuous covariate.

LSM, SE, and two-sided 95% CI for the change from baseline in serum albumin level will be reported for each treatment group. Additionally, the difference in LSM between treatment groups (Group A minus Group B), along with the corresponding SE and two-sided 95% CI, will be reported.

5.2.3.8 Rate of Ascites Improvement at Completion of Last Intravenous Infusion During the Retreatment Period

This endpoint applies to participants who received retreatment with the investigational product from Day 29 to Day 76; only the first retreatment episode will be analyzed.

Based on the Retreatment Analysis Set, the proportion of participants with ascites improvement at completion of the final intravenous infusion during the retreatment period will be calculated by treatment group, with corresponding two-sided 95% CI computed using the Clopper-Pearson (exact)

method. The rate difference between treatment groups (Group A minus Group B) and its corresponding 95% CI will be calculated using the Newcombe method. Bar charts displaying the rate of ascites improvement at completion of the final intravenous infusion during the retreatment period by treatment group will be provided.

5.2.3.9 Change in Ascites Depth at Completion of Last Intravenous Administration During the Retreatment Period Relative to Retreatment Baseline

The above endpoint applies to participants who received retreatment with the investigational product from Day 29 to Day 76, and only the first retreatment episode will be analyzed.

Based on the Retreatment Analysis Set, the observed ascites depth and change from retreatment baseline at completion of the final intravenous administration during the retreatment period will be tabulated by treatment group (number of participants, mean, median, standard deviation, minimum, maximum).

An ANCOVA model will be used, with the change from retreatment baseline in ascites depth at completion of the final intravenous administration during the retreatment period as the dependent variable, treatment group as a fixed effect, and retreatment baseline ascites depth (continuous) as a covariate. The LSM, SE, and corresponding 95% CI for the change from retreatment baseline in ascites depth at completion of the final intravenous administration during the retreatment period will be reported for each treatment group. Additionally, the difference in LSM between treatment groups (Group A - Group B) for the change from retreatment baseline in ascites depth at completion of the final intravenous administration during the retreatment period, along with its SE and 95% CI, will be reported.

5.2.3.10 Proportion of Participants with Serum Albumin Level ≥ 35 g/L Confirmed by Albumin Assessment During the Retreatment Period

The above endpoint applies to participants who received retreatment with the investigational product from Day 29 to Day 76, and only the first retreatment episode will be analyzed.

Based on the Retreatment Analysis Set, the proportion of participants with serum albumin level ≥ 35 g/L confirmed by albumin assessment during the retreatment period and the corresponding 95% CI (based on the Clopper-Pearson method) will be calculated by treatment group. The difference in proportions between treatment groups (Group A - Group B) and the corresponding 95% CI will be calculated based on the Newcombe method. A bar chart showing the proportion of participants with serum albumin level ≥ 35 g/L confirmed by albumin assessment during the retreatment period for each treatment group will be provided.

5.2.3.11 Time to Reach Serum Albumin Level ≥ 35 g/L Confirmed by Albumin Assessment During the Retreatment Period

The above endpoint applies to participants who received retreatment with the investigational product from Day 29 to Day 76, and only the first retreatment episode will be analyzed.

The time to reach serum albumin level ≥ 35 g/L confirmed by albumin assessment during the retreatment period is defined as the time from the first intravenous administration during the retreatment period to the first post-dose serum albumin level ≥ 35 g/L during the retreatment period.

For participants who do not achieve serum albumin level ≥ 35 g/L after the first intravenous administration during the retreatment period, censoring will occur at the date of the actual RD7 visit or the last day of retreatment visit. Participants who do not have any serum albumin assessment after the first intravenous administration during the retreatment period will be censored at the date of the first intravenous administration.

Based on the Retreatment Analysis Set, the Kaplan-Meier method will be used to obtain the first quartile (Q1), median, and third quartile (Q3) of the time to reach serum albumin level ≥ 35 g/L confirmed by albumin assessment during the retreatment period for each treatment group, and the Brookmeyer-Crowley method with log-log transformation will be used to construct the two-sided 95% CI for the corresponding estimates. The Kaplan-Meier curve will also be plotted.

Treatment group comparison will be performed using the Log-rank test; the Cox proportional hazards model will be used to estimate the Hazard Ratio (HR) of the investigational product relative to the control product and its 95% CI, along with the p-value from the test.

5.2.3.12 Change in Abdominal Circumference at Completion of Last Intravenous Administration During the Retreatment Period Relative to Retreatment Baseline

The above endpoint applies to participants who received retreatment with the investigational product from Day 29 to Day 76, and only the first retreatment episode will be analyzed.

Based on the Retreatment Analysis Set, the observed abdominal circumference and change from retreatment baseline at completion of the final intravenous administration during the retreatment period will be tabulated by treatment group (number of participants, mean, median, standard deviation, minimum, maximum).

An ANCOVA model will be used, with the change from retreatment baseline in abdominal circumference at completion of the final intravenous administration during the retreatment period as the dependent variable, treatment group as a fixed effect, and baseline abdominal circumference (continuous) as a covariate. The LSM, SE, and corresponding 95% CI for the change from retreatment baseline in abdominal circumference at completion of the final intravenous administration during the retreatment period will be reported for each treatment group. Additionally, the difference in LSM between treatment groups (Group A - Group B) for the change from retreatment baseline in abdominal circumference at completion of the final intravenous administration during the retreatment period, along with its SE and 95% CI, will be reported.

5.2.3.13 Change in Body Weight at Completion of Last Intravenous Administration During the Retreatment Period Relative to Retreatment Baseline

The above endpoint applies to participants who received retreatment with the investigational product from Day 29 to Day 76, and only the first retreatment episode will be analyzed.

Based on the Retreatment Analysis Set, the observed body weight and change from retreatment baseline at completion of the final intravenous administration during the retreatment period will be tabulated by treatment group (number of participants, mean, median, standard deviation, minimum, maximum).

An ANCOVA model will be used, with the change from retreatment baseline in body weight at completion of the final intravenous administration during the retreatment period as the dependent variable, treatment group as a fixed effect, and baseline body weight (continuous) as a covariate. The LSM, SE, and corresponding 95% CI for the change from retreatment baseline in body weight at completion of the final intravenous administration during the retreatment period will be reported for each treatment group. Additionally, the difference in LSM between treatment groups (Group A - Group B) for the change from retreatment baseline in body weight at completion of the final intravenous administration during the retreatment period, along with its SE and 95% CI, will be reported.

5.2.4 Exploratory Analysis

5.2.4.1 Change from Baseline in Glycated Albumin After Administration

Based on the FAS, glycated albumin will be summarized by treatment group and visit. Observed values and changes from baseline will be tabulated (n, mean, standard deviation, median, minimum, and maximum).
Glycated albumin will be listed for all randomized participants by treatment group and visit.

5.2.4.2 Change from Baseline in Glycated Hemoglobin After Administration

Based on the FAS, glycated hemoglobin will be summarized by treatment group and visit. Observed values and changes from baseline will be tabulated (n, mean, standard deviation, median, minimum, and maximum).
Glycated hemoglobin will be listed for all randomized participants by treatment group and visit.

5.3 Safety Analysis

Safety analyses for the Treatment Period will be based on the SS, and safety analyses for the Retreatment Period will be based on the Retreatment Analysis Set.

5.3.1 Extent of Drug Exposure and Compliance

Based on the SS, descriptive statistics (number of participants, mean, standard deviation, median, minimum, and maximum) will be provided by treatment group for the actual number of dose administrations, total duration of dosing, total actual dose received, average daily dose, and medication compliance during the Treatment Period. The number and percentage of participants with different medication compliance categories (< 80%, 80%–120%, > 120%) will also be summarized.

• Actual number of dose administrations = Sum of all dosing occasions where the actual dose administered was not zero;

• Total duration of dosing (days) = Date of last dose – Date of first dose + 1;
• Total actual dose received (g) = Sum of all actual doses administered;
• Average daily dose (g/day) = Total actual dose received (g) / Total duration of dosing (days);
• Medication compliance (%) = Total actual dose received (g) / Planned total dose (g) × 100%, where the planned total dose is calculated as the total dose planned according to the protocol for the dose level received over the actual dosing duration.

In addition, based on the SS, the number and percentage of participants who experienced at least one infusion interruption, infusion discontinuation, or medication error during the Treatment Period will be summarized by treatment group, together with the reasons for these events.

Based on the Retreatment Analysis Set, the above statistical methods will be repeated to analyze drug exposure and compliance during the Retreatment Period.

A listing of investigational medicinal product administration for all randomized participants will be provided by treatment group.

5.3.2 Adverse Events

Adverse events will be coded using MedDRA 27.0 or higher.

Adverse events will be classified as follows:

• **Pre-treatment adverse events:** Events occurring after informed consent is obtained but prior to the first administration of investigational product.

• **Treatment-emergent adverse events (TEAEs):** Events that emerge during the treatment period (from the first dose of investigational product through the end of the follow-up period), including those not present prior to treatment or worsening of pre-existing conditions relative to baseline. *Note: Including adverse events occurring during the retreatment period.*

• **Retreatment-period adverse events:** Events that emerge during the retreatment period (from the first dose of investigational product in the retreatment period through the end of the follow-up period), including those not present prior to retreatment or worsening relative to pre-retreatment status.

If the temporal relationship of an adverse event to investigational product administration during the treatment period cannot be determined, the event will be classified as a TEAE.

If the temporal relationship of an adverse event to investigational product administration during the retreatment period cannot be determined, the event will be classified as a retreatment-period adverse event.

Treatment-related TEAEs (TRAEs) are defined as TEAEs with a causal relationship to investigational product assessed as "definitely related," "probably related," or "possibly related." In the absence of a relationship assessment, TEAEs will be conservatively considered as treatment-related.

Retreatment-period adverse events related to investigational product are defined as events with a causal relationship to investigational product assessed as "definitely related," "probably related," or "possibly related." In the absence of a relationship assessment, retreatment-period adverse events will be conservatively considered as related to investigational product.

Summaries of adverse events will focus on TEAEs and retreatment-period adverse events, tabulated by treatment group. All adverse events for randomized participants will be listed.

5.3.2.1 Summary of TEAEs/Summary of Adverse Events in the Retreatment Period

Based on the SS, summarize the number and percentage of participants and the number of events for the following categories of TEAEs by treatment group:

All TEAEs

- All TRAEs
- Grade ≥3 TEAEs per CTCAE
- Grade ≥3 TRAEs per CTCAE
- TEAEs leading to interruption of Study Drug
- TRAEs leading to interruption of Study Drug
- TEAEs leading to discontinuation of Study Drug
- TRAEs leading to discontinuation of Study Drug
- Infusion-related reactions
- Serious TEAEs (SAEs)
- Serious TRAEs
- Fatal TEAEs
- Fatal TRAEs

Based on the Retreatment Analysis Set, summarize adverse events occurring in the retreatment period using the same methodology described above.

5.3.2.2 Analysis of TEAEs/Adverse Events in the Retreatment Period by SOC and PT

Based on the SS, the number and percentage of participants and the number of events will be summarized by treatment group by MedDRA System Organ Class (SOC) and Preferred Term (PT) for the following:

- All TEAEs
- All TRAEs.

Based on the Retreatment Analysis Set, the above statistical methods will be repeated to analyze adverse events in the retreatment period by SOC and PT.

5.3.2.3 Analysis of TEAEs/Adverse Events in the Retreatment Period by SOC, PT, and Severity

Based on the SS, the number and percentage of participants with TEAEs or TRAEs will be summarized by treatment group by MedDRA SOC, PT, and severity (CTCAE grade). If a participant experience multiple TEAEs/TRAEs with the same SOC or PT, the participant will be counted only once at the corresponding SOC or PT level according to the maximum CTCAE grade.

Based on the Retreatment Analysis Set, the above statistical methods will be repeated to analyze adverse events in the retreatment period by SOC, PT, and severity.

5.3.2.4 Death, SAE, and Other Important TEAEs

5.3.2.4.1 Death

Based on the SS, the number and percentage of participants with fatal TEAEs/TRAEs and the number of corresponding events will be summarized by MedDRA SOC and PT for each treatment group.
Based on the Retreatment Analysis Set, the above analyses will be repeated for fatal TEAEs/TRAEs during the retreatment period by SOC and PT.
All fatal TEAEs for all randomized participants will be listed by treatment group.

5.3.2.4.2 SAE

Based on the SS, the number and percentage of participants with SAEs or serious TRAEs and the number of serious TEAE/TRAE events will be summarized by MedDRA SOC and PT for each treatment group.
Based on the Retreatment Analysis Set, the above analyses will be repeated for serious adverse events/serious adverse reactions during the retreatment period by SOC and PT.
All SAEs for all randomized participants will be listed by treatment group.

5.3.2.4.3 Other Important TEAEs/Adverse Events During the Retreatment Period

Based on the SS, the following important TEAEs will be summarized by MedDRA SOC and PT for each treatment group: the number and percentage of participants and the number of events:

- TEAEs with CTCAE Grade ≥3
- TRAEs with CTCAE Grade ≥3

Based on the Retreatment Analysis Set, the above analyses will be repeated for important adverse events during the retreatment period by SOC and PT.

In addition, the following important TEAEs will be presented in separate data listings:

- TEAEs with CTCAE Grade ≥3
- TRAEs with CTCAE Grade ≥3
- TEAEs leading to interruption of study drug
- TEAEs leading to discontinuation of study drug
- Infusion-related reactions

5.3.3 Laboratory Assessments

Laboratory assessments include hematology, clinical chemistry, coagulation tests, and urinalysis.

For the SS, quantitative laboratory parameters (hematology, clinical chemistry, and coagulation tests) will be summarized by treatment group and visit. Observed values and changes from baseline will be tabulated (n, mean, standard deviation, median, minimum, and maximum).

Additionally, for the SS, shift tables will be generated by treatment group and visit to display changes from baseline to the worst post-baseline result and to the final assessment (last non-missing assessment following the first dose of investigational product during the study period, including unscheduled visits), using the categories "Normal," "Abnormal without clinical significance," and "Abnormal with clinical significance," as determined by the investigator.

For the Retreatment Analysis Set, quantitative laboratory parameters (hematology, clinical chemistry, and coagulation tests) will be summarized by treatment group and visit. Observed values and changes from the pre-retreatment baseline will be tabulated (n, mean, standard deviation, median, minimum, and maximum).

Additionally, for the Retreatment Analysis Set, shift tables will be generated by treatment group and visit to display changes from the pre-retreatment baseline to the worst post-baseline result and to the final assessment (last non-missing assessment following the first dose of investigational product during the study period, including unscheduled visits), using the categories "Normal," "Abnormal without clinical significance," and "Abnormal with clinical significance," as determined by the investigator.

All laboratory data for all randomized participants will be listed by treatment group and visit, with values outside the normal range flagged. Additionally, all clinically significant abnormal laboratory findings occurring after dosing will be presented in a separate listing.

5.3.4 Vital Signs

Based on the SS, vital signs will be summarized by treatment group and visit/time point. Observed values and changes from baseline will be tabulated (n, mean, standard deviation, median, minimum, and maximum).

Additionally, for the SS, shift tables will be generated by treatment group and visit to display changes from baseline to the worst post-baseline result and to the final assessment (last non-missing assessment following the first dose of investigational product during the study period, including unscheduled visits), using the categories "Normal," "Abnormal without clinical significance," and "Abnormal with clinical significance," as determined by the investigator.

For the Retreatment Analysis Set, vital signs will be summarized by treatment group and visit/time point. Observed values and changes from the pre-retreatment baseline will be tabulated (n, mean, standard deviation, median, minimum, and maximum).

Additionally, for the Retreatment Analysis Set, shift tables will be generated by treatment group and visit to display changes from the pre-retreatment baseline to the worst post-baseline result and to the final assessment (last non-missing assessment following the first dose of investigational product during the study period, including unscheduled visits), using the categories "Normal," "Abnormal without clinical significance," and "Abnormal with clinical significance," as determined by the investigator.

Vital signs for all randomized participants will be listed by treatment group and visit/time point.

5.3.5 12-Lead Electrocardiogram (ECG)

Based on the SS, 12-lead ECGs will be summarized by treatment group and visit. Descriptive statistics (n, mean, standard deviation, median, minimum, and maximum) will be presented for observed values and change from baseline.

In addition, based on the Safety Set, shift tables by treatment group and visit will present changes from baseline to the worst post-baseline result and to the last assessment (the last non-missing assessment following the first dose of investigational product during the treatment period, including unscheduled visits), using the categories "Normal," "Abnormal not clinically significant," and "Abnormal clinically significant," as judged by the investigator.

Based on the Retreatment Analysis Set, 12-lead ECGs will be summarized by treatment group and visit. Descriptive statistics (n, mean, standard deviation, median, minimum, and maximum) will be presented for observed values and change from retreatment baseline.

In addition, based on the Retreatment Analysis Set, shift tables by treatment group and visit will present changes from retreatment baseline to the worst post-retreatment result and to the last assessment (the last non-missing assessment following the first dose of investigational product during the treatment period, including unscheduled visits), using the categories "Normal," "Abnormal not clinically significant," and "Abnormal clinically significant," as judged by the investigator.

A listing of 12-lead ECG assessments will be provided for all randomized participants by treatment group and visit.

5.3.6 Physical Examination

Based on the SS, shift tables showing changes from baseline to the worst post-baseline result and to the last observation (defined as the last non-missing assessment after the first administration of investigational product during the study period, including unscheduled assessments) will be provided by treatment group and visit, using the categories of "Normal," "Abnormal, not clinically significant," and "Abnormal, clinically significant," as assessed by the investigator.

Based on the Retreatment Analysis Set, shift tables showing changes from the pre-retreatment baseline to the worst post-baseline result and to the last observation (defined as the last non-missing assessment after the first administration of investigational product during the study period, including unscheduled assessments) will be provided by treatment group and visit, using the categories of "Normal," "Abnormal, not clinically significant," and "Abnormal, clinically significant," as assessed by the investigator.

Data listings of physical examinations will be provided for all randomized participants by treatment group and visit.

5.3.7 Fundus examination

Based on the SS, present shift tables showing changes from Baseline to the worst post-baseline result and to the last assessment (defined as the last non-missing assessment following the first dose of investigational product during the treatment period, including unscheduled visits) by treatment group and visit, using the categories "Normal", "Abnormal but not clinically significant", and "Abnormal and clinically significant" as determined by the investigator.

Provide listings of fundus examination results for all randomized participants by treatment group and visit.

5.3.8 Thyroid function test

Based on the SS, summarize the thyroid function tests according to the treatment group and visit, and tabulate the observed values and the changes from the baseline (number of cases, mean, standard deviation, median, minimum, and maximum).

In addition, based on the SS, according to the treatment group and visit, and as judged by the investigator, using the classifications of "normal", "abnormal without clinical significance", and "abnormal with clinical significance", a cross - table showing the changes from baseline to the most severe result after baseline and the last examination (the last non - missing examination after the first administration of the test article during the trial, including unscheduled examinations) is provided.

List the thyroid function tests of all randomized participants according to the treatment group and visit.

5.3.9 Thyroid Ultrasonography

Based on the SS, present shift tables of changes from baseline to the worst post-baseline result and to the last assessment (defined as the last non-missing examination after the first dose of study treatment during the trial period, including unscheduled visits) by treatment group and visit, using the categories "Normal," "Abnormal Not Clinically Significant," and "Abnormal Clinically Significant" based on the investigator's assessment.

List thyroid ultrasound examinations for all randomized participants by treatment group and visit.

5.3.10 Iodine Uptake Test

Based on the SS, iodine uptake tests will be summarized by treatment group and visit. Observed values and changes from baseline will be tabulated (n, mean, standard deviation, median, minimum, and maximum).

In addition, shift tables from baseline to the worst post-baseline result and to the last examination (defined as the last non-missing measurement after the first dose of investigational product during the trial period, including unscheduled visits) will be presented by treatment group and visit, using the categories "Normal," "Abnormal without Clinical Significance," and "Abnormal with Clinical Significance," as assessed by the investigator.

Listings of iodine uptake tests will be provided for all randomized participants by treatment group and visit.

5.3.11 Other Safety Assessments

Listings of other safety assessments (e.g., pregnancy tests) will be presented by treatment group and visit for all randomized participants.

5.4 PD Analysis

Based on the PD analysis set.

Plasma colloid osmotic pressure and change from baseline will be summarized by treatment group and visit, including the number of participants, mean, standard deviation, median, minimum, and maximum.

Plasma colloid osmotic pressure will be listed by treatment group and visit for all randomized participants.

5.5 Immunogenicity Analysis

Based on the immunogenicity analysis set.

The number and percentage of participants in each treatment group will be calculated for ADA results against rHSA according to the following categories:

Study-positive for rHSA ADA, defined as positive rHSA ADA test results at any visit during the study.

Baseline-positive for rHSA ADA.

Baseline-negative but post-treatment-positive for rHSA ADA, where post-treatment-positive is defined as positive rHSA ADA test results at any visit following administration of the investigational product.

The number and percentage of participants with positive rHSA ADA will be summarized by visit. For participants with positive rHSA ADA, antibody titers will be further analyzed, if data permit.

If data permit, PD, efficacy, and safety analyses will also be conducted for participants with positive rHSA ADA.

Statistical analyses of ADA and specific IgE against HCP will be performed using methods similar to those for rHSA ADA.

Immunogenicity data for all randomized participants will be listed by treatment group and visit.

6 Planned Analyses

6.1 IDMC

An Independent Data Monitoring Committee (IDMC) will be established for this phase 3 study to review safety data and other critical information from the clinical trial. Requirements for analyses to be performed by the IDMC are detailed in the IDMC Charter, IDMC SAP, and IDMC Mockup.

6.2 Final Analysis

The complete statistical analysis of the phase 3 study will be conducted after all participants have completed their final study visit.

7 Deviations from Planned Analyses

This Statistical Analysis Plan contains no deviations from the analyses planned in the protocol.

8 Templates for Statistical Tables, Listings, and Figures

Templates for statistical tables, listings, and figures will be provided in a separate document.
