# Supplementary material for: A randomized, double-blind, active-controlled phase 3 multicenter trial of rHSA for cirrhotic ascites
Source: Signal Transduct Target Ther. 2026 Sep 10;11:390. doi: 10.1038/s41392-026-02951-7 (PMC13588796; doi:10.1038/s41392-026-02951-7)
Supplement: Supplementary file 3 [file 41392_2026_2951_MOESM1_ESM.docx]

**Supplementary Materials for**

**A Randomized, Double-Blind, Active-Controlled Phase 3 Multicenter Trial of rHSA for Cirrhotic Ascites**

Jidong Jia, Yajun An, Xiaojuan Ou, Weijia Duan, Yongzhong Li, Yong Deng, Jun Xie, Qinghai Wang, Xing Li, Zhongwei Pan, Yawen Luo, Lijia Wang, Xuanfang Zhong, Dajun Wu, Zhongji Meng, Zhirong Liu, Zonghua Chen, Liang Hong, Yali Zong, Yingmei Tang, Lihua Zhong, Cheng Wo, Bin Xu, Baolian Shu, Fei Shi, Guangming Li, Guangming Xiao, Fengqian Song, Guoqiang Zhang, Haiyan Chen, Aijun Liao, Hongjian Wei, Junyi Li, Xuan An, Fangfang Li, Chenwei Pan, Jie Pan, Zhanzhou Lin, Wei Hou, Hong Li, Jianyong Chen, Xiaoping Wu, Shuilin Sun, Yueming Shen, Xiaoqu Zhu, Bin Chen, Yuehong Zeng, Xingguo Xiao, Jiawei Geng, Heng Zhang, Caiyan Zhao, Lu Chen, Qinfang Wu, Yang Li, Xiongxiang Liu, Xueqiang Jiang, Hui Tian, Zhongkuo Li, Ming Zhao, Huai Li, Xiaobo Xiong, Rui Yao, Li Shao, Fengzhu Liu, Lirong Fu, Youwei Liao, Tao Guan, Xinhua Luo, Wenfang Yuan, Hongbo Ji, Xiaolin Zhou, Yongzhang Luo.

Correspondence to: jia_jd@ccmu.edu.cn; yluo@tsinghua.edu.cn

**This PDF file includes:**

Supplementary Figs. 1 and 2

Tables S1 to S19

**Other Supplementary Materials for this manuscript include the following:**

Data S1 to S7

Data S1. MS_ Figure 2a. Primary endpoint: Change from baseline in serum albumin (ALB) levels (central laboratory) to Day 7 or the end of treatment (EOT).

Data S2. MS_ Figure 2b. Key secondary endpoint: Ascites improvement rate after the final administration.

Data S3. MS_ Figure 2c. Secondary endpoint: Change from baseline in serum ALB levels (local laboratory) to Day 7 or EOT.

Data S4. MS_ Figure 2d. Serum ALB levels over time (local laboratory)

Data S5. MS_ Figure 2e, 2f. Proportion of patients with serum ALB ≥35 gL at EOT (central laboratory) and (local laboratory).

Data S6. MS_ Figure 3a-3d. Liver function parameters (Figure 3a. Total bilirubin; Figure 3b. Serum creatinine; Figure 3c. Child‑Pugh score; Figure 3d. MELD 3.0 score).

Data S7. MS_ Figure 4b. Molecular integrity profiling of rHSA and pHSA [4b (i). Free thiol; 4b (ii). Homocysteine (Hcy) concentrations; 4b (iii). Advanced glycation end-product (AGE) concentrations; 4b (iv). Carbonyl concentrations].

# **Supplementary Fig.** **1 Subgroup analyses of changes in serum ALB levels from baselines (Full Analysis Set)**

A forest plot of subgroup analyses for changes in serum ALB levels from baseline confirmed by the immediate albumin assay after the final administration.

IWRS (Interactive Web Response System): the stratification factor selected at the time of randomization. CRF (Case Report Form): the outcome as documented in the electronic data capture (EDC) database.

# **Supplementary Fig. 2 Purity analysis of rHSA and pHSA by silver-stained SDS-PAGE at the laboratory scale.**

**a,** rHSA exhibited a single, distinct protein band, indicating high electrophoretic purity. In contrast, pHSA (a representative) displayed multiple minor bands. Sample load per lane: 5 μg.

**b,** Silver-stained SDS-PAGE of rHSA with increasing protein loads (10, 20, 30, and 40 μg per lane). The consistent observation of a single band, even at the highest loading amount, confirms the high purity of rHSA.

The silver staining method, with a detection limit of ≤ 1 ng, demonstrates the superior purity of rHSA.

# **Table S1. List of ADCS Site Principal Investigators**

| **PI name**  **(Last Name, First Name)** | **Institution Name** | **City** | **Approval No.** |
| --- | --- | --- | --- |
| Jidong Jia | Beijing Friendship Hospital, Capital Medical University | Beijing | 2022-P1-076-06 |
| Bin Xu | Beijing YouAn Hospital, Capital Medical University | Beijing | [2024]030 |
| Zhongjie Hu | Beijing YouAn Hospital, Capital Medical University | Beijing | [2024]030 |
| Jie Du; Changming Liu | The First Hospital of Changsha | Changsha | 20240117CSSY00107 |
| Xing Li | Pingxiang People's Hospital | Pingxiang | 2022Y090-KS10 |
| Shuqiang Ou | Pingxiang No.2 People's Hospital | Pingxiang | 2024-(Y)002-01 |
| Guangming Li | The Sixth People's Hospital of Zhengzhou | Zhengzhou | IEC-2023-002-06 |
| Yingjie Ma | People's Hospital of Zhengzhou | Zhengzhou | YW202300105 |
| Haiyan Chen | Xinyang Central Hospital | Xinyang | 2024-01-002-K05 |
| Jingbo Wang | Shandong Public Health Clinical Center | Jinan | GWLCZXEC2022-75-8 |
| Aijun Liao | The First Affiliated Hospital of University of South China | Hengyang | 【2022】(45-05) |
| Zhongwei Pan | Meihekou Central Hospital | Tonghua | 2022【015】-05 |
| Guangming Xiao | Guangzhou Eighth People's Hospital, Guangzhou Medical University | Guangzhou | 202301195 |
| Qinghai Wang | The First People's Hospital of Changde City | Changde | 2022-264-05 |
| Hong Li | Shunde Hospital of Southern Medical University | Foshan | 20240110 |
| Dajun Wu | Zhuzhou Central Hospital | Zhuzhou | ZZCHGCPEC2022026-05 |
| Yali Zong | The Ninth Hospital of Nanchang | Nanchang | JYLHS-2024.01/X-2022.YWSY.02 |
| Yuehong Zeng | Yiyang Central Hospital | Yiyang | 2024003 |
| Hongjian Wei | The Third People's Hospital of Hunan Province | Yueyang | EC-20230613-01 |
| Huili Wu | Zhengzhou Central Hospital | Zhengzhou | 2024-006-01 |
| Xuanfang Zhong | Huizhou First Hospital | Huizhou | HZFHIRB-2024-001-01 |
| Fei Shi | Shenzhen People's Hospital | Shenzhen | SYL-2024-002-01 |
| Jiawei Geng | The First People's Hospital of Yunnan Province | Kunming | KHLL2024-006 |
| Junyi Li | The Third People’s Hospital of Kunming city | Kunming | 2024-GL001 |
| Yingmei Tang | The Second Affiliated Hospital of Kunming Medical University | Kunming | PJ-2024-3 |
| Yawen Luo | Affiliated Hospital of Zunyi Medical University | Zunyi | 2024007-1 |
| Xinhua Luo | Guizhou Provincial People's Hospital | Guiyang | 2024-018 |
| Guicheng Wu | Chongqing University Three Gorges Hospital | Chongqing | (2024) - (388) |
| Zonghua Chen | The Second People's Hospital of Yibin | Yibin | 2024-007-01 |
| Jianyong Chen | Jiangxi Provincial People's Hospital | Nanchang | 2024LLYJ18 |
| Xiaoping Wu | The First Affiliated Hospital of Nanchang University | Nanchang | 2024-033 |
| Shuilin Sun | The Second Affiliated Hospital of Nanchang University | Nanchang | 【2024】(04) |
| Jun Xie | First Affiliated Hospital of Gannan Medical University | Ganzhou | (2024)043 |
| Yueming Shen | Changsha Central Hospital | Changsha | 2024-008 |
| Fangfang Li | First People's Hospital of Chenzhou | Chenzhou | 2024007H |
| Xiongxiang Liu | The Central Hospital of Xiangtan | Xiangtan | 2024-01-17-1 |
| Fengqian Song | Loudi Central Hospital | Loudi | 2024-01-01 |
| Yongzhong Li | Hunan University of Medicine General Hospital | Huaihua | YW-2024020202 |
| Heng Zhang | The Central Hospital of Wuhan | Wuhan | 2024-028-01 |
| Zhongji Meng | Taihe Hospital | Shiyan | 2024001-(01) |
| Xueqiang Jiang | Sinopharm Dongfeng General Hospital | Shiyan | ZC-2024-EC-01.01 |
| Caiyan Zhao | Hebei Medical University Third Hospital | Shijiazhuang | 2024-009-1 |
| Wenfang Yuan | Shijiazhuang Fifth Hospital | Shijiazhuang | 【2024】-YW-001-1 |
| Guoqiang Zhang | Luoyang Central Hospital | Luoyang | 2024-YW-010-(1) |
| Mingqin Lu | The First Affiliated Hospital of Wenzhou Medical University | Wenzhou | 2024-047 |
| Liang Hong | The Third Affiliated Hospital of Wenzhou Medical University | Wenzhou | YW2024001-SY-01 |
| Hui Tian | Xuancheng People's Hospital | Xuancheng | 2024-006-01 |
| Lihua Zhong | The Fourth Affiliated Hospital of Harbin Medical University | Harbin | 2024-008-01 |
| Hongbo Ji | Luoyang Third People's Hospital (now Luoyang First People’s Hospital) | Luoyang | 2024-01-001-H01 |
| Chenwei Pan | The Second Affiliated Hospital and Yuying Children's Hospital of Wenzhou Medical University | Wenzhou | 2024-Y-11-01 |
| Jie Pan | Wenzhou Central Hospital | Wenzhou | 2024-010 |
| Xiaoqu Zhu | Wenzhou Traditional Chinese Medicine Hospital of Zhejiang Chinese Medical University | Wenzhou | WZY2024-YW-002-01 |
| Cheng Wo | Jiujiang Third People's Hospital | Jiujiang | IEC-2024-002(YW)-01 |
| Jinwen Gong | Jiujiang NO.1 People's Hospital | Jiujiang | JJSDYRMYY-LLHS-2024-010 |
| Baolian Shu | Central Hospital of Hengyang | Hengyang | 2024HS [005] |
| Shangyuan Cai | The First Affiliated Hospital of Henan University | Kaifeng | 2024-01-011-H01 |
| Bin Chen | The First Hospital of Hunan University of Chinese Medicine | Changsha | HN-LL-2024-046-01 |
| Zhanzhou Lin | Huizhou Central People's Hospital | Huizhou | LLHS2024109-01 |
| Aifeng Yang | Puyang Oilfield General Hospital | Puyang | 2024-01-0035-F01 |
| Xiaolin Zhou | Yichang Central People's Hospital | Yichang | 2024-213-01 |
| Ming Zhao | Zhoukou Central Hospital | Zhoukou | GCP-2024-YW018-01 |

**Table S2. Results of the intention-to-treat (ITT) analysis for the primary endpoint***

| **Changes from baseline in serum ALB levels (g/L)** | | |
| --- | --- | --- |
|  | **rHSA Group**  **(N = 195)** | **pHSA Group**  **(N = 195)** |
| LSM (SE) | 11.12 (0.372) | 10.86 (0.402) |
| LSM difference (95% CI) | 0.26 (-0.71, 1.23) | |
| * Plus-minus values are LSM ± SE. LSM, least-squares mean; SE, standard error.  Central laboratory measurements were used for the primary endpoint. For the 3 patients who did not receive study medication after randomization (1 in the rHSA group and 2 in the pHSA group), baseline albumin (ALB) values were imputed using local laboratory results at randomization, and the change from baseline at treatment completion was set to 0.  An analysis of covariance (ANCOVA) model was used, with the change in serum ALB levels measured by the central laboratory from baseline to immediately after the final administration as the dependent variable, treatment group as the fixed effect, and baseline serum ALB levels, baseline ascites depth (> 0 to < 3 cm; ≥ 3 to < 6 cm; ≥ 6 to ≤ 10 cm), and baseline diuretic use (yes/no) as covariates. | | |

# **Table S3. Results of the sensitivity analysis 1 (Full Analysis Set) for the primary endpoint***

| **Changes from baseline in serum ALB levels (g/L)** | | |
| --- | --- | --- |
|  | **rHSA Group (N = 194)** | **pHSA Group**  **(N = 193)** |
| LSM (SE) | 11.39 (0.345) | 11.24 (0.390) |
| LSM difference (95% CI) | 0.15 (-0.89, 1.18) | |
| *Plus-minus values are LSM ± SE. LSM, least-squares mean; SE, standard error.  An analysis of variance (ANOVA) model was used, with the change in serum ALB levels measured by the central laboratory from baseline to immediately after the final administration as the dependent variable, and treatment group as the fixed effect. | | |

# **Table S4. Results of the sensitivity analysis 2 (Full Analysis Set) for the primary endpoint***

| **Changes from baseline in serum ALB levels (g/L)** | | |
| --- | --- | --- |
|  | **rHSA Group**  **(N = 194)** | **pHSA Group (N = 193)** |
| LSM (SE) | 11.42 (0.317) | 11.21 (0.365) |
| LSM difference (95% CI) | 0.21 (-0.75, 1.17) | |
| * Plus-minus values are LSM ± SE. LSM, least-squares mean; SE, standard error.  An analysis of covariance (ANCOVA) model was used, with the change in serum ALB levels measured by the central laboratory from baseline to immediately after the final administration as the dependent variable, treatment group as the fixed effect, and baseline serum ALB levels as a continuous covariate. | | |

# **Table S5. Results of the supplementary analysis 1 (Full Analysis Set) for the primary endpoint***

| **Changes from baseline in serum ALB levels (g/L)** | | |
| --- | --- | --- |
|  | **rHSA Group (N = 194)** | **pHSA Group (N = 193)** |
| LSM (SE) | 11.34 (0.328) | 11.31 (0.331) |
| LSM difference (95% CI) | 0.03 (-0.77, 0.83) | |
| * Plus-minus values are LSM ± SE. LSM, least-squares mean; SE, standard error.  Intercurrent events (premature discontinuation, changes to diuretic therapy, and use of prohibited medications/albumin/blood products) were handled using a hypothetical strategy, in which post-event efficacy data were excluded from the analysis and missing data were not imputed.  An analysis of covariance (ANCOVA) model was used, with the change in serum ALB levels measured by the central laboratory from baseline to immediately after the last administration as the dependent variable, treatment group as the fixed effect, and baseline serum ALB levels, baseline ascites depth (> 0 to < 3 cm; ≥ 3 to < 6 cm; ≥ 6 to ≤ 10 cm), and baseline diuretic use (yes/no) as covariates. | | |

# **Table S6. Results of the supplementary analysis 2 (Full Analysis Set) for the primary endpoint***

| **Changes from baseline in serum ALB levels (g/L)** | | |
| --- | --- | --- |
|  | **rHSA Group (N = 194)** | **pHSA Group**  **(N = 193)** |
| LSM (SE) | 11.27 (0.325) | 11.13 (0.328) |
| LSM difference (95% CI) | 0.14 (-0.66, 0.93) | |
| * Plus-minus values are LSM ± SE. LSM, least-squares mean; SE, standard error.  Intercurrent events (premature discontinuation, changes to diuretic therapy, and use of prohibited medications/albumin/blood products) were handled using a treatment policy strategy, in which post-event efficacy data were included in the analysis.  An analysis of covariance (ANCOVA) model was used, with the change in serum ALB levels measured by the central laboratory from baseline to immediately after the final administration as the dependent variable, treatment group as the fixed effect, and baseline serum ALB levels, baseline ascites depth (> 0 to < 3 cm; ≥ 3 to < 6 cm; ≥ 6 to ≤ 10 cm), and baseline diuretic use (yes/no) as covariates. | | |

# **Table S7. Results of the supplementary analysis 3 (****Per-protocol Set) for the primary endpoint***

| **Changes from baseline in serum ALB levels (g/L)** | | |
| --- | --- | --- |
|  | **rHSA Group**  **(N = 184)** | **pHSA Group (N = 181)** |
| LSM (SE) | 11.39 (0.325) | 11.35 (0.326) |
| LSM difference (95% CI) | 0.04 (-0.76, 0.84) | |
| * Plus-minus values are LSM ± SE. LSM, least-squares mean; SE, standard error.  Intercurrent events were handled per the primary analysis strategy.  An analysis of covariance (ANCOVA) model was used, with the change in serum ALB levels measured by the central laboratory from baseline to immediately after the last administration as the dependent variable, treatment group as the fixed effect, and baseline serum ALB levels, baseline ascites depth (> 0 to < 3 cm; ≥ 3 to < 6 cm; ≥ 6 to ≤ 10 cm), and baseline diuretic use (yes/no) as covariates. | | |

# **Table S8. Results of the sensitivity analysis (Full Analysis Set, without data imputation) for the primary endpoint***

| **Changes from baseline in serum ALB levels (g/L)** | | |
| --- | --- | --- |
|  | **rHSA Group (N = 194)** | **pHSA Group (N = 193)** |
| LSM (SE) | 11.29 (0.326) | 11.36 (0.327) |
| LSM difference (95% CI) | -0.07 (-0.87, 0.73) | |
| * Plus-minus values are LSM ± SE. LSM, least-squares mean; SE, standard error.  Intercurrent events handled per the primary analysis strategy.  An analysis of covariance (ANCOVA) model was used, with the change from baseline in serum albumin concentration (confirmed by the albumin test immediately after the completion of the last intravenous administration) as the dependent variable, treatment group as a fixed effect, and baseline serum albumin concentration (continuous outcome), baseline ascites depth (< 3 cm; 3 to < 6 cm; 6 to 10 cm) (IWRS), and baseline diuretic use (Yes; No) (IWRS) as covariates. | | |

# **Table S9. Results of the sensitivity analysis (Full Analysis Set, without data imputation) for the primary endpoint***

| **Changes from baseline in serum ALB levels (g/L)** | | |
| --- | --- | --- |
|  | **rHSA Group (N = 194)** | **pHSA Group (N = 193)** |
| LSM (SE) | 11.55 (0.320) | 11.62 (0.323) |
| LSM difference (95% CI) | -0.07 (-0.96, 0.83) | |
| * Plus-minus values are LSM ± SE. LSM, least-squares mean; SE, standard error.  Intercurrent events handled per the primary analysis strategy; no missing data imputation. An analysis of variance (ANOVA) model was used, with the change from baseline in serum albumin (confirmed immediately post-last IV dose) as the dependent variable; treatment as the fixed effect. | | |

# **Table S10. Results of the sensitivity analysis 2 (Full Analysis Set, without data imputation) for the primary endpoint***

| **Changes from baseline in serum ALB levels (g/L)** | | |
| --- | --- | --- |
|  | **rHSA Group (N = 194)** | **pHSA Group (N = 193)** |
| LSM (SE) | 11.55 (0.286) | 11.62 (0.288) |
| LSM difference (95% CI) | -0.07 (-0.87, 0.73) | |
| * Plus-minus values are LSM ± SE. LSM, least-squares mean; SE, standard error.  Intercurrent events handled per the primary analysis strategy; no missing data imputation. An analysis of covariance (ANCOVA) model was used, with the change from baseline in serum albumin (confirmed immediately post-last IV dose) as the dependent variable; treatment as the fixed effect; and baseline serum albumin (continuous) as covariate. | | |

# **Table S11. Results of the male subgroup analysis (Full Analysis Set) for the primary endpoint***

| **Changes from baseline in serum ALB levels (g/L)** | | |
| --- | --- | --- |
|  | **rHSA Group**  **(N = 133)** | **pHSA Group (N = 148)** |
| LSM (SE) | 11.12 (0.413) | 10.48 (0.425) |
| LSM difference (95% CI) | 0.64 (-0.44, 1.72) | |
| * Plus-minus values are LSM ± SE. LSM, least-squares mean; SE, standard error.  Intercurrent events were handled per the primary analysis strategy.  An analysis of covariance (ANCOVA) model was used, with the change in serum ALB levels measured by the central laboratory from baseline to immediately after the final administration as the dependent variable, treatment group as the fixed effect, and baseline serum ALB levels, baseline ascites depth (> 0 to < 3 cm; ≥ 3 to < 6 cm; ≥ 6 to ≤ 10 cm), and baseline diuretic use (yes/no) as covariates. | | |

# **Table S12. Results of the intention-to-treat (ITT) analysis for the key secondary endpoint**

|  | **rHSA Group**  **(N = 195)** | **pHSA Group**  **(N = 195)** |
| --- | --- | --- |
| Patients with Ascites Improvement — no. (%) | 113 (57.7) | 94 (48.2) |
| rHSA Group *vs.* pHSA Group difference (95% CI) | 9.7 (-0.31, 19.35) | |
| For the 3 patients who did not receive treatment after randomization (1 in the rHSA group and 2 in the pHSA group), baseline ALB values were imputed using local laboratory results at randomization, and these patients were classified as non-responders. | | |

**Table S13 Diuretic Use (Full Analysis Set)**

| **Baseline** | **rHSA 20g/day**  **N=194**  **n (%)** | **pHSA 20g/day**  **N=193**  **n (%)** | **Treatment** | **rHSA 20g/day**  **N=194**  **n (%)** | **pHSA 20g/day**  **N=193**  **n (%)** | **Follow-up** | **rHSA 20g/day**  **N=194**  **n (%)** | **pHSA 20g/day**  **N=193**  **n (%)** |
| --- | --- | --- | --- | --- | --- | --- | --- | --- |
| **At least one diuretic use** | **153 (78.9)** | **151 (78.2)** | **At least one diuretic use** | **19 (9.8)** | **26 (13.5)** | **At least one diuretic use** | **84 (43.3)** | **80 (41.5)** |
| **Spironolactone** | **145 (74.7)** | **145 (75.1)** | **Furosemide** | **13 (6.7)** | **17 (8.8)** | **Furosemide** | **68 (35.1)** | **68 (35.2)** |
| Oral | 145 (74.7) | 145 (75.1) | Oral | 9 (4.6) | 11 (5.7) | Oral | 57 (29.4) | 59 (30.6) |
| Mean total dose (mg) | 252.8 | 277.02 | Mean total dose (mg) | 108.89 | 110 | Mean total dose (mg) | 1218.06 | 928.9 |
| **Furosemide** | **132 (68.0)** | **136 (70.5)** | Intravenous (IV) | 6 (3.1) | 9 (4.7) | Intravenous (IV) | 20 (10.3) | 27 (14.0) |
| Oral | 117 (60.3) | 119 (61.7) | Mean total dose (mg) | 103.75 | 55 | Mean total dose (mg) | 63.91 | 98.78 |
| Mean total dose (mg) | 116.06 | 117.38 | Subcutaneous Injection | 0 | 1 (0.5) | Subcutaneous Injection | 1 (0.5) | 0 |
| Intravenous (IV) | 36 (18.6) | 36 (18.7) | Mean total dose (mg) | 0 | 160 | Mean total dose (mg) | 20 | 0 |
| Mean total dose (mg) | 63.18 | 76.59 | **Spironolactone** | **10 (5.2)** | **7 (3.6)** | **Spironolactone** | **58 (29.9)** | **63 (32.6)** |
| **Torasemide** | **12 (6.2)** | **8 (4.1)** | Oral | 10 (5.2) | 7 (3.6) | Oral | 58 (29.9) | 63 (32.6) |
| Intravenous (IV) | 9 (4.6) | 6 (3.1) | Mean total dose (mg) | 188 | 240 | Mean total dose (mg) | 2398.27 | 1978.68 |
| Mean total dose (mg) | 36 | 43.33 | **Torasemide** | **3 (1.5)** | **5 (2.6)** | **Torasemide** | **5 (2.6)** | **13 (6.7)** |
| Oral | 4 (2.1) | 3 (1.6) | Intravenous | 2 (1.0) | 4 (2.1) | Oral | 4 (2.1) | 4 (2.1) |
| Mean total dose (mg) | 50 | 50 | Mean total dose (mg) | 10 | 41.67 | Mean total dose (mg) | 424 | 160 |
| **Tolvaptan** | **3 (1.5)** | **10 (5.2)** | Oral | 1 (0.5) | 1 (0.5) | Intravenous | 2 (1.0) | 10 (5.2) |
| Oral | 3 (1.5) | 10 (5.2) | Mean total dose (mg) | 70 | 40 | Mean total dose (mg) | 106.67 | 42.27 |
| Mean total dose (mg) | 50 | 42.95 | **Bumetanide** | **0** | **2 (1.0)** | **Tolvaptan** | **3 (1.5)** | **7 (3.6)** |
| **Bumetanide** | **2 (1.0)** | **3 (1.6)** | Intravenous | 0 | 2 (1.0) | Oral | 3 (1.5) | 7 (3.6) |
| Intravenous (IV) | 2 (1.0) | 2 (1.0) | Mean total dose |  | 2 | Mean total dose (mg) | 294.38 | 210 |
| Mean total dose (mg) | 2.5 | 1.33 | **Tolvaptan** | **0** | **1 (0.5)** | **Bumetanide** | **2 (1.0)** | **4 (2.1)** |
| Oral | 1 (0.5) | 1 (0.5) | Oral | 0 | 1 (0.5) | Intravenous | 1 (0.5) | 3 (1.6) |
| Mean total dose (mg) | 2 |  | Mean total dose (mg) |  | 60 | Mean total dose (mg) | 2 | 5.63 |
| **Hydrochlorothiazide** | **0** | **2 (1.0)** |  |  |  | Oral | 1 (0.5) | 1 (0.5) |
| Oral | 0 | 2 (1.0) |  |  |  | Mean total dose (mg) | 14 | 38 |
| Mean total dose (mg) | 0 | 200 |  |  |  | **Fructose; Glycerol; Sodium Chloride** | **0** | **1 (0.5)** |
|  |  |  |  |  |  | Intravenous | 0 | 1 (0.5) |
|  |  |  |  |  |  | Mean total dose (mg) | 0 | 100000 |
|  |  |  |  |  |  | **Hydrochlorothiazide** | **0** | **1 (0.5)** |
|  |  |  |  |  |  | Oral | 0 | 1 (0.5) |
|  |  |  |  |  |  | Mean total dose (mg) | 0 | 800 |
| Baseline diuretic is defined as any diuretic use within 3 days prior to randomization.  Treatment-emergent diuretic is defined as any diuretic initiated from the first dose of study drug (including the day of dosing) through Day 7 or End of Treatment (EOT) (including the day of EOT).  Follow-up diuretic is defined as any diuretic initiated after Day 7 or EOT.  Diuretics are coded using WHODrug Global (March 2025 version).  Percentages are based on the number of subjects in each treatment group within the Full Analysis Set (FAS). | | | | | | | | |

# **Table S14: Changes from baselines in body weight and abdominal circumference, and the PCOP at the final intravenous administration**

|  | **rHSA Group** | **pHSA Group** |
| --- | --- | --- |
| **body weight** |  |  |
| n | 191 | 191 |
| Mean (SD), kg | -1.28 (2.282) | -1.04 (2.548) |
|  |  |  |
| **abdominal circumference** |  |  |
| n | 191 | 192 |
| Mean (SD), cm | -2.12 (3.617) | -1.71 (3.851) |
|  | | |
| **PCOP** |  |  |
| n | 189 | 192 |
| Mean (SD), mmHg | 4.27 (2.828) | 4.00 (2.910) |

# **Table S15. Changes in serum ALB levels from retreatment baselines after the final administration (Retreatment Analysis Set)***

| **Changes from per-retreatment baseline in serum ALB levels** | | |
| --- | --- | --- |
|  | **rHSA Group**  **(N = 25)** | **pHSA Group (N = 20)** |
| n | 14 | 15 |
| LSM (SE) | 10.830 (0.6226) | 9.835 (0.6010) |
| LSM difference (95% CI) | 0.994 (-0.806, 2.795) | |
| * Plus-minus values are LSM ± SE. LSM, least-squares mean; SE, standard error.  Pre-re-treatment baseline was defined as the last non-missing assessment prior to the first dose of the investigational product in the re-treatment period. This analysis was applicable only to patients who received the investigational product again during Days 29–76.  Analysis was based on observed data (missing data were not imputed).  An analysis of covariance (ANCOVA) model was used, with the change from the pre-re-treatment baseline in serum ALB levels (measured immediately after the last intravenous dose during the re-treatment period) as the dependent variable, treatment group as the fixed effect, and the pre-re-treatment baseline serum ALB levels as a continuous covariate. | | |

# **Table S16. Glycated hemoglobin after treatment (Full Analysis Set)**

|  | **rHSA Group** | **pHSA Group** |
| --- | --- | --- |
| baseline |  |  |
| n | 193 | 192 |
| Mean (SD), % | 5.189 (1.3142) | 5.190 (1.1682) |
|  | | |
| D29 |  |  |
| n | 188 | 185 |
| Mean (SD), % | 5.223 (1.1832) | 5.155 (1.0628) |
|  | | |
| D90 |  |  |
| n | 183 | 180 |
| Mean (SD), % | 5.503 (1.4962) | 5.285 (1.1013) |

# **Table S17. Summary of infusion reactions by the MedDRA System Organ Class and Preferred Term (Safety Analysis Set)**

| **System Organ Class/**  **Preferred Term** | **rHSA Group**  **(N = 194) n (%)** | **pHSA Group (N = 193) n (%)** |
| --- | --- | --- |
| **Infusion-related AEs** | **3 (1.5)** | **1 (0.5)** |
|  | | |
| **General disorders and administration site conditions** | **2 (1)** | **0** |
| Fever | 1 (0.5) | 0 |
| Infusion site pain | 1 (0.5) | 0 |
| **Injury, poisoning and procedural complications** | **1 (0.5)** | **0** |
| Infusion-related reaction | 1 (0.5) | 0 |
| **Immune system disorders** | 0 | 1 (0.5) |
| Hypersensitivity reaction | 0 | 1 (0.5) |
| Adverse events were coded using MedDRA v28.0.  The calculation of percentages is based on the number of patients in each treatment group within the Safety Analysis Set (SS). | | |

# **Table S18. Summary of serious related adverse events by the MedDRA System Organ Class and Preferred Term (Safety Analysis Set)**

| **System Organ Class/**  **Preferred Term** | **rHSA Group**  **(N = 194) n (%)** | **pHSA Group (N = 193) n (%)** |
| --- | --- | --- |
| **Serious related adverse events** | **1 (0.5)** | **1 (0.5)** |
|  | | |
| **Injury, poisoning and procedural complications** | **1 (0.5)** | **0** |
| Infusion-related reactions | 1 (0.5) | 0 |
| **Gastrointestinal disorders** | **0** | **1 (0.5)** |
| Esophageal varices hemorrhage | 0 | 1 (0.5) |
| Adverse events were coded using MedDRA v28.0.  The calculation of percentages is based on the number of patients in each treatment group within the Safety Analysis Set. | | |

# **Table S19. Summary of adverse events leading to death by the MedDRA System Organ Class and Preferred Term (Safety Analysis Set)**

| **System Organ Class/**  **Preferred Term** | **rHSA Group (N = 194) n (%)** | **pHSA Group (N = 193) n (%)** |
| --- | --- | --- |
| **Death** | **5 (2.6)** | **7 (3.6)** |
| **Gastrointestinal disorders** | **3 (1.5)** | **2 (1.0)** |
| Upper gastrointestinal hemorrhage | 1 (0.5) | 1 (0.5) |
| Esophageal varices hemorrhage | 1 (0.5) | 0 |
| Gastrointestinal hemorrhage | 1 (0.5) | 1 (0.5) |
| **Hepatobiliary disorders** | **1 (0.5)** | **1 (0.5)** |
| Liver cirrhosis | 1 (0.5) | 0 |
| Acute-on-chronic liver failure | 0 | 1 (0.5) |
| **Infections and infestations** | **1 (0.5)** | **1 (0.5)** |
| Septic shock | 1 (0.5) | 0 |
| Pulmonary tuberculosis | 0 | 1 (0.5) |
| Infectious pneumonia | 0 | 1 (0.5) |
| **Nervous system disorders** | **1 (0.5)** | **2 (1.0)** |
| Hepatic encephalopathy | 1 (0.5) | 1 (0.5) |
| Intracerebral hemorrhage | 0 | 1 (0.5) |
| **Vascular and lymphatic disorders** | **1 (0.5)** | **0** |
| Haemorrhagic shock | 1 (0.5) | 0 |
| **Respiratory, thoracic and mediastinal disorders** | **0** | **1 (0.5)** |
| Chronic obstructive pulmonary disease | 0 | 1 (0.5) |
| **Cardiac disorders** | **0** | **1 (0.5)** |
| Acute coronary syndrome | 0 | 1 (0.5) |
| Adverse events were coded using MedDRA v28.0.  The calculation of percentages is based on the number of patients in each treatment group within the Safety Analysis Set. | | |
